# Synergistic Pd/Cu-catalyzed enantioselective Csp^2^–F bond alkylation of fluoro-1,3-dienes with aldimine esters

**DOI:** 10.1038/s41467-022-30152-7

**Published:** 2022-05-05

**Authors:** Huimin Yu, Qinglong Zhang, Weiwei Zi

**Affiliations:** 1 State Key Laboratory and Institute of Elemento-Organic Chemistry, College of Chemistry, Nankai University, Tianjin, 300071 China; 2 Haihe Laboratory of Sustainable Chemical Transformations, Tianjin, 300071 China

**Keywords:** Asymmetric synthesis, Organic chemistry

## Abstract

Due to high bond dissociation energies of Csp^2^–F bonds, using fluorinated compounds in Csp^2^–Csp^3^ cross-coupling is difficult. Here the authors report a protocol for enantioselective Csp^2^–Csp^3^ coupling of dienyl fluorides with aldimine esters, enabled by synergistic copper and palladium catalysis. This reaction represents the first example of asymmetric Csp^2^–Csp^3^ cross-coupling involving an inert Csp^2^–F bond and provides expeditious access to chiral α-alkenyl α-amino acids with high enantioselectivity. Control experiments suggest that the Csp^2^–F bond activation occurs through a pathway involving PdH migratory insertion and subsequent allylic defluorination, rather than by direct oxidative addition of the Csp^2^–F bond to Pd(0). The detailed mechanism is further investigated by DFT calculation and the enantioselectivity is rationalized.

## Abstract (web summary)

Due to high bond dissociation energies of C(sp2)–F bonds, using fluorinated compounds in C(sp2)–C(sp3) cross-coupling is difficult. Here the authors report a protocol for enantioselective C(sp2)–C(sp3) coupling of dienyl fluorides with aldimine esters, enabled by synergistic copper and palladium catalysis.

## Introduction

**Fig. 1:**
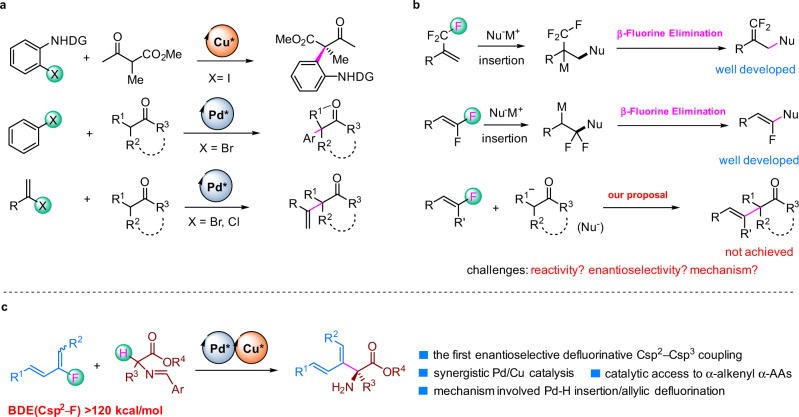
Transition-metal-catalyzed asymmetric Csp^2^–Csp^3^ cross-coupling reactions. **a** Enantioselective Csp^2^–Csp^3^ cross-coupling of Csp^2^–I (Br, Cl) Bonds. **b** C–F bond Activation by Nu-M Insertion/β-fluorine elimination. **c** This work: C–F bond activation by Pd-H insertion/allylic defluorination for Csp^2^–Csp^3^ cross-coupling.

Transition-metal-catalyzed enantioselective cross-coupling reactions between Csp^2^–X compounds (X = halogen) and enolizable carbonyl compounds are commonly used transformations for asymmetric construction Csp^2^–Csp^3^ bonds^1–7^. Many successful examples of this method have been reported, including pioneering work by the research groups of Ma^1^, Hartwig^2^, and Buchwald^3,6^, who used chiral-ligand-bearing transition metals such as Cu and Pd to achieve enantiocontrol (Fig. 1a). These reactions initiated with oxidative addition of Csp^2^–X bond to the Pd(0) or Cu(I), followed by ligand exchange and reductive elimination to form the Csp^2^–Csp^3^ bond. Halogenated compounds with Csp^2^–I, Csp^2^–Br, and even Csp^2^–Cl bonds are suitable reaction substrates. However, because Csp^2^–F bonds have high energies (120–129 kcal/mol for olefinic C–F bonds), fluorinated compounds have rarely been used as coupling partners^8–15^.

Recently, C–F activation has been an important research topic in synthetic organic chemistry^16–20^. One of the most successful strategies in this area is using transition-metal-mediated or -catalyzed metal-Nu insertion/β–F elimination process^21^. Trifluoro-, difluoroalkenes have been intensively investigated for this purpose during the past few years^22–30^; however, monofluoroalkenes are rarely explored class of compounds for similar C–F activation reactions (Fig. 1b). Moreover, despite those achievements in defluorinative carbon–carbon and carbon–heteroatom bond formation, enantioselective variants have seldom been realized. We envisioned that if an ingenious insertion/β–F elimination mechanism was designed together with a suitable chiral induction strategy, the aforementioned challenged defluorinative Csp^2^–Csp^3^ coupling might be achieved.

In this work, we report a Cu/Pd cooperative system^31^ for enantioselective Csp^2^–Csp^3^ cross-coupling between dienyl fluorides and aldimine esters (Fig. 1c). Experimental and computational studies revealed that this reaction involved a unique Pd-H insertion/allylic defluorination process. This work not only represents the first example of enantioselective defluorinative Csp^2^–Csp^3^ coupling but also provides a highly efficient catalytic method to prepare chiral α-alkenyl α-amino acids (α-AAs), which are important synthetic targets^32–36^ because of their potential biochemical and pharmacological activities^37^.

## Results and discussion

### Reaction development

Our studies begin with investigating the reaction of dienyl fluoride *E*-**1a** with aldimine ester^38–40^ **2a** to generate α-alkenyl, α-methyl α-AA **3aa**. Using the stereocontrol exhibited by Cu-azomethine ylides in two-metal catalytic systems^41–43^, we designed a synergistic Pd/Cu catalyst system^44–55^ for controlling the stereochemistry of the newly formed chiral center by means of an appropriate combination of ligands on the two metals during the coupling step (Table 1). First, we tested Phosferrox Cu complex L1-Cu with Pd catalysts bearing a bisphosphine ligand (dppe, dppp, Xantphos, or DPEphos; **L4-Pd**–**L7-Pd**, respectively) and found that none of these combinations catalyzed the desired reaction (entries 1–4). In contrast, BINAP-ligated catalyst **L8-Pd** afforded **3aa** in 53% yield with 96% ee (entry 5). SEGPHOS- and Biphep-derived catalysts (**L9-Pd**–**L11-Pd**, entries 6–8) were also examined, and **L11-Pd** gave the best yield of the product. Subsequent tests of **L11-Pd** in combination with other Cu catalysts (**L2-Cu** and **L3-Cu**, entries 9 and 10) revealed that **L11-Pd**/**L3-Cu** gave the best results (86% yield, 99% ee). A slight decrease in enantioselectivity was observed when the opposite enantiomer of the Pd catalyst (*ent***-L11-Pd**) was used together with **L3-Cu** (entry 11). This result implies the enantioselectivity was mainly controlled by the chiral Cu catalyst but the mismatched chirality between the two catalysts was slightly deleterious to the enantiocontrol.

**Table 1:** Optimization of catalyst system for the cross-coupling reaction.

Entry^a^	Pd catalyst	Cu catalyst	Yield [%]^b^	ee [%]^c^
1	**L4-Pd**	**L1-Cu**	<5	*n.d.*
2	**L5-Pd**	**L1-Cu**	<5	*n.d.*
3	**L6-Pd**	**L1-Cu**	<5	*n.d.*
4	**L7-Pd**	**L1-Cu**	<5	*n.d.*
5	**L8-Pd**	**L1-Cu**	53	96
6	**L9-Pd**	**L1-Cu**	50	98
7	**L10-Pd**	**L1-Cu**	73	99
8	**L11-Pd**	**L1-Cu**	87	96
9	**L11-Pd**	**L2-Cu**	75	98
10	**L11-Pd**	**L3-Cu**	86	99
11	*ent*-**L11-Pd**	**L3-Cu**	82	93
12	**L11-Pd**	—	*n.r.*	*n.d.*
13	—	**L3-Cu**	*n.r.*	*n.d.*

*n.d.* not determined, *n.r.* no reaction.

^a^Reaction conditions: (i) **1a** (0.2 mmol), **2a** (0.1 mmol), Pd catalyst (4 mol%), Cu catalyst (5 mol%), Et_3_N (200 mol%), THF (0.5 mL), 30 °C, 24 h; (ii) citric acid (10 wt%, 4 mL).

^b^Isolated yields are provided.

^c^The ee values were determined by HPLC using a column with a chiral stationary phase.

### Substrate scope

**Fig. 2:**
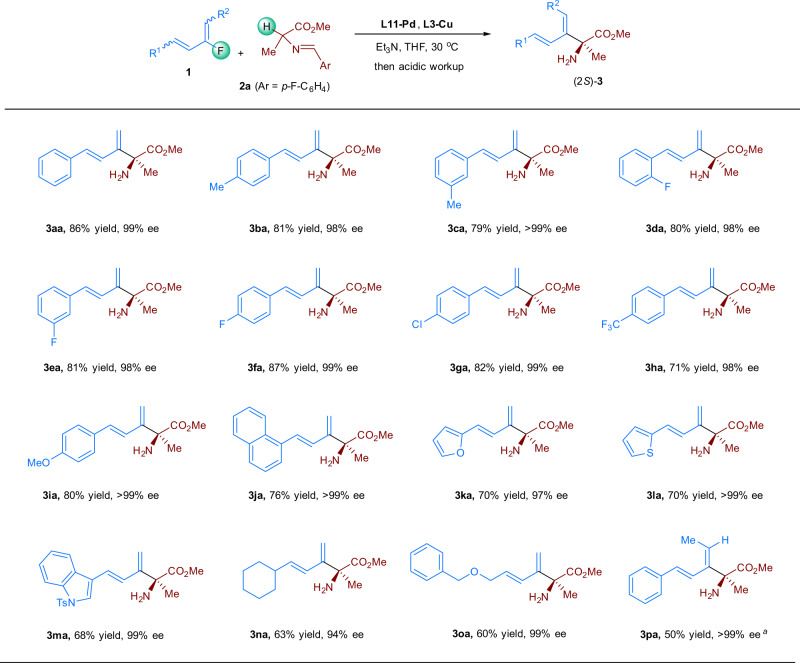
Substrate scope with respect to the dienyl fluorides. Reaction conditions: (i) **1a** (0.4 mmol), **2a** (0.2 mmol), **L11-Pd** (4 mol%)**, L3-Cu** (5 mol%), Et_3_N (200 mol%), THF (0.5 mL), 30 °C, 24 h; (ii) citric acid (10 wt%, 4 mL). Isolated yields are provided. The ee values were determined by HPLC on a column with a chiral stationary phase. ^*a*^**L11-Pd** (8 mol%), **L3-Cu** (10 mol%), THF (0.2 mL), 40 °C, 120 h.

Having developed an effective dual-metal catalyst system, we investigated the substrate scope of the reaction, starting with dienyl fluorides **1** bearing various R^1^ and R^2^ substituents (Fig. 2). Phenyl rings with a methyl group (**3ba**, **3ca**), a fluorine atom (**3da**–**3fa**), a chlorine atom (**3ga**), a trifluoromethyl group (**3** **ha**), or a methoxy group (**3ia**) were well tolerated, regardless of the location of the substituent, affording the corresponding coupling products in 71–87% yields with enantioselectivities exceeding 98% ee. Replacing the phenyl ring with a different aromatic ring—naphthyl (**3ja**), furyl (**3ka**), thiophenyl (**3la**), or indolyl (**3ma**)—had little influence on the reaction outcome, the corresponding α-alkenyl, α-alkyl α-AAs were obtained with excellent enantioselectivities. An alkyl-substituted substrate (R^1^ = cyclohexyl) furnished **3na** in 63% yield, albeit with a reduced ee (94%). Even though allylic ethers are commonly sensitive to Pd owing to the possibility of C–O bond cleavage, a substrate with an allylic BnO ether moiety was well tolerated in this reaction system, giving **3oa** in 60% yield with 99% ee. A 1,4-disubstituted dienyl fluoride (R^1^ = Ph, R^2^ = Me) was also investigated, which afforded the desired product **3pa** in 50% yield with >99% ee. To determine the stereochemistry of the product, we converted **3aa** to its *p*-toluenesulfonamide derivative and confirmed its structure by means of X-ray crystallographic analysis, which allowed us to assign the absolute configuration as 2 *S*.

**Fig. 3:**
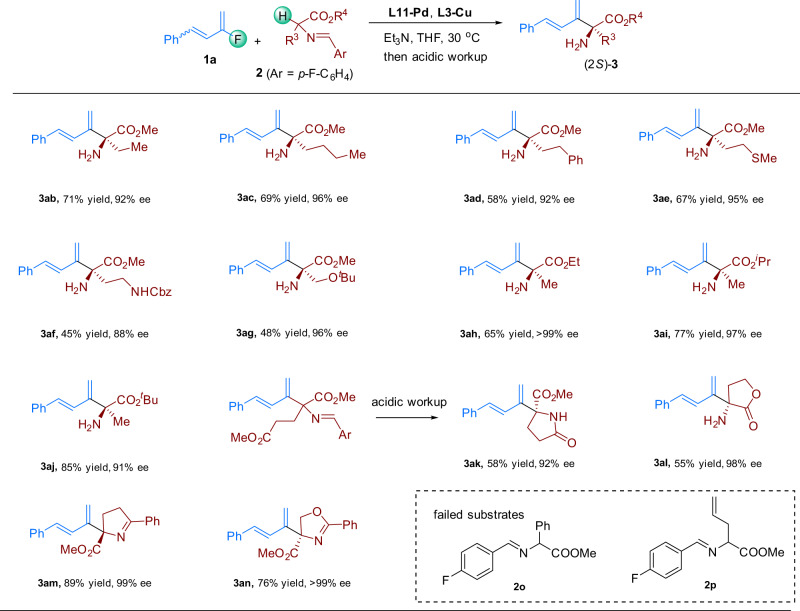
Substrate scope with respect to the aldimine esters. Reaction conditions: (i) **1a** (0.4 mmol), **2a** (0.2 mmol), **L11-Pd** (4 mol%), **L3-Cu** (5 mol%), Et_3_N (200 mol%), THF (0.5 mL), 30 °C, 48 h; (ii) citric acid (10 wt%, 4 mL). Isolated yields are provided. The ee values were determined by HPLC on a column with a chiral stationary phase.

Next, we probed the scope of aldimine esters substrate (Fig. 3). When R^4^ was methyl, R^3^ could be Et (**3ab**), ^*n*^Bu (**3ac**), or phenylethyl (**3ad**). Heteroatom-tethered alkyl chains were also well-tolerated, as indicated by the formation of coupling products **3ae**–**3ag** in moderate to good yields with good enantioselectivities. In addition, we investigated compounds with various alkyl R^4^ groups (**3ah**–**3aj**), revealing that the reaction was not sensitive to the steric bulk of the ester. An aldimine ester derived from glutamic acid also reacted smoothly but gave lactam **3ak** in 58% yield with 92% ee, as a result of cyclization during the acidic workup. α-Amino-γ-butyrolactone derived imine underwent reaction with **1a** to give **3al** in moderate yield. In addition to aldimine ester, cyclic ketimine ester and oxazoline ester were also compatible reaction partners. As shown in the formation of **3am** and **3an**, both the yields and enantioselectivities were well maintained for these types of nucleophiles. Phenylalanine derived aldimine ester **2o** failed to undergo this transformation due to the steric bulk, and substrate **2p** bearing allyl group only gave complexed products, probably because the isomerization of the terminal olefin moiety.

### Synthetic application

**Fig. 4:**
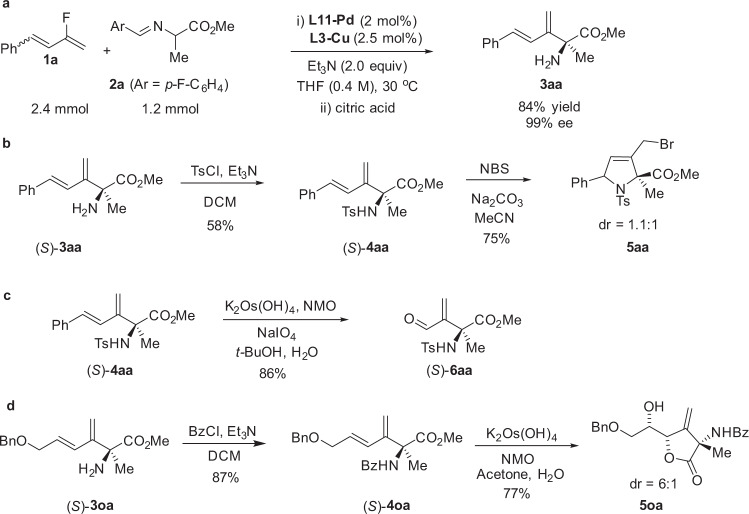
Synthetic application. **a** Scale-up reaction at lower catalyst loading. **b** Intramolecular bromoamination reaction. **c** Selective cleavage of the internal alkene. **d** Dihydroxylation/lactonization reaction.

The reaction was scaled up to more than one mmol scale with a reduced catalyst loading [**L11-Pd** (2 mol%) and **L3-Cu** (2.5 mol%)] and the yield and enantioselectivity were well maintained (Fig. 4a). To demonstrate the utility of the reaction, the coupling products were transformed to other chiral scaffolds. Protection (*S*)-**3aa** with *p*-tolylsulfonyl group gave (*S*)-**4aa**, and the latter was treatment with NBS/Na_2_CO_3_ in acetonitrile to elaborate the bromoamination product **5aa** in 75% yield (Fig. 4b). The internal alkene moiety of (*S*)-**4aa** could be selectively cleaved with K_2_Os(OH)_4_/NMO, followed by NaIO_4_, affording aldehyde (*S*)-**6aa** in 86% yield (Fig. 4c). Moreover, protection the amine of (*S*)-**3oa** with benzoyl group gave (*S*)-**4oa**, which was further subjected to sequential dihydroxylation/lactonization conditions to furnish densely functionalized lactone **5oa** with good diastereoselectivity (Fig. 4d).

### Mechanistic studies

**Fig. 5:**
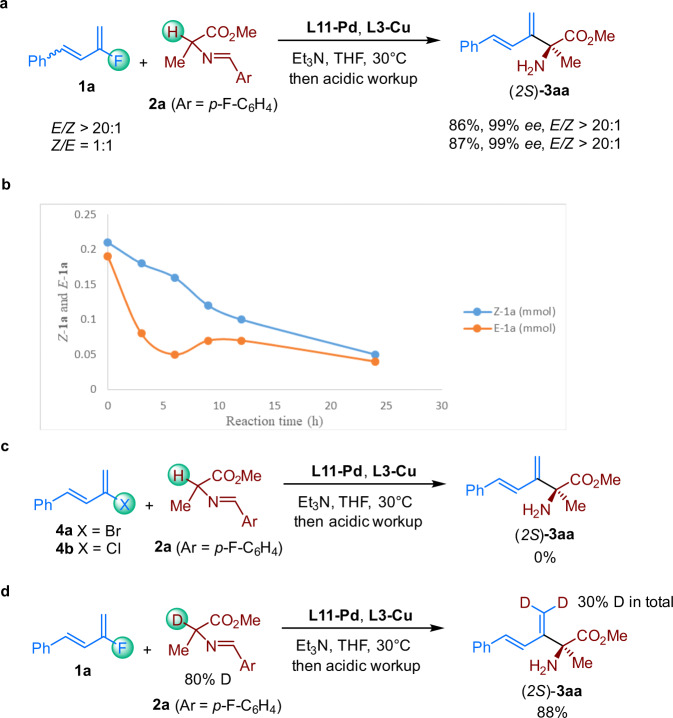
Control experiments. **a** Relationship between the ratio of *Z*/*E*-**1a** and ratio of *Z*/*E*-**3aa**. **b** The absolute amount the *Z-***1a** and *E*-**1a** during the reaction. **c** Reaction of dienyl bromide **4a** or dienyl chloride **4b** under the standard conditions. **d** Deuterium atom scramble experiment.

To gain insight into the mechanism, we carried out some control experiments. We found that reactions of both >20:1 and 1:1 *E*/*Z-***1a** gave >20:1 *E*/*Z-***3aa** with essentially identical yields and ee values (Fig. 5a). On the other hand, when **1a** with the *E/Z* ratio of 1:1.1 was subjected to the reaction, the absolute amount of the *Z*-**1a** and *E*-**1a** was monitored (Fig. 5b). Interestingly, the concentration of *Z*-**1a** and *E*-**1a** are both decreased during the reaction; however, the *E*-**1a** has a faster consumption rate than *Z*-**1a** did. Most importantly, an inflection point appeared around 5 h in the *E*-**1a** consumption curve. These results indicate a possibility that *E*-**1a** preferentially reacted under the optimized conditions at the early stage, and *Z*-**1a** gradually isomerized to *E*-**1a**. In addition, when dienyl bromide **4a** or dienyl chloride **4b** was subjected to the reaction conditions, almost none of the coupling product (**3aa**) was observed (Fig. 5c). Consequently, we reasoned that direct oxidative addition of the Csp^2^–F bond to Pd(0) was probably not involved in the reaction pathway^56,57^. Finally, the reaction between **1a** and deuterium-labeled **2a** resulted in the incorporation of a total 30% of the deuterium at the terminal carbon of the double bond in the coupling product (Fig. 5d). As a result, we speculated that a Pd–D insertion process might be involved in the reaction, which would lead to deuterium enriching at the terminus of the alkenes^58^.

**Fig. 6:**
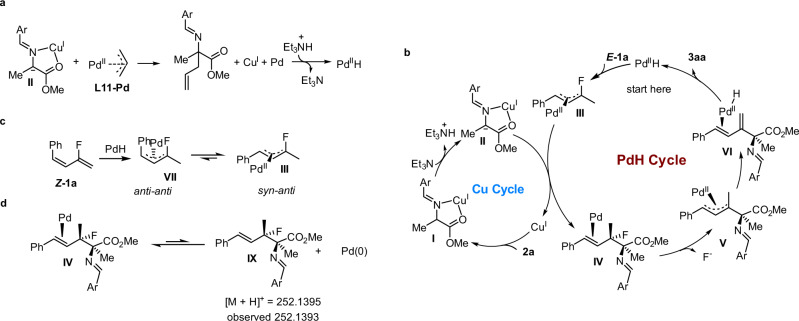
Proposed mechanism. **a** Initial generation of PdH catalyst from the precatalyst **L11-Pd**. **b** Proposed catalytic cycle. **c** Isomerization of *anti*-*anti* π-allyl-Pd to *syn*-*anti* π-allyl-Pd. **d** HRMS detection of [M + H]^+^ for Intermediate IX. The ligands were omitted for clarity.

Based on the above-described results, as well as our previous studies^59,60^ on synergistic Pd/Cu-catalyzed coupling reactions of aldimine esters with unsaturated compounds, we proposed that this coupling reaction proceeds based on a mechanism involving the Cu and PdH cycles shown in Fig. 6. In the Cu catalytic cycle, Cu acts as a Lewis acid to activate aldimine ester **2a** to form metallated azomethine ylide **II**, which serves as a nucleophile in the coupling reaction. Allylation of **II** with pre-palladium catalyst **L11-Pd** forms the **L11-Pd**(0), which then undergoes oxidative addition with Et_3_NH^+^ to generate the PdH catalyst (Fig. 6a). In the PdH catalytic cycle (Fig. 6b), PdH migratory insertion into the C = C bond of the vinyl fluoride moiety of *E*-**1a** generates fluorinated Pd-allyl **III**^61–64^. An allylic substitution reaction between **III** and metallated azomethine ylide **II** affords Pd intermediate **IV** and regenerates the Cu catalyst. Intermediate **IV** undergoes rapid allylic defluorination, giving allyl-Pd **V**, which is converted to **VI** via β-H elimination. Product **3aa** dissociates from **VI**, and the PdH regenerated^65–67^. For *Z*-**1a**, the *anti-anti η*^3^-Pd-allyl **VII** is generated after PdH migratory insertion, which equilibrates to thermodynamically more stable *syn-anti η*^3^-Pd-allyl **III** through *η*^3^-*η*^1^-*η*^3^ isomerization (Fig. 6c). Therefore *Z*-**1a** would then undertake the same catalytic cycle to form the same *E*-type product **3aa**. This proposed mechanism was highly consistent with the controlled experiments in Fig. 3. Moreover, we observed a [M + H]^+^ signal at 252.1393 in the HRMS spectrum of the reaction mixtures. This result indicates the formation of **IX**, which comes from the dissociation of Pd(0) from intermediate **IV** (Fig. 6d).

### Computational studies

**Fig. 7:**
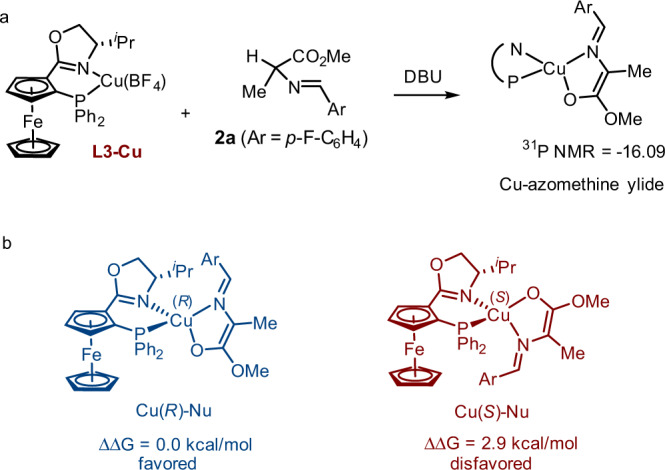
Determination of the structure for the Cu-azomethine ylide. **a** Observation of the Cu-zaomethine ylide by ^31^P NMR. **b** Energy comparison of Cu(*R*)-Nu and Cu(*S*)-Nu.

**Fig. 8:**
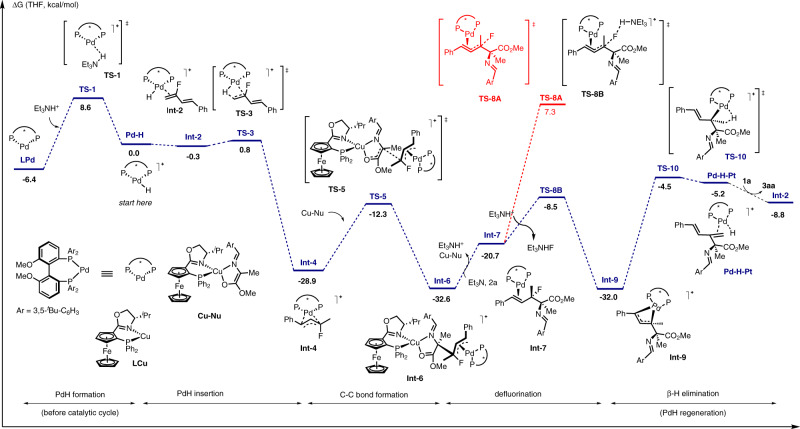
Energy profile for the proposed mechanism. Calculations were carried out at the M06-2x(SMD)/def2-TZVP//B3LYP-D3BJ/6-31 g(d)/Lanl2dz level of theory.

To further understand the mechanism, we performed DFT calculation to investigate the energy profile of this reaction. Stoichiometric reaction of **L3-Cu** and substrate **2a** with DBU, only one isomeric Cu-azomethine ylide was formed, as indicated by a single ^31^P NMR signal at −16.09 (Fig. 7). The structures of Cu-azomethine ylide featuring a (*R*)- or (*S*)- metal chirality were calculated and we found the former is 2.9 kcal/mol stable than the latter. Therefore, we rationalized that Cu(*R*)-Nu rather than Cu(*S*)-Nu was the form for nucleophile and therefore its structure was adopted for the remaining DFT calculation (Fig. 8).

As proposed in Fig. 8, the PdH was initially formed via oxidative protonation of Pd(0) with Et_3_NH^+^. The transition state for this step was located as **TS-1**, which has an energy barrier of 15.0 kcal/mol. After being coordinated by substrate **2a**, the resulting intermediate **Int-2** occurs migratory insertion of the Pd–H bond into the terminal olefin moiety to afford π-allyl-Pd species **Int-4**. The energy barrier for this step is only 1.1 kcal/mol; however, the reserved β–H elimination step requires activation energy of 29.7 kcal/mol (**Int-4** → **TS-3** → **Int-2**). Therefore, the Pd–H migratory insertion is not a reversible process. The resulting **Int-4** accepts nucleophilic attach from the *si*-face of the metallated azomethine ylide Cu-Nu, to give C–C bond formation intermediate **Int-6**. Dissociation of LCu from **Int-6** affords species **Int-7**, which then undergoes allylic defluorination. The direct nucleophilic displacement/ionization mechanism for the defluorination transition state (**TS-8A**) has a high energy barrier of 28.0 kcal/mol. But a Et_3_NH^+^ mediated process, of which the transition state was located as **TS-8B**, only requires an activation barrier of 12.2 kcal/mol. The resulting **Int-9** then occurs β–H elimination step via **TS-10** with an energy barrier of 27.5 kcal/mol to elaborate product (*2* *S*)-**3aa** and regenerates the PdH catalyst.

**Fig. 9:**
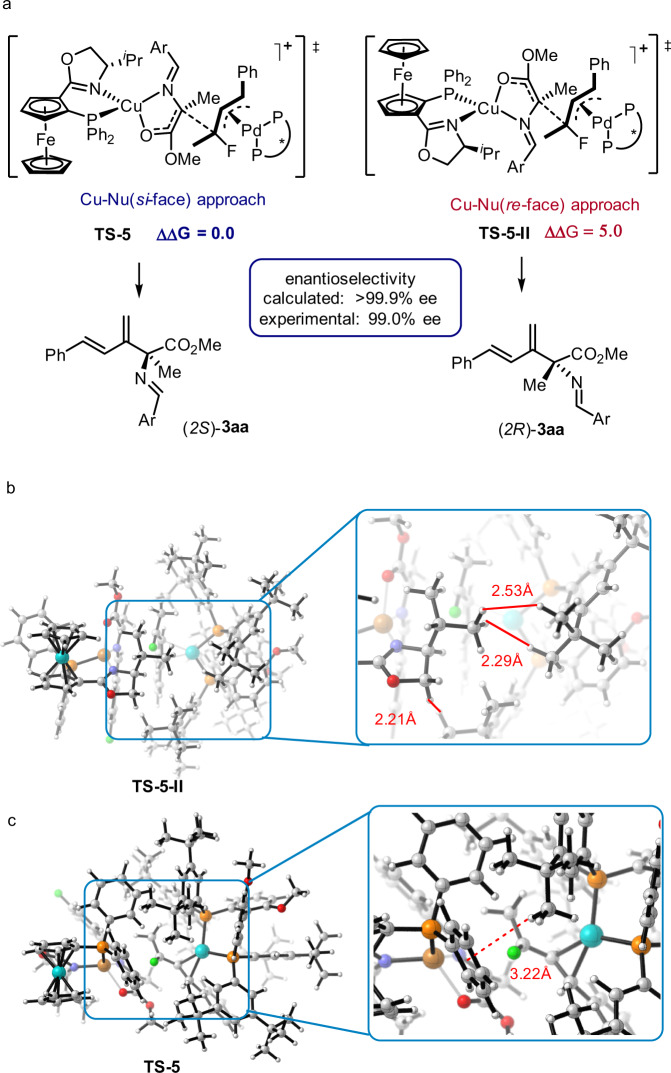
Rationalization of the enantioselectivity. **a** Comparison of Transition states **TS-5** and **TS-5-II**. **b** Steric interaction between the oxazoline ring and *t*-Bu group in **TS-5-II**. **c** Attractive C–H⋯Ar interaction in **TS-5**.

To elucidate the enantioselectivity, the approach from the *re*-face of Cu-Nu during the C–C bond formation was also calculated and the transition state is identified as **TS-5-II** (Fig. 9a). Comparing the energies of **TS-5** and **TS-5-II**, the latter is disfavored over the former by 5.0 kcal/mol, which is in agreement with the experimental result that (*2* *S*)-**3aa** is formed with 99% ee. In the transition state structure **TS-5-II**, the oxazoline ring group on the phosferrox ligand has a very short distance away from *t-*Bu groups of DTB-Biphep and which leads to significant steric interaction between the two ligands (Fig. 9b). This repulsive interaction likely contributes to the higher energy observed for this competing transition state. In contrast, in the transition state structure **TS-5**, the electrophile approaches from the *si*-face of Cu-Nu which avoids the steric repulsion between the oxazoline ring and *t-*Bu groups. Moreover, an attractive C–H ∙ ∙ ∙ Ar interaction is observed in **TS-5**, which also contributes to a lower energy (Fig. 9c).

In summary, the first enantioselective defluorinative Csp^2^–Csp^3^ cross-coupling was achieved by means of synergistic Cu/Pd-catalyzed asymmetric coupling between aldimine esters and dienyl fluorides. This reaction has a wide substrate scope and shows good to excellent enantioselectivities and it provide an efficient catalytic method for preparing chiral α-vinyl, α-alkyl α-amino acid derivatives. Both experimental and computational studies revealed that the reaction is initiated by a PdH migratory insertion, which is followed by nucleophilic allylic substitution by a Cu-azomethine ylide to form the C–C bond. Then a Pd/Et_3_NH^+^-mediated allylic defluorination undergoes, subsequently followed by a β–H elimination to elaborate the coupling product and regenerate the PdH catalyst.

## Methods

### General procedure for coupling of dienyl flurides and aldimine esters

In glove box, Cu(MeCN)_4_PF_6_ (3.7 mg, 0.01 mmol, 5 mol%) and chiral ligand (*S,S*_*p*_)-**L3** (5.3 mg, 0.011 mmol, 5.5 mol%) were dissolved in dry THF (0.4 M, 0.5 mL) and stirred at room temperature for 0.5 h. To the solution, substrate aldimine esters **2** (0.2 mmol), Et_3_N (0.4 mmol), dienes **1** (0.4 mmol) and palladium catalyst L11-Pd (10.1 mg, 0.008 mmol, 4 mol%) were added sequentially. The reaction mixture was stirred at 30 °C for 24 h. To the reaction mixture was added citric acid solution (4 mL, 10 wt%) and the mixture was stirred for 2 h. The mixture was neutralized with solid K_2_CO_3_ and extracted with EtOAc (10 mL × 3). The combined extracts were dried over MgSO_4_ and concentrated in vacuo to afford a residue. The residue was then purified by SiO_2_ column chromatography to give the desired product.

## Supplementary information

Supplementary information

Description of Additional Supplementary Files

Supplementary Data 1

## Data Availability

The authors declare that the data supporting the findings of this study are available within the article and its Supplementary Information Files as well as from the corresponding author on request. The Cartesian coordinates for the calculated structures are available within the Supplementary Data 1. The X-ray crystallographic coordinates for structure reported in this study have been deposited at the Cambridge Crystallographic Data Centre (CCDC), under deposition numbers CCDC 2036502 (*p*-toluenesulfonamide derivative of **3aa**). These data can be obtained free of charge from The Cambridge Crystallographic Data Centre via www.ccdc.cam.ac.uk/data_request/cif.
