# Supplementary material for: Synergistic Pd/Cu-catalyzed enantioselective Csp2–F bond alkylation of fluoro-1,3-dienes with aldimine esters
Source: Nat Commun. 2022 May 5;13:2470. doi: 10.1038/s41467-022-30152-7 (PMC9072389; doi:10.1038/s41467-022-30152-7)
Supplement: Supplementary file 1 — Supplementary information [file 41467_2022_30152_MOESM1_ESM.pdf]

Supplementary Information

Synergistic Pd/Cu-Catalyzed Enantioselective Csp^2–F Bond Alkylation of Fluoro-1,3-Dienes with Aldimine Esters

Huimin Yu[1], Qinglong Zhang[1] and Weiwei Zi[1,2,*]

[1] State Key Laboratory and Institute of Elemento-Organic Chemistry, College of Chemistry, Nankai University, Tianjin, 300071, China

[2] Haihe Laboratory of Sustainable Chemical Transformations, Tianjin 300071, China

[*] Corresponding author: W. Z. (zi@nankai.edu.cn)

Table of Contents

Supplementary Methods....................S2
Supplementary Discussion....................S17
Supplementary Data....................S32
Supplementary References....................S89

1. Supplementary Methods

1.1 General information. Unless otherwise noted, all reactions were assembled on a Schlenk vacuum line or in a glovebox using oven-dried glassware and were stirred with Teflon-coated magnetic stirring bars. All the ligands were purchased from Strem Chemicals or Sinocompound Catalysts Co., Ltd. and were used as received. Unless otherwise noted, reagents were obtained from commercial sources and used without further purification. THF, Toluene were distilled with Na before using. Dichloromethane was distilled with CaH_2 before using. All other dried solvents (DMF, CH_3CN, 1,4-dioxane, Et_3N, DIPEA, DBU) were purchased from MERYER (Shanghai) Chemical Technology Co., Ltd. TLC analysis of reaction mixtures was performed on huanghai silica gel 60 F254 TLC plates and visualized by UV, I_2/silica, and/or ceric ammonium molybdate stain. All work-up and purification procedures were carried out with reagent grade solvents in air. Reaction temperatures above 23 ℃ refer to temperatures of an aluminum heating block or a silicon oil bath, which were controlled by an electronic temperature modulator from IKA. Flash chromatography was carried out with silica gel 200–300 or 300–400 mesh. ^{1}H, ^{13}C and ^{19}F NMR spectra were recorded with Bruker AV-400 spectrometers and were referenced to residual ^{1}H and ^{13}C signals of the deuterated solvents respectively (δ H 7.26, δ C 77.00 for chloroform). Abbreviations are as follows: s (singlet), d (doublet), t (triplet), q (quartet), m (multiplet), br (broad). The ee value was determined on Shimadzu LC-20A HPLC workstation (Daicel chiral columns Chiralpak IA, IC, ID, IE, IF (4.6 x 250 mm)). HRMS was performed on a Varian QFT-ESI instrumental. Optical rotation were determined on a IP-digi 300/2 apparatus. Melting points were determined on an X-4B melting point apparatus.

1.2 The Structures of the Substrates Used in This Work.

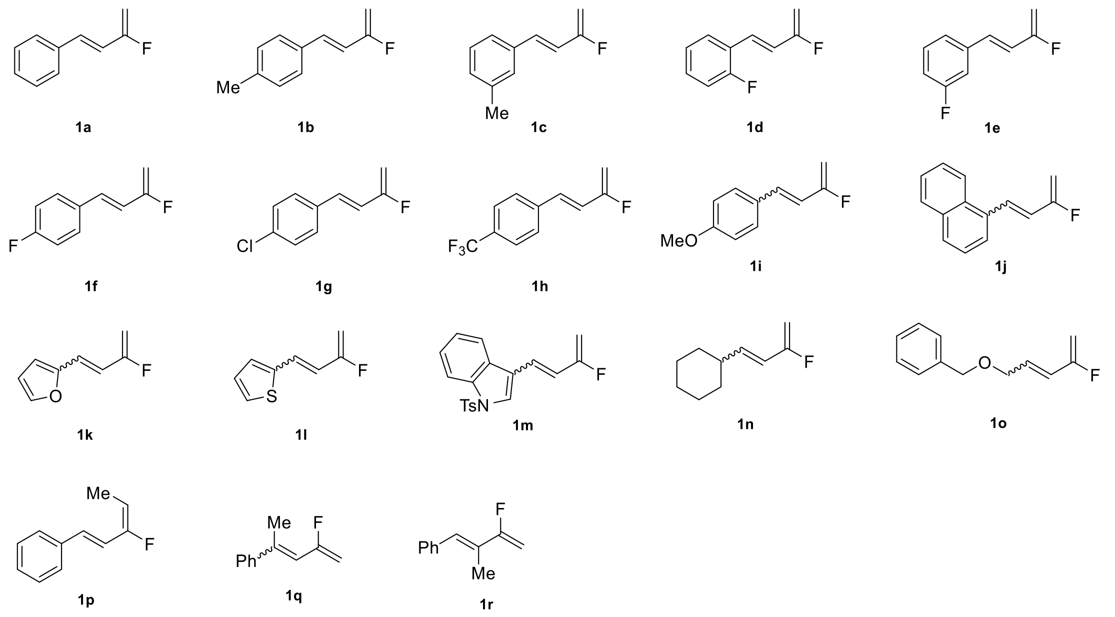

Supplementary Fig. 1 Dienyl fluorides investigated in this work.

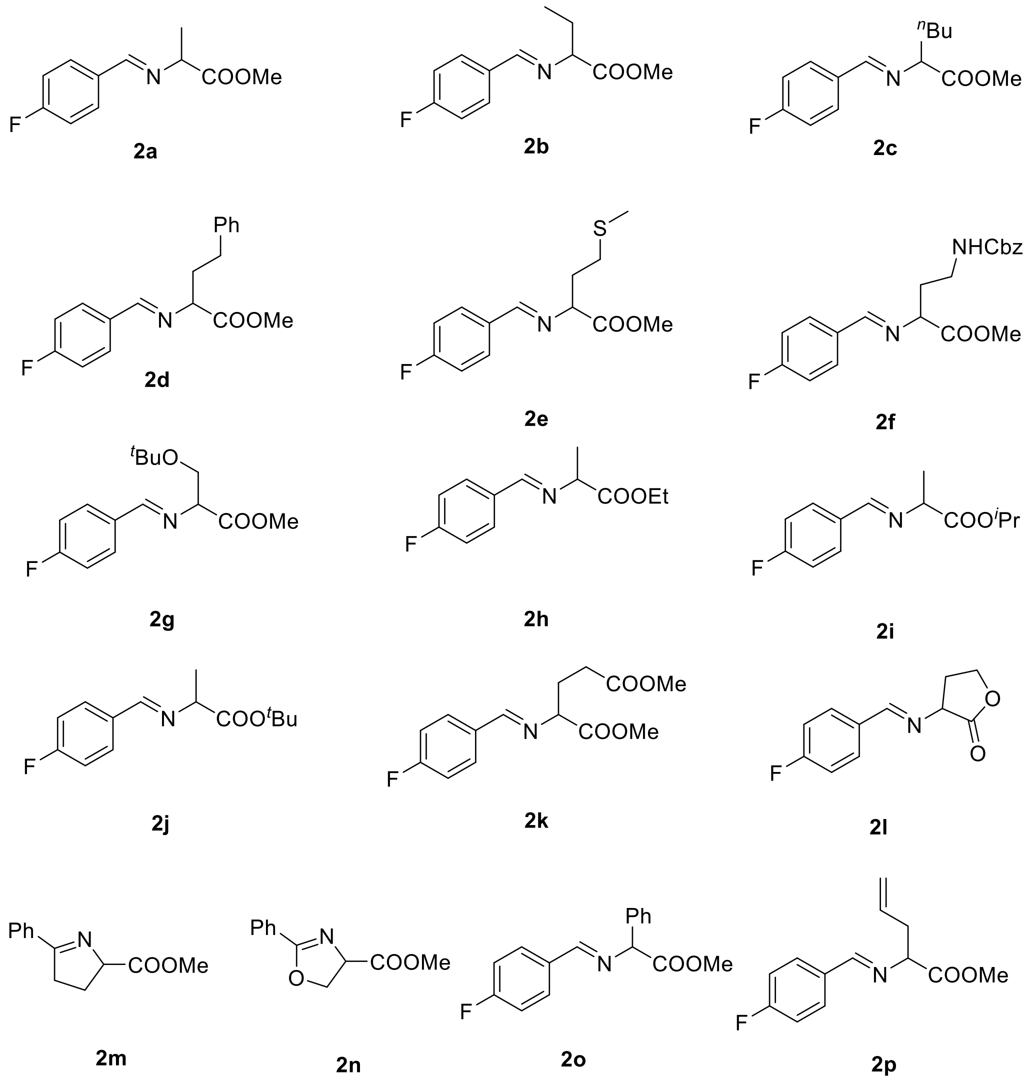

Supplementary Fig. 2 Aldimine esters investigated in this work.

1.3 Preparation and Characterization of Substrates

The dienyl fluride **1a**[1], **4a**, **4b**[2] were known compounds. The aldimine esters used in this work were known compounds and were prepared according to literature procedure[3]. Among the substrates used in this work, **1b**, **1c**, **1d, 1e, 1f, 1g, 1h, 1i, 1j, 1k, 1l, 1m, 1n, 1o, 1p, 1q, 1r** and **2g,** were prepared for the first time and their preparation and characterization were given as below.

1.4 Typical procedure A for the synthesis of (*Z* and *E*)-mixtures of Dienyl Flurides 1i-1o

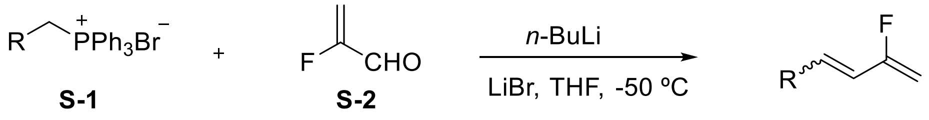

To a 100 ml three-neck flask, **S-1** (16 mmol, 1.6 equiv.) and LiBr (16 mmol, 1.6 equiv.) were added. And then 30 mL THF was added under argon. The slurry was

cooled to -50 ℃ and 6.7 mL (16 mmol, 1.6 equiv.) of 2.4 M *n*-butyllithium solution in hexanes was added slowly by syringe (ca. 10 min). After the addition was complete, the reaction was stirred at -50 ℃ for 1 h, and the addition funnel was charged with the 2-fluoroacrylaldehyde **S-2** (10 mmol, 1.0 equiv.) and 5 ml THF. The aldehyde solution was added dropwise over 15 min and the reaction was stirred at -50 ℃ for 2 h. The reaction was quenched with water (50 mL) and the aqueous phase was extracted with diethyl ether (3 x 20 mL). The combined organic phases were treated with brine (50 mL) and dried with $MgSO_4$. The organics were filtered and concentrated in vacuo. Purification by silica gel flash column chromatography afforded dienyl flurides as colorless oil.

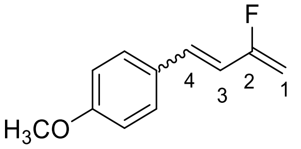

1i

1-(3-fluorobuta-1,3-dien-1-yl)-4-methoxybenzene

Prepared according to typical procedure A from bromo(4-methoxybenzyl)triphenylphosphorane and 2-fluoroacrylaldehyde, Colorless oil, *Z/E* = 2:1, 998 mg, 56% yield.

^{1}H NMR (400 MHz, CDCl$_3$) δ 7.33 – 7.27 (m, 2H), 6.86 – 6.80 (m, 2H), 6.43 (d, *J* = 12.7 Hz, 1H), 5.82 (dd, *J* = 26.7, 12.6 Hz, 1H), 4.72 (dd, *J* = 16.4, 0.8 Hz, 1H), 4.53 (d, *J* = 47.6 Hz, 1H), 3.76 (s, 3H).

^{13}C NMR (101 MHz, CDCl$_3$) δ 162.2 (d, *J* = 254.7 Hz, C2), 159.3, 132.3, 130.6 (d, *J* = 5.1 Hz, C4), 128.3, 118.7 (d, *J* = 25.3 Hz, C3), 113.4, 95.5 (d, *J* = 21.7 Hz, C1), 55.2.

^{19}F NMR (376 MHz, CDCl$_3$) δ -99.1.

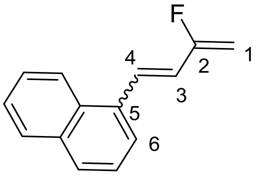

1j

1-(3-fluorobuta-1,3-dien-1-yl)naphthalene

Prepared according to typical procedure A from bromo(naphthalen-1-ylmethyl)triphenylphosphorane and 2-fluoroacrylaldehyde, Colorless oil, *Z/E* = 5:4, 811 mg, 41% yield.

^{1}H NMR (400 MHz, CDCl$_3$) δ 7.82 – 7.73 (m, 4H), 7.52 – 7.40 (m, 3H), 6.68 (d, *J* = 12.6 Hz, 1H), 6.01 (dd, *J* = 26.2, 12.6 Hz, 1H), 4.80 (d, *J* = 16.1 Hz, 1H), 4.59 (d, *J* = 47.7 Hz, 1H).

^{13}C NMR (101 MHz, CDCl$_3$) δ 162.0 (d, *J* = 255.1 Hz, C2), 133.6, 133.3, 132.8, 132.7, 128.4 (d, *J* = 3.9 Hz, C4), 128.2, 127.7, 127.6, 126.9 (d, *J* = 5.4 Hz C5), 126.2 (d, *J* = 8.6 Hz, C6), 123.4, 120.7 (d, *J* = 25.6 Hz, C3), 96.3 (d, *J* = 21.4 Hz, C1).

^{19}F NMR (376 MHz, CDCl$_3$) δ -102.1.

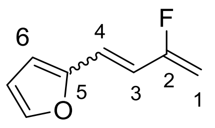

1k

2-(3-fluorobuta-1,3-dien-1-yl)furan

Prepared according to typical procedure A from bromo(furan-2-ylmethyl)triphenylphosphorane and 2-fluoroacrylaldehyde, Colorless oil, *Z/E* = 1:2, 621 mg, 45% yield.

^{1}H NMR (400 MHz, CDCl$_3$) δ 7.43 (d, *J* = 10.3 Hz, 1H), 6.76 (d, *J* = 11.0 Hz, 1H), 6.59 – 6.47 (m, 1H), 6.39 (d, *J* = 18.8 Hz, 1H), 5.77 (dd, *J* = 29.2, 13.3 Hz, 1H), 4.88 (d, *J* = 15.9 Hz, 1H), 4.71 (d, *J* = 48.8 Hz, 1H).

^{13}C NMR (101 MHz, CDCl$_3$) δ 161.9 (d, *J* = 253.7 Hz, C2), 151.9 (d, *J* = 118.2 Hz, C5), 142.1 (d, *J* = 9.4 Hz, C6), 133.8 (d, *J* = 19.4 Hz, C3), 116.4 (d, *J* = 28.5 Hz, C4), 114.0, 108.8, 96.1 (d, *J* = 22.6 Hz, C1).

^{19}F NMR (376 MHz, CDCl$_3$) δ -100.92.

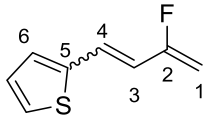

1l

2-(3-fluorobuta-1,3-dien-1-yl)thiophene

Prepared according to typical procedure A from bromotriphenyl(thiophen-2-ylmethyl)phosphorane and 2-fluoroacrylaldehyde, Colorless oil, *Z/E* = 2:3, 723 mg, 47% yield.

^{1}H NMR (400 MHz, CDCl$_3$) δ 7.28 (d, *J* = 5.1 Hz, 1H), 7.22 – 7.18 (m, 1H), 7.14 – 7.09 (m, 1H), 6.50 (d, *J* = 12.9 Hz, 1H), 5.74 (dd, *J* = 28.3, 12.9 Hz, 1H), 4.81 (d, *J* = 15.9 Hz, 1H), 4.62 (d, *J* = 46.9 Hz, 1H).

^{13}C NMR (101 MHz, CDCl$_3$) δ 161.8 (d, *J* = 251.4 Hz, C2), 138.9 (d, *J* = 40.4 Hz, C5), 133.8 (d, *J* = 19.5 Hz, C3), 127.9, 126.9 (d, *J* = 13.3 Hz, C6), 122.9, 117.1 (d, *J* = 27.1 Hz, C4), 96.2 (d, *J* = 22.1 Hz, C1).

^{19}F NMR (376 MHz, CDCl$_3$) δ -99.1.

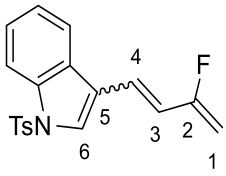

1m

3-(3-fluorobuta-1,3-dien-1-yl)-1-tosyl-1H-indole

Prepared according to typical procedure A from 3-((bromotriphenylphosphoranyl)methyl)-1-tosyl-1H-indole and 2-fluoroacrylaldehyde, Colorless oil, *Z/E* = 3:2, 1.7 g, 51% yield.

^{1}H NMR (400 MHz, CDCl$_3$) δ 8.02 (d, *J* = 10.7 Hz, 1H), 7.81 – 7.73 (m, 3H), 7.45 (t, *J* = 7.0 Hz, 1H), 7.34 – 7.20 (m, 2H), 7.15 – 7.09(d, *J* = 7.0 Hz, 2H), 6.42 (dd, *J* = 12.5, 6.3 Hz, 1H), 5.98 (ddd, *J* = 29.8, 12.6, 6.7 Hz, 1H), 4.82 (d, *J* = 16.3 Hz, 1H), 4.59 (d, *J* = 47.4 Hz, 1H), 2.22 (s, 3H).

^{13}C NMR (101 MHz, CDCl$_3$) δ 162.3 (d, *J* = 255.1 Hz, C2), 145.1, 134.8 (d, *J* = 64.4 Hz, C3), 129.9, 126.9, 125.9 (d, *J* = 18.9 Hz, C5), 125.2 (d, *J* = 12.5 Hz, C6), 124.8, 123.5, 121.3 (d, *J* = 4.4

Hz, C4), 120.6, 120.3, 119.3, 117.5, 113.6, 96.9 (d, *J* = 21.7 Hz, C1), 21.5.
^{19}F NMR (376 MHz, $CDCl_3$) δ -102.1.

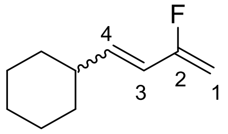

1n

(3-fluorobuta-1,3-dien-1-yl)cyclohexane

Prepared according to typical procedure A from bromo(cyclohexylmethyl)triphenylphosphorane and 2-fluoroacrylaldehyde, Colorless oil, *Z/E* = 8:1, 862 mg, 56% yield.

^{1}H NMR (400 MHz, $CDCl_3$) δ 5.60 (dd, *J* = 28.5, 12.0 Hz, 1H), 5.36 (dd, *J* = 31.8, 20.6 Hz, 1H), 4.64 (d, *J* = 16.9 Hz, 1H), 4.38 (d, *J* = 48.4 Hz, 1H), 2.31 – 2.16 (m, *J* = 5.2, 1H), 1.77 – 1.53 (m, 5H), 1.38 – 0.96 (m, 5H).

^{13}C NMR (101 MHz, $CDCl_3$) δ 163.1 (d, *J* = 254.7 Hz, C2), 141.5 (d, *J* = 2.0 Hz, C4), 117.9 (d, *J* = 24.9 Hz, C3), 93.9 (d, *J* = 22.1 Hz, C1), 36.6, 33.8, 26.1, 26.0.

^{19}F NMR (377 MHz, $CDCl_3$) δ -102.4.

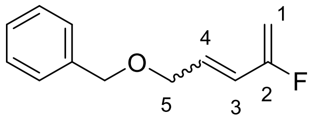

1o

(((4-fluoropenta-2,4-dien-1-yl)oxy)methyl)benzene

Prepared according to typical procedure A from (2-(benzyloxy)ethyl)bromotriphenylphosphorane and 2-fluoroacrylaldehyde, Colorless oil, *Z/E* = 2:1, 1.2 g, 65% yield.

^{1}H NMR (400 MHz, $CDCl_3$) δ 7.36 – 7.26 (m, 5H), 6.17 – 6.10 (m, 1H), 5.75 (dd, *J* = 11.8, 5.4 Hz, 1H), 4.67 (dd, *J* = 16.1, 2.6 Hz, 1H), 4.52 (s, 2H), 4.51 – 4.48 (m, 1H), 4.38 (d, *J* = 4.6 Hz, 2H).

^{13}C NMR (101 MHz, $CDCl_3$) δ 162.4 (d, *J* = 253.8 Hz, C2), 138.1, 132.4 (d, *J* = 3.3 Hz, C4), 128.5, 127.8, 127.7, 120.8 (d, *J* = 26.5 Hz, C3), 95.6 (d, *J* = 21.5 Hz, C1), 72.6, 67.6 (d, *J* = 9.9 Hz, C5).

^{19}F NMR (377 MHz, $CDCl_3$) δ -102.6.

1.5 Typical procedure B for the synthesis of (*E*)-Dienyl Flurides 1a-1h

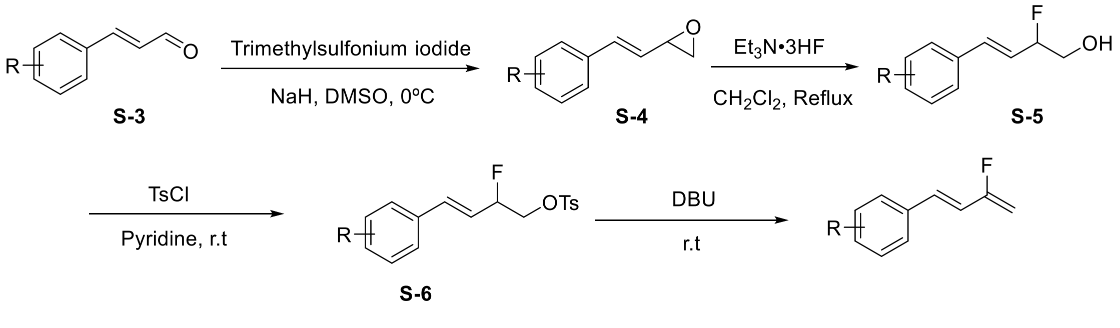

A 500 mL oven-dried round-bottom flask was charged with NaH (60% dispersion in oil, 1.2 equiv.). Dry DMSO (1.0 M) were added to the flask and the resulting slurry solution was cooled to 0 ℃. Trimethylsulfonium iodide (1.15 equiv.) and DMSO (15 mL) were added to a separate oven-dried round-bottom flask. The solution was transferred slowly to the NaH solution via cannula. After the transfer was complete,

acrylaldehyde **S-3** (1.0 equiv.) was then added to the reaction mixture in one portion. The resulting mixture was stirred at 0 ℃ for 1 h, then at room temperature for another 1 h and quenched with distilled water. The resulting mixture was then diluted with diethyl ether (40 mL) and brine (30 mL). The two layers were separated, and the aqueous layer was back extracted with diethyl ether (2 x 100 mL). The combined organic layer was dried with $MgSO_4$, filtered, and concentrated in vacuo to afford **S-4** without further purification.

A 250 mL oven-dried round-bottom flask was charged with **S-4** (1.0 equiv.). Dry CH_2Cl_2 (0.5 M) were added to the flask and the resulting solution was cooled to 0 ℃. $Et_3N{\cdot}3HF$ (2.0 equiv.) were added dropwise. The resulting mixture was stirred at 50 ℃ for 8 h, and then cooled to the room temperature. The reaction mixture was quenched by the addition of $NaHCO_3$ (aq.) and diluted with CH_2Cl_2. The two layers were separated, and the aqueous layer was back extracted with CH_2Cl_2 (2 x 30 mL). The combined organic layer was dried with $MgSO_4$, filtered, and concentrated in vacuo to afford **S-5** without further purification.

To a solution of **S-5** (1.0 equiv.) and pyridine (1.0 M), TsCl (1.0 equiv.) was added stepwise at room temperature and stirred 24 h. The reaction mixture was quenched by the addition of 4N HCl and diluted with CH_2Cl_2. The two layers were separated, and the aqueous layer was back extracted with CH_2Cl_2 (2 x 30 mL). The combined organic layer was dried with $MgSO_4$, filtered, and concentrated in vacuo. Purification by silica gel flash column chromatography afforded **S-6** as a yellow oil.

In a 25 ml oven-dried Schlenk flask, **S-6** (5 mmol, 1.0 equiv.) and DBU (5mmol, 1.0 equiv.) were added. The resulting mixture was stirred at room temperature for 9 h. Water was added and diluted with diethyl ether (3 x 10 mL). The combined organic layer was dried with $MgSO_4$, filtered, and concentrated in vacuo. Purification by silica gel flash column chromatography afforded dienyl fluride as colorless oil.

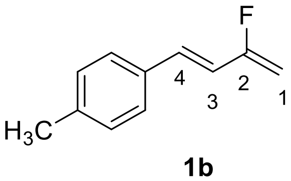

1b

(*E*)-1-(3-fluorobuta-1,3-dien-1-yl)-4-methylbenzene

Prepared according to typical procedure B from 3-(p-tolyl)acrylaldehyde, Colorless oil, 566 mg, 70% yield.

^{1}H NMR (400 MHz, $CDCl_3$) δ 7.32 (d, *J* = 7.0 Hz, 2H), 7.15 (d, *J* = 7.4 Hz, 2H), 6.87 (d, *J* = 16.0 Hz, 1H), 6.48 (ddd, *J* = 24.9, 16.0, 1.2 Hz, 1H), 4.74 (d, *J* = 16.1 Hz, 1H), 4.53 (d, *J* = 48.5 Hz, 1H), 2.35 (s, 3H).

^{13}C NMR (101 MHz, $CDCl_3$) δ 162.4 (d, *J* = 251.3 Hz, C2), 138.5, 133.1, 130.5 (d, *J* = 4.0 Hz, C4), 129.5, 126.9, 118.6 (d, *J* = 24.2 Hz, C3), 93.4 (d, *J* = 21.7 Hz, C1), 21.3.

^{19}F NMR (376 MHz, $CDCl_3$) δ -111.9.

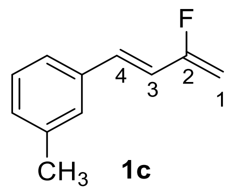

1c

(*E*)-1-(3-fluorobuta-1,3-dien-1-yl)-3-methylbenzene

Prepared according to typical procedure B from 3-(m-tolyl)acrylaldehyde, Colorless oil, 501 mg, 62% yield.

^{1}H NMR (400 MHz, CDCl$_3$) δ 7.22 (d, *J* = 4.9 Hz, 3H), 7.09 (s, 1H), 6.86 (d, *J* = 16.0 Hz, 1H), 6.50 (dd, *J* = 24.8, 16.0 Hz, 1H), 4.75 (d, *J* = 16.1 Hz, 1H), 4.54 (dd, *J* = 48.5, 1.8 Hz, 1H), 2.34 (s, 3H).

^{13}C NMR (101 MHz, CDCl$_3$) δ 162.4 (d, *J* = 251.6 Hz, C2), 138.3, 135.8, 130.7 (d, *J* = 3.8 Hz, C4), 130.7, 129.3, 128.7, 124.1, 119.4 (d, *J* = 24.1 Hz, C3), 93.8 (d, *J* = 21.7 Hz, C1), 21.4.

^{19}F NMR (376 MHz, CDCl$_3$) δ -111.9.

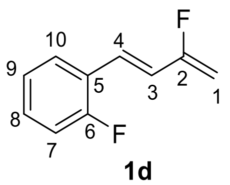

1d

(*E*)-1-fluoro-2-(3-fluorobuta-1,3-dien-1-yl)benzene

Prepared according to typical procedure B from 3-(2-fluorophenyl)acrylaldehyde, Colorless oil, 648 mg, 78% yield.

^{1}H NMR (400 MHz, CDCl$_3$) δ 7.43 (t, *J* = 7.4 Hz, 1H), 7.23 (dd, *J* = 13.2, 6.1 Hz, 1H), 7.12 – 6.99 (m, 3H), 6.62 (dd, *J* = 24.6, 16.2 Hz, 1H), 4.80 (dd, *J* = 15.9, 2.7 Hz, 1H), 4.59 (dd, *J* = 48.1, 2.7 Hz, 1H).

^{13}C NMR (101 MHz, CDCl$_3$) δ 162.1 (d, *J* = 252.0 Hz, C2), 160.8 (d, *J* = 251.2 Hz, C6), 129.7 (d, *J* = 8.5 Hz, C8), 128.0 (d, *J* = 3.4 Hz, C10), 124.3 (d, *J* = 3.6 Hz, C9), 123.8 (d, *J* = 11.8 Hz, C4), 123.4 (dd, *J* = 4.5, 3.0 Hz, C5), 122.1 (dd, *J* = 23.8, 6.7 Hz, C3), 115.9 (d, *J* = 22.1 Hz, C7), 94.8 (d, *J* = 21.4 Hz, C1).

^{19}F NMR (376 MHz, CDCl$_3$) δ -112.4, -116.4 (2-Ar).

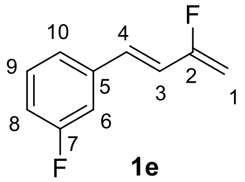

1e

(*E*)-1-fluoro-3-(3-fluorobuta-1,3-dien-1-yl)benzene

Prepared according to typical procedure B from 3-(3-fluorophenyl)acrylaldehyde, Colorless oil, 621 mg, 75% yield.

^{1}H NMR (400 MHz, CDCl$_3$) δ 7.36 – 7.25 (m, 1H), 7.16 (dd, *J* = 25.0, 8.6 Hz, 2H), 6.98 (t, *J* = 8.2 Hz, 1H), 6.86 (d, *J* = 16.0 Hz, 1H), 6.52 (ddd, *J* = 24.5, 16.1, 2.1 Hz, 1H), 4.82 (d, *J* = 16.0 Hz, 1H), 4.61 (d, *J* = 48.2 Hz, 1H).

^{13}C NMR (101 MHz, CDCl$_3$) δ163.1 (d, *J* = 245.2 Hz, C2), 162.3 (d, *J* = 251.2 Hz, C7), 138.1 (d, *J* = 7.2 Hz, C5), 130.2 (d, *J* = 8.4 Hz, C4), 129.4 (dd, *J* = 6.4, 3.2 Hz, C9), 122.9 (d, *J* = 2.7 Hz), 120.9 (d, *J* = 27.0 Hz, C3), 115.3 (d, *J* = 21.4 Hz, C8), 113.2 (d, *J* = 21.9 Hz, C6), 94.9 (d, *J* = 21.3

Hz, C1).

^{19}F NMR (376 MHz, $CDCl_3$) δ -116.9, -117.9.

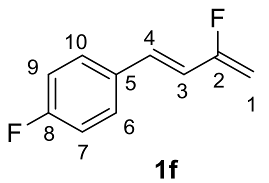

1f

(*E*)-1-fluoro-4-(3-fluorobuta-1,3-dien-1-yl)benzene

Prepared according to typical procedure B from 3-(4-fluorophenyl)acrylaldehyde, Colorless oil, 563 mg, 68% yield.

^{1}H NMR (400 MHz, $CDCl_3$) δ 7.39 (dd, *J* = 7.9, 5.7 Hz, 2H), 7.03 (t, *J* = 8.5 Hz, 2H), 6.85 (d, *J* = 16.0 Hz, 1H), 6.44 (dd, *J* = 24.6, 16.0 Hz, 1H), 4.77 (dd, *J* = 16.1, 2.4 Hz, 1H), 4.56 (dd, *J* = 48.4, 2.4 Hz, 1H).

^{13}C NMR (101 MHz, $CDCl_3$) δ 162.8 (d, *J* = 248.6 Hz, C2), 162.0 (d, *J* = 251.5 Hz, C8), 132.0 (d, *J* = 3.4 Hz, C4), 129.4 (d, *J* = 4.3 Hz, C5), 128.5 (d, *J* = 8.1 Hz, C6 and C10), 119.4 (dd, *J* = 24.3, 2.4 Hz, C3), 115.8 (d, *J* = 21.8 Hz, C7 and C9), 94.0 (d, *J* = 20.9 Hz, C1).

^{19}F NMR (376 MHz, $CDCl_3$) δ -116.8, -117.5.

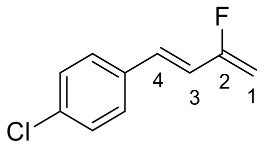

1g

(*E*)-1-chloro-4-(3-fluorobuta-1,3-dien-1-yl)benzene

Prepared according to typical procedure B from 3-(3-chlorophenyl)acrylaldehyde, Colorless oil, 746 mg, 82% yield.

^{1}H NMR (400 MHz, $CDCl_3$) δ 7.39 – 7.27 (m, 4H), 6.83 (d, *J* = 16.0 Hz, 1H), 6.48 (dd, *J* = 24.6, 16.0 Hz, 1H), 4.79 (dd, *J* = 16.0, 2.9 Hz, 1H), 4.57 (dd, *J* = 48.2, 2.8 Hz, 1H).

^{13}C NMR (101 MHz, $CDCl_3$) δ 161.9 (d, *J* = 251.6 Hz, C2), 134.3, 134.1, 129.3 (d, *J* = 4.1 Hz, C4), 128.9, 128.1, 120.2 (d, *J* = 24.4 Hz, C3), 94.5 (d, *J* = 21.3 Hz, C1).

^{19}F NMR (376 MHz, $CDCl_3$) δ -112.0.

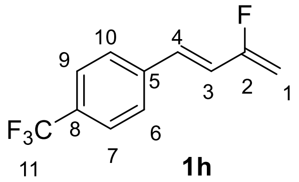

1h

(*E*)-1-(3-fluorobuta-1,3-dien-1-yl)-4-(trifluoromethyl)benzene

Prepared according to typical procedure B from 3-(3-(trifluoromethyl)phenyl)acrylaldehyde, Colorless oil, 831 mg, 77% yield.

^{1}H NMR (400 MHz, $CDCl_3$) δ 7.59 (d, *J* = 8.3 Hz, 2H), 7.50 (d, *J* = 8.2 Hz, 2H), 6.90 (d, *J* = 16.0 Hz, 1H), 6.59 (dd, *J* = 24.4, 16.0 Hz, 1H), 4.85 (dd, *J* = 15.8, 2.9 Hz, 1H), 4.64 (dd, *J* = 48.0, 2.9 Hz, 1H).

^{13}C NMR (101 MHz, $CDCl_3$) δ 161.7 (d, *J* = 251.9 Hz, C2), 139.3, 130.1 (q, *J* = 32.5 Hz, C8), 129.0 (d, *J* = 3.8 Hz, C4), 127.0, 125.7 (q, *J* = 3.8 Hz, C7 and C9), 124.1 (q, *J* = 272.0 Hz, C11), 122.0 (d, *J* = 24.4 Hz, C3), 95.6 (d, *J* = 21.3 Hz, C1).

^{19}F NMR (376 MHz, $CDCl_3$) δ -62.6, -112.1.

1.6 Typical procedure C for the synthesis of Dienyl Fluride 1p

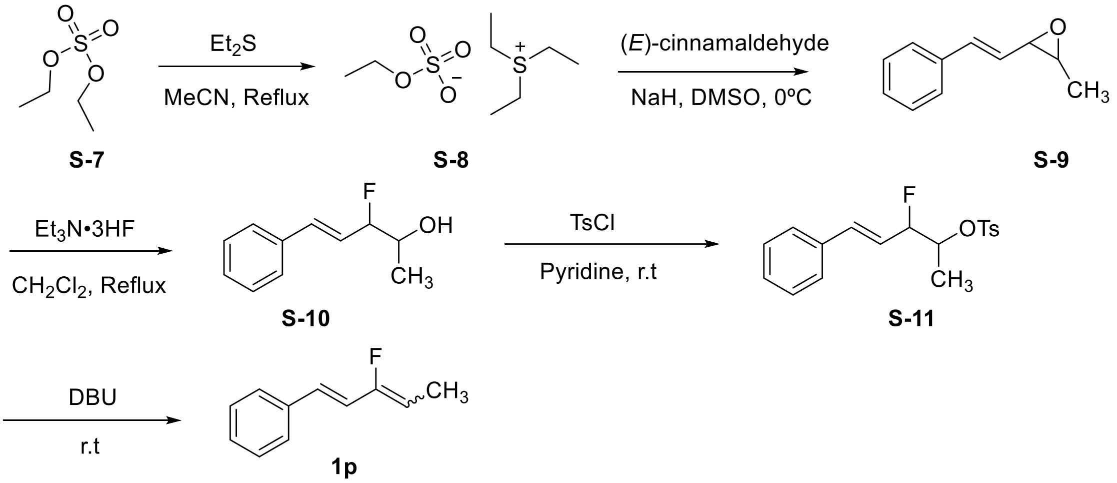

A mixture of diethylsulfide **S-7** **(**100 mmol, 1.0 equiv.) and diethylsulfate (100 mmol, 1.0 equiv.) in acetonitrile (20 ml) is heated to reflux during 2.5 hours. Acetonitrile is evaporated under vacuum and the remaining oil is washed several times with dry ether. Residual solvents are removed under vacuum. The salt **S-8** was used directly for the epoxidations.

A 500 mL oven-dried round-bottom flask was charged with NaH (60% dispersion in oil, 1.2 equiv.). Dry DMSO (1 M) were added to the flask and the resulting slurry solution was cooled to 0 ℃. **S-8** (1.15 equiv.) and DMSO (15 mL) were added to a separate oven-dried round-bottom flask. The solution was transferred slowly to the NaH solution via cannula. After the transfer was complete, (*E*)-cinnamaldehyde (1.0 equiv.) was then added to the reaction mixture in one portion. The resulting mixture was stirred at 0 ℃ for 1 h, then at room temperature for another 1 h and quenched with distilled water. The resulting mixture was then diluted with diethyl ether (40 mL) and brine (30 mL). The two layers were separated, and the aqueous layer was back extracted with diethyl ether (2 x 100 mL). The combined organic layer was dried with $MgSO_4$, filtered, and concentrated in vacuo to afford **S-9** without further purification.

A 250 mL oven-dried round-bottom flask was charged with **S-9** (1.0 equiv.). Dry CH_2Cl_2 (0.5 M) were added to the flask and the resulting solution was cooled to 0 ℃. $Et_3N{\cdot}3HF$ (2.0 equiv.) were added dropwise. The resulting mixture was stirred at 50 ℃ for 8 h, and then cooled to the room temperature. The reaction mixture was quenched by the addition of $NaHCO_3$ (aq.) and diluted with CH_2Cl_2. The two layers were separated, and the aqueous layer was back extracted with CH_2Cl_2 (2 x 30 mL). The combined organic layer was dried with $MgSO_4$, filtered, and concentrated in vacuo to afford **S-10** without further purification.

To a solution of **S-10** (1.0 equiv.) and pyridine (1M), TsCl (1.0 equiv.) was added stepwise at room temperature and stirred 24 h. The reaction mixture was quenched by the addition of 4N HCl and diluted with CH_2Cl_2. The two layers were separated, and the aqueous layer was back extracted with CH_2Cl_2 (2 x 30 mL). The combined organic

layer was dried with $MgSO_4$, filtered, and concentrated in vacuo. Purification by silica gel flash column chromatography afforded **S-11** as a yellow oil.

In a 25 ml oven-dried Schlenk flask, **S-11** (5 mmol, 1.0 equiv.) and DBU (5 mmol, 1.0 equiv.) were added. The resulting mixture was stirred at room temperature for 9 h. Water was added and diluted diethyl ether (3 x 10 mL). The combined organic layer was dried with $MgSO_4$, filtered, and concentrated in vacuo. Purification by silica gel flash column chromatography afforded **1p** as colorless oil.

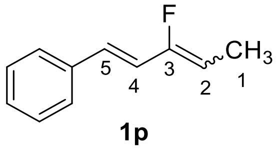

1p

((1*E*)-3-fluoropenta-1,3-dien-1-yl)benzene

Prepared according to typical procedure C, Colorless oil, 550 mg, 68% yield.

^{1}H NMR (400 MHz, $CDCl_3$) δ 7.39 – 7.34 (m, 2H), 7.30 (m, 7.32-7.28, 3H), 6.93 – 6.68 (m, 2H), 4.89 (dq, *J* = 36.0, 7.2 Hz, 1H), 1.72 (t, *J* = 2.5 Hz, 3H).

^{13}C NMR (101 MHz, $CDCl_3$) δ 157.5 (d, *J* = 247.6 Hz, C3), 136.5, 128.7, 127.9, 127.5 (d, *J* = 3.6 Hz, C5), 126.7, 120.4 (d, *J* = 24.0 Hz, C4), 106.2 (d, *J* = 17.5 Hz, C2), 9.8 (d, *J* = 5.8 Hz, C1).

^{19}F NMR (377 MHz, $CDCl_3$) δ -125.2.

1.7 Typical procedure C for the synthesis of Dienyl Fluride 1q

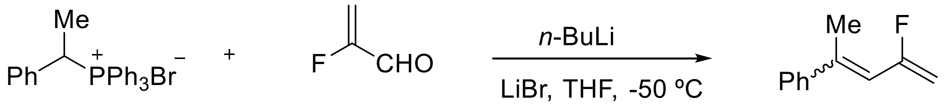

To a 100 ml three-neck flask, **S-1** (16 mmol, 1.6 equiv.) and LiBr (16 mmol, 1.6 equiv.) were added. And then 30 mL THF was added under argon. The slurry was cooled to -50 ℃ and 6.7 mL (16 mmol, 1.6 equiv.) of 2.4 M *n*-butyllithium solution in hexanes was added slowly by syringe (ca. 10 min). After the addition was complete, the reaction was stirred at -50 ℃ for 1 h, and the addition funnel was charged with the 2-fluoroacrylaldehyde **S-2** (10 mmol, 1.0 equiv.) and 5 ml THF. The aldehyde solution was added dropwise over 15 min and the reaction was stirred at -50 ℃ for 2h. The reaction was quenched with water (50 mL) and the aqueous phase was extracted with diethyl ether (3 x 20 mL). The combined organic phases were treated with brine (50 mL) and dried with $MgSO_4$. The organics were filtered and concentrated in vacuo. Purification by silica gel flash column chromatography afforded dienyl fluride **1q** as colorless oil (*Z/E* = 1:5, 973 mg, 61% yield).

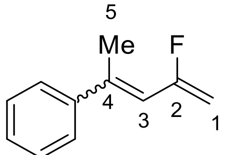

1q

(4-fluoropenta-2,4-dien-2-yl)benzene

^{1}H NMR (400 MHz, $CDCl_3$) δ 7.44 – 7.40 (m, 2H), 7.37 – 7.26 (m, 3H), 6.09 (d, *J* = 24.8 Hz, 1H), 4.79 (dd, *J* = 17.1, 2.6 Hz, 1H), 4.52 (dd, *J* = 48.6, 2.6 Hz, 1H), 2.32 (s, 3H).

^{13}C NMR (101 MHz, CDCl$_3$) δ 163.1 (d, *J* = 252.5 Hz, C2), 140.9, 133.8 (d, *J* = 19.4 Hz, C4), 128.4, 127.8, 126.1, 118.6 (d, *J* = 25.3 Hz, C3), 95.1 (d, *J* = 23.2 Hz, C1), 17.9 (d, *J* = 8.1 Hz, C5).
^{19}F NMR (376 MHz, CDCl$_3$) δ -100.56.

1.8 Typical procedure for the synthesis of (*E*)-Dienyl Flurides 1r

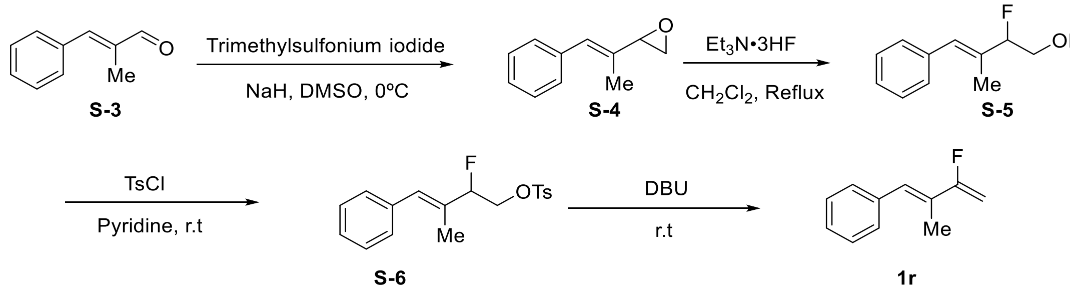

A 500 mL oven-dried round-bottom flask was charged with NaH (60% dispersion in oil, 1.2 equiv.). Dry DMSO (1.0 M) were added to the flask and the resulting slurry solution was cooled to 0 ℃. Trimethylsulfonium iodide (1.15 equiv.) and DMSO (15 mL) were added to a separate oven-dried round-bottom flask. The solution was transferred slowly to the NaH solution via cannula. After the transfer was complete, acrylaldehyde **S-3** (1.0 equiv.) was then added to the reaction mixture in one portion. The resulting mixture was stirred at 0 ℃ for 1 h, then at room temperature for another 1 h and quenched with distilled water. The resulting mixture was then diluted with diethyl ether (40 mL) and brine (30 mL). The two layers were separated, and the aqueous layer was back extracted with diethyl ether (2 x 100 mL). The combined organic layer was dried with $MgSO_4$, filtered, and concentrated in vacuo to afford **S-4** without further purification.

A 250 mL oven-dried round-bottom flask was charged with **S-4** (1.0 equiv.). Dry CH_2Cl_2 (0.5 M) were added to the flask and the resulting solution was cooled to 0 ℃. $Et_3N{\cdot}3HF$ (2.0 equiv.) were added dropwise. The resulting mixture was stirred at 50 ℃ for 8 h, and then cooled to the room temperature. The reaction mixture was quenched by the addition of $NaHCO_3$ (aq.) and diluted with CH_2Cl_2. The two layers were separated, and the aqueous layer was back extracted with CH_2Cl_2 (2 x 30 mL). The combined organic layer was dried with $MgSO_4$, filtered, and concentrated in vacuo to afford **S-5** without further purification.

To a solution of **S-5** (1.0 equiv.) and pyridine (1.0 M), TsCl (1.0 equiv.) was added stepwise at room temperature and stirred 24 h. The reaction mixture was quenched by the addition of 4N HCl and diluted with CH_2Cl_2. The two layers were separated, and the aqueous layer was back extracted with CH_2Cl_2 (2 x 30 mL). The combined organic layer was dried with $MgSO_4$, filtered, and concentrated in vacuo. Purification by silica gel flash column chromatography afforded **S-6** as a yellow oil.

In a 25 ml oven-dried Schlenk flask, **S-6** (5 mmol, 1.0 equiv.) and DBU (5mmol, 1.0 equiv.) were added. The resulting mixture was stirred at room temperature for 9 h. Water was added and diluted with diethyl ether (3 x 10 mL). The combined organic layer was dried with $MgSO_4$, filtered, and concentrated in vacuo. Purification by silica gel flash column chromatography afforded dienyl fluride **1r** as colorless oil (899mg,

55% yield).

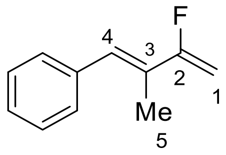

1r

(*E*)-(3-fluoro-2-methylbuta-1,3-dien-1-yl)benzene

^{1}H NMR (400 MHz, $CDCl_3$) δ 7.36 – 7.34 (m, 2H), 7.33 – 7.21 (m, 3H), 7.00 (s, 1H), 4.80 (d, *J* = 18.5 Hz, 1H), 4.67 (d, *J* = 50.2 Hz, 1H), 1.99 (s, 3H).

^{13}C NMR (101 MHz, $CDCl_3$) δ 164.6 (d, *J* = 251.4 Hz, C2), 136.8, 129.4, 128.2, 127.8 (d, *J* = 19.6 Hz, C3), 127.7 (d, *J* = 8.8 Hz, C4), 127.2, 91.1 (d, *J* = 23.9 Hz, C1), 14.1 (d, *J* = 3.2 Hz, C5).

^{19}F NMR (376 MHz, $CDCl_3$) δ -109.29.

Aldimine ester **2g** is a new compound, and its NMR data is as follows.

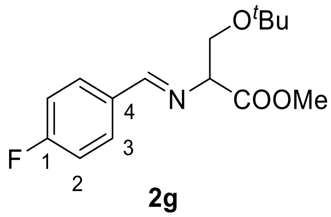

2g

(*E*)-methyl 3-(tert-butoxy)-2-((4-fluorobenzylidene)amino)propanoate

^{1}H NMR (400 MHz, $CDCl_3$) δ 8.42 (s, 1H), 7.98 – 7.90 (m, 2H), 7.24 (t, *J* = 8.4 Hz, 2H), 4.29 (t, *J* = 6.4 Hz, 1H), 4.07 (dd, *J* = 8.7, 5.8 Hz, 1H), 3.91 (s, 3H), 3.77 (t, *J* = 8.2 Hz, 1H), 1.30 (s, 9H).

^{13}C NMR (101 MHz, $CDCl_3$) δ 171.2, 164.6 (d, *J* = 251.2 Hz, C1), 163.1, 132.1 (d, *J* = 2.4 Hz, C4), 130.5 (d, *J* = 8.7 Hz, C3), 115.7 (d, *J* = 21.9 Hz, C2), 73.6, 73.3, 62.9, 52.1, 27.5.

^{19}F NMR (376 MHz, $CDCl_3$) δ -108.8.

1.9 General Procedure for Coupling of Dienyl Flurides with Aldimine Esters

Pd catalyst preparation: In an Ar-filled glove box, to a dried Schlenk tube were added (*S*)-DT-Biphep(0.6 mmol) and [Pd(η^3-allyl)Cl]$_2$ (0.3 mmol), dry DCM (5 mL) was then added and the mixture was stirred at room temperature for 1 h, then $AgBF_4$ (0.6 mmol) was added and the mixture was stirred for another 1 h. The mixture was removed from the glove box. The solution was filtered through a pad of celite eluting with CH_2Cl_2 (5 mL x 3) and the filtrate was concentrated in vacuo to afford catalyst **L11-Pd** as a yellow solid, which was stored at -20 ℃ under inert atmosphere. The palladium catalyst was used without further purification. The *ent*-**L11-Pd** catalyst was prepared using the same procedure but employing (*R*)-DT-Biphep as the ligand.

Coupling reaction procedure A: In glove box, $Cu(MeCN)_4PF_6$ (3.7 mg, 0.01 mmol, 5 mol%) and chiral ligand (S,S_p)-**L3** (5.3 mg, 0.011 mmol, 5.5 mol%) were dissolved in dry THF (0.4 M, 0.5 mL) and stirred at room temperature for 0.5 h. To the solution, substrate aldimine esters **2** (0.2 mmol), Et_3N (0.4 mmol), dienes (0.4 mmol) and palladium catalyst **L11-Pd** or *ent*-**L11-Pd** (10.1 mg, 0.008 mmol, 4 mol%) were added sequentially. The reaction mixture was stirred at 30 ℃ for 24 h. To the reaction mixture was added citric acid solution (4 mL, 10 wt.%) and the mixture was stirred for 2 h. The mixture was neutralized with solid K_2CO_3 and extracted with EtOAc (10 mL x 3). The combined extracts were dried over $MgSO_4$ and concentrated in vacuo to afford a residue. The residue was then purified by SiO_2 column chromatography (PE/EA = 5:1 to 1:1) to give the desired product.
Reactions in Table 1 and Table 2 were performed using the above procedure A.

Coupling reaction procedure B: In glove box, $Cu(MeCN)_4PF_6$ (3.7 mg, 0.01 mmol, 5 mol%) and chiral ligand (S,S_p)-**L3** (5.3 mg, 0.011 mmol, 5.5 mol%) were dissolved in dry THF (0.4M, 0.5 mL) and stirred at room temperature for 0.5 h. To the solution, substrate aldimine esters **2** (0.2 mmol), Et_3N (0.4 mmol), dienes (0.4 mmol) and **L11-Pd** (10.1 mg, 0.008 mmol, 4 mol%) were added sequentially. The reaction mixture was stirred at 30 ℃ for 48 h. To the reaction mixture was added citric acid solution (4 mL, 10 wt.%) and the mixture was stirred for 2 h. The mixture was neutralized with solid K_2CO_3 and extracted with EtOAc (10 mL x 3). The combined extracts were dried over $MgSO_4$ and concentrated in vacuo to afford a residue. The residue was then purified by SiO_2 column chromatography (PE/EA = 5:1 to 1:1) to give the desired product.
Reactions in Table 3 were performed using the above procedure B.

Coupling reaction procedure C: In glove box, $Cu(MeCN)_4PF_6$ (3.7 mg, 0.02 mmol, 10 mol%) and chiral ligand (S,S_p)-**L3** (5.3 mg, 0.022 mmol, 11 mol%) were dissolved in dry THF (1M, 0.2 mL) and stirred at room temperature for 0.5 h. To the solution, substrate aldimine ester **2** (0.2 mmol), Et_3N (0.4 mmol), diene (0.4 mmol) and **L11-Pd** (20.2 mg, 0.016 mmol, 8 mol%) were added sequentially. The reaction mixture was stirred at 40 ℃ for 120 h. To the reaction mixture was added citric acid solution (4 mL, 10 wt.%) and the mixture was stirred for 2 h. The mixture was neutralized with solid K_2CO_3 and extracted with EtOAc (10 mL x 3). The combined extracts were dried over

$MgSO_4$ and concentrated in vacuo to afford a residue. The residue was then purified by SiO_2 column chromatography (PE/EA = 5:1 to 1:1) to give the desired product.

1.10 Synthetic Transformation of the Products

A. methyl (*2S*)-3-(bromomethyl)-2-methyl-5-phenyl-1-tosyl-2, 5-dihydro-1H-pyrrole-2-carboxylate (5aa)

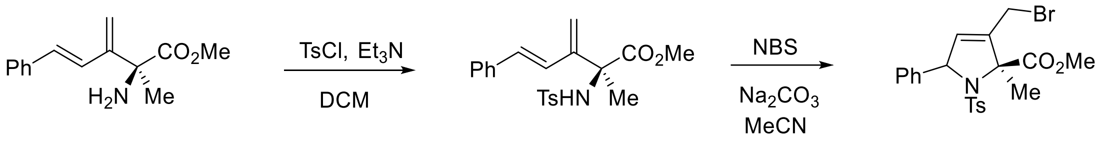

(*S*)-3aa **(*S*)-4aa** **5aa**

A solution of **(*S*)-3aa** (231 mg, 1 mmol, 1.0 equiv.) and triethylamine (278 ul, 2 mmol, 2.0 equiv.) in 6 mL of DCM was cooled to 0 ℃, and TsCl (228 mg, 1.2 mmol, 1.2 equiv.) was added portionwise. The mixture was stirred for 10 min, warmed to room temperature, and then stirred for 24 hours. The mixture was diluted with 5 mL of water, and extracted with DCM (2 x 15 mL). The organic layers were dried over $MgSO_4$. The solvents were removed under reduced pressure and the crude product was purified by flash column chromatography to give the title product **(*S*)-4aa** as a pale yellow solid (225 mg, 58%).

To a solution of **(*S*)-4aa** (77mg, 0.2 mmol, 1.0 equiv.) and MeCN (2 mL, 0.1 M), NBS (44 mg, 0.24 mmol, 1.2 equiv.) and Na_2CO_3 (42 mg, 0.4 mmol, 2.0 equiv.) was added stepwise at room temperature and stirred 6 h. The mixture was diluted with 3 mL of water, and extracted with diethyl ether (3 x 3 mL). The organic layers were dried over $MgSO_4$. The solvents were removed under reduced pressure and the crude product was purified by flash column chromatography afforded **5aa** as a white solid (69.7 mg, 75% yield, 1:1.1 dr, m.p. 63-65 ℃).

^{1}H NMR (400 MHz, $CDCl_3$) δ 7.98 (d, *J* = 8.3 Hz, 2H), 7.36 (d, *J* = 8.2 Hz, 2H), 7.30 – 7.23 (m, 5H), 6.55 (d, *J* = 15.6 Hz, 1H), 6.30 (d, *J* = 15.6 Hz, 1H), 4.24 (d, *J* = 11.1 Hz, 1H), 4.01 (d, *J* = 11.1 Hz, 1H), 3.56 (s, 3H), 2.45 (s, 3H), 2.01 (s, 3H).

^{13}C NMR (101 MHz, $CDCl_3$) δ 167.8, 144.6, 137.6, 135.6, 134.3, 129.6, 128.6, 128.4, 127.7, 126.8, 121.9, 58.3, 56.1, 52.7, 29.6, 21.7, 12.9.

$[\alpha]^{25}_D$ = 6.8 (c 0.6, $CHCl_3$)

HRMS (ESI) calcd. for $C_{21}H_{23}BrNO_4S^+$ $(M + H)^+$: 464.0526, Found: 464.0527

B: methyl (*S*)-2-amino-3-formyl-2-methylbut-3-enoate (6aa)

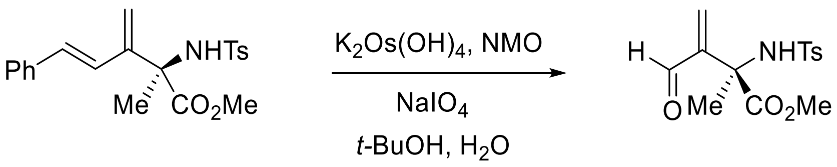

(*S*)-4aa **(*S*)-6aa**

To a solution of **(*S*)-4aa** (77mg, 0.2 mmol, 1.0 equiv.) and *t*-BuOH : H_2O (1.2 mL : 1.2 mL), $K_2Os(OH)_4$ (1.2 mg, 0.0032 mmol, 0.016 equiv.), NMO (70.3 mg, 0.6 mmol,

3.0 equiv.) and $NaIO_4$ (128.3 mg, 0.6 mmol, 3.0 equiv.) was added stepwise at room temperature and stirred 5 h. The mixture was diluted with 3 mL of water, and extracted with diethyl ether (3 x 3 mL). The organic layers were dried over $MgSO_4$. The solvents were removed under reduced pressure and the crude product was purified by flash column chromatography afforded **(*S*)-6aa** as a white solid (53.4 mg, 86% yield m.p. 93-95 ℃).

^{1}H NMR (400 MHz, $CDCl_3$) δ 8.92 (s, 1H), 7.59 (d, *J* = 8.3 Hz, 2H), 7.23 (d, *J* = 8.0 Hz, 2H), 6.62 (s, 1H), 6.15 (s, 1H), 6.04 (s, 1H), 3.67 (s, 3H), 2.40 (s, 3H), 1.77 (s, 3H).

^{13}C NMR (101 MHz, $CDCl_3$) δ 191.5, 172.2, 146.8, 143.3, 138.5, 136.8, 129.3, 127.6, 59.3, 53.4, 22.8, 21.5.

$[\alpha]^{25}_D$ = -35.4 (c 0.5, $CHCl_3$)

HRMS (ESI) calcd. for $C_{14}H_{18}NO_5S^+$ $(M + H)^+$: 312.0901, Found: 312.0898

C: N-((3*S*)-5-(2-(benzyloxy)-1-hydroxyethyl)-3-methyl-4-methylene-2-oxotetrahydrofuran-3-yl)benzamide (5oa)

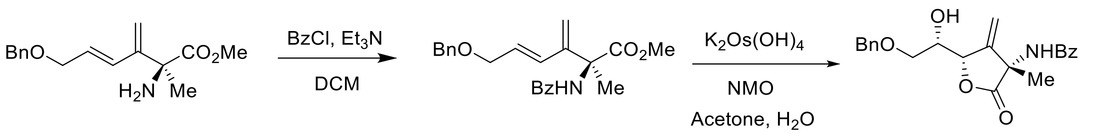

(*S*)-3oa **(*S*)-4oa** **5oa**

A solution of **(*S*)-3oa** (275 mg, 1 mmol, 1.0 equiv.) and triethylamine (278 ul, 2 mmol, 2.0 equiv.) in 5 mL of DCM was cooled to 0 ℃, and BzCl (155 mg, 1.1 mmol, 1.1 equiv.) was added dropwise. The mixture was stirred for 10 min, warmed to room temperature, and then stirred for 12 hours. The mixture was diluted with 5 mL of water, and extracted with DCM (2 x 10 mL). The organic layers were dried over $MgSO_4$. The solvents were removed under reduced pressure and the crude product was purified by flash column chromatography to give the product **(*S*)-4oa** as yellow oil (328 mg, 87%).

To a solution of **(*S*)-4oa** (76mg, 0.2 mmol, 1.0 equiv.) and Acetone : H_2O (1.2 mL :0.4 mL), $K_2Os(OH)_4$ (1.2 mg, 0.0032 mmol, 0.016 equiv.), and NMO (70.3 mg, 0.6 mmol, 3.0 equiv.) was added stepwise at room temperature and stirred 15 h. The mixture was diluted with 3 mL of water, and extracted with diethyl ether (3 x 3 mL). The organic layers were dried over $MgSO_4$. The solvents were removed under reduced pressure and the crude product was purified by flash column chromatography afforded **5oa** as a white solid (58.5 mg, 77% yield, 6:1 dr, m.p. 51-53 ℃).

^{1}H NMR (400 MHz, $CDCl_3$) δ 7.76 (d, *J* = 7.3 Hz, 2H), 7.46 (t, *J* = 7.4 Hz, 1H), 7.41 – 7.25 (m, 7H), 7.23 (s, 1H), 5.32 (s, 1H), 5.27 (s, 1H), 5.10 (s, 1H), 4.63 – 4.47 (m, 2H), 4.03 (s, 1H), 3.67 (d, *J* = 5.9 Hz, 2H), 2.65 (d, *J* = 2.6 Hz, 1H), 1.63 (s, 3H).

^{13}C NMR (101 MHz, $CDCl_3$) δ 176.9, 166.7, 147.9, 137.6, 132.4, 132.2, 128.6, 128.5, 127.9, 127.9, 127.3, 107.6, 81.5, 73.7, 72.0, 71.1, 57.4, 23.9.

$[\alpha]^{25}_D$ = -77.5 (c 1.0, $CHCl_3$)

HRMS (ESI) calcd. for $C_{22}H_{24}NO_5^+$ $(M + H)^+$: 382.1649, Found: 382.1649

2. Supplementary Discussion

2.1 Comprehensive results for reaction condition optimization

Supplementary Table 1. Investigation of the Bases [a]

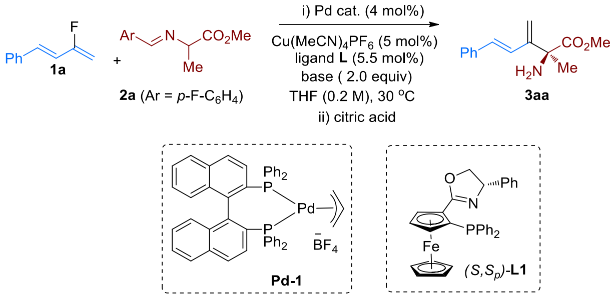

entry	Pd cat.	Base	yield (%)[b]	ee (%)[c]
1	**Pd-1**	Et_3N	53	96
2	**Pd-1**	^{i}Pr_2NEt	trace	N.D
3	**Pd-1**	DBU	NR	-
4	**Pd-1**	DABCO	40	96
5	**Pd-1**	Cs_2CO_3	NR	-

[a]Reaction conditions: i) **1a** (0.2 mmol), **2a** (0.1 mmol), Pd cat. (4 mol%), $Cu(MeCN)_4PF_6$ (5 mol %), (S,S_p)-**L1** (5.5 mol%), base (200 mol%), THF (0.5 mL), 30 ℃, 24 h; ii) citric acid (10%, 4 mL). [b]Isolated yields. NR, no reaction. N.D, not determined. [c]Determined by HPLC.

Supplementary Table 2. Investigation of the Solvents [a]

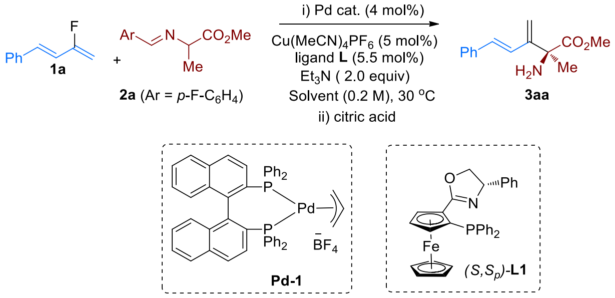

entry	Pd cat.	Solvent	yield (%)[b]	ee (%)[c]
1	**Pd-1**	THF	53	96
2	**Pd-1**	1,4-Dioxane	38	98
3	**Pd-1**	DMF	trace	N.D
4	**Pd-1**	Toluene	trace	N.D
5	**Pd-1**	MeCN	trace	N.D
6	**Pd-1**	DCM	40	96

[a]Reaction conditions: i) **1a** (0.2 mmol), **2a** (0.1 mmol), Pd cat. (4 mol%), $Cu(MeCN)_4PF_6$ (5 mol %), (S,S_p)-**L1** (5.5 mol%), base (200 mol%), THF (0.5 mL), 30 ℃, 24 h; ii) citric acid (10%, 4 mL). [b]Isolated yields. N.D, not determined. [c]Determined by HPLC

Supplementary Table 3. Investigation of the Ligands for Pd [a]

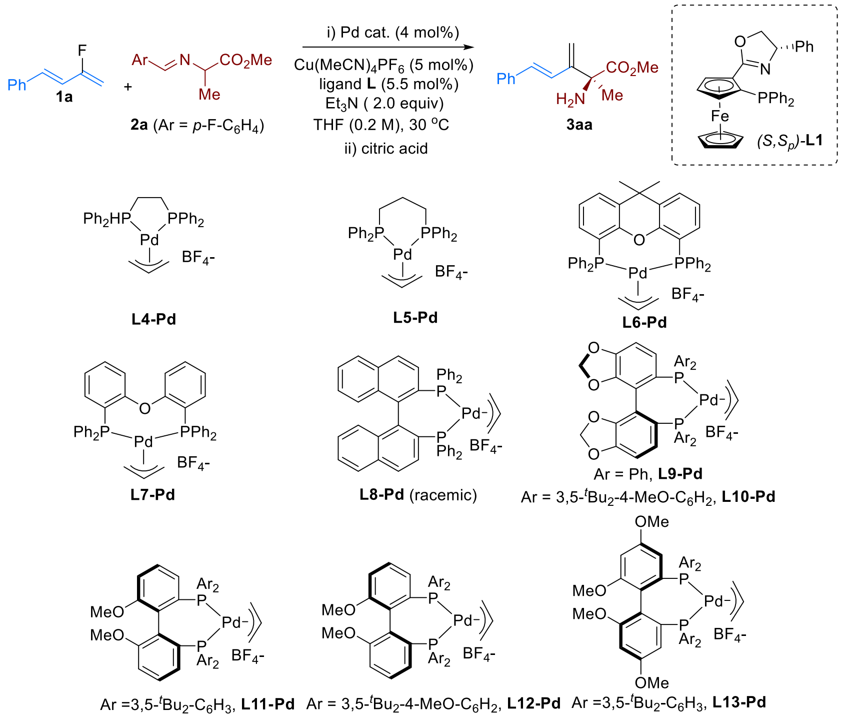

entry	Pd cat.	Cu ligand L	yield (%)[b]	ee (%)[c]
1	**L4-Pd**	(*S*,*S*$_p$)-**L1**	trace	N.D
2	**L5-Pd**	(*S*,*S*$_p$)-**L1**	trace	N.D
3	**L6-Pd**	(*S*,*S*$_p$)-**L1**	trace	N.D
4	**L7-Pd**	(*S*,*S*$_p$)-**L1**	trace	N.D
5	**L8-Pd**	(*S*,*S*$_p$)-**L1**	53	96
6	**L9-Pd**	(*S*,*S*$_p$)-**L1**	50	98
7	**L10-Pd**	(S,*S*$_p$)-**L1**	73	99
8	**L11-Pd**	(*S*,*S*$_p$)-**L1**	87	96
9	**L12-Pd**	(*S*,*S*$_p$)-**L1**	81	99
10	L13-Pd	(*S*,*S*$_p$)-**L1**	70	94

[a]Reaction conditions: i) **1a** (0.2 mmol), **2a** (0.1 mmol), Pd cat. (4 mol%), Cu(MeCN)$_4$PF$_6$ (5 mol %), (*S*,*S*$_p$)-**L1** (5.5 mol%), Et$_3$N (200 mol%), THF (0.5 mL), 30 ℃, 24 h; ii) citric acid (10%, 4 mL). [b]Isolated yields. NR, no reaction. N.D, not determined. [c]Determined by HPLC.

Supplementary Table 4. Investigation of the Ligands for Cu [a]

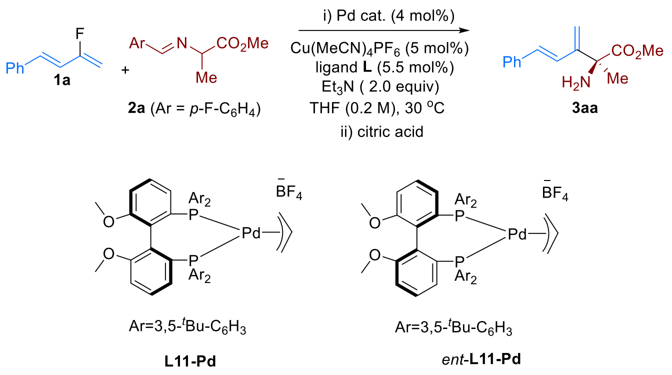

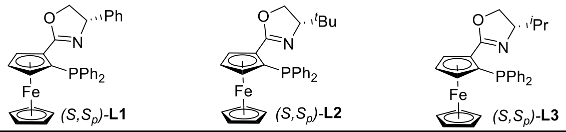

entry	Pd cat.	Cu ligand L	yield (%)[b]	ee (%)[c]
1	**L11-Pd**	(*S*,*S*$_p$)-**L1**	87	96
2	**L11-Pd**	(*S*,*S*$_p$)-**L3**	86	99
3	**L11-Pd**	(*S*,*S*$_p$)-**L2**	75	98
4	***ent*-L11-Pd**	(*S*,*S*$_p$)-**L1**	86	95
5	***ent*-L11-Pd**	(*S*,*S*$_p$)-**L3**	82	93
6	***ent*-L11-Pd**	(*S*,*S*$_p$)-**L2**	82	97
7	-	(*S*,*S*$_p$)-**L3**	NR	-
8	**L11-Pd**	-	NR	-

[a]Reaction conditions: i) **1a** (0.2 mmol), **2a** (0.1 mmol), **L11-Pd**, *ent*-**L11-Pd** (4 mol%), $Cu(MeCN)_4PF_6$ (5 mol %), (*S*,*S*$_p$)-**L** (5.5 mol%), Et_3N (200 mol%), solvent (0.5 mL), 30 ℃, 24 h; ii) citric acid (10%, 4 mL). [b]Isolated yields. NR, no reaction. [c]Determined by HPLC.

2.2 Unsuccessful Substrates

Some more substrates were tested in this study but failed to give desired products efficiently. For the structures of these substrates, see below:

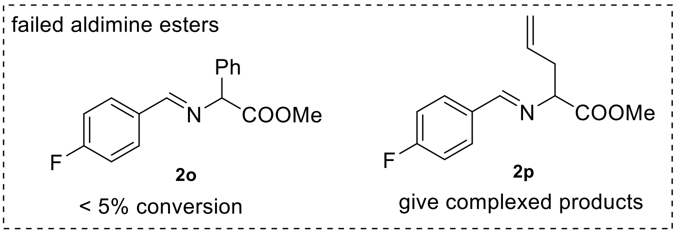

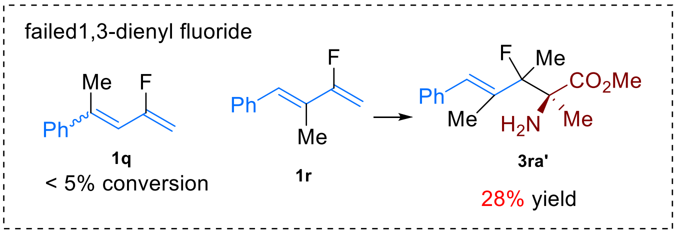

2.3 Scaled-Up experiments

Supplementary Table 5. Scaled-up experiments

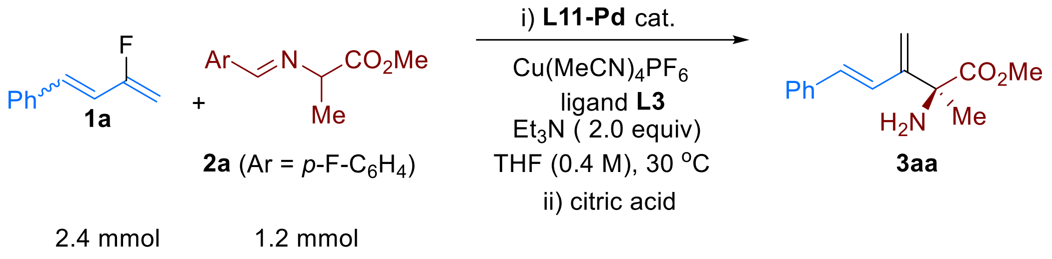

Entry	**L11-Pd** cat.	$Cu(MeCN)_4PF_6$	Ligand **L3**	yield (%)	ee (%)
1	4 mol%	5 mol%	5.5 mol%	86	99
2	2 mol%	2.5 mol%	2.75 mol%	84	99
3	1 mol%	1.25 mol%	1.375 mol%	47	99

In glove box, $Cu(MeCN)_4PF_6$ and chiral ligand (S,S_p)-**L3** were dissolved in dry THF (0.4 M, 3 mL) and stirred at room temperature for 0.5 h. To the solution, substrate aldimine esters **2a** (1.2 mmol), Et_3N (2.4 mmol), dienes **1a** (2.4 mmol) and palladium catalyst **L11-Pd** were added sequentially. The reaction mixture was stirred at 30 ℃ for 24 h. To the reaction mixture was added citric acid solution (24 mL, 10 wt.%) and the mixture was stirred for 2 h. The mixture was neutralized with solid K_2CO_3 and extracted with EtOAc (30 mL x 3). The combined extracts were dried over $MgSO_4$ and concentrated in vacuo to afford a residue. The residue was then purified by SiO_2 column chromatography (PE/EA = 5:1 to 1:1) to give the desired product.

2.4 Rationalization of Ligand Effect of Pd to the Chirality Induction

Based on our previous report [ref 58, *J. Am. Chem. Soc.* **141,** 14554-14559 (2019)], we rationalized that under the *ent*-**L11-Pd/L3-Cu** catalyst system, the diastereoisomeric (2*R*, 3*S*)-**Int 7** is formed. However, during the thereafter defluorination/β-H elimination step the stereochemistry at the C3-position is eliminated; therefore, totally either *ent*-**L11-Pd/L3-Cu** or **L11-Pd/L3-Cu** catalyst system give the same product (2*S*)-**3aa.**

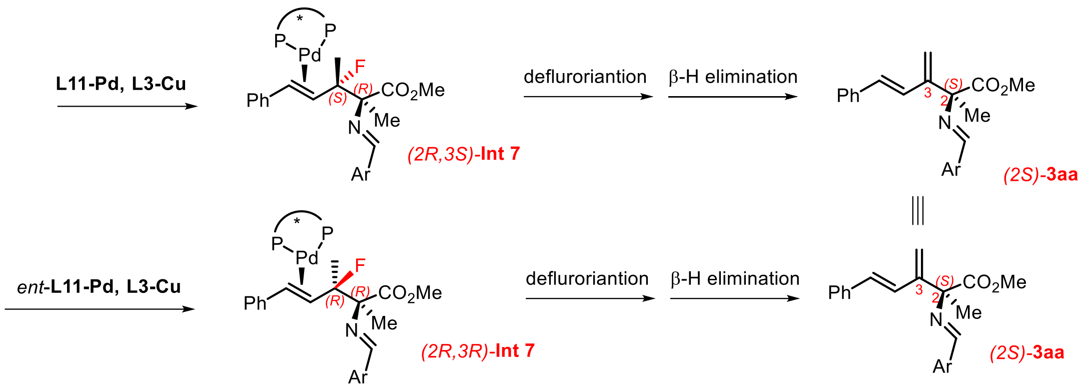

2.5 Control Experiments

A: Coupling of >20:1 or 1:1 *E/Z*-1a with aldimine esters

In glove box, $Cu(MeCN)_4PF_6$ (3.7 mg, 0.01 mmol, 5 mol%) and chiral ligand (*S*,*S*$_p$)-**L3** (5.3 mg, 0.011 mmol, 5.5 mol%) were dissolved in dry THF (0.4 M, 0.5 mL) and stirred at room temperature for 0.5 h. To the solution, substrate aldimine ester **2a** (0.2 mmol), Et_3N (0.4 mmol), >20:1 or 1:1 *E/Z*-**1a** (0.4 mmol) and palladium catalyst **L11-Pd** (10.1 mg, 0.008 mmol, 4 mol%) were added sequentially. The reaction mixture was stirred at 30 ℃ for 24h. To the reaction mixture was added citric acid solution (4 mL, 10 wt.%) and the mixture was stirred for 24 h. The mixture was neutralized with solid K_2CO_3 and extracted with EtOAc (10 mL x 3). The combined extracts were dried over $MgSO_4$ and concentrated in vacuo to afford a residue. The residue was then purified by SiO_2 column chromatography (PE/EA = 5:1 to 1:1) to give > 20:1 *E/Z*-**3aa**.

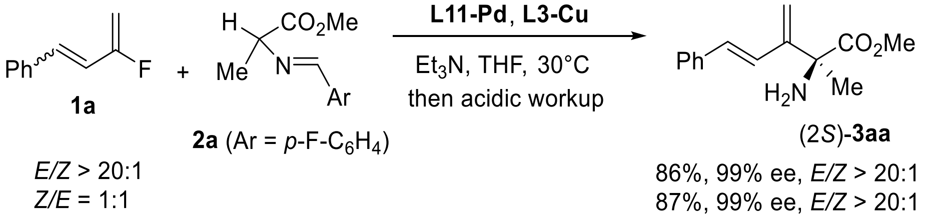

B: The absolute amounts of *Z*-1a and *E*-1a with the change of reaction time

In glove box, $Cu(MeCN)_4PF_6$ (3.7 mg, 0.01 mmol, 5 mol%) and chiral ligand (*S*,*S*$_p$)-**L3** (5.3 mg, 0.011 mmol, 5.5 mol%) were dissolved in dry THF (0.4 M, 0.5 mL) and stirred at room temperature for 0.5 h. To the solution, substrate aldimine ester **2a** (0.2 mmol), Et_3N (0.4 mmol), 1:1 *E/Z*-**1a** (0.4 mmol) and palladium catalyst **L11-Pd** (10.1 mg, 0.008 mmol, 4 mol%) were added sequentially. The reaction mixture was stirred at 30 ℃. Aliquots(10ul) were taken every 3 hours to detemine the absolute *Z*-**1a** and *E*-**1a** by GC-MS.

Reaction time (h)	*Z*-**1a** (mmol)	*E*-**1a** (mmol)
0	0.21	0.19
3	0.18	0.08
6	0.16	0.05
9	0.12	0.07

12	0.10	0.07
24	0.05	0.04

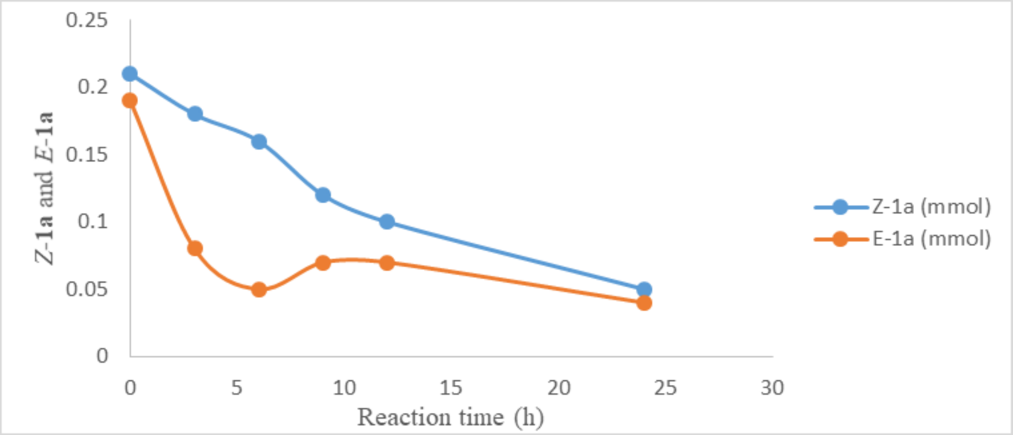

Supplementary Fig. 3 Absolute amounts of Z-1a and E-1a

C. Coupling of dienyl bromide or dienyl chloride with Aldimine Ester

In glove box, $Cu(MeCN)_4PF_6$ (3.7 mg, 0.01 mmol, 5 mol%) and chiral ligand (*S*,*S*$_p$)-**L3** (5.3 mg, 0.011 mmol, 5.5 mol%) were dissolved in dry THF (0.4 M, 0.5 mL) and stirred at room temperature for 0.5 h. To the solution, substrate aldimine ester **2a** (0.2 mmol), Et_3N (0.4 mmol), dienyl bromide **4a** or dienyl chloride **4b** (0.4 mmol) and palladium catalyst **L11-Pd** (10.1 mg, 0.008 mmol, 4 mol%) were added sequentially. The reaction mixture was stirred at 30 ℃ for 24 h. Almost none of the coupling product **3aa** was observed.

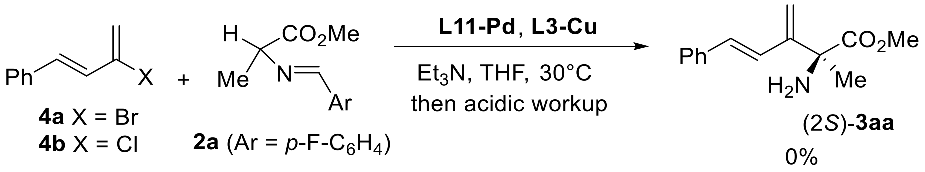

Discussion: We think the PdH insertion step is sensitive to the steric bulk at C-3 position; thereby no corresponding debromo- or dechloro- coupling reaction occurs.

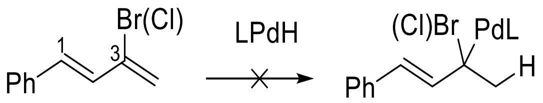

We further checked the coupling of dienyl bromide **4a** or dienyl chloride **4b** with aldimine ester **2a** with other Pd catalysts (shown below). No **3aa** could be observed in all cases. Herein, reaction also did not occur using **4a** or **4b** via the conventional mechanism, involving an oxidative addition, a ligand exchange, and reductive elimination. That's because for such reaction pathway, strong bases such as ^{t}BuOK, Cs_2CO_3, are needed for completely deprotonation of the NuH and at the same time for formation of inorganic salts (KX or CsX). Here in our reaction conditions, Et_3N can't play the role of these strong bases.

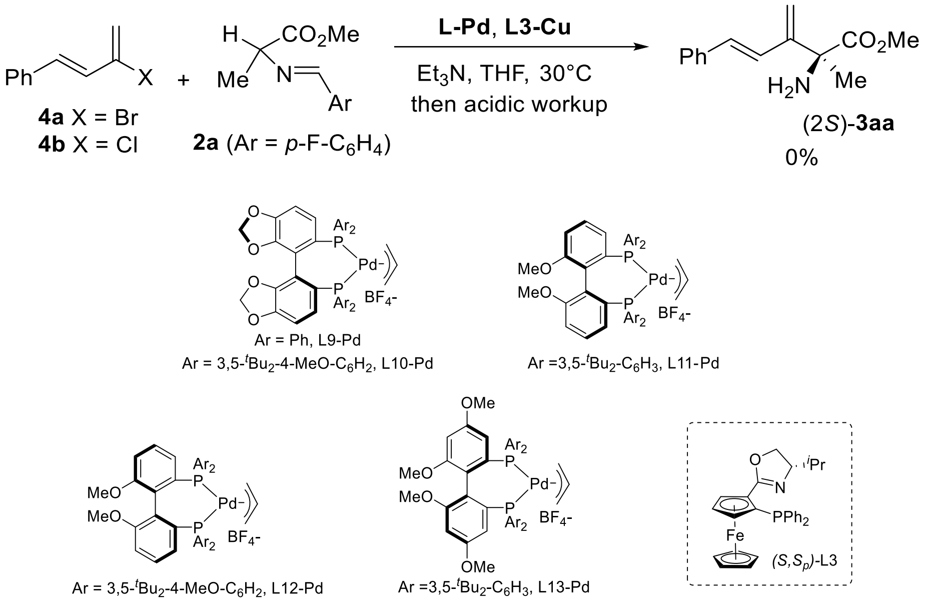

D. Deuterium experiments

To a 50 ml three-neck flask, **2a** (10 mmol, 1.0 equiv.) and K_2CO_3 (15 mmol, 1.5 equiv.) were added. And then 5 mL CH_3OD (98% D) was added under argon. The reaction was stirred at room temperature for 24 h. The organics were filtered and concentrated in vacuo afforded **2a**-*d* as yellow oil. Deuterium rate was determined by ^{1}H NMR analysis in CD_2Cl_2.

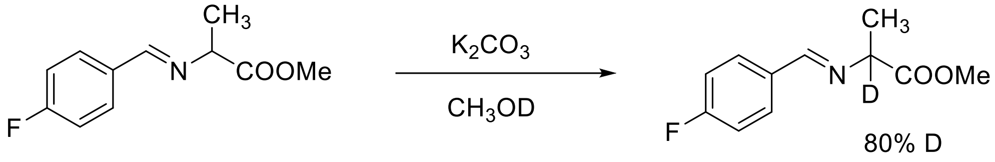

2a **2a**-*d*

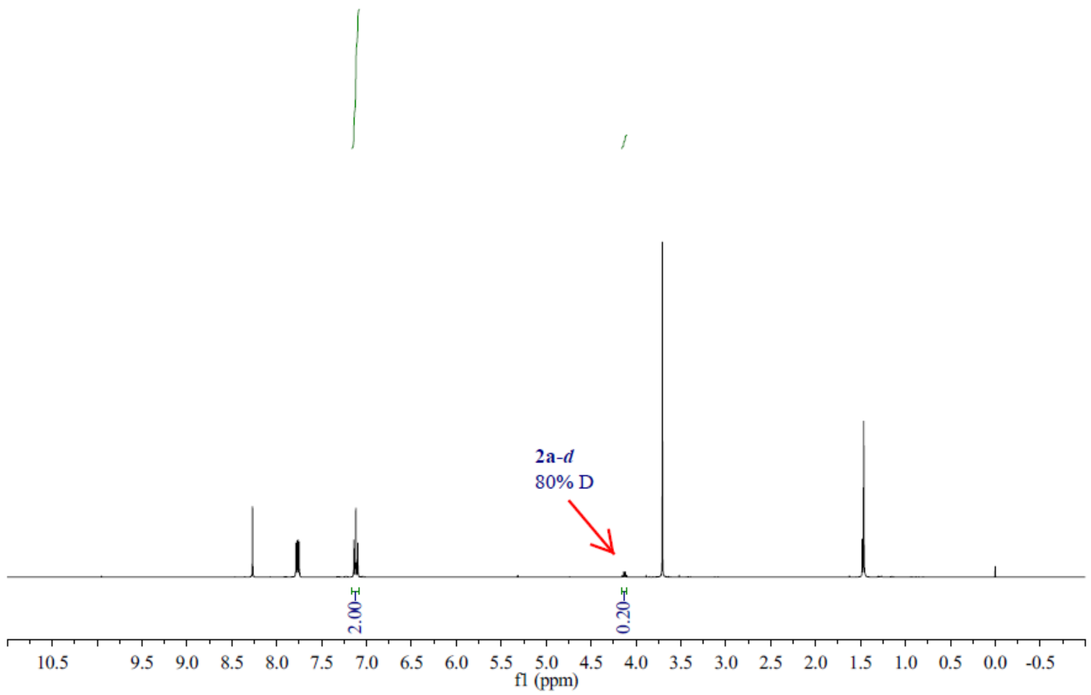

Supplementary Fig. 4. Deuterium rate of 2a-*d*

In glove box, Cu(MeCN)$_4$PF$_6$ (3.7 mg, 0.01 mmol, 5 mol%) and chiral ligand (*S*,*S*$_p$)-**L3** (5.3 mg, 0.011 mmol, 5.5 mol%) were dissolved in dry THF (0.4 M, 0.5 mL) and stirred at room temperature for 0.5 h. To the solution, substrate deuterium-labeled **2a** (80% D, 0.2 mmol), Et$_3$N (0.4 mmol), **1a** (0.4 mmol) and palladium catalyst **L11-Pd** (10.1 mg, 0.008 mmol, 4 mol%) were added sequentially. The reaction mixture was stirred at 30 ℃ for 24h. To the reaction mixture was added citric acid solution (4 mL, 10 wt.%) and the mixture was stirred for 2 h. The mixture was neutralized with solid K$_2$CO$_3$ and extracted with EtOAc (10 mL x 3). The combined extracts were dried over MgSO$_4$ and concentrated in vacuo to afford a residue. The residue was then purified by SiO$_2$ column chromatography (PE/EA = 5:1 to 1:1) to give **3aa** (30% D in total).

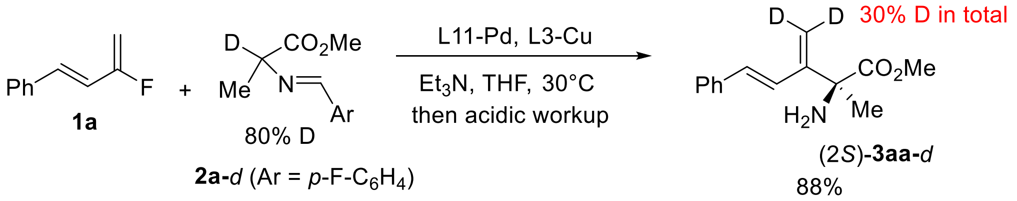

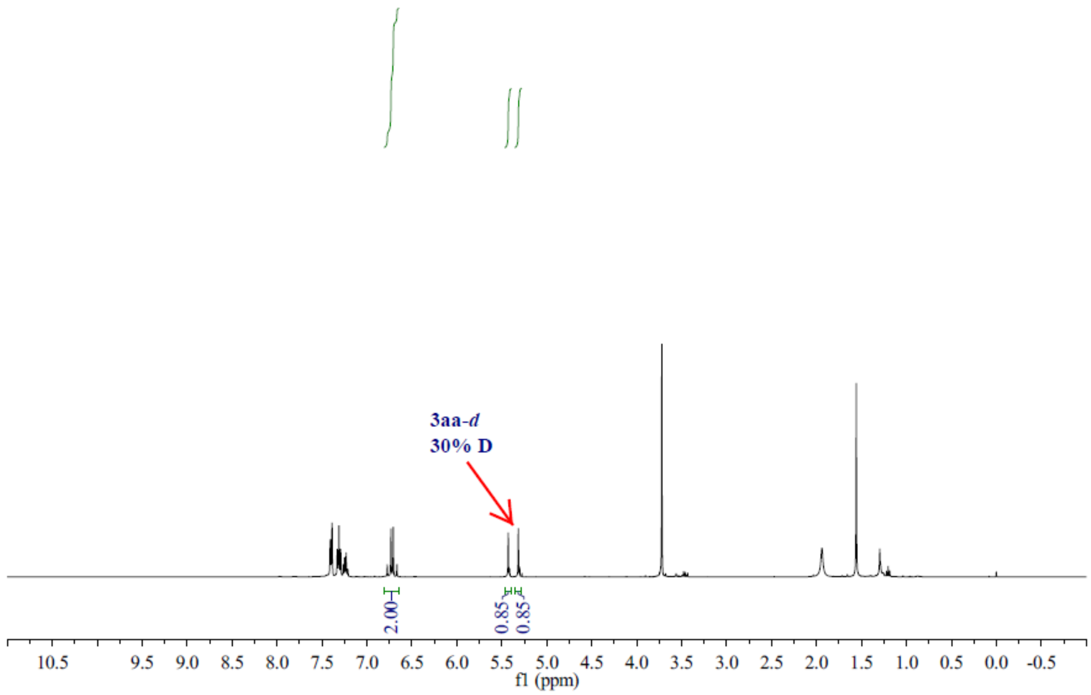

Supplementary Fig. 5. Deuterium rate of 3aa-*d*

Discussion: For the deuterium incorporation experiments shown in the manuscript (Figure 2d), 80% D in the **2a** but resulted in only 30% D incorporation in the product **3aa**. That's because both PdH and PdD were existed in the reaction mixture and played the role of catalyst. However, PdH involved pathway has a large reaction rate compared to the PdD involved pathway (kH > kD). Therefore, **3aa** is generated faster than D-**3aa**. On the other hand, for the PdD involved pathway (path b shown below), the β-hydride elimination step (int 9 to TS 10 in figure 5) might have two possibilities (β-H vs β-D). We can rationalize that the β-H elimination step is a little faster than the β-D elimination step. Thereby PdH is generated after one catalytic cycle (PdD is replaced by PdH after one cycle before entering the second cycle). The above two reasons make that the H atom in substrate 2a (herein is 20% H) was enriched in the product **3aa** (herein is 70% H in **3aa**). Similar phenomenon that loss of deuterium in PdH-catalyzed transformation was also observed in previous report. (*JACS*, **2021**, *143*, 10948-10962)

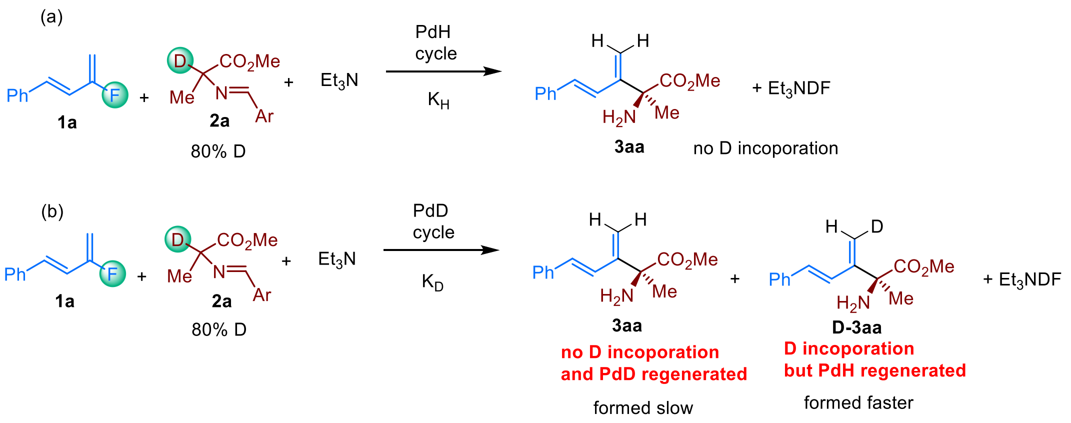

E: Stoichiometric reaction which indicated only one isomeric Cu-azomethine ylide

In glove box, $Cu(MeCN)_4BF_4$ (15.7 mg, 0.05 mmol, 1.0 equiv.) and chiral ligand (S,S_p)-L3 (24.1 mg, 0.05 mmol, 1.0 equiv.) were dissolved in d^8-THF (0.5 mL) and stirred at room temperature for 0.5 h. Cu-3 was used to determined by ^{31}P NMR analysis.

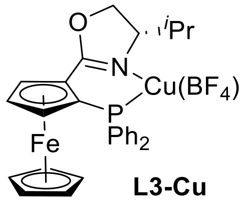

L3-Cu

^{31}P NMR (162 MHz, THF) δ -20.48.

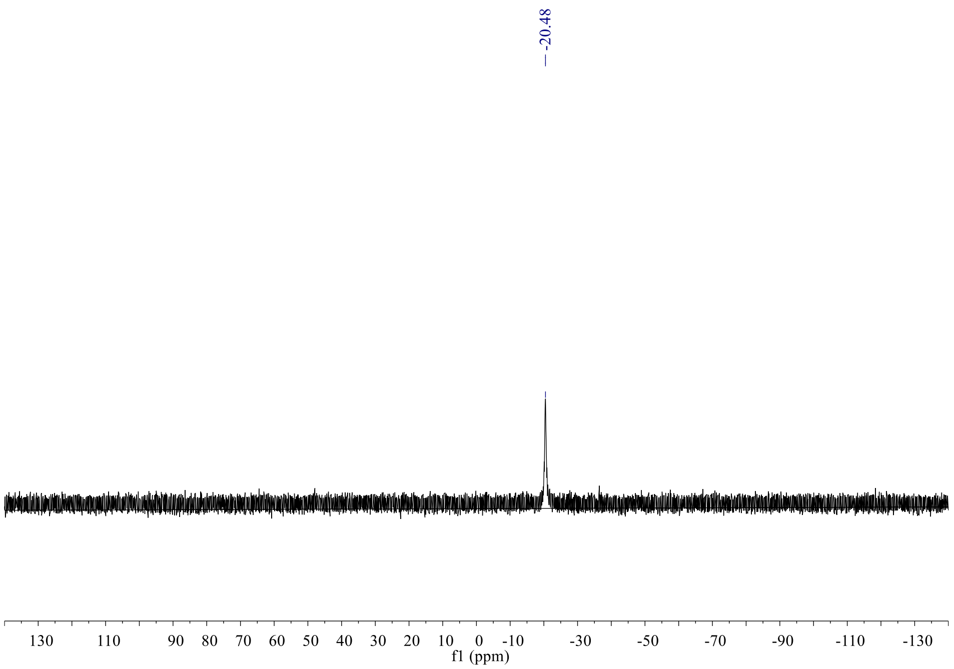

Supplementary Fig. 6. ^{31}P NMR of L3-Cu

In glove box, $Cu(MeCN)_4BF_4$ (15.7 mg, 0.05 mmol, 1.0 equiv.) and chiral ligand (S,S_p)-**L3** (24.1 mg, 0.05 mmol, 1.0 equiv.) were dissolved in d^8-THF (0.5 mL) and stirred at room temperature for 0.5 h. To the solution, substrate **2a** (10.5mg, 0.05 mmol, 1.0 equiv.) was added. The reaction mixture was stirred at 30 ℃ for 2h. Cu-Nu was used to determined by ^{31}P NMR analysis.

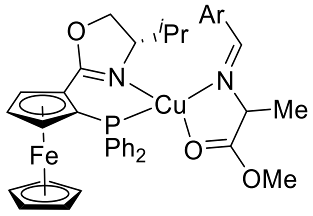

Cu-Nu

^{31}P NMR (162 MHz, THF) δ -20.87.

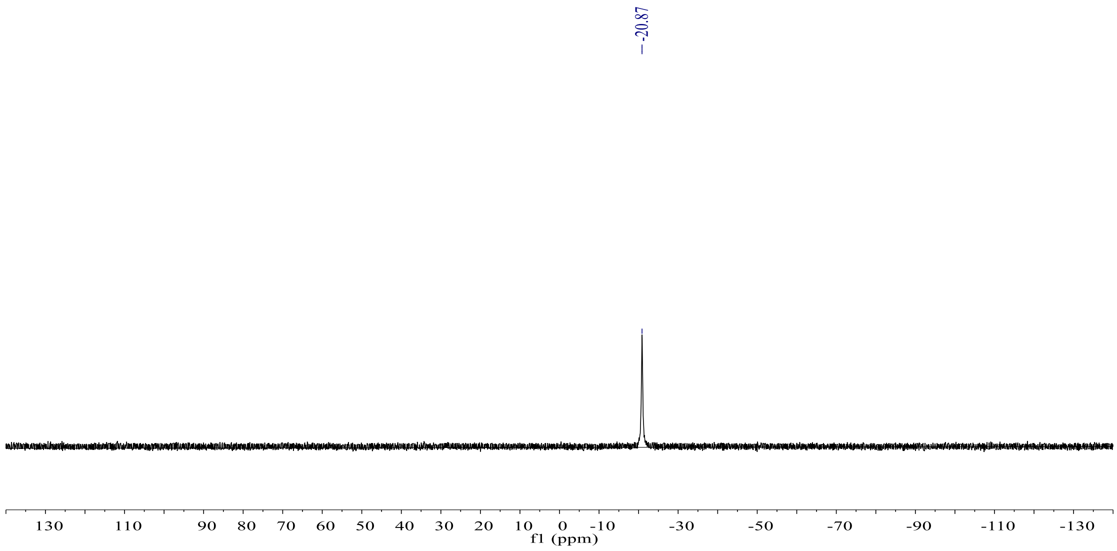

Supplementary Fig. 7. ^{31}P NMR of Cu-Nu

In glove box, $Cu(MeCN)_4BF_4$ (15.7 mg, 0.05 mmol, 1.0 equiv.) and chiral ligand (S,S_P)-**L3** (24.1 mg, 0.05 mmol, 1.0 equiv.) were dissolved in d^8-THF (0.5 mL) and stirred at room temperature for 0.5 h. To the solution, substrate **2a** (10.5 mg, 0.05 mmol, 1.0 equiv.), DBU (7 uL, 0.05 mmol, 1.0 equiv.) were added sequentially. The reaction mixture was stirred at 30 ℃ for 2h. Cu-azomethine ylide was used to determined by ^{31}P NMR analysis.

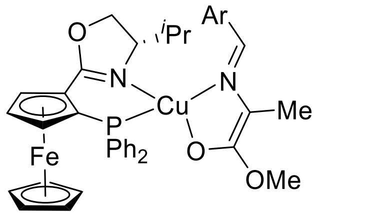

Cu-azomethine ylide

^{31}P NMR (162 MHz, THF) δ -16.09, -21.17.

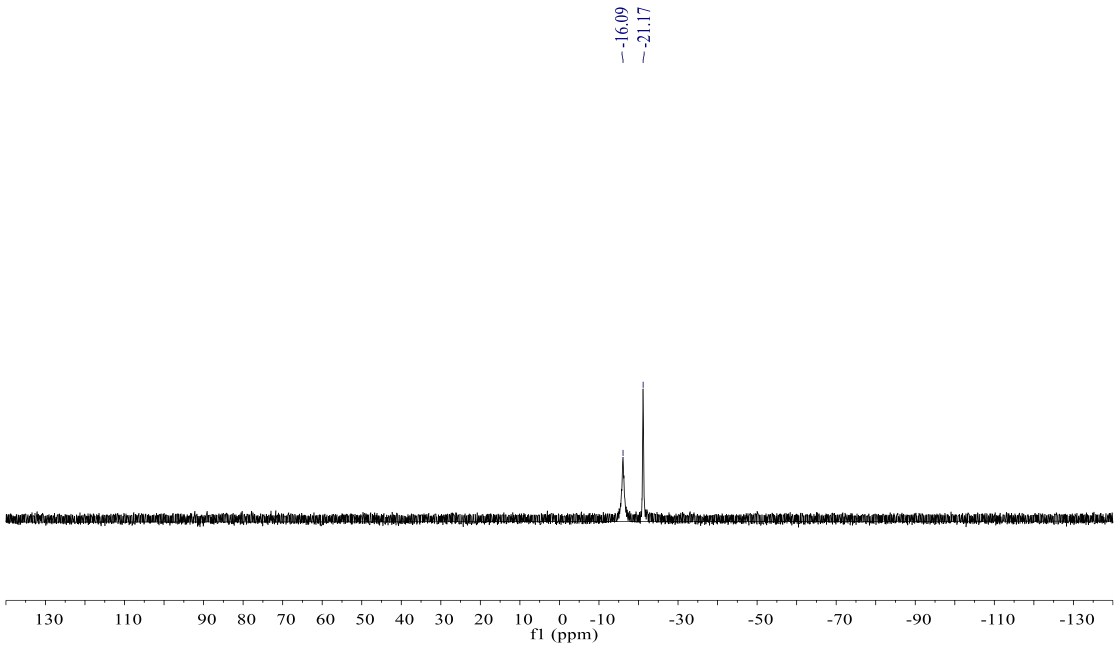

Supplementary Fig. 8. ^{31}P NMR of Cu-azomethine ylide

F: HRMS (ESI) spectrum observed for IX species.

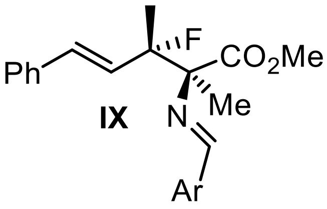

$[M + H]^+ = 252.1395$

observed 252.1393

Analysis Name:	62	Acquisition Date:	20210207
Comment:	ESI Positive	Operator:	TIPC

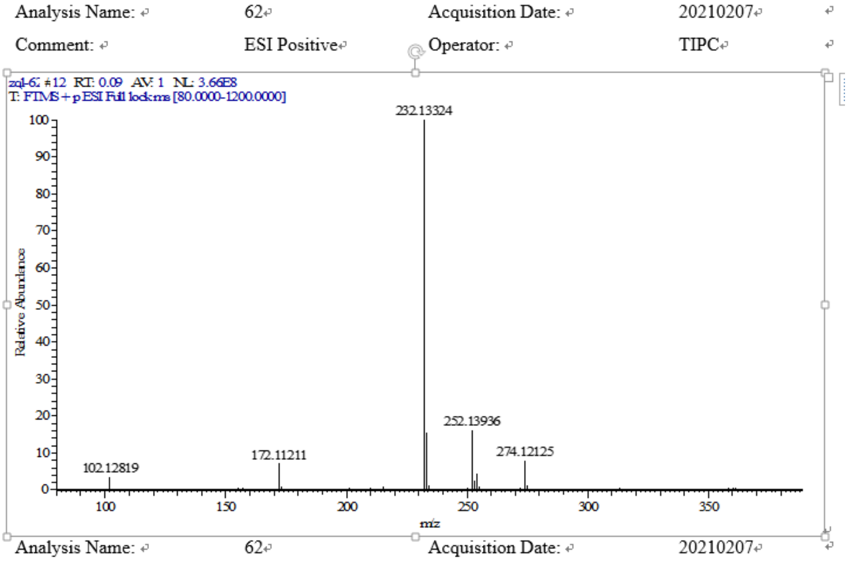

Analysis Name:	62	Acquisition Date:	20210207
Comment:	ESI Positive	Operator:	TIPC

Supplementary Fig. 9. HRMS spectrum for IX species.

2.6 DFT calculation Details

(i) General Information for Computation

All calculations were performed with the Gaussian 16 program. (Full citation of Gaussian 16: M. J. Frisch, G. W. Trucks, H. B. Schlegel, G. E. Scuseria, M. A. Robb, J. R. Cheeseman, G. Scalmani, V. Barone, G. A. Petersson, H. Nakatsuji, X. Li, M. Caricato, A. V. Marenich, J. Bloino, B. G. Janesko, R. Gomperts, B. Mennucci, H. P. Hratchian, J. V. Ortiz, A. F. Izmaylov, J. L. Sonnenberg, D. Williams-Young, F. Ding, F. Lipparini, F. Egidi, J. Goings, B. Peng, A. Petrone, T. Henderson, D. Ranasinghe, V. G. Zakrzewski, J. Gao, N. Rega, G. Zheng, W. Liang, M. Hada, M. Ehara, K. Toyota, R. Fukuda, J. Hasegawa, M. Ishida, T. Nakajima, Y. Honda, O. Kitao, H. Nakai, T. Vreven, K. Throssell, J. A. Montgomery, Jr., J. E. Peralta, F. Ogliaro, M. J. Bearpark, J. J. Heyd, E. N. Brothers, K. N. Kudin, V. N. Staroverov, T. A. Keith, R. Kobayashi, J. Normand, K. Raghavachari, A. P. Rendell, J. C. Burant, S. S. Iyengar, J. Tomasi, M. Cossi, J. M. Millam, M. Klene, C. Adamo, R. Cammi, J. W. Ochterski, R. L. Martin, K. Morokuma, O. Farkas, J. B. Foresman, and D. J. Fox, Gaussian, Inc., Wallingford CT, 2016. Gaussian 16, Revision A.03).

Geometry optimizations were conducted with the Gaussian 16 software package, B3LYP functional [with gd3(BJ) dispersion correction], and LANL2DZ basis set for Pd and 6-31g(d) basis set for all other atoms. Single-point energy calculations were conducted with the M06-2X functional and def2-TZVP basis set for atoms, along with the SMD CH2ClCH2Cl solvent correction. Concentration was corrected from 1 atm to 1 mol/L by addition 1.89 kcal/mol to the Gibbs energy of each species. Reaction paths were traced by the intrinsic reaction coordinate method for all transition states. All energetics reported throughout the text are in kcal/mol.

(ii) Comparison of the Energy Profile with Different Calculation Methods.

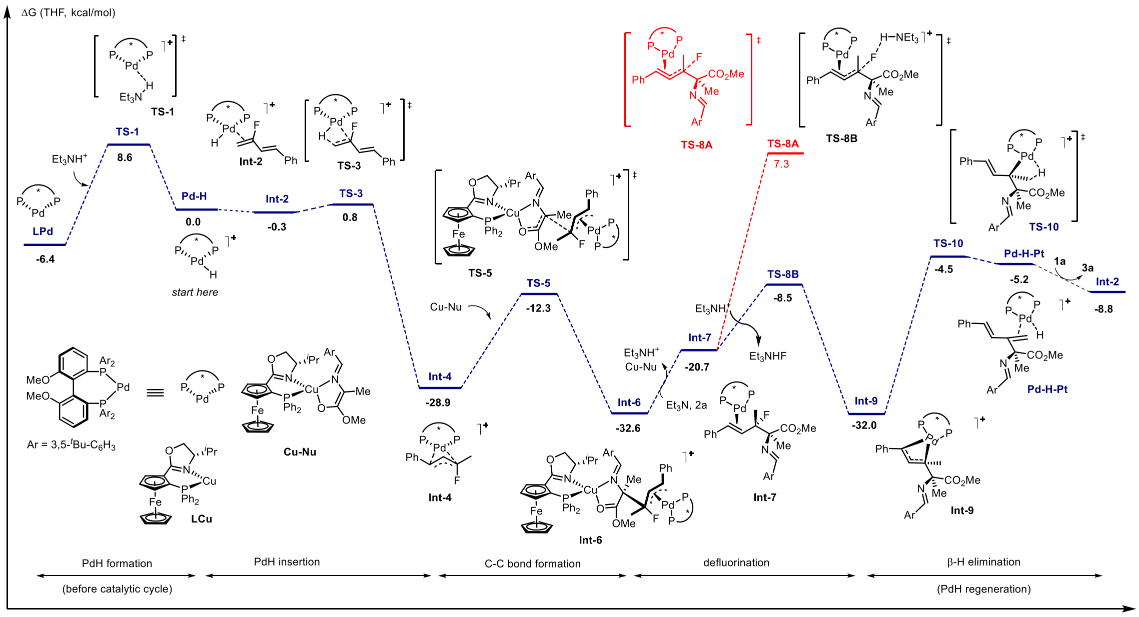

Supplementary Fig. 10. Energy profile for the proposed mechanism. Calculations were carried out at the M06-2x(SMD)/def2-TZVP//B3LYP-D3BJ/6-31g(d)/Lanl2dz level of theory.

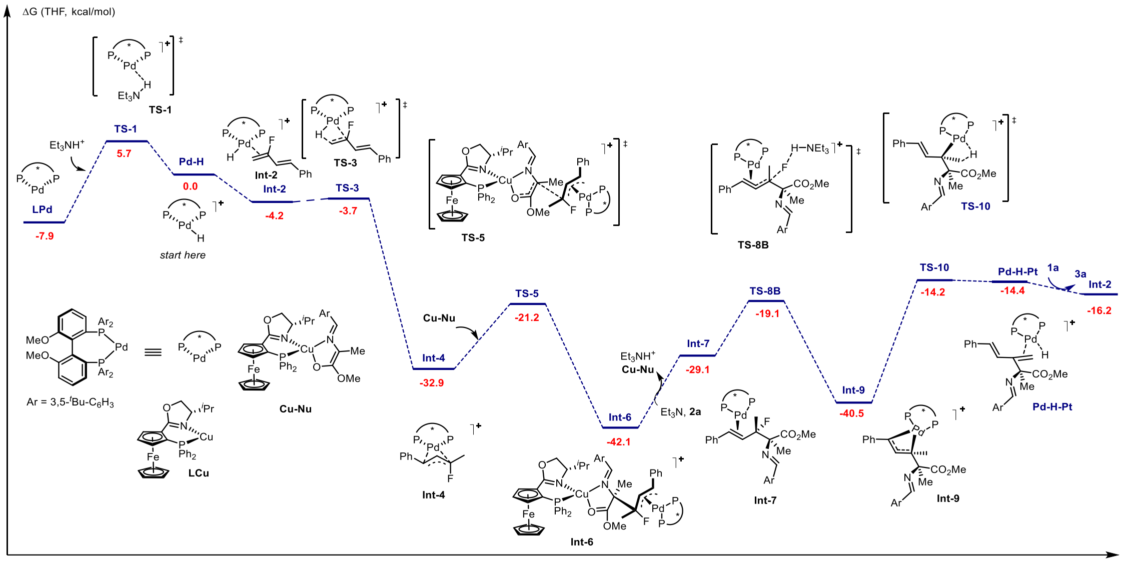

Supplementary Fig. 11. Energy profile for the proposed mechanism. Calculations were carried out at the M06-2x(SMD)/6-311++g(d,p)/SDD//B3LYP-D3BJ/6-31g(d)/Lanl2dz level of theory.

Supplementary Table 6. Comparison of the energy barriers with the two methods in the key steps.

Energy Barrier	M06-2x(SMD)/def2-TZVP //B3LYP-D3BJ/6-31g(d)/Lanl2dz	M06-2x(SMD)/6-311++g(d,p)/SDD //B3LYP-D3BJ/6-31g(d)/Lanl2dz
Pd-H formation	ΔG = 15.0 kcal/mol	ΔG = 13.6 kcal/mol
Pd-H insertion	ΔG = 1.1 kcal/mol	ΔG = 0.5 kcal/mol
C-C formation	ΔG = 16.6 kcal/mol	ΔG = 11.7 kcal/mol

defluorination	ΔG = 24.1 kcal/mol	ΔG = 23.0 kcal/mol
β-H elimination	ΔG = 27.5 kcal/mol	ΔG = 26.3 kcal/mol

(iii) Comparison of the ΔΔG of C-C bond formation step from different calculation methods.

Supplementary Table 7. Comparison different calculation methods for the C-C bond formation step.

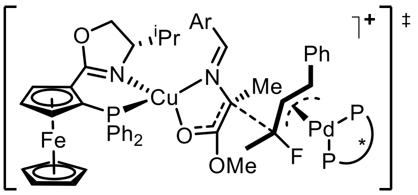

Cu-Nu(*si*-face) approach

TS-5

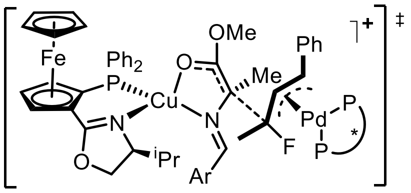

Cu-Nu(*re*-face) approach

TS-5-II

M06-2x(SMD)/def2-TZVP//B3LYP-D3BJ/6-31g(d)/Lanl2dz	**ΔΔG = 5.0**
M06-2x(SMD)/def2-TZVPP//B3LYP-D3BJ/6-31g(d)/Lanl2dz	**ΔΔG = 5.1**
M06-2x(SMD)/6-311++g(d,p)/SDD//B3LYP-D3BJ/6-31g(d)/Lanl2dz	**ΔΔG = 6.0**
B3LYP-D3BJ(SMD)/6-311++g(d,p)/SDD//B3LYP-D3BJ/6-31g(d)/Lanl2dz	**ΔΔG = 8.2**

(iv) Calculated Energy barrier with different methods for the β-H elimination step

Supplementary Table 8. Comparison different calculation methods for the Cβ-H elimination step.

Methods	Energy barrier
M06-2x(SMD)/def2-TZVP//B3LYP-D3BJ/6-31g(d)/Lanl2dz	ΔG = 27.5 kcal/mol
M06-2x(SMD)/6-311++g(d,p)/SDD//B3LYP-D3BJ/6-31g(d)/Lanl2dz	ΔG = 26.3 kcal/mol
wB97X-D(SMD)/def2-TZVP//B3LYP-D3BJ/6-31g(d)/Lanl2dz	ΔG = 30.0 kcal/mol
B3LYP-D3BJ(SMD)/def2-TZVP//B3LYP-D3BJ/6-31g(d)/Lanl2dz	ΔG = 26.6 kcal/mol
M06-2x(SMD)/def2-TZVPP//B3LYP-D3BJ/6-31g(d)/Lanl2dz	ΔG = 27.2 kcal/mol
B3LYP-D3BJ(SMD)/6-311++g(d,p)/SDD//B3LYP-D3BJ/6-31g(d)/Lanl2dz	ΔG = 25.6 kcal/mol

(v) Calculated ΔΔG of C-C bond formation step from Cu(S)-Nu nucleophile

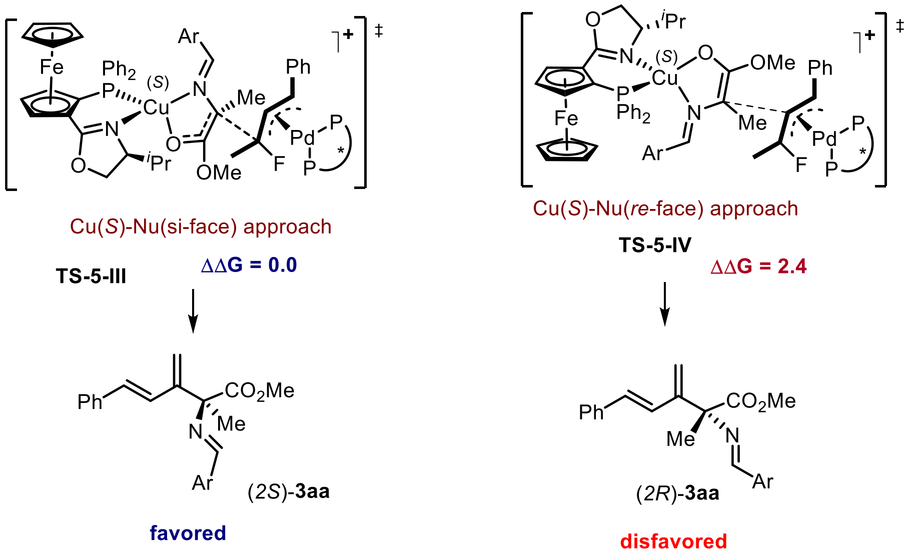

3. Supplementary Data

3.1 Compound Characterization

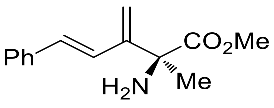

(S)-**3aa**

(*S*,*E*)-methyl 2-amino-2-methyl-3-methylene-5-phenylpent-4-enoate

Prepared according to General Procedure A using **L11-Pd** and (*S*,*S*$_p$)-**L3-Cu**, Purification by flash chromatography (PE/EA = 5:1 to 1:1) afforded the product, pale yellow oil, 86% yield , 99% ee, R_f = 0.4 (PE/EA, 1:1).

$[\alpha]^{25}_D$ = 19.1 (c 1.0, $CHCl_3$)

^{1}H NMR (400 MHz, $CDCl_3$) δ 7.40 (d, *J* = 7.4 Hz, 2H), 7.31 (t, *J* = 7.5 Hz, 2H), 7.27 – 7.19 (m, 1H), 6.72 (q, *J* = 16.3 Hz, 2H), 5.43 (s, 1H), 5.31 (s, 1H), 3.72 (s, 3H), 2.01 (s, 2H), 1.56 (s, 3H).

^{13}C NMR (101 MHz, $CDCl_3$) δ 176.6, 148.7, 136.9, 130.5, 128.6, 127.8, 126.8, 126.6, 112.3, 60.50, 52.6, 25.7.

HRMS (ESI) calcd. for $C_{14}H_{20}NO_2^+$ $(M + H)^+$: 232.1338, Found: 232.1331

the ee value was 99%, t_r (major) = 25.016 min, t_r (minor) = 34.565 min, (Chiralcel IC, λ = 220 nm, hexanes : ^{i}PrOH = 98 : 2, flow rate = 1.0 mL/min).

(检测器 A = **detector A,** 总计 = **total**)

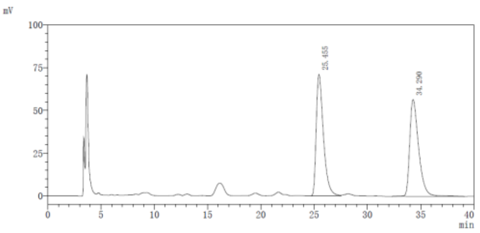

检测器A 220nm

Peak#	Time	Area	Height	Height%	Area%
1	25.455	3345828	71212	55.701	50.349
2	34.290	3299500	56634	44.299	49.651
总计		6645328	127845	100.000	100.000

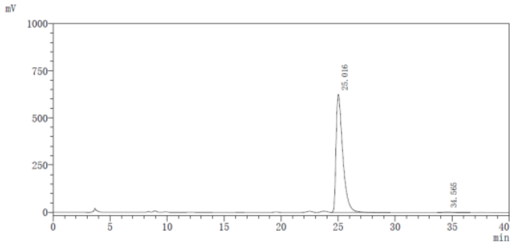

检测器A 220nm

Peak#	Time	Area	Height	Height%	Area%
1	25.016	25673738	626726	99.596	99.454
2	34.565	140962	2543	0.404	0.546
总计		25814700	629269	100.000	100.000

Supplementary Fig. 12 HPLC chromatogram for compound (S)-3aa

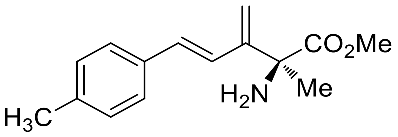

(S)-**3ba**

(*S*,*E*)-methyl 2-amino-2-methyl-3-methylene-5-(p-tolyl)pent-4-enoate

Prepared according to General Procedure A using **L11-Pd** and (*S*,*S*$_p$)-**L3-Cu**, Purification by flash chromatography (PE/EA = 5:1 to 1:1) afforded the product as a pale yellow oil, 39.5 mg, 81 % yield, 98% ee, R_f = 0.4 (PE/EA, 1:1).

$[\alpha]^{25}_D$ = 5.3 (c 1.0, $CHCl_3$)

^{1}H NMR (400 MHz, $CDCl_3$) δ 7.29 (d, *J* = 8.0 Hz, 2H), 7.12 (d, *J* = 7.9 Hz, 2H), 6.68 (q, *J* = 16.2 Hz, 2H), 5.40 (s, 1H), 5.29 (s, 1H), 3.72 (s, 3H), 2.33 (s, 3H), 1.96 (s, 2H), 1.55 (s, 3H).

^{13}C NMR (101 MHz, $CDCl_3$) δ 176.6, 148.8, 137.7, 134.2, 130.4, 129.3, 126.5, 125.7, 111.8, 60.54, 52.6, 25.8, 21.2.

HRMS (ESI) calcd. for $C_{14}H_{20}NO_2^+$ $(M + H)^+$: 246.1494, Found: 246.1486

the ee value was 98%, t_r (major) = 16.486 min, t_r (minor) = 23.556 min (Chiralcel IC, λ = 220 nm, hexanes : ^{i}PrOH = 95 : 5, flow rate = 1.0 mL/min).

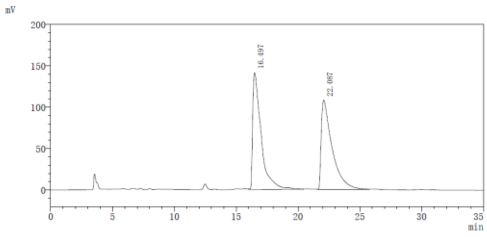

检测器A 220nm

Peak#	Time	Area	Height	Height%	Area%
1	16.497	6696896	141211	56.746	50.673
2	22.087	6519053	107637	43.254	49.327
总计		13215949	248849	100.000	100.000

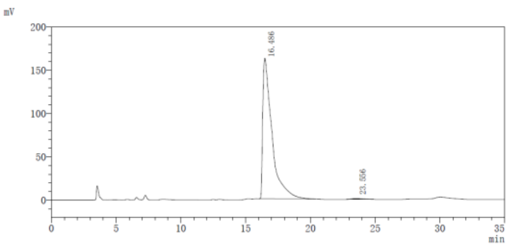

检测器A 220nm

Peak#	Time	Area	Height	Height%	Area%
1	16.486	8040140	162226	99.420	99.169
2	23.556	67401	946	0.580	0.831
总计		8107541	163172	100.000	100.000

Supplementary Fig. 13 HPLC chromatogram for compound (S)-3ba

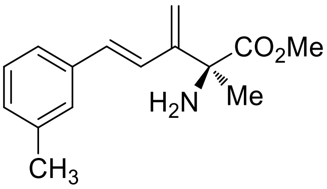

(S)-**3ca**

(*S*,*E*)-methyl 2-amino-2-methyl-3-methylene-5-(m-tolyl)pent-4-enoate

Prepared according to General Procedure A using **L11-Pd** and (*S*,*S*$_P$)-**L3-Cu**, Purification by flash chromatography (PE/EA = 5:1 to 1:1) afforded the product as a pale yellow oil, 38 mg, 79% yield, >99% ee, R_f = 0.4 (PE/EA, 1:1).

$[\alpha]^{25}_D$ = 20.7 (c 1.0, $CHCl_3$)

^{1}H NMR (400 MHz, $CDCl_3$) δ 7.20 (d, *J* = 4.8 Hz, 3H), 7.05 (s, 1H), 6.79 – 6.63 (m, 2H), 5.41 (s, 1H), 5.30 (s, 1H), 3.72 (s, 3H), 2.34 (s, 3H), 1.94 (s, 2H), 1.56 (s, 3H).

^{13}C NMR (101 MHz, $CDCl_3$) δ 176.6, 148.8, 138.2, 136.9, 130.6, 128.6, 128.5, 127.3, 126.5, 123.8, 112.1, 60.5, 52.6, 25.8, 21.4.

HRMS (ESI) calcd. for $C_{14}H_{20}NO_2^+$ $(M + H)^+$: 246.1494, Found: 246.1485

the ee value was > 99%, t_r (major) = 14.677 min, t_r (minor) = 18.779 min (Chiralcel IC, λ = 220 nm, hexanes : ^{i}PrOH = 95 : 5, flow rate = 1.0 mL/min).

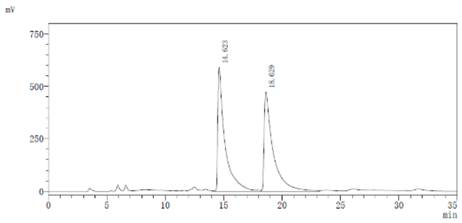

检测器A 220nm

Peak#	Time	Area	Height	Height%	Area%
1	14.623	25346771	591197	55.519	50.156
2	18.629	25189056	473658	44.481	49.844
总计		50535828	1064855	100.000	100.000

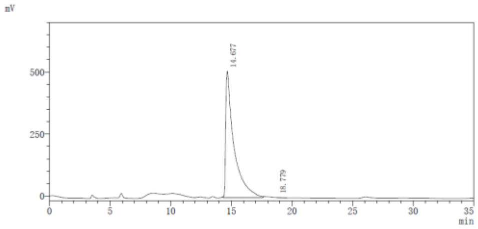

检测器A 220nm

Peak#	Time	Area	Height	Height%	Area%
1	14.677	23866691	509293	99.781	99.918
2	18.779	19547	1116	0.219	0.082
总计		23886239	510408	100.000	100.000

Supplementary Fig. 14 HPLC chromatogram for compound (S)-3ca

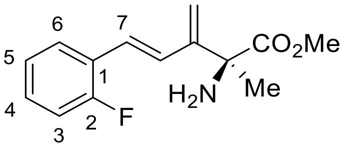

(S)-**3da**

(*S*,*E*)-methyl 2-amino-5-(2-fluorophenyl)-2-methyl-3-methylenepent-4-enoate

Prepared according to General Procedure A using **L11-Pd** and (*S*,*S*$_P$)-**L3-Cu**, Purification by flash chromatography (PE/EA = 5:1 to 1:1) afforded the product as a pale yellow oil, 40 mg, 80% yield, 98% ee, R_f = 0.4 (PE/EA, 1:1).

$[\alpha]^{25}_D$ = 7.0 (c 1.0, $CHCl_3$)

^{1}H NMR (400 MHz, $CDCl_3$) δ 7.47 (t, *J* = 7.5 Hz, 1H), 7.20 (dd, *J* = 12.9, 6.6 Hz, 1H), 7.09 (t, *J* = 7.4 Hz, 1H), 7.06 – 6.99 (m, 1H), 6.84 (dd, *J* = 61.6, 16.4 Hz, 2H), 5.46 (s, 1H), 5.34 (s, 1H), 3.74 (s, 3H), 1.89 (s, 2H), 1.56 (s, 3H).

^{13}C NMR (101 MHz, $CDCl_3$) δ 176.4, 160.4 (d, *J* = 249.7 Hz, C2), 148.9, 129.0, 129.0 (d, *J* = 14.3 Hz, C4), 127.2 (d, *J* = 3.3 Hz, C6), 124.9 (d, *J* = 12.1 Hz, C7), 124.1 (d, *J* = 3.4 Hz, C5), 122.8 (d, *J* = 3.7 Hz, C1), 115.7 (d, *J* = 22.1 Hz, C3), 112.7, 60.5, 52.6, 25.8.

^{19}F NMR (376 MHz, DMSO) δ -122.5.
HRMS (ESI) calcd. for $C_{14}H_{20}NO_2^+$ $(M + H)^+$: 250.1243, Found: 250.1237

the ee value was 98%, t_r (major) = 15.914 min, t_r (minor) = 19.070 min (Chiralcel IC, λ = 220 nm, hexanes : ^{i}PrOH = 95 : 5, flow rate = 1.0 mL/min).

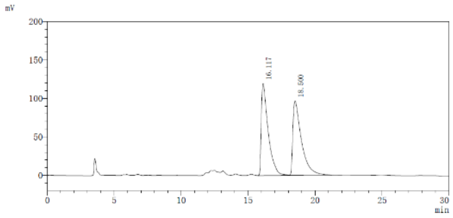

检测器A 220nm

Peak#	Time	Area	Height	Height%	Area%
1	16.117	4308690	119747	55.244	49.648
2	18.500	4369700	97013	44.756	50.352
总计		8678390	216759	100.000	100.000

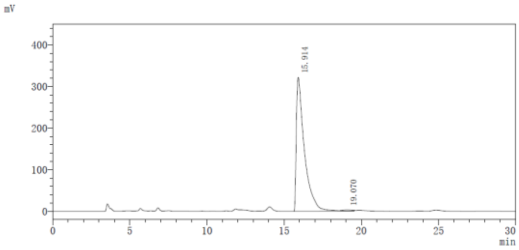

检测器A 220nm

Peak#	Time	Area	Height	Height%	Area%
1	15.914	11718253	321847	99.138	99.058
2	19.070	111386	2798	0.862	0.942
总计		11829640	324646	100.000	100.000

Supplementary Fig. 15 HPLC chromatogram for compound (S)-3da

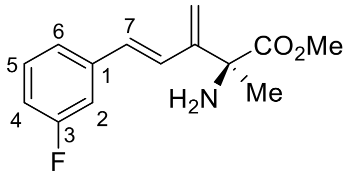

(S)-**3ea**

(*S*,*E*)-methyl 2-amino-5-(3-fluorophenyl)-2-methyl-3-methylenepent-4-enoate
Prepared according to General Procedure A using **L11-Pd** and (*S*,*S$_p$*)-**L3-Cu**, Purification by flash chromatography (PE/EA = 5:1 to 1:1) afforded the product as a pale yellow oil, 40.2 mg, 81% yield, 98% ee, R_f = 0.4 (PE/EA, 1:1).
$[\alpha]^{25}_D$ = 8.6 (c 0.5, $CHCl_3$)
^{1}H NMR (400 MHz, $CDCl_3$) δ 7.27 (dd, *J* = 13.8, 7.9 Hz, 1H), 7.13 (dd, *J* = 21.7, 8.9 Hz, 2H), 6.93 (t, *J* = 8.2 Hz, 1H), 6.71 (s, 2H), 5.44 (s, 1H), 5.35 (s, 1H), 3.73 (s, 3H), 1.91 (s, 2H), 1.56 (s, 3H).
^{13}C NMR (101 MHz, $CDCl_3$) δ 176.4, 163.1 (d, *J* = 245.3 Hz, C3), 148.4, 139.4 (d, *J* = 7.6 Hz, C7), 130.0 (d, *J* = 8.4 Hz, C1), 129.3 (d, *J* = 2.4 Hz, C5), 128.2, 122.5 (d, *J* = 2.4 Hz, C6), 114.6 (d, *J* = 21.4 Hz, C4), 113.1, 112.9 (d, *J* = 21.9 Hz, C2), 60.5, 52.7, 25.8.
^{19}F NMR (377 MHz, $CDCl_3$) δ -113.4.
HRMS (ESI) calcd. for $C_{14}H_{20}NO_2^+$ $(M + H)^+$: 250.1243, Found: 250.1236

the ee value was 98%, t_r (major) = 13.172 min, t_r (minor) = 15.572 min (Chiralcel IC, λ = 220 nm, hexanes : ^{i}PrOH = 95 : 5, flow rate = 1.0 mL/min).

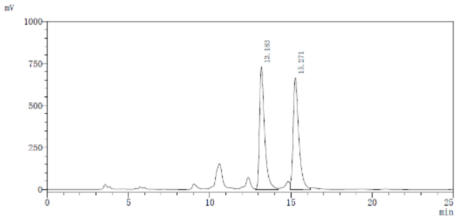

检测器A 220nm

Peak#	Time	Area	Height	Height%	Area%
1	13.183	15976874	731773	52.351	49.967
2	15.271	15997842	666041	47.649	50.033
总计		31974715	1397814	100.000	100.000

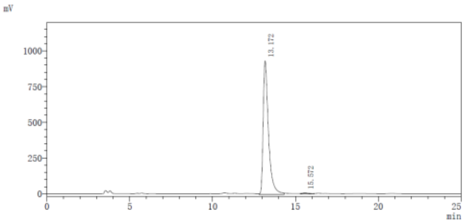

检测器A 220nm

Peak#	Time	Area	Height	Height%	Area%
1	13.172	20959703	936358	99.336	99.150
2	15.572	179717	6259	0.664	0.850
总计		21139420	942617	100.000	100.000

Supplementary Fig. 16 HPLC chromatogram for compound (S)-3ea

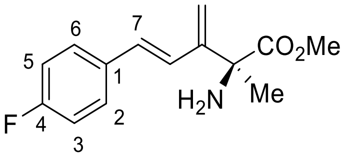

(S)-**3fa**

(*S*,*E*)-methyl 2-amino-5-(4-fluorophenyl)-2-methyl-3-methylenepent-4-enoate

Prepared according to General Procedure A using **L11-Pd** and (*S*,*S*$_p$)-**L3-Cu**, Purification by flash chromatography (PE/EA = 5:1 to 1:1) afforded the product as a pale yellow oil 43.5mg, 87% yield, 99% ee, R_f = 0.4 (PE/EA, 1:1).

$[\alpha]^{25}_D$ = 13.3 (c 1.0, $CHCl_3$)

^{1}H NMR (400 MHz, $CDCl_3$) δ 7.36 (dd, *J* = 8.6, 5.5 Hz, 2H), 7.00 (t, *J* = 8.7 Hz, 2H), 6.66 (dd, *J* = 45.1, 16.2 Hz, 2H), 5.41 (s, 1H), 5.31 (s, 1H), 3.73 (s, 3H), 1.88 (s, 2H), 1.56 (s, 3H).

^{13}C NMR (101 MHz, $CDCl_3$) δ 176.5, 162.4 (d, *J* = 247.5 Hz, C4), 148.6, 133.2 (d, *J* = 3.3 Hz, C7), 129.3, 128.1 (d, *J* = 7.9 Hz, C2 and C6), 126.6 (d, *J* = 2.0 Hz, C1), 115.5 (d, *J* = 21.7 Hz, C3 and C5), 112.3, 60.5, 52.6, 25.8.

^{19}F NMR (377 MHz, $CDCl_3$) δ -113.9.

HRMS (ESI) calcd. for $C_{14}H_{20}NO_2^+$ $(M + H)^+$: 250.1243, Found: 250.1235

the ee value was 99%, t_r (major) = 13.830 min, t_r (minor) = 16.528 min (Chiralcel IC, λ = 220 nm, hexanes : ^{i}PrOH = 95 : 5, flow rate = 1.0 mL/min).

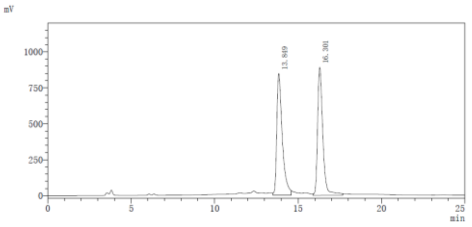

检测器A 220nm

Peak#	Time	Area	Height	Height%	Area%
1	13.849	19393994	847037	48.754	49.772
2	16.301	19571828	890340	51.246	50.228
总计		38965822	1737378	100.000	100.000

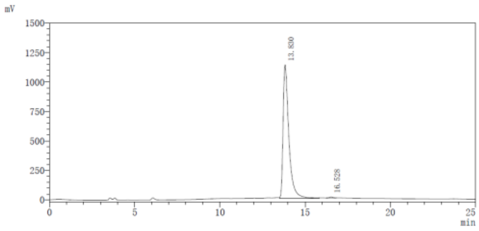

检测器A 220nm

Peak#	Time	Area	Height	Height%	Area%
1	13.830	26624561	1133562	99.281	99.482
2	16.528	138596	8206	0.719	0.518
总计		26763157	1141768	100.000	100.000

Supplementary Fig. 17 HPLC chromatogram for compound (S)-3fa

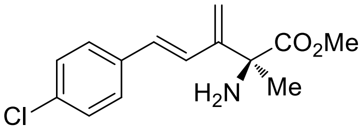

(S)-**3ga**

(*S*,*E*)-methyl 2-amino-5-(4-chlorophenyl)-2-methyl-3-methylenepent-4-enoate

Prepared according to General Procedure A using **L11-Pd** and (*S*,*S*$_p$)-**L3-Cu**, Purification by flash chromatography (PE/EA = 5:1 to 1:1) afforded the product as a pale yellow solid, 43.4 mg, 82% yield, m.p. 92–93 ℃, 99% ee, R_f = 0.4 (PE/EA, 1:1).

$[\alpha]^{25}_D$ = 12.2 (c 1.0, $CHCl_3$)

^{1}H NMR (400 MHz, $CDCl_3$) δ 7.32 (d, *J* = 8.4 Hz, 2H), 7.28 (d, *J* = 8.0 Hz, 2H), 6.78 – 6.62 (m, 2H), 5.43 (s, 1H), 5.33 (s, 1H), 3.73 (s, 3H), 1.92 (s, 2H), 1.56 (s, 3H).

^{13}C NMR (101 MHz, $CDCl_3$) δ 176.5, 148.5, 135.5, 133.4, 129.2, 128.8, 127.8, 127.4, 112.7, 60.49,

52.7, 25.8.

HRMS (ESI) calcd. for $C_{14}H_{20}NO_2^+$ $(M + H)^+$: 266.0948, Found: 266.0940

the ee value was 99%, t_r (major) = 14.062 min, t_r (minor) = 17.129 min (Chiralcel IC, λ = 220 nm, hexanes : ^{i}PrOH = 95 : 5, flow rate = 1.0 mL/min).

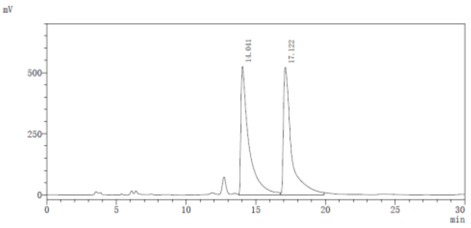

检测器A 220nm

Peak#	Time	Area	Height	Height%	Area%
1	14.041	21074407	526002	50.171	50.389
2	17.122	20749353	522425	49.829	49.611
总计		41823760	1048427	100.000	100.000

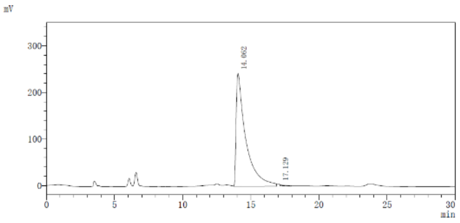

检测器A 220nm

Peak#	Time	Area	Height	Height%	Area%
1	14.062	11888383	242159	99.106	99.632
2	17.129	43940	2183	0.894	0.368
总计		11932323	244342	100.000	100.000

Supplementary Fig. 18 HPLC chromatogram for compound (S)-3ga

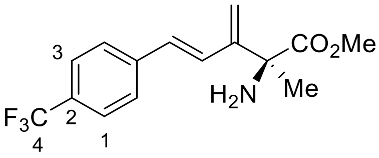

(S)-**3ha**

(*S*,*E*)-methyl 2-amino-2-methyl-3-methylene-5-(4-(trifluoromethyl)phenyl)pent-4-enoate

Prepared according to General Procedure A using **L11-Pd** and (*S*,*S*$_p$)-**L3-Cu**, Purification by flash chromatography (PE/EA = 5:1 to 1:1) afforded the product as a pale yellow oil, 42.4 mg, 71% yield , 98% ee, R_f = 0.3 (PE/EA, 1:1).

$[\alpha]^{25}_D$ = 5.9 (c 1.0, $CHCl_3$)

^{1}H NMR (400 MHz, $CDCl_3$) δ 7.56 (d, *J* = 8.1 Hz, 2H), 7.49 (d, *J* = 8.2 Hz, 2H), 6.88 – 6.73 (m, 2H), 5.48 (s, 1H), 5.39 (s, 1H), 3.73 (s, 3H), 1.90 (s, 2H), 1.57 (s, 3H).

^{13}C NMR (101 MHz, $CDCl_3$) δ 176.4, 148.4, 140.5, 129.5 (q, *J* = 32.5 Hz, C2), 129.4, 129.0, 126.7, 125.6 (q, *J* = 3.8 Hz, C1 and C3), 124.2 (q, *J* = 272.0 Hz, C4), 113.6, 60.5, 52.6, 25.9.

^{19}F NMR (376 MHz, DMSO) δ -67.2.

HRMS (ESI) calcd. for $C_{14}H_{20}NO_2^+$ $(M + H)^+$: 300.1211, Found: 300.1202

the ee value was 98%, t_r (major) = 10.155 min, t_r (minor) = 12.021 min (Chiralcel IC, λ = 220 nm, hexanes : ^{i}PrOH = 95 : 5, flow rate = 1.0 mL/min).

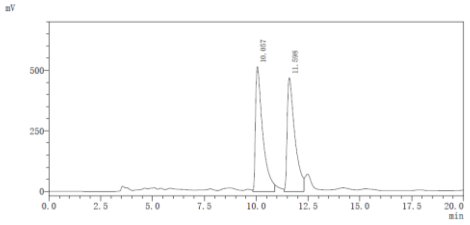

检测器A 220nm

Peak#	Time	Area	Height	Height%	Area%
1	10.057	12427609	514160	52.305	50.773
2	11.598	12049255	468848	47.695	49.227
总计		24476863	983009	100.000	100.000

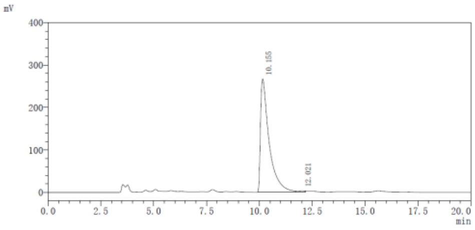

检测器A 220nm

Peak#	Time	Area	Height	Height%	Area%
1	10.155	7531989	266336	98.785	98.923
2	12.021	82012	3276	1.215	1.077
总计		7614001	269612	100.000	100.000

Supplementary Fig. 19 HPLC chromatogram for compound (S)-3ha

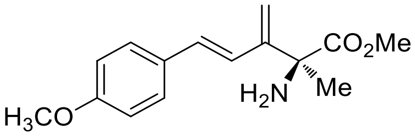

(S)-**3ia**

(*S*,*E*)-methyl 2-amino-5-(4-methoxyphenyl)-2-methyl-3-methylenepent-4-enoate

Prepared according to General Procedure A using **L11-Pd** and (*S*,*S*$_P$)-**L3-Cu**, Purification by flash chromatography (PE/EA = 5:1 to 1:2) afforded the product as a pale yellow solid, 41.5 mg, 80% yield, m.p. 74–76 ℃, >99% ee, R_f = 0.2 (PE/EA, 1:1).

$[\alpha]^{25}_D$ =10.1 (c 1.0, $CHCl_3$)

^{1}H NMR (400 MHz, $CDCl_3$) δ 7.33 (d, *J* = 8.3 Hz, 2H), 6.85 (d, *J* = 8.3 Hz, 2H), 6.63 (dd, *J* = 63.1, 16.2 Hz, 2H), 5.38 (s, 1H), 5.26 (s, 1H), 3.80 (s, 3H), 3.72 (s, 3H), 1.98 (s, 2H), 1.55 (s, 3H).

^{13}C NMR (101 MHz, $CDCl_3$) δ 176.6, 159.4, 148.9, 130.0, 129.8, 127.8, 124.6, 114.1, 111.3, 60.6, 55.3, 52.6, 25.8.

HRMS (ESI) calcd. for $C_{14}H_{20}NO_2^+$ $(M + H)^+$: 262.1443, Found: 262.1435

the ee value was > 99%, t_r (major) = 17.395 min, t_r (minor) = 23.754 min (Chiralcel IC, λ = 220 nm, hexanes : ^{i}PrOH = 90 : 10, flow rate = 1.0 mL/min).

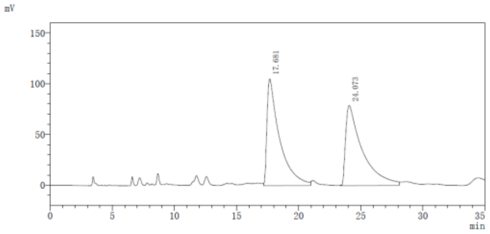

检测器A 220nm

Peak#	Time	Area	Height	Height%	Area%
1	17.681	7405533	105261	57.113	50.182
2	24.073	7351914	79040	42.887	49.818
总计		14757447	184301	100.000	100.000

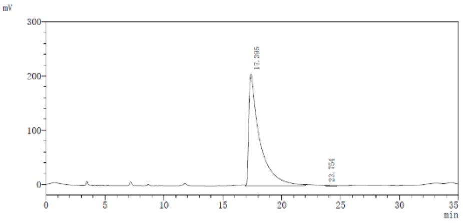

检测器A 220nm

Peak#	Time	Area	Height	Height%	Area%
1	17.395	13297141	206598	99.686	99.787
2	23.754	28429	650	0.314	0.213
总计		13325569	207248	100.000	100.000

Supplementary Fig. 20 HPLC chromatogram for compound (S)-3ia

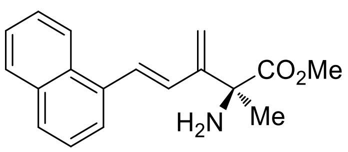

(S)-**3ja**

(*S*,*E*)-methyl 2-amino-2-methyl-3-methylene-5-(naphthalen-2-yl)pent-4-enoate

Prepared according to General Procedure A using **L11-Pd** and (*S*,*S*$_P$)-**L3-Cu**, Purification by flash chromatography (PE/EA = 5:1 to 1:1) afforded the product as a pale yellow solid, 42.6 mg, 76% yield, m.p.103–104 ℃, >99% ee, R_f = 0.3 (PE/EA, 1:1).

$[\alpha]^{25}_D$ =1.3 (c 1.0, $CHCl_3$)

^{1}H NMR (400 MHz, $CDCl_3$) δ 7.85 – 7.70 (m, 4H), 7.60 (d, *J* = 8.6 Hz, 1H), 7.50 – 7.36 (m, 2H), 6.87 (dd, *J* = 40.0, 16.2 Hz, 2H), 5.48 (s, 1H), 5.35 (s, 1H), 3.74 (s, 3H), 1.88 (s, 2H), 1.59 (s, 3H).

^{13}C NMR (101 MHz, $CDCl_3$) δ 176.7, 148.8, 134.5, 133.6, 133.1, 130.6, 128.3, 128.0, 127.7, 127.1, 126.8, 126.4, 126.0, 123.5, 112.4, 60.6, 52.7, 25.9.

HRMS (ESI) calcd. for $C_{14}H_{20}NO_2^+$ $(M + H)^+$: 282.1494, Found: 282.1486

the ee value was > 99%, t_r (major) = 19.590 min, t_r (minor) = 27.329 min (Chiralcel IC, λ = 220 nm, hexanes : ^{i}PrOH = 95 : 5, flow rate = 1.0 mL/min).

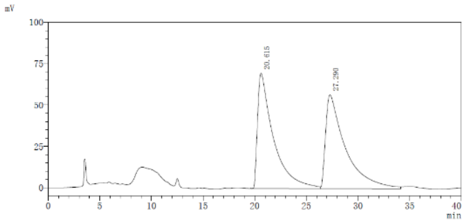

检测器A 220nm

Peak#	Time	Area	Height	Height%	Area%
1	20.615	7469831	69708	55.146	49.768
2	27.290	7539588	56698	44.854	50.232
总计		15009419	126406	100.000	100.000

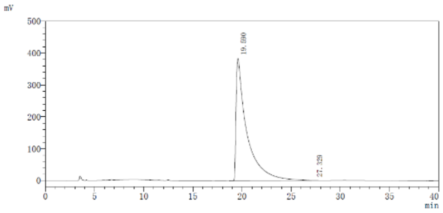

检测器A 220nm

Peak#	Time	Area	Height	Height%	Area%
1	19.590	29337756	384109	99.853	99.949
2	27.329	14929	566	0.147	0.051
总计		29352685	384675	100.000	100.000

Supplementary Fig. 21 HPLC chromatogram for compound (S)-3ja

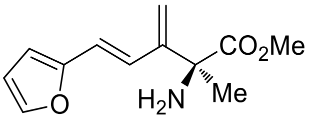

(S)-**3ka**

(*S*,*E*)-methyl 2-amino-5-(furan-2-yl)-2-methyl-3-methylenepent-4-enoate

Prepared according to General Procedure A using **L11-Pd** and (S,S_p)-**L3-Cu**, Purification by flash chromatography (PE/EA = 5:1 to 1:1) afforded the product as a pale yellow oil, 31.2 mg, 70% yield, 97% ee, R_f = 0.3 (PE/EA, 1:1).

$[\alpha]^{25}_D$ = 4.9 (c 0.8, $CHCl_3$)

^{1}H NMR (400 MHz, $CDCl_3$) δ 7.36 (s, 1H), 6.59 (q, *J* = 16.2 Hz, 2H), 6.40 – 6.37 (m, 1H), 6.28 (d, *J* = 2.8 Hz, 1H), 5.40 (s, 1H), 5.31 (s, 1H), 3.73 (s, 3H), 2.02 (s, 2H), 1.55 (s, 3H).

^{13}C NMR (101 MHz, $CDCl_3$) δ 176.6, 152.7, 148.4, 142.3, 125.2, 118.3, 112.4, 111.6, 109.0, 60.4, 52.7, 25.8.

HRMS (ESI) calcd. for $C_{14}H_{20}NO_2^+$ $(M + H)^+$: 222.1130, Found: 222.1124

the ee value was 97%, t_r (major) = 31.875 min, t_r (minor) = 35.959 min (Chiralcel IE, λ = 220 nm, hexanes : ^{i}PrOH = 98 : 2, flow rate = 1.0 mL/min).

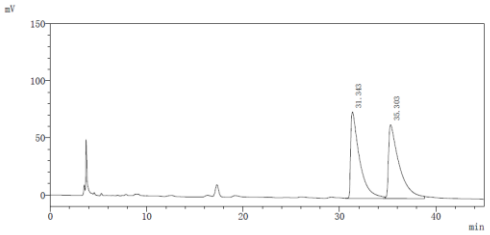

检测器A 220nm

Peak#	Time	Area	Height	Height%	Area%
1	31.343	4843930	75673	53.946	49.375
2	35.303	4966478	64603	46.054	50.625
总计		9810408	140276	100.000	100.000

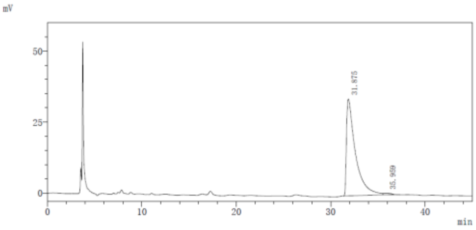

检测器A 220nm

Peak#	Time	Area	Height	Height%	Area%
1	31.875	2244726	34090	98.252	98.608
2	35.959	31678	606	1.748	1.392
总计		2276404	34696	100.000	100.000

Supplementary Fig. 22 HPLC chromatogram for compound (S)-3ka

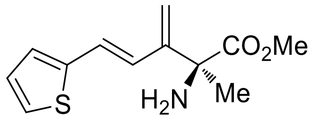

(S)-**3la**

(*S*,*E*)-methyl 2-amino-2-methyl-3-methylene-5-(thiophen-2-yl)pent-4-enoate

Prepared according to General Procedure A using **L11-Pd** and (*S*,*S_p*)-**L3-Cu**, Purification by flash chromatography (PE/EA = 5:1 to 1:1) afforded the product as a pale yellow oil, 33.4 mg, 70% yield, >99% ee, R_f = 0.3 (PE/EA, 1:1).

$[\alpha]^{25}_D$ = -1.1 (c 0.8, $CHCl_3$)

^{1}H NMR (400 MHz, $CDCl_3$) δ 7.17 (d, *J* = 4.7 Hz, 1H), 7.02 – 6.94 (m, 2H), 6.87 (d, *J* = 16.1 Hz, 1H), 6.52 (d, *J* = 16.1 Hz, 1H), 5.39 (s, 1H), 5.31 (s, 1H), 3.73 (s, 3H), 1.97 (s, 2H), 1.55 (s, 3H).

^{13}C NMR (101 MHz, $CDCl_3$) δ 177.4, 148.3, 142.5, 127.5, 126.5, 126.4, 124.6, 123.6, 112.6, 60.4, 52.7, 25.9.

HRMS (ESI) calcd. for $C_{14}H_{20}NO_2^+$ $(M + H)^+$: 238.0902, Found: 238.0895

the ee value was > 99%, t_r (major) = 18.849 min, t_r (minor) = 23.771 min (Chiralcel IC, λ = 220 nm, hexanes : ^{i}PrOH = 95 : 5, flow rate = 1.0 mL/min).

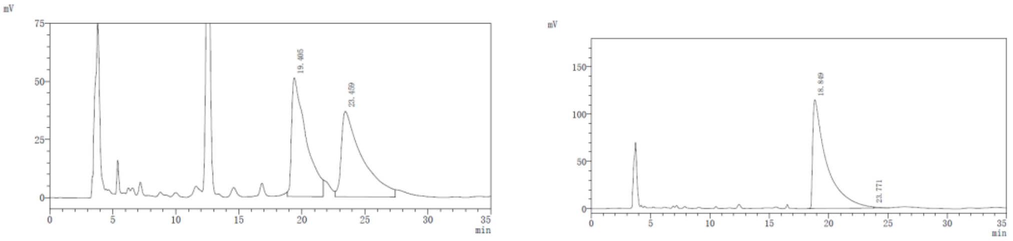

检测器A 220nm

Peak#	Time	Area	Height	Height%	Area%
1	19.405	4064755	51055	58.160	49.017
2	23.459	4227703	36728	41.840	50.983
总计		8292458	87783	100.000	100.000

检测器A 220nm

Peak#	Time	Area	Height	Height%	Area%
1	18.849	9346201	114958	99.104	99.719
2	23.771	26372	1039	0.896	0.281
总计		9372573	115997	100.000	100.000

Supplementary Fig. 23 HPLC chromatogram for compound (S)-3la

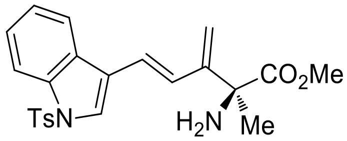

(S)-**3ma**

(*S*,*E*)-methyl 2-amino-2-methyl-3-methylene-5-(1-tosyl-1H-indol-3-yl)pent-4-enoate

Prepared according to General Procedure A using **L11-Pd** and (*S*,*S_p*)-**L3-Cu**, Purification by flash chromatography (PE/EA = 5:1 to 1:2) afforded the product as a pale yellow oil, 57.3 mg, 68 % yield, 99% ee, R_f = 0.2 (PE/EA, 1:1).

$[\alpha]^{25}_D$ = -8.4 (c 1.0, $CHCl_3$)

^{1}H NMR (400 MHz, $CDCl_3$) δ 7.99 (d, *J* = 8.2 Hz, 1H), 7.77 (d, *J* = 8.2 Hz, 2H), 7.70 (d, *J* = 7.8 Hz, 1H), 7.63 (s, 1H), 7.33 (t, *J* = 7.6 Hz, 1H), 7.29 – 7.24 (m, 1H), 7.21 (d, *J* = 8.1 Hz, 2H), 6.80 (q, *J* = 16.4 Hz, 2H), 5.43 (s, 1H), 5.32 (s, 1H), 3.74 (s, 3H), 2.33 (s, 3H), 1.58 (s, 3H).

^{13}C NMR (101 MHz, $CDCl_3$) δ 176.6, 148.9, 145.1, 135.5, 135.1, 129.9, 129.0, 127.8, 126.9, 125.0, 124.0, 123.6, 121.0, 120.5, 120.3, 113.8, 112.0, 60.5, 52.7, 25.9, 21.6.

HRMS (ESI) calcd. for $C_{14}H_{20}NO_2^+$ $(M + H)^+$: 425.1535, Found: 425.1527

the ee value was 99%, t_r (major) = 41.029 min, t_r (minor) = 49.988 min (Chiralcel IC, λ = 220 nm, hexanes : ^{i}PrOH = 80 : 20, flow rate = 1.0 mL/min).

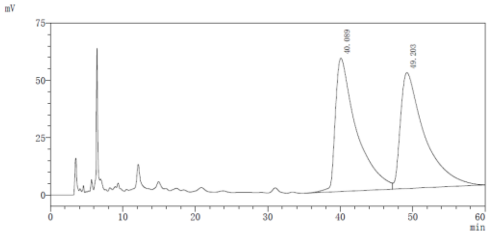

检测器A 220nm

Peak#	Time	Area	Height	Height%	Area%
1	40.089	11785379	58086	53.484	50.262
2	49.203	11662422	50519	46.516	49.738
总计		23447801	108606	100.000	100.000

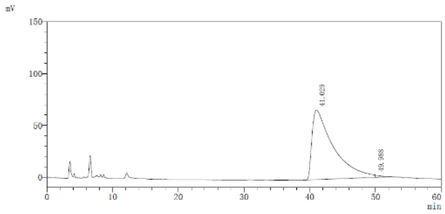

检测器A 220nm

Peak#	Time	Area	Height	Height%	Area%
1	41.029	15126717	67092	97.046	99.260
2	49.988	112791	2042	2.954	0.740
总计		15239508	69134	100.000	100.000

Supplementary Fig. 24 HPLC chromatogram for compound (S)-3ma

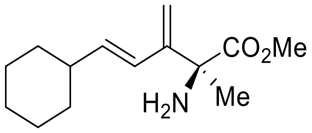

(S)-**3na**

(*S*,*E*)-methyl 2-amino-5-cyclohexyl-2-methyl-3-methylenepent-4-enoate

Prepared according to General Procedure B using **L11-Pd** and (*S*,*S*$_p$)-**L3-Cu**, Purification by flash chromatography (PE/EA = 5:1 to 1:2) afforded the product as a pale yellow oil, 29.8 mg, 63% yield, 94% ee, R_f = 0.2 (PE/EA, 1:1).

$[\alpha]^{25}_D$ = 7.4 (c 0.8, $CHCl_3$)

^{1}H NMR (400 MHz, $CDCl_3$) δ 5.89 (d, *J* = 15.9 Hz, 1H), 5.77 (dd, *J* = 15.9, 6.8 Hz, 1H), 5.18 (s, 1H), 5.14 (s, 1H), 3.70 (s, 3H), 1.98 (dt, *J* = 10.7, 9.1 Hz, 1H), 1.79 (s, 2H), 1.72 – 1.63 (m, 5H), 1.48(s, 3H), 1.33 – 1.19 (m, 3H), 1.13 – 1.04 (m, 2H).

^{13}C NMR (101 MHz, $CDCl_3$) δ 176.8, 149.1, 138.7, 125.4, 110.5, 60.4, 52.4, 41.1, 32.7, 32.8, 26.1, 25.9, 25.5.

HRMS (ESI) calcd. for $C_{14}H_{20}NO_2^+$ $(M + H)^+$: 238.1807, Found: 238.1799

the ee value was 94%, t_r (major) = 14.269 min, t_r (minor) = 16.302 min (Chiralcel IF, λ = 220 nm, hexanes : ^{i}PrOH = 99 : 1, flow rate = 1.0 mL/min).

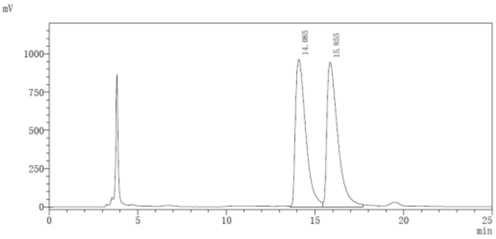

检测器A 220nm

Peak#	Time	Area	Height	Height%	Area%
1	14.085	38785542	963812	50.487	49.213
2	15.855	40025572	945221	49.513	50.787
总计		78811114	1909032	100.000	100.000

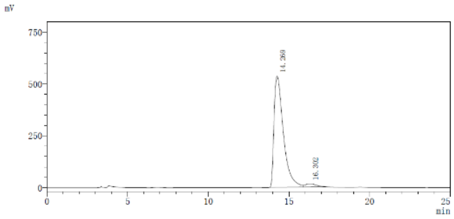

检测器A 220nm

Peak#	Time	Area	Height	Height%	Area%
1	14.269	21528671	535428	97.167	96.765
2	16.302	719812	15611	2.833	3.235
总计		22248483	551038	100.000	100.000

Supplementary Fig. 25 HPLC chromatogram for compound (S)-3na

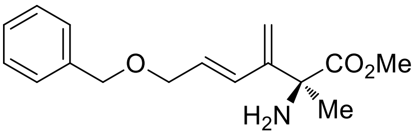

(S)-**3oa**

(*S*,*E*)-methyl 2-amino-6-(benzyloxy)-2-methyl-3-methylenehex-4-enoate

Prepared according to General Procedure A using **L11-Pd** and (*S*,*S*$_p$)-**L3-Cu**, Purification by flash chromatography (PE/EA = 5:1 to 1:2) afforded the product as a pale yellow oil, 33.1 mg, 60% yield, 99% ee, R_f = 0.2 (PE/EA, 1:1).

$[\alpha]^{25}_D$ = 7.7 (c 0.5, $CHCl_3$)

^{1}H NMR (400 MHz, $CDCl_3$) δ 7.61 – 7.07 (m, 5H), 6.21 (d, *J* = 16.0 Hz, 1H), 5.96 (dt, *J* = 15.9, 5.9 Hz, 1H), 5.27 (d, *J* = 21.2 Hz, 2H), 4.51 (s, 2H), 4.06 (dd, *J* = 5.9, 1.2 Hz, 2H), 3.70 (s, 3H), 1.83 (s, 2H), 1.50 (s, 3H).

^{13}C NMR (101 MHz, $CDCl_3$) δ 176.5, 148.1, 138.2, 130.5, 128.4, 128.1, 127.8, 127.7, 112.6, 72.1, 70.6, 60.3, 52.6, 25.6.

HRMS (ESI) calcd. for $C_{14}H_{20}NO_2^+$ $(M + H)^+$: 276.1600, Found: 276.1596

the ee value was 99%, t_r (major) = 10.914 min, t_r (minor) = 11.846 min (Chiralcel IA, λ = 220 nm, hexanes : ^{i}PrOH = 95 : 5, flow rate = 1.0 mL/min).

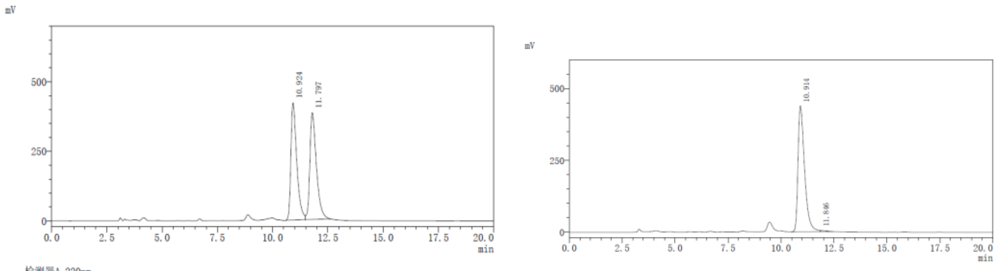

检测器A 220nm

Peak#	Time	Area	Height	Height%	Area%
1	10.924	7771319	420616	52.304	49.719
2	11.797	7859009	383558	47.696	50.281
总计		15630329	804174	100.000	100.000

检测器A 220nm

Peak#	Time	Area	Height	Height%	Area%
1	10.914	9468675	438482	99.025	99.239
2	11.846	72602	4319	0.975	0.761
总计		9541277	442801	100.000	100.000

Supplementary Fig. 26 HPLC chromatogram for compound (S)-3oa

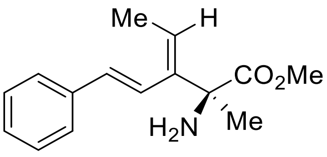

(S)-**3pa**

(*S*,*E*)-methyl 2-amino-2-methyl-3-((*E*)-styryl)pent-3-enoate

Prepared according to General Procedure C using **L11-Pd** (0.08 equiv.)and (*S*,*S*$_p$)-**L3-Cu**, Purification by flash chromatography (PE/EA = 5:1 to 1:2) afforded the product as a pale yellow oil, 24.5 mg, 50% yield, >99% ee, R_f = 0.2 (PE/EA, 1:1).

$[\alpha]^{25}_D$ = -58.2 (c 0.5, $CHCl_3$)

^{1}H NMR (400 MHz, $CDCl_3$) δ 7.40 (d, *J* = 7.4 Hz, 2H), 7.32 (t, *J* = 7.5 Hz, 2H), 7.23 (t, *J* = 7.3 Hz, 1H), 6.75 (d, *J* = 16.7 Hz, 1H), 6.51 (d, *J* = 16.7 Hz, 1H), 5.94 (q, *J* = 7.0 Hz, 1H), 3.70 (s, 3H), 1.89 – 1.87 (m, 5H), 1.52 (s, 3H).

^{13}C NMR (101 MHz, CDCl$_3$) δ 177.5, 139.8, 137.4, 131.9, 128.6, 127.6, 126.3, 123.7, 123.5, 60.9, 52.5, 26.0, 14.6.
HRMS (ESI) calcd. for $C_{14}H_{20}NO_2^+$ (M + H)$^+$: 246.1494, Found: 246.1486

the ee value was >99%, t_r (major) = 39.345 min, t_r (minor) = 34.958 min (Chiralcel IC, λ = 220 nm, hexanes : ^{i}PrOH = 98 : 2, flow rate = 1.0 mL/min).

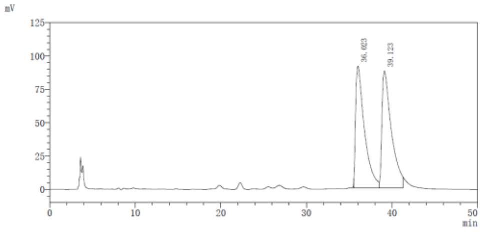

检测器A 220nm

Peak#	Time	Area	Height	Height%	Area%
1	36.023	6538265	91273	50.996	49.262
2	39.123	6734265	87706	49.004	50.738
总计		13272530	178979	100.000	100.000

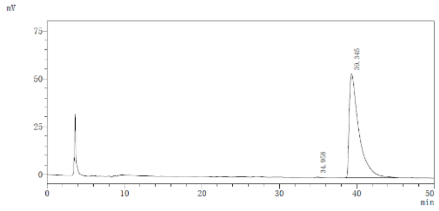

检测器A 220nm

Peak#	Time	Area	Height	Height%	Area%
1	34.958	7825	210	0.387	0.167
2	39.345	4671627	54105	99.613	99.833
总计		4679453	54316	100.000	100.000

Supplementary Fig. 27 HPLC chromatogram for compound (S)-3pa

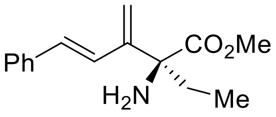

(S)-**3ab**

(*S*,*E*)-methyl 2-amino-2-ethyl-3-methylene-5-phenylpent-4-enoate
Prepared according to General Procedure B using **L11-Pd** and (*S*,*S*$_p$)-**L3-Cu**, Purification by flash chromatography (PE/EA = 5:1 to 1:1) afforded the product as a pale yellow oil, 35 mg, 71% yield, 92% ee, R_f = 0.4 (PE/EA, 1:1).
$[\alpha]^{25}_D$ = 6.9 (c 0.8, CHCl$_3$)
^{1}H NMR (400 MHz, CDCl$_3$) δ 7.40 (d, *J* = 7.3 Hz, 2H), 7.32 (t, *J* = 7.5 Hz, 2H), 7.26 – 7.24 (m, 1H), 6.74 (q, *J* = 16.1 Hz, 2H), 5.48 (s, 1H), 5.29 (s, 1H), 3.73 (s, 3H), 2.05 – 1.88 (m, 4H), 0.89 (t, *J* = 7.4 Hz, 3H).
^{13}C NMR (101 MHz, CDCl$_3$) δ 175.9, 148.0, 137.0, 130.5, 128.6, 127.8, 126.9, 126.6, 112.5, 64.1, 52.5, 30.2, 8.0.
HRMS (ESI) calcd. for $C_{14}H_{20}NO_2^+$ (M + H)$^+$: 246.1494, Found: 246.1486

the ee value was 92%, t_r (major) = 12.140 min, t_r (minor) = 13.877 min (Chiralcel IC, λ = 220 nm, hexanes : ^{i}PrOH = 95 : 5, flow rate = 1.0 mL/min).

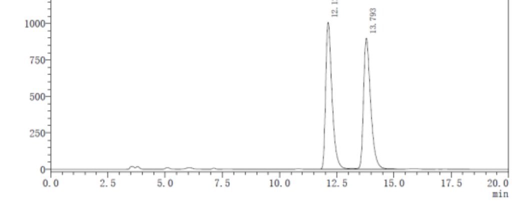

检测器A 220nm

Peak#	Time	Area	Height	Height%	Area%
1	12.121	19563151	1008778	52.839	49.923
2	13.793	19623406	900381	47.161	50.077
总计		39186558	1909159	100.000	100.000

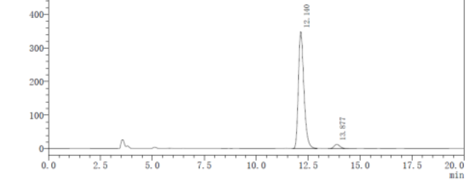

检测器A 220nm

Peak#	Time	Area	Height	Height%	Area%
1	12.140	6503594	348988	96.452	96.113
2	13.877	263047	12839	3.548	3.887
总计		6766641	361828	100.000	100.000

Supplementary Fig. 28 HPLC chromatogram for compound (S)-3ab

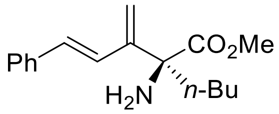

(S)-**3ac**

(*S*,*E*)-methyl 2-amino-2-(4-phenylbuta-1,3-dien-2-yl)hexanoate

Prepared according to General Procedure B using **L11-Pd** and (*S*,*S$_p$*)-**L3-Cu**, Purification by flash chromatography (PE/EA = 5:1 to 1:1) afforded the product as a pale yellow oil, 38.9 mg, 69% yield, dr, 96% ee, R_f = 0.4 (PE/EA, 1:1).

$[\alpha]^{25}_{D}$ = 6.2 (c 1.0, $CHCl_3$)

^{1}H NMR (400 MHz, $CDCl_3$) δ 7.40 (d, *J* = 7.3 Hz, 2H), 7.32 (t, *J* = 7.5 Hz, 2H), 7.24 (dd, *J* = 12.1, 4.9 Hz, 1H), 6.74 (q, *J* = 16.1 Hz, 2H), 5.46 (s, 1H), 5.29 (s, 1H), 3.73 (s, 3H), 1.99 – 1.81(m, 4H), 1.39 – 1.26 (m, 4H), 0.90 (t, *J* = 7.0 Hz, 3H).

^{13}C NMR (101 MHz, $CDCl_3$) δ 176.0, 148.3, 137.1, 130.5, 128.1, 127.8, 126.9, 126.6, 112.3, 63.8, 52.5, 37.2, 25.9, 23.0, 14.0.

HRMS (ESI) calcd. for $C_{14}H_{20}NO_2^+$ $(M + H)^+$: 274.1807, Found: 274.1801

the ee value was 96%, t_r (major) = 18.100 min, t_r (minor) = 21.493 min (Chiralcel IC, λ = 220 nm, hexanes : ^{i}PrOH = 95 : 5, flow rate = 1.0 mL/min).

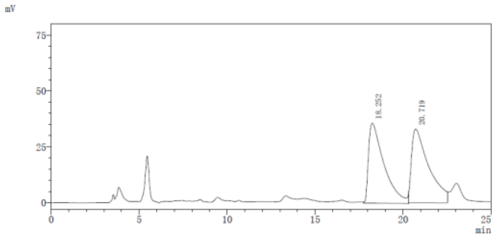

检测器A 220nm

Peak#	Time	Area	Height	Height%	Area%
1	18.252	2202124	35781	52.104	49.747
2	20.719	2224564	32891	47.896	50.253
总计		4426688	68672	100.000	100.000

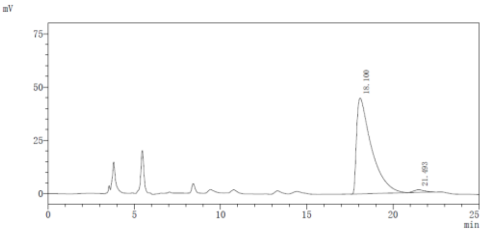

检测器A 220nm

Peak#	Time	Area	Height	Height%	Area%
1	18.100	2682750	44908	97.561	98.163
2	21.493	50203	1123	2.439	1.837
总计		2732953	46031	100.000	100.000

Supplementary Fig. 29 HPLC chromatogram for compound (S)-3ac

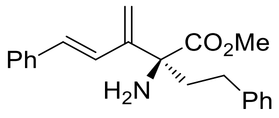

(*S*)-**3ad**

(*S*,*E*)-methyl 2-amino-3-methylene-2-phenethyl-5-phenylpent-4-enoate

Prepared according to General Procedure B using **L11-Pd** and (*S*,*S$_p$*)-**L3-Cu**, Purification by flash chromatography (PE/EA = 5:1 to 1:1) afforded the product as a pale yellow oil, 37.4 mg, 58% yield, 92% ee, R_f = 0.4 (PE/EA, 1:1).

$[\alpha]^{25}_{D}$ = 8.2 (c 1.0, $CHCl_3$)

^{1}H NMR (400 MHz, $CDCl_3$) δ 7.40 (d, *J* = 7.4 Hz, 2H), 7.32 (t, *J* = 7.5 Hz, 2H), 7.29 – 7.23 (m, 3H), 7.22 – 7.15 (m, 3H), 6.77 (q, *J* = 16.1 Hz, 2H), 5.50 (s, 1H), 5.33 (s, 1H), 3.74 (s, 3H), 2.69 (td, *J* = 12.6, 5.2 Hz, 1H), 2.60 – 2.51 (m, 1H), 2.22 (tdd, *J* = 25.5, 13.6, 5.1 Hz, 2H), 1.89 (s, 2H).

^{13}C NMR (101 MHz, $CDCl_3$) δ 175.6, 147.9, 141.8, 137.0, 130.8, 128.6, 128.5, 127.9, 126.7, 126.6, 126.0, 112.5, 63.8, 52.6, 39.4, 30.3.

HRMS (ESI) calcd. for $C_{14}H_{20}NO_2^+$ $(M + H)^+$: 322.1807, Found: 322.1799

the ee value was 92%, t_r (major) = 19.189 min, t_r (minor) = 15.555 min (Chiralcel IC, λ = 220 nm, hexanes : ^{i}PrOH = 95 : 5, flow rate = 1.0 mL/min).

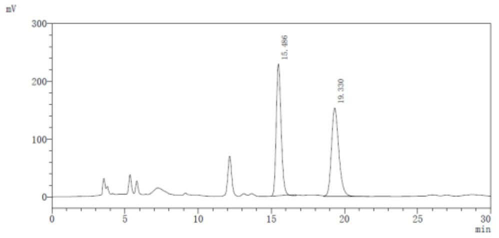

检测器A 220nm

Peak#	Time	Area	Height	Height%	Area%
1	15.486	5244896	228071	59.802	49.397
2	19.330	5373037	153307	40.198	50.603
总计		10617933	381378	100.000	100.000

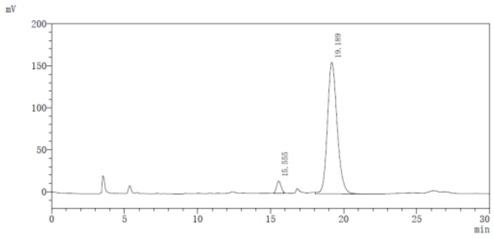

检测器A 220nm

Peak#	Time	Area	Height	Height%	Area%
1	15.555	314020	14736	8.591	4.155
2	19.189	7243309	156782	91.409	95.845
总计		7557329	171517	100.000	100.000

Supplementary Fig. 30 HPLC chromatogram for compound (S)-3ad

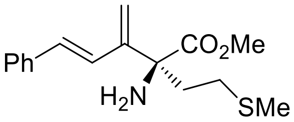

(*S*)-**3ae**

(*S*,*E*)-methyl 2-amino-3-methylene-2-(2-(methylthio)ethyl)-5-phenylpent-4-enoate

Prepared according to General Procedure B using **L11-Pd** and (*S*,*S*$_p$)-**L3-Cu**, Purification by flash chromatography (PE/EA = 5:1 to 1:1) afforded the product as a pale yellow oil, 39 mg, 67% yield, 95% ee, R_f = 0.3 (PE/EA, 1:1).

$[\alpha]^{25}_D$ = -2.1 (c 1.0, $CHCl_3$)

^{1}H NMR (400 MHz, $CDCl_3$) δ 7.40 (d, *J* = 7.2 Hz, 2H), 7.32 (t, *J* = 7.2 Hz, 2H), 7.28 – 7.22 (m, 1H), 6.72 (dd, *J* = 43.4, 16.1 Hz, 2H), 5.49 (s, 1H), 5.32 (s, 1H), 3.74 (s, 3H), 2.59 – 2.41 (m, 2H), 2.25 – 2.16 (m, 2H), 2.09 (s, 3H), 1.86 (s, 2H).

^{13}C NMR (101 MHz, $CDCl_3$) δ 175.3, 147.4, 136.9, 130.9, 128.7, 127.9, 126.6, 126.3, 112.6, 63.61, 52.7, 36.8, 28.7, 15.6.

HRMS (ESI) calcd. for $C_{14}H_{20}NO_2^+$ $(M + H)^+$: 292.1371, Found: 292.1361

the ee value was 95%, t_r (major) = 27.541 min, t_r (minor) = 37.019 min (Chiralcel IE, λ = 220 nm, hexanes : ^{i}PrOH = 98 : 2, flow rate = 1.0 mL/min).

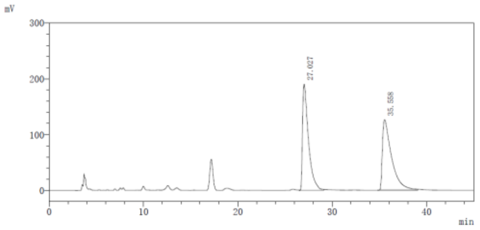

检测器A 220nm

Peak#	Time	Area	Height	Height%	Area%
1	27.027	8295036	190174	60.038	49.088
2	35.558	8603256	126584	39.962	50.912
总计		16898291	316758	100.000	100.000

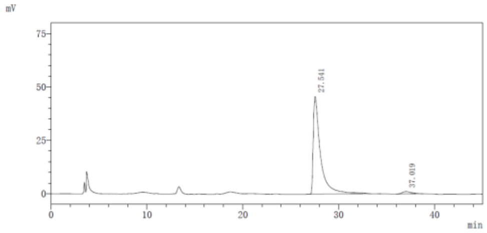

检测器A 220nm

Peak#	Time	Area	Height	Height%	Area%
1	27.541	2391155	45663	97.824	97.294
2	37.019	66502	1016	2.176	2.706
总计		2457657	46679	100.000	100.000

Supplementary Fig. 31 HPLC chromatogram for compound (S)-3ae

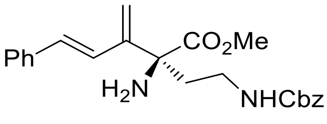

(*S*)-**3af**

(*S*,*E*)-methyl2-amino-2-(2-(((benzyloxy)carbonyl)amino)ethyl)-3-methylene-5-phenylpent-4-enoate

Prepared according to General Procedure B using **L11-Pd** and (*S*,*S*$_P$)-**L3-Cu**, Purification by flash chromatography (PE/EA = 5:1 to 1:3) afforded the product as a pale yellow oil, 35.6 mg, 45 % yield, 88% ee, R_f = 0.3 (PE/EA, 1:2).

$[\alpha]^{25}_D$ = 3.4 (c 1.0, $CHCl_3$)

^{1}H NMR (400 MHz, $CDCl_3$) δ 7.38 (d, *J* = 7.4 Hz, 2H), 7.34 – 7.29 (m, 7H), 7.26– 7.22 (m, 1H), 6.69 (dd, *J* = 52.8, 16.1 Hz, 2H), 5.61 (s, 1H), 5.48 (s, 1H), 5.33 (s, 1H), 5.07 (s, 2H), 3.70 (s, 3H), 3.30 (d, *J* = 5.7 Hz, 2H), 2.20 – 2.07 (m, 2H), 1.91 (s, 2H).

^{13}C NMR (101 MHz, $CDCl_3$) δ 175.3, 156.3, 147.2, 136.8, 136.7, 131.1, 128.7, 128.5, 128.1, 128.0, 128.0, 126.7, 126.1, 112.6, 66.6, 63.3, 52.7, 37.1, 36.2.

HRMS (ESI) calcd. for $C_{14}H_{20}NO_2^+$ $(M + H)^+$: 395.1971, Found: 395.1963

the ee value was 88%, t_r (major) = 36.764 min, t_r (minor) = 33.605 min (Chiralcel IC, λ = 220 nm, hexanes : ^{i}PrOH = 85 : 15, flow rate = 1.0 mL/min).

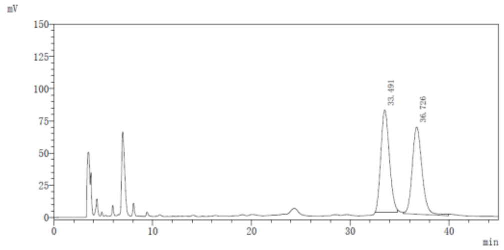

检测器A 220nm

Peak#	Time	Area	Height	Height%	Area%
1	33.491	4793542	79621	54.053	50.707
2	36.726	4659838	67681	45.947	49.293
总计		9453381	147302	100.000	100.000

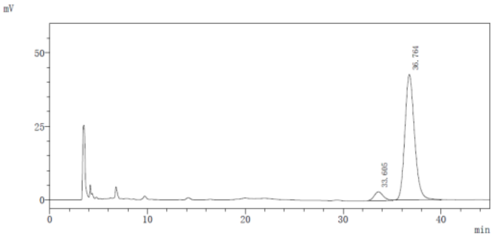

检测器A 220nm

Peak#	Time	Area	Height	Height%	Area%
1	33.605	189971	2966	6.504	6.092
2	36.764	2928504	42640	93.496	93.908
总计		3118475	45606	100.000	100.000

Supplementary Fig. 32 HPLC chromatogram for compound (S)-3af

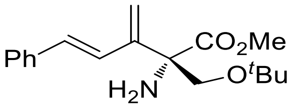

(*S*)-**3ag**

(*S*,*E*)-methyl 2-amino-2-(tert-butoxymethyl)-3-methylene-5-phenylpent-4-enoate

Prepared according to General Procedure B using **L11-Pd** and (*S*,*S*$_P$)-**L3-Cu**, Purification by flash chromatography (PE/EA = 5:1 to 1:1) afforded the product as a pale yellow oil, 29 mg, 48% yield , 96% ee, R_f = 0.3 (PE/EA, 1:1).

$[\alpha]^{25}_D$ = -11.1 (c 0.5, $CHCl_3$)

^{1}H NMR (400 MHz, $CDCl_3$) δ 7.41 (d, *J* = 7.6 Hz, 2H), 7.32 (t, *J* = 7.4 Hz, 2H), 7.23 (d, *J* = 6.9 Hz, 1H), 6.78 (t, *J* = 9.1 Hz, 2H), 5.46 (s, 1H), 5.19 (s, 1H), 3.90 (d, *J* = 8.0 Hz, 1H), 3.74 (s, 3H), 3.47 (d, *J* = 7.9 Hz, 1H), 2.24 (s, 2H), 1.17 (s, 9H).

^{13}C NMR (101 MHz, $CDCl_3$) δ 175.1, 146.2, 137.1, 130.8, 128.6, 127.8, 126.7, 126.5, 112.6, 73.3, 66.3, 65.0, 52.4, 27.4.

HRMS (ESI) calcd. for $C_{14}H_{20}NO_2^+$ $(M + H)^+$: 304.1913, Found: 304.1906

the ee value was 96%, t_r (major) = 17.026 min, t_r (minor) = 20.895 min (Chiralcel IC, λ = 220 nm, hexanes : ^{i}PrOH = 98 : 2, flow rate = 1.0 mL/min).

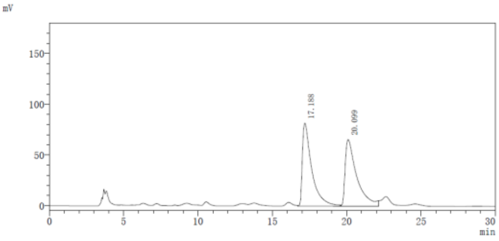

检测器A 220nm

Peak#	Time	Area	Height	Height%	Area%
1	17.188	3593927	81857	55.419	50.144
2	20.099	3573320	65848	44.581	49.856
总计		7167247	147706	100.000	100.000

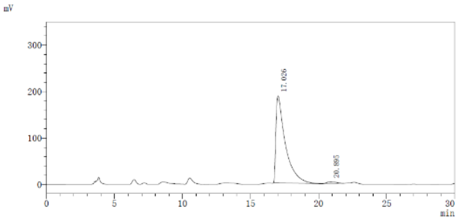

检测器A 220nm

Peak#	Time	Area	Height	Height%	Area%
1	17.026	9018809	187511	98.097	98.178
2	20.895	167353	3637	1.903	1.822
总计		9186162	191148	100.000	100.000

Supplementary Fig. 33 HPLC chromatogram for compound (S)-3ag

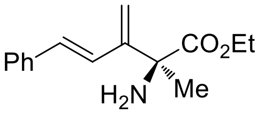

(S)-**3ah**

(*S*,*E*)-ethyl 2-amino-2-methyl-3-methylene-5-phenylpent-4-enoate

Prepared according to General Procedure B using **L11-Pd** and (*S*,*S*$_p$)-**L3-Cu**, Purification by flash chromatography (PE/EA = 5:1 to 1:1) afforded the product as a pale yellow oil, 32.9 mg, 65% yield, >99% ee, R_f = 0.4 (PE/EA, 1:1).

$[\alpha]^{25}_D$ = 5.2 (c 1.0, $CHCl_3$)

^{1}H NMR (400 MHz, $CDCl_3$) δ 7.39 (d, *J* = 7.8 Hz, 2H), 7.31 (t, *J* = 7.5 Hz, 2H), 7.26 – 7.21 (m, 1H), 6.79 – 6.67 (m, 2H), 5.42 (s, 1H), 5.32 (s, 1H), 4.19 (q, *J* = 7.1 Hz, 2H), 1.86 (s, 2H), 1.55 (s, 3H), 1.24 (t, *J* = 7.1 Hz, 3H).

^{13}C NMR (101 MHz, $CDCl_3$) δ 176.1, 148.8, 137.0, 130.4, 128.6, 127.8, 126.9, 126.6, 112.3, 61.4, 60.4, 25.8, 14.2.

HRMS (ESI) calcd. for $C_{14}H_{20}NO_2^+$ $(M + H)^+$: 246.1494, Found: 246.1486

the ee value was > 99%, t_r (major) = 14.083 min, t_r (minor) = 18.497 min (Chiralcel IC, λ = 220 nm, hexanes : ^{i}PrOH = 95 : 5, flow rate = 1.0 mL/min).

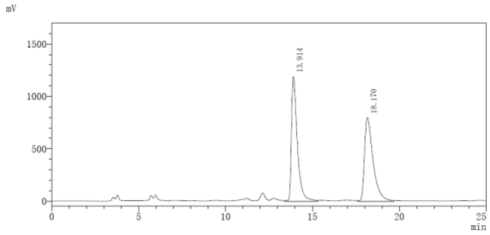

检测器A 220nm

Peak#	Time	Area	Height	Height%	Area%
1	13.914	27688237	1188706	59.859	50.413
2	18.170	27234791	797124	40.141	49.587
总计		54923029	1985829	100.000	100.000

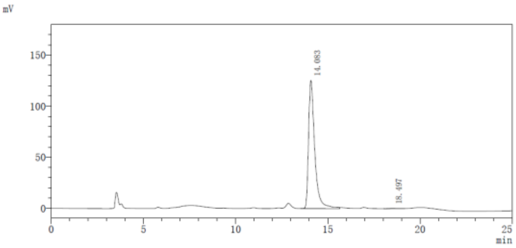

检测器A 220nm

Peak#	Time	Area	Height	Height%	Area%
1	14.083	2946543	125591	99.864	99.844
2	18.497	4608	171	0.136	0.156
总计		2951151	125762	100.000	100.000

Supplementary Fig. 34 HPLC chromatogram for compound (S)-3ah

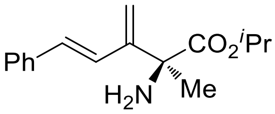

(S)-**3ai**

(*S*,*E*)-isopropyl 2-amino-2-methyl-3-methylene-5-phenylpent-4-enoate

Prepared according to General Procedure B using **L11-Pd** and (*S*,*S*$_p$)-**L3-Cu**, Purification by flash chromatography (PE/EA = 5:1 to 1:1) afforded the product as a pale yellow oil, 40.2 mg, 77% yield, 97% ee, R_f = 0.5 (PE/EA, 1:1).

$[\alpha]^{25}_{D}$ = 4.3 (c 1.0, $CHCl_3$)

^{1}H NMR (400 MHz, $CDCl_3$) δ 7.39 (d, *J* = 7.3 Hz, 2H), 7.31 (t, *J* = 7.5 Hz, 2H), 7.23 (t, *J* = 7.2 Hz, 1H), 6.77 – 6.65 (m, 2H), 5.41 (s, 1H), 5.32 (s, 1H), 5.10 – 4.99 (m, 1H), 1.87 (s, 2H), 1.54 (s, 3H), 1.21 (t, *J* = 5.9 Hz, 6H).

^{13}C NMR (101 MHz, $CDCl_3$) δ 175.6, 148.9, 137.1, 130.3, 128.6, 127.7, 127.1, 126.5, 112.3, 68.8, 60.4, 25.7, 21.6, 21.6.

HRMS (ESI) calcd. for $C_{14}H_{20}NO_2^+$ $(M + H)^+$: 260.1651, Found: 260.1643

the ee value was 97%, t_r (major) = 11.964 min, t_r (minor) = 15.942 min (Chiralcel IC, λ = 220 nm, hexanes : ^{i}PrOH = 95 : 5, flow rate = 1.0 mL/min).

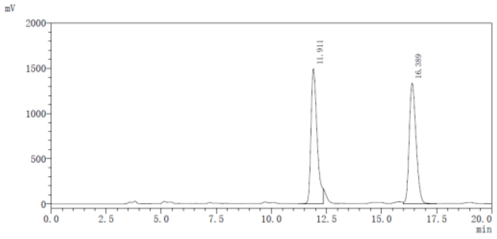

检测器A 220nm

Peak#	Time	Area	Height	Height%	Area%
1	11.911	28645312	1495771	52.795	49.807
2	16.389	28867784	1337423	47.205	50.193
总计		57513097	2833194	100.000	100.000

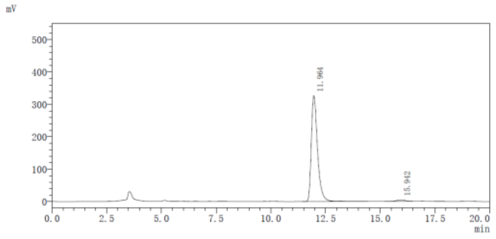

检测器A 220nm

Peak#	Time	Area	Height	Height%	Area%
1	11.964	6397082	327097	98.773	98.334
2	15.942	108394	4062	1.227	1.666
总计		6505476	331159	100.000	100.000

Supplementary Fig. 35 HPLC chromatogram for compound (S)-3ai

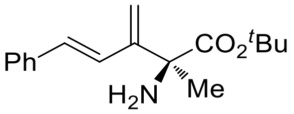

(S)-**3aj**

(*S*,*E*)-tert-butyl 2-amino-2-methyl-3-methylene-5-phenylpent-4-enoate

Prepared according to General Procedure B using **L11-Pd** and (*S*,*S*$_p$)-**L3-Cu**, Purification by flash chromatography (PE/EA = 10:1 to 5:1) afforded the product as a pale yellow oil, 46.3 mg, 85% yield, 91% ee, R_f = 0.3 (PE/EA, 5:1).

$[\alpha]^{25}_{D}$ = 8.0 (c 1.0, $CHCl_3$)

^{1}H NMR (400 MHz, $CDCl_3$) δ 7.39 (d, *J* = 7.6 Hz, 2H), 7.31 (t, *J* = 7.1 Hz, 2H), 7.23 (t, *J* = 7.0 Hz, 1H), 6.79 – 6.67 (m, 2H), 5.39 (s, 1H), 5.32 (s, 1H), 2.05 (s, 2H), 1.52 (s, 3H), 1.43 (s, 9H).

^{13}C NMR (101 MHz, $CDCl_3$) δ 175.3, 149.1, 137.2, 130.2, 128.6, 127.7, 127.4, 126.5, 112.3, 81.3, 60.7, 27.8, 25.7.

HRMS (ESI) calcd. for $C_{14}H_{20}NO_2^+$ $(M + H)^+$: 274.1807, Found: 274.1799

the ee value was 91%, t_r (major) = 9.473 min, t_r (minor) = 14.874 min (Chiralcel IC, λ = 220 nm, hexanes : ^{i}PrOH = 95 : 5, flow rate = 1.0 mL/min).

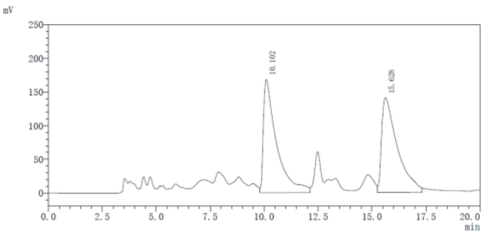

检测器A 220nm

Peak#	Time	Area	Height	Height%	Area%
1	10.102	7153118	168549	54.497	49.976
2	15.628	7159908	140733	45.503	50.024
总计		14313027	309282	100.000	100.000

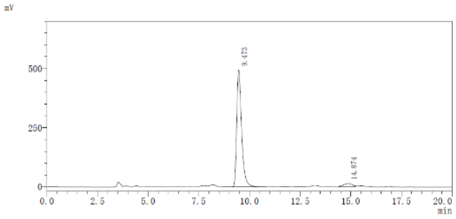

检测器A 220nm

Peak#	Time	Area	Height	Height%	Area%
1	9.473	8366478	494116	97.484	95.632
2	14.874	382152	12752	2.516	4.368
总计		8748631	506868	100.000	100.000

Supplementary Fig. 36 HPLC chromatogram for compound (S)-3aj

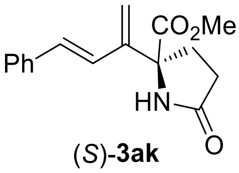

(*S*)-**3ak**

(*S*,*E*)-methyl 5-oxo-2-(4-phenylbuta-1,3-dien-2-yl)pyrrolidine-2-carboxylate

Prepared according to General Procedure B using **L11-Pd** and (*S*,*S*$_p$)-**L3-Cu**, Purification by flash chromatography (PE/EA = 5:1 to 1:3) afforded the product as a pale yellow solid, 31.6 mg, 58% yield, 92% ee, R_f = 0.4 (PE/EA, 1:2).

$[\alpha]^{25}_D$ = -20.1 (c 0.8, $CHCl_3$)

^{1}H NMR (400 MHz, $CDCl_3$) δ 7.40 (d, *J* = 7.3 Hz, 2H), 7.33 (t, *J* = 7.4 Hz, 2H), 7.26 (t, *J* = 7.2 Hz, 1H), 6.82 (s, 1H), 6.70 (q, *J* = 16.4 Hz, 2H), 5.46 (s, 1H), 5.31 (s, 1H), 3.76 (s, 3H), 2.96 (ddd, *J* = 13.1, 9.1, 6.5 Hz, 1H), 2.52 – 2.34 (m, 2H), 2.25 (ddd, *J* = 13.1, 9.0, 7.1 Hz, 1H).

^{13}C NMR (101 MHz, $CDCl_3$) δ 177.0, 171.9, 145.4, 136.5, 131.4, 128.7, 128.2, 126.7, 125.1, 112.9, 68.2, 53.2, 30.1, 29.5.

HRMS (ESI) calcd. for $C_{14}H_{20}NO_2^+$ $(M + H)^+$: 272.1287, Found: 272.1278

the ee value was 92%, t_r (minor) = 25.970 min, t_r (major) = 46.271 min (Chiralcel IC, λ = 220 nm, hexanes : ^{i}PrOH = 85 : 15, flow rate = 1.0 mL/min).

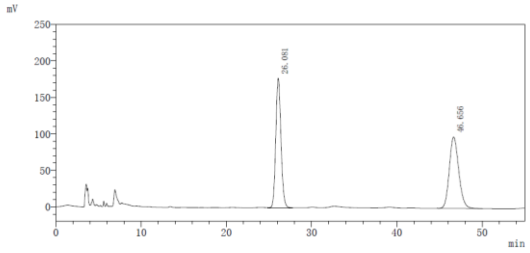

检测器A 220nm

Peak#	Time	Area	Height	Height%	Area%
1	26.081	7500304	177777	64.477	49.972
2	46.656	7508653	97943	35.523	50.028
总计		15008957	275720	100.000	100.000

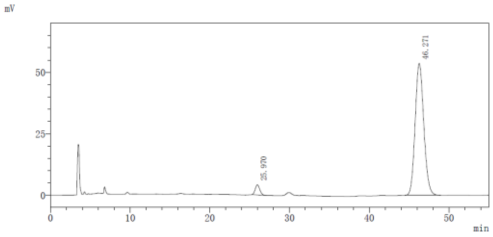

检测器A 220nm

Peak#	Time	Area	Height	Height%	Area%
1	25.970	164055	4100	7.096	3.897
2	46.271	4045909	53688	92.904	96.103
总计		4209963	57789	100.000	100.000

Supplementary Fig. 37 HPLC chromatogram for compound (S)-3ak

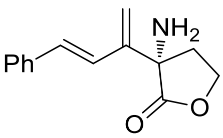

(*S*)-**3al**

(*S*,*E*)-3-amino-3-(4-phenylbuta-1,3-dien-2-yl)dihydrofuran-2(3H)-one

Prepared according to General Procedure B using **L11-Pd** and (S,S_P)-**L3-Cu**, Purification by flash chromatography (PE/EA = 5:1 to 1:3) afforded the product as a pale yellow solid, 25.2 mg, 55% yield, 98% ee, R_f = 0.3 (PE/EA, 1:2). m. p. 98-100 °C.

$[\alpha]^{25}_D$ = -35.0 (c 0.8, $CHCl_3$)

^{1}H NMR (400 MHz, $CDCl_3$) δ 7.44 (d, *J* = 7.3 Hz, 2H), 7.34 (t, *J* = 7.5 Hz, 2H), 7.26 (dd, *J* = 7.6, 6.9 Hz, 1H), 6.88 (dd, *J* = 77.5, 16.2 Hz, 2H), 5.50 (s, 1H), 5.15 (s, 1H), 4.42 – 4.30 (m, 1H), 4.16 (td, *J* = 9.5, 6.1 Hz, 1H), 2.65 (ddd, *J* = 13.0, 6.1, 2.5 Hz, 1H), 2.24 (ddd, *J* = 13.0, 9.8, 8.2 Hz, 1H), 1.78 (s, 2H).

^{13}C NMR (101 MHz, $CDCl_3$) δ 179.4, 144.7, 136.7, 132.3, 128.7, 128.1, 126.7, 125.2, 113.9, 64.7, 62.6, 36.6.

HRMS (ESI) calcd. for $C_{14}H_{20}NO_2^+$ $(M + H)^+$: 230.1181, Found: 230.1174

the ee value was 98%, t_r (major) = 28.922 min, t_r (minor) = 31.593 min (Chiralcel IC, λ = 220 nm, hexanes : ^{i}PrOH = 85 : 15, flow rate = 1.0 mL/min).

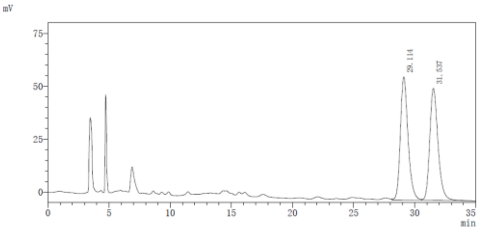

检测器A 220nm

Peak#	Time	Area	Height	Height%	Area%
1	29.114	2525484	58110	52.358	50.346
2	31.537	2490761	52876	47.642	49.654
总计		5016245	110986	100.000	100.000

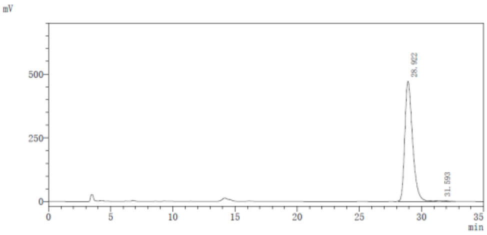

检测器A 220nm

Peak#	Time	Area	Height	Height%	Area%
1	28.922	20466562	472627	99.219	99.198
2	31.593	165454	3721	0.781	0.802
总计		20632015	476348	100.000	100.000

Supplementary Fig. 38 HPLC chromatogram for compound (S)-3al

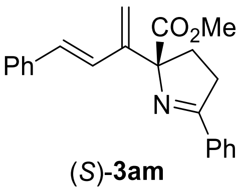

(*S*)-**3am**

(*S*,*E*)-methyl 5-phenyl-2-(4-phenylbuta-1,3-dien-2-yl)-3,4-dihydro-2H-pyrrole-2-carboxylate

Prepared according to General Procedure B using **L11-Pd** and (S,S_P)-**L3-Cu**, Purification by flash chromatography (PE/EA = 30:1 to 15:1) afforded the product as a pale yellow solid, 59.1 mg, 89% yield, 99% ee, R_f = 0.3 (PE/EA, 15:1). m. p. 96-98 °C.

$[\alpha]^{25}_D$ = -180.2 (c 1.5, $CHCl_3$)

^{1}H NMR (400 MHz, $CDCl_3$) δ 7.81 – 7.74 (m, 2H), 7.32 – 7.21 (m, 5H), 7.14 (t, *J* = 7.5 Hz, 2H), 7.06 (dd, *J* = 8.4, 6.1 Hz, 1H), 6.55 (dd, *J* = 71.7, 16.6 Hz, 2H), 5.27 (s, 1H), 5.21 (s, 1H), 3.56 (s, 3H), 3.09 – 2.83 (m, 3H), 1.93 (ddd, *J* = 12.0, 9.3, 5.9 Hz, 1H).

^{13}C NMR (101 MHz, $CDCl_3$) δ 175.5, 173.3, 147.4, 137.2, 133.9, 131.2, 129.56, 128.7, 128.5, 128.3, 128.2, 127.8, 126.6, 115.3, 85.6, 53.1, 35.8, 32.2.

HRMS (ESI) calcd. for $C_{14}H_{20}NO_2^+$ $(M + H)^+$: 332.1651, Found: 332.1643

the ee value was 99%, t_r (major) = 13.730 min, t_r (minor) = 17.041 min (Chiralcel IC, λ = 220 nm, hexanes : ^{i}PrOH = 98 : 2, flow rate = 1.0 mL/min).

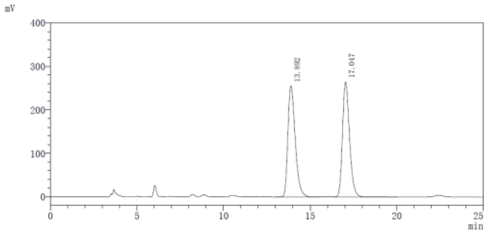

检测器A 220nm

Peak#	Time	Area	Height	Height%	Area%
1	13.892	7548319	255582	49.133	50.187
2	17.047	7491986	264605	50.867	49.813
总计		15040305	520187	100.000	100.000

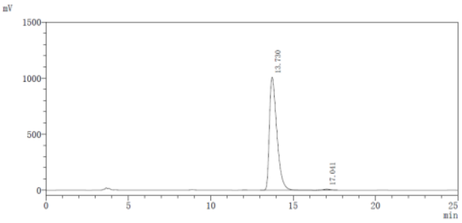

检测器A 220nm

Peak#	Time	Area	Height	Height%	Area%
1	13.730	31626281	1008537	99.181	99.276
2	17.041	230735	8330	0.819	0.724
总计		31857016	1016867	100.000	100.000

Supplementary Fig. 39 HPLC chromatogram for compound (S)-3am

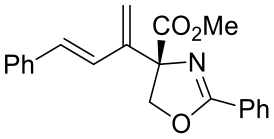

(*S*)-**3an**

(*S*,*E*)-methyl 2-phenyl-4-(4-phenylbuta-1,3-dien-2-yl)-4,5-dihydrooxazole-4-carboxylate

Prepared according to General Procedure B using **L11-Pd** and (*S*,S_p)-**L3-Cu**, Purification by flash chromatography (PE/EA = 30:1 to 15:1) afforded the product as a pale yellow solid, 50.9 mg, 76% yield, >99% ee, R_f = 0.3 (PE/EA, 15:1). m. p. 148-149 °C.

$[\alpha]^{25}_D$ = -350.5 (c 0.5, $CHCl_3$)

^{1}H NMR (400 MHz, $CDCl_3$) δ 8.06 (d, *J* = 7.3 Hz, 2H), 7.52 (t, *J* = 7.3 Hz, 1H), 7.43 (dd, *J* = 12.6, 7.5 Hz, 4H), 7.33 (t, *J* = 7.5 Hz, 2H), 7.26 (t, *J* = 7.2 Hz, 1H), 6.80 (d, *J* = 16.8 Hz, 1H), 6.35 (d, *J* = 16.8 Hz, 1H), 5.70 (s, 1H), 5.48 (s, 1H), 5.46 (d, *J* = 8.6 Hz, 1H), 4.33 (d, *J* = 8.6 Hz, 1H), 3.79 (s, 3H).

^{13}C NMR (101 MHz, $CDCl_3$) δ 172.1, 165.2, 145.9, 136.7, 132.0, 129.4, 128.8, 128.7, 128.4, 128.1, 127.4, 127.0, 126.6, 117.6, 79.8, 74.9, 53.5.

HRMS (ESI) calcd. for $C_{14}H_{20}NO_2^+$ $(M + H)^+$: 334.1443, Found: 334.1434

the ee value was > 99%, t_r (major) = 12.575 min, t_r (minor) = 13.910 min (Chiralcel IC, λ = 220 nm, hexanes : ^{i}PrOH = 98 : 2, flow rate = 1.0 mL/min).

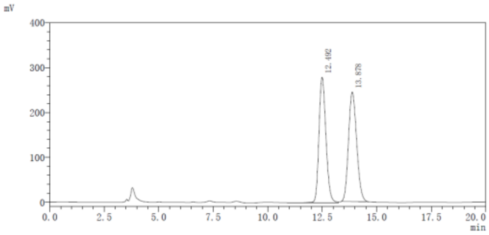

检测器A 220nm

Peak#	Time	Area	Height	Height%	Area%
1	12.492	6223672	279977	53.437	49.637
2	13.878	6314649	243957	46.563	50.363
总计		12538321	523935	100.000	100.000

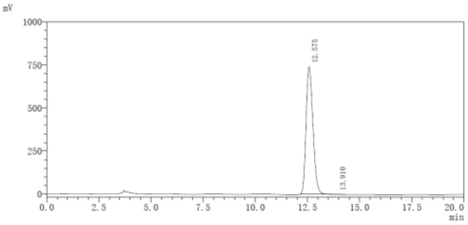

检测器A 220nm

Peak#	Time	Area	Height	Height%	Area%
1	12.575	17037432	742466	99.856	99.872
2	13.910	21891	1070	0.144	0.128
总计		17059323	743536	100.000	100.000

Supplementary Fig. 40 HPLC chromatogram for compound (S)-3an

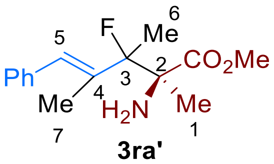

3ra'

Prepared according to General Procedure A using **L11-Pd** and (*S*,*S*$_P$)-**L3-Cu**, Purification by flash chromatography (PE/EA = 5:1 to 1:1) afforded the product **3ra'**, pale yellow oil, 28% yield , > 99% ee, >20:1 dr, R_f = 0.4 (PE/EA, 1:1).

$[\alpha]^{25}_D$ = -2.4 (c 0.5, $CHCl_3$)

^{1}H NMR (400 MHz, $CDCl_3$) δ 7.34 (t, *J* = 7.5 Hz, 2H), 7.27 – 7.23 (m, 3H), 6.61 (s, 1H), 3.72 (s, 4H), 1.84 (s, 3H), 1.81 (s, 3H), 1.68 (d, *J* = 24.0 Hz, 4H), 1.47 (s, 3H).

^{13}C NMR (101 MHz, $CDCl_3$) δ 174.7, 137.3, 137.0 (d, *J* = 18.0 Hz, C4), 129.1, 128.2, 126.9 (d, *J* = 14.7 Hz, C5), 126.7, 100.3 (d, *J* = 183.8 Hz, C3), 63.8 (d, *J* = 25.3 Hz, C2), 52.4, 21.9 (d, *J* = 3.1 Hz, C1), 21.1 (d, *J* = 24.6 Hz, C6), 15.7 (d, *J* = 5.5 Hz, C7).

^{19}F NMR (376 MHz, $CDCl_3$) δ -142.09.

HRMS (ESI) calcd. for $C_{15}H_{21}FNO_2^+$ $(M + H)^+$: 266.1551, Found: 266.1551

the ee value was >99%, t_r (major) = 21.047 min, t_r (minor) = 18.170 min (Chiralcel IC, λ = 220 nm, hexanes : ^{i}PrOH = 98 : 2, flow rate = 1.0 mL/min).

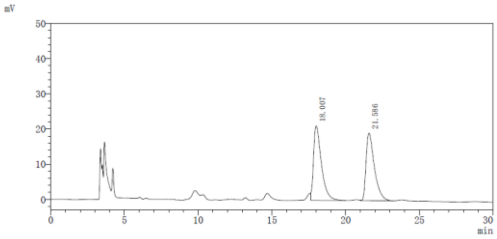

检测器A 220nm

Peak#	Time	Area	Height	Height%	Area%
1	18.007	776904	21159	52.408	50.764
2	21.586	753517	19214	47.592	49.236
总计		1530421	40373	100.000	100.000

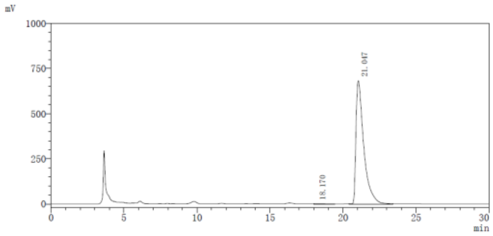

检测器A 220nm

Peak#	Time	Area	Height	Height%	Area%
1	18.170	12018	318	0.047	0.048
2	21.047	25271307	682166	99.953	99.952
总计		25283325	682484	100.000	100.000

Supplementary Fig. 41 HPLC chromatogram for compound (S)-3ra′

3.2 Copies of NMR Spectrum

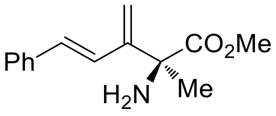

(S)-**3aa**

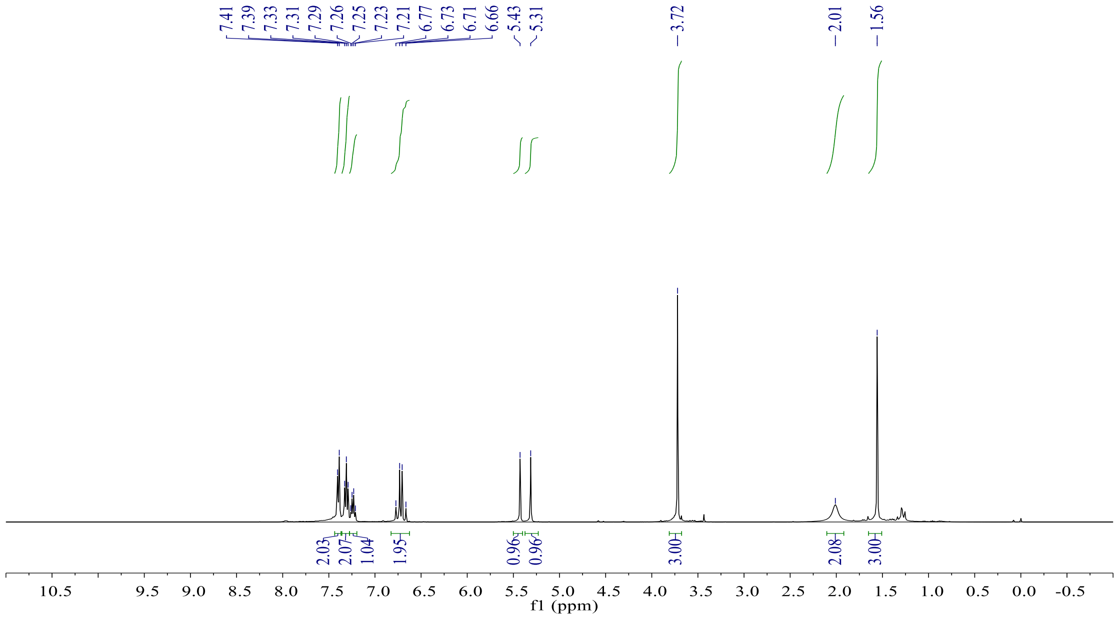

Supplementary Fig. 42 ^{1}H NMR (400 MHz, CDCl$_3$) spectrum of (*S*)-3aa

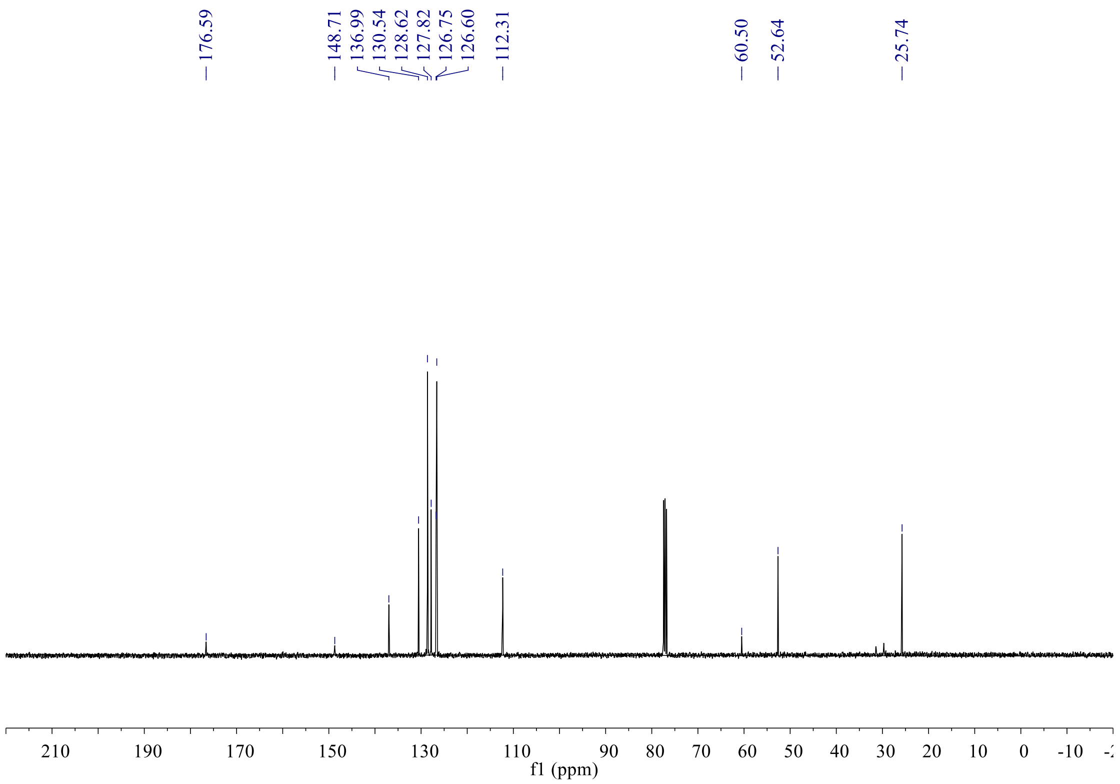

Supplementary Fig. 43 ^{13}C NMR (100 MHz, CDCl$_3$) spectrum of (*S*)-3aa

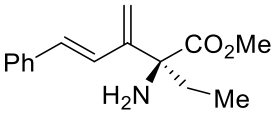

(S)-**3ab**

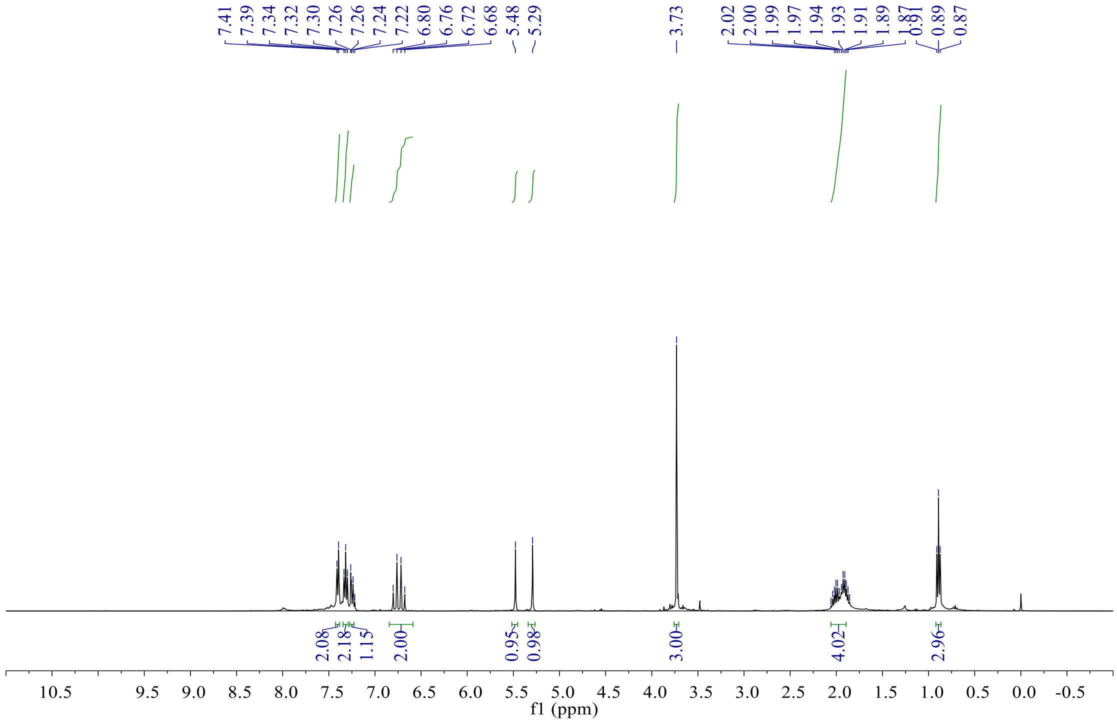

Supplementary Fig. 44 ^{1}H NMR (400 MHz, CDCl$_3$) spectrum of (*S*)-3ab

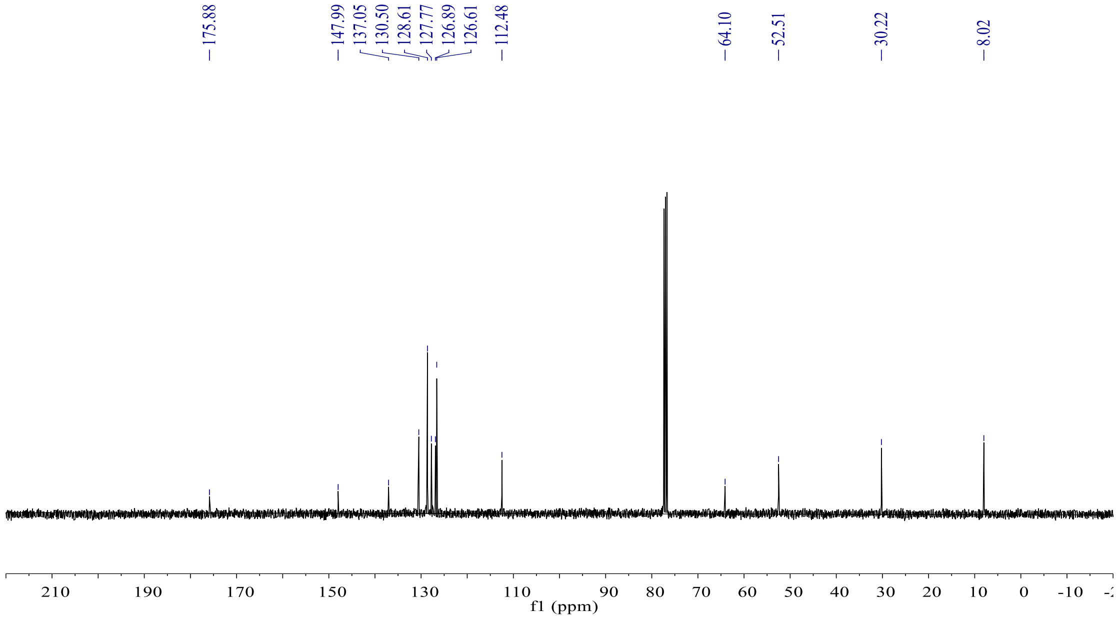

Supplementary Fig. 45 ^{13}C NMR (100 MHz, CDCl$_3$) spectrum of (*S*)-3ab

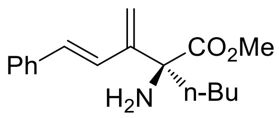

(S)-**3ac**

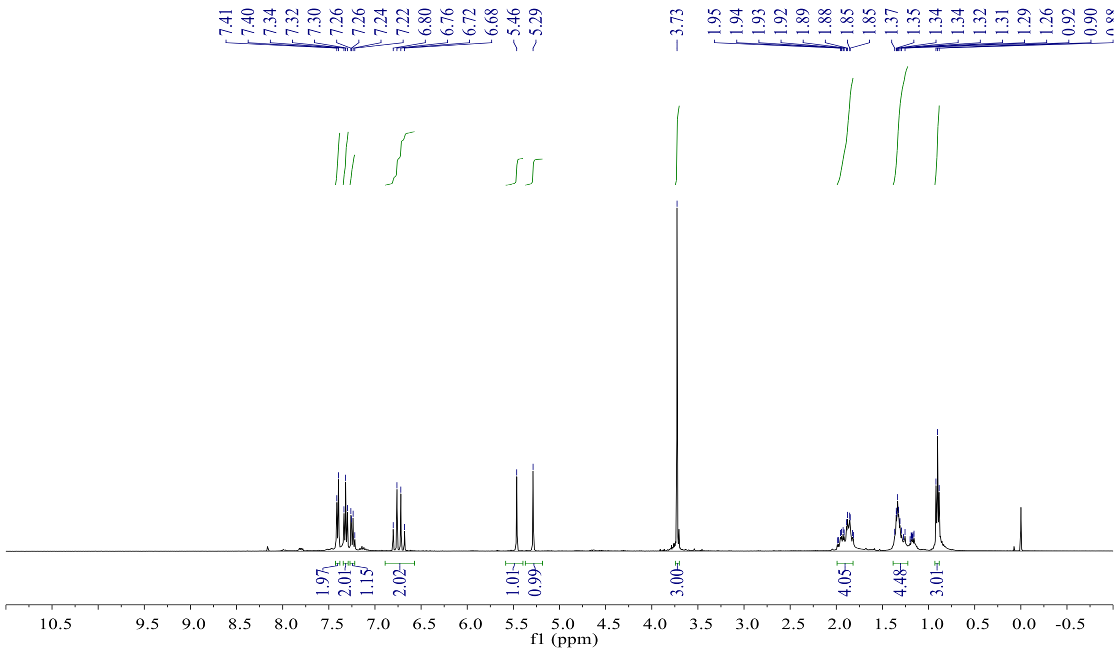

Supplementary Fig. 46 ^{1}H NMR (400 MHz, CDCl$_3$) spectrum of (*S*)-3ac

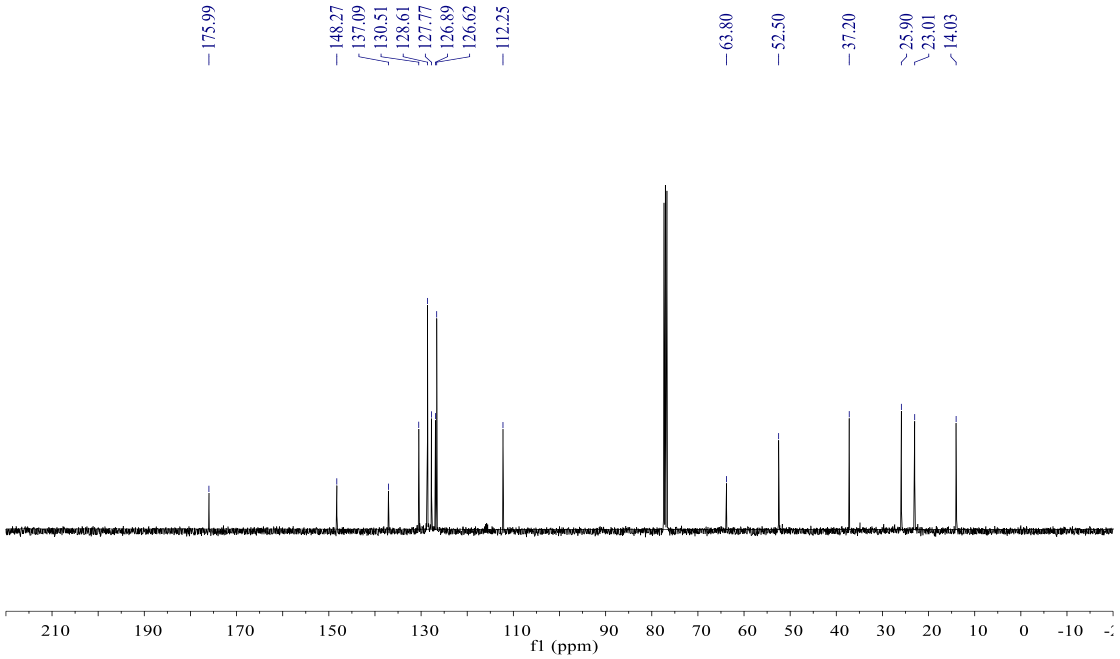

Supplementary Fig. 47 ^{13}C NMR (100 MHz, CDCl$_3$) spectrum of (*S*)-3ac

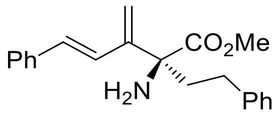

(*S*)-**3ad**

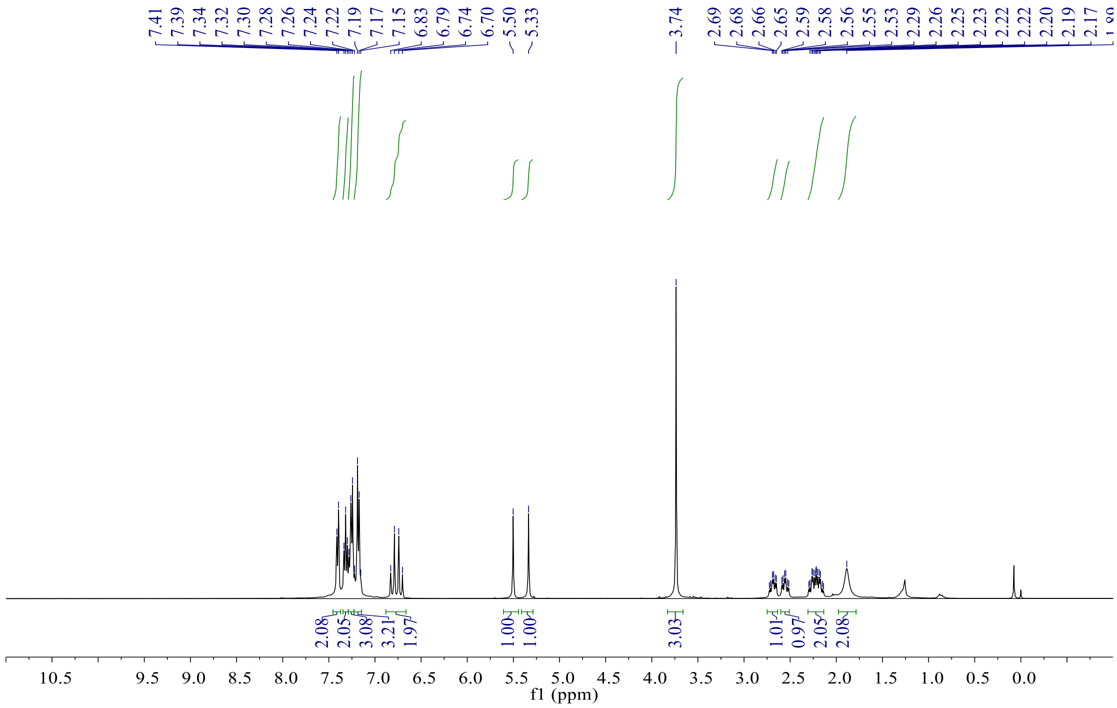

Supplementary Fig. 48 ^{1}H NMR (400 MHz, CDCl3) spectrum of (*S*)-3ad

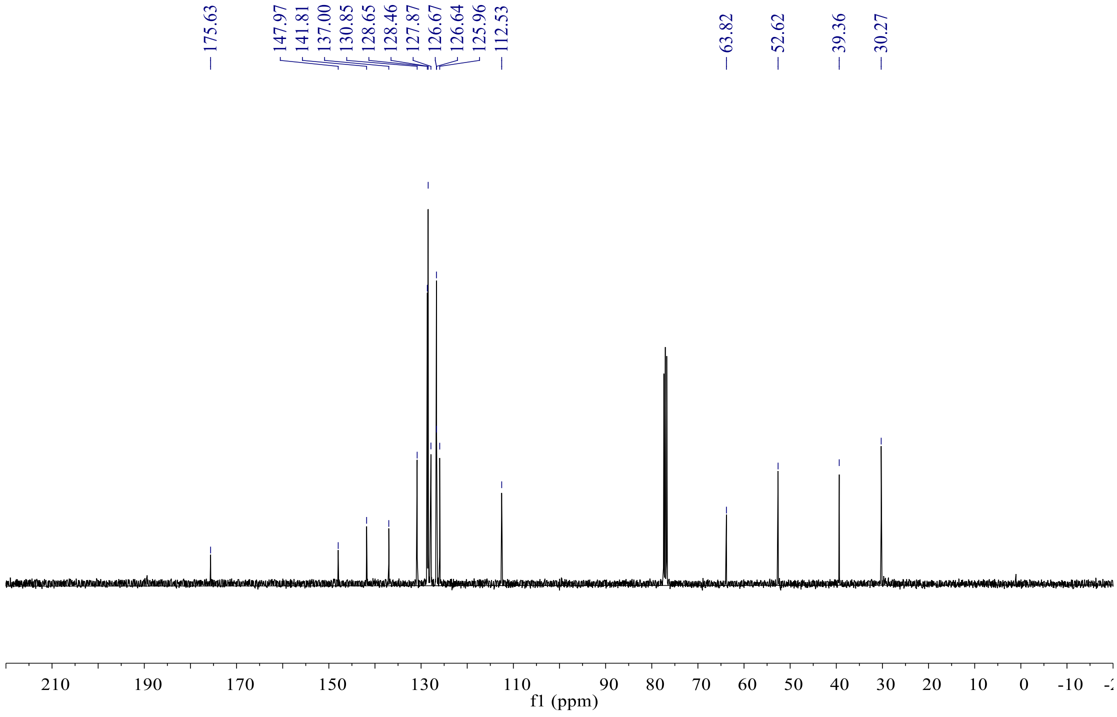

Supplementary Fig. 49 ^{13}C NMR (100 MHz, CDCl3) spectrum of (*S*)-3ad

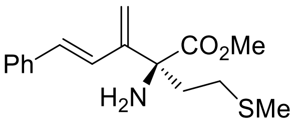

(*S*)-**3ae**

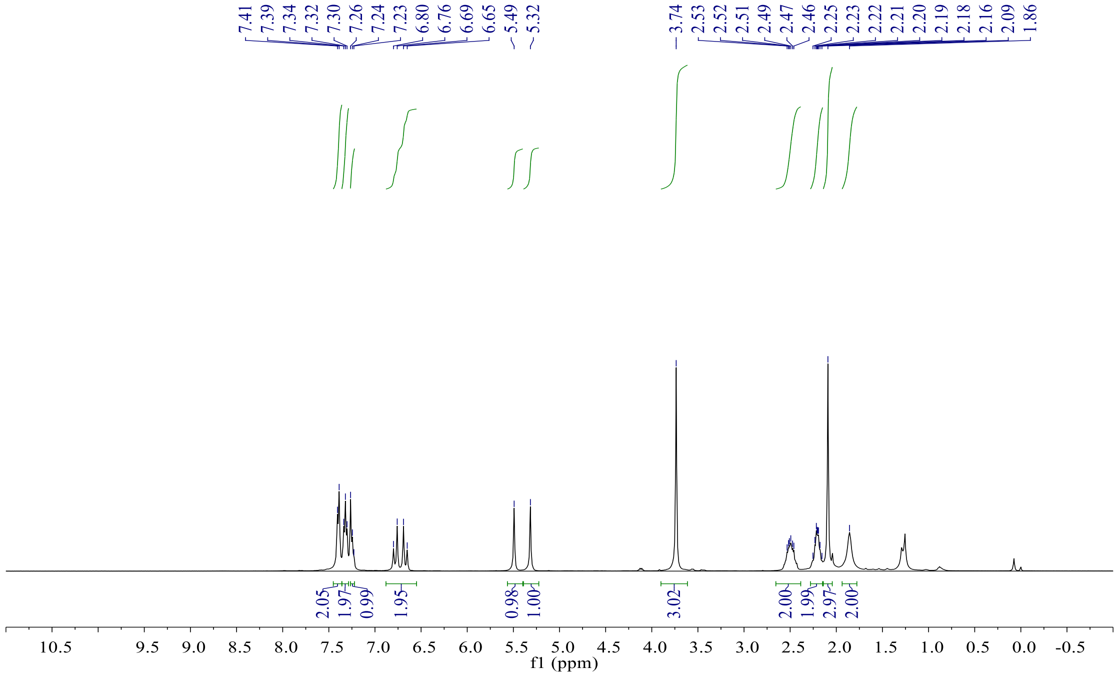

Supplementary Fig. 50 ^{1}H NMR (400 MHz, $CDCl_3$) spectrum of (*S*)-3ae

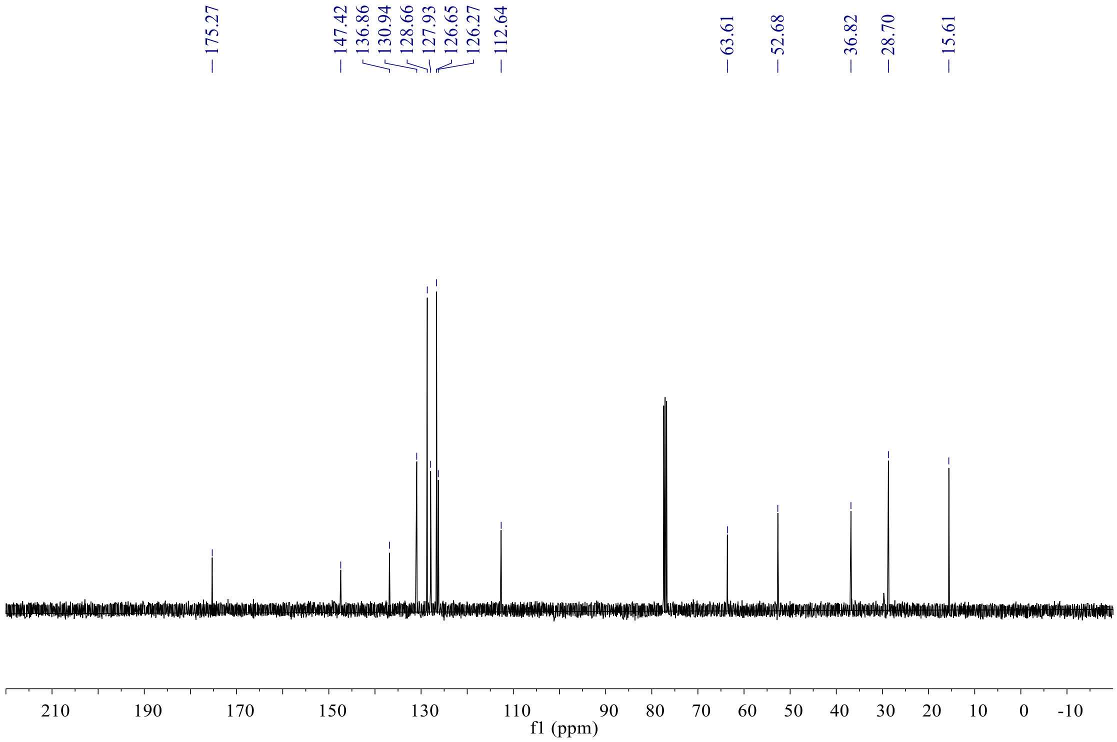

Supplementary Fig. 51 ^{13}C NMR (100 MHz, $CDCl_3$) spectrum of (*S*)-3ae

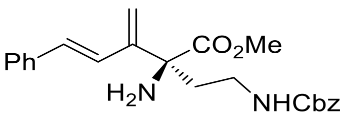

(*S*)-**3af**

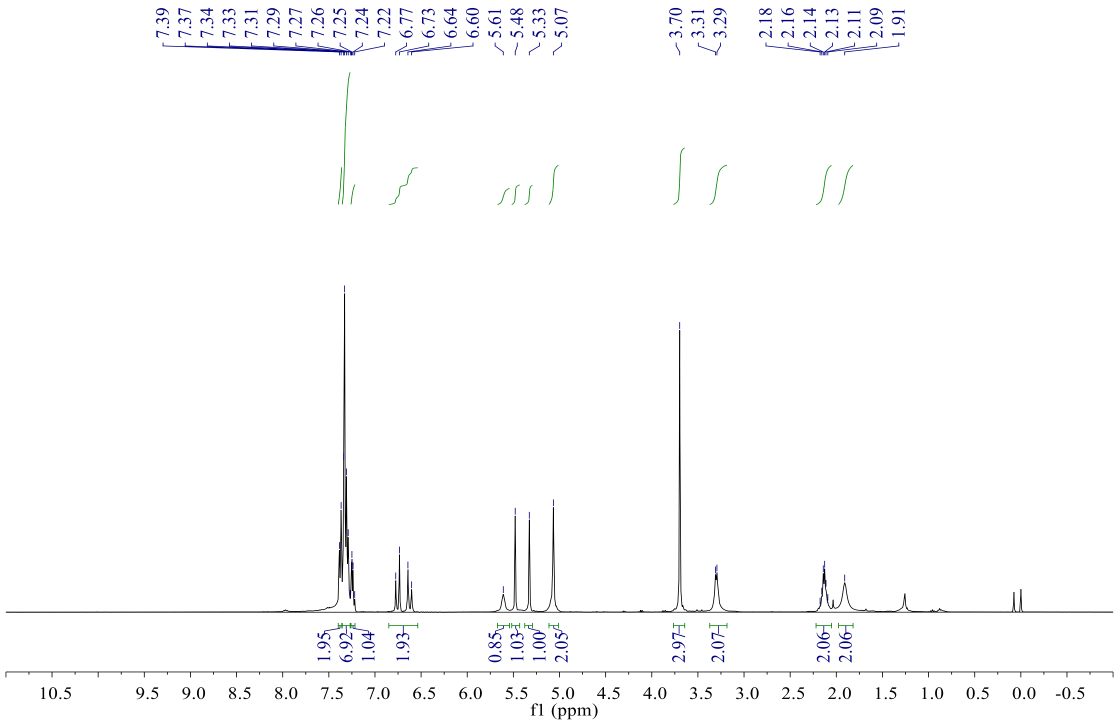

Supplementary Fig. 52 ^{1}H NMR (400 MHz, CDCl$_3$) spectrum of (*S*)-3af

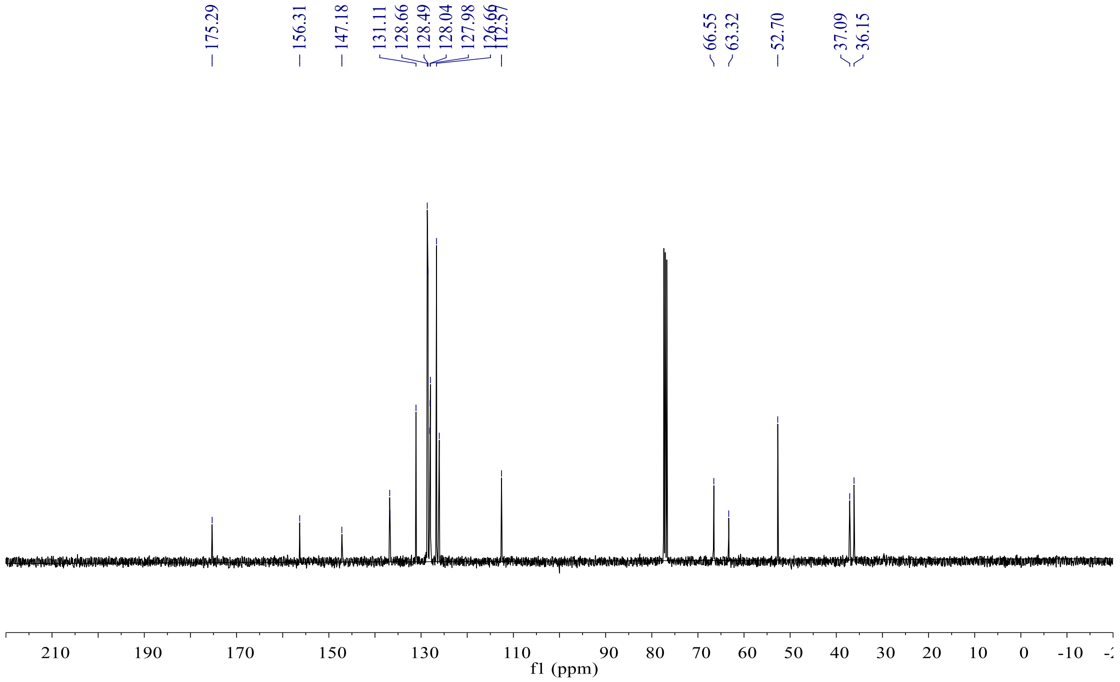

Supplementary Fig. 53 ^{13}C NMR (100 MHz, CDCl$_3$) spectrum of (*S*)-3af

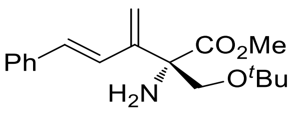

(*S*)-**3ag**

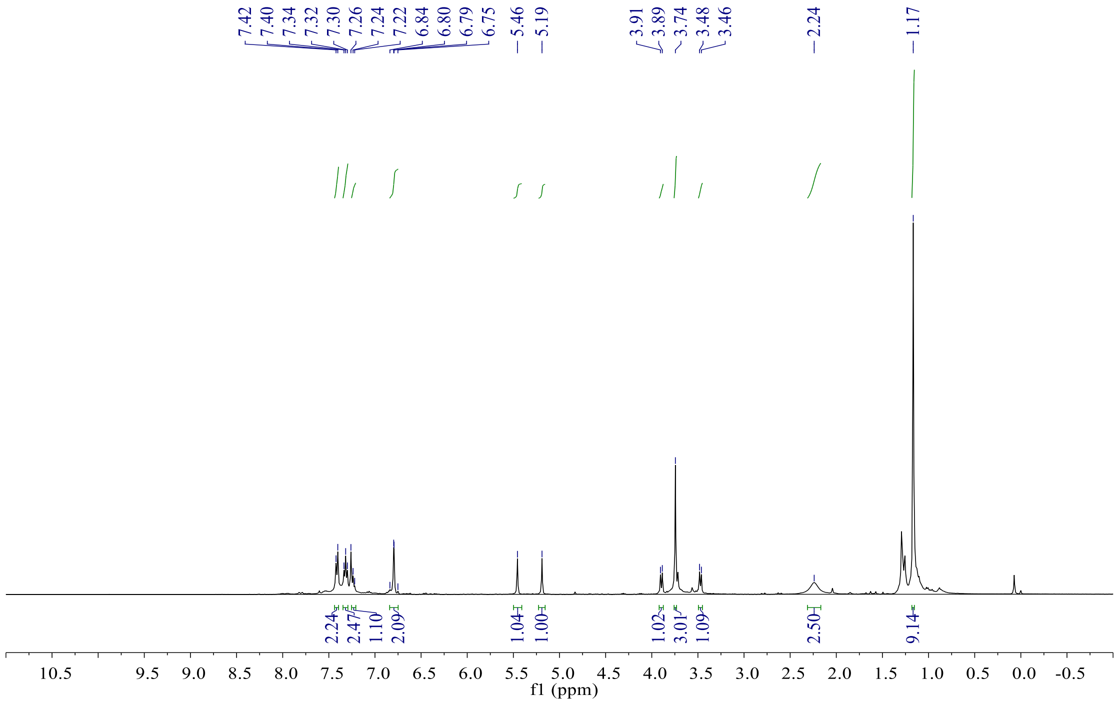

Supplementary Fig. 54 ^{1}H NMR (400 MHz, $CDCl_3$) spectrum of (*S*)-3ag

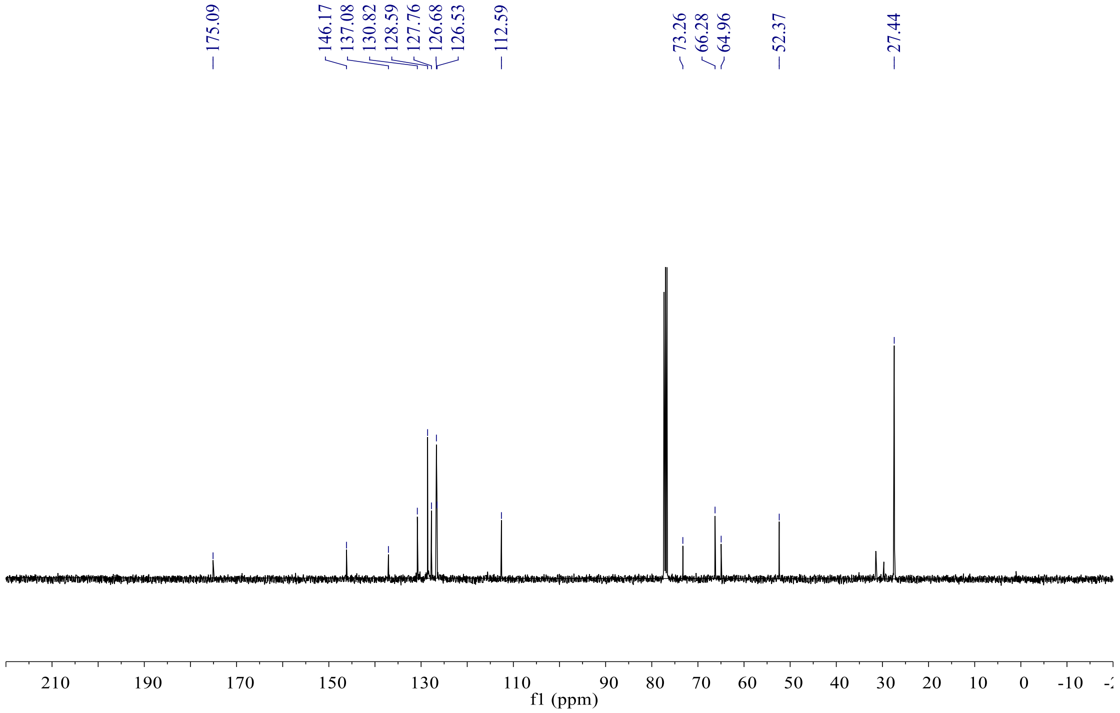

Supplementary Fig. 55 ^{13}C NMR (100 MHz, $CDCl_3$) spectrum of (*S*)-3ag

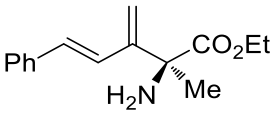

(S)-**3ah**

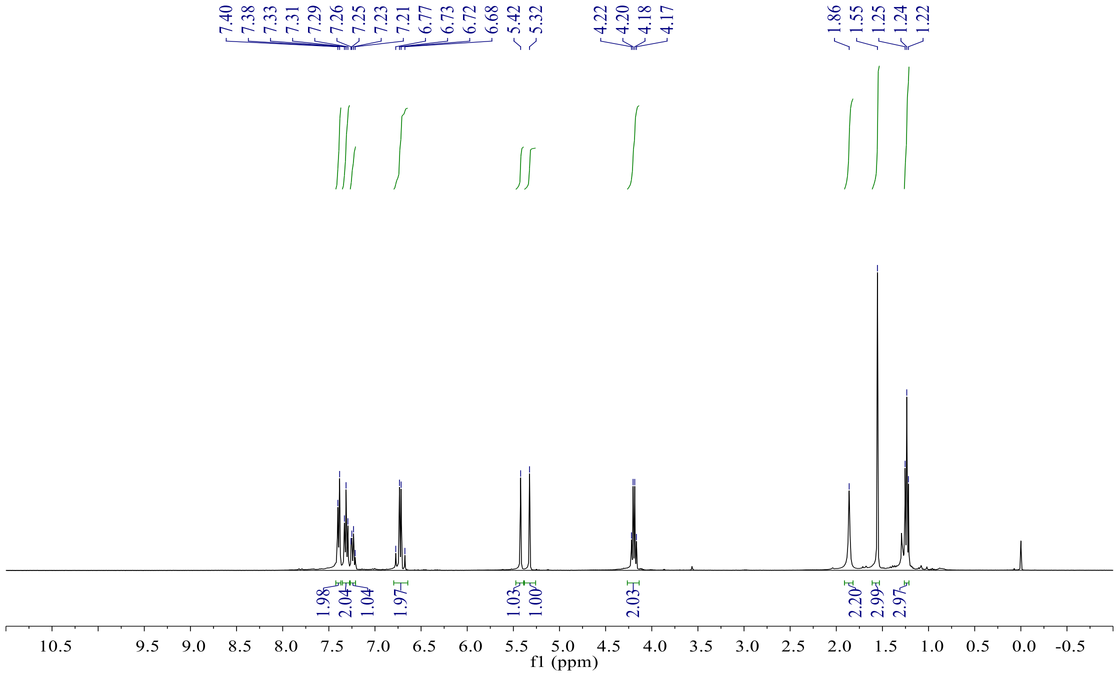

Supplementary Fig. 56 ^{1}H NMR (400 MHz, $CDCl_3$) spectrum of (*S*)-3ah

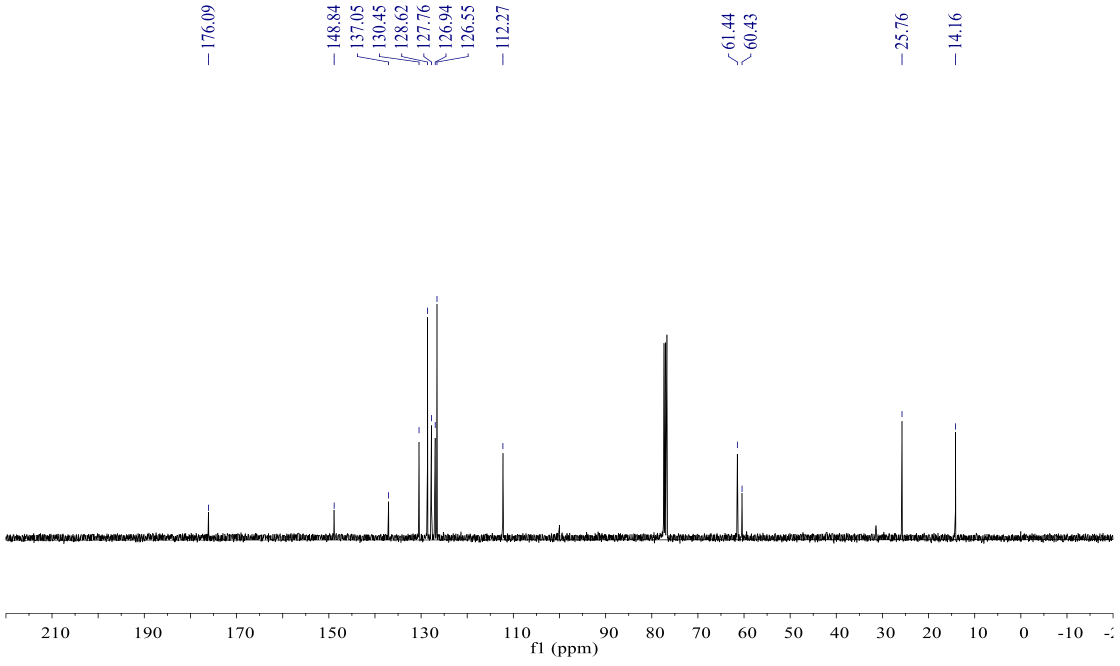

Supplementary Fig. 57 ^{13}C NMR (100 MHz, $CDCl_3$) spectrum of (*S*)-3ah

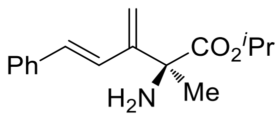

(S)-**3ai**

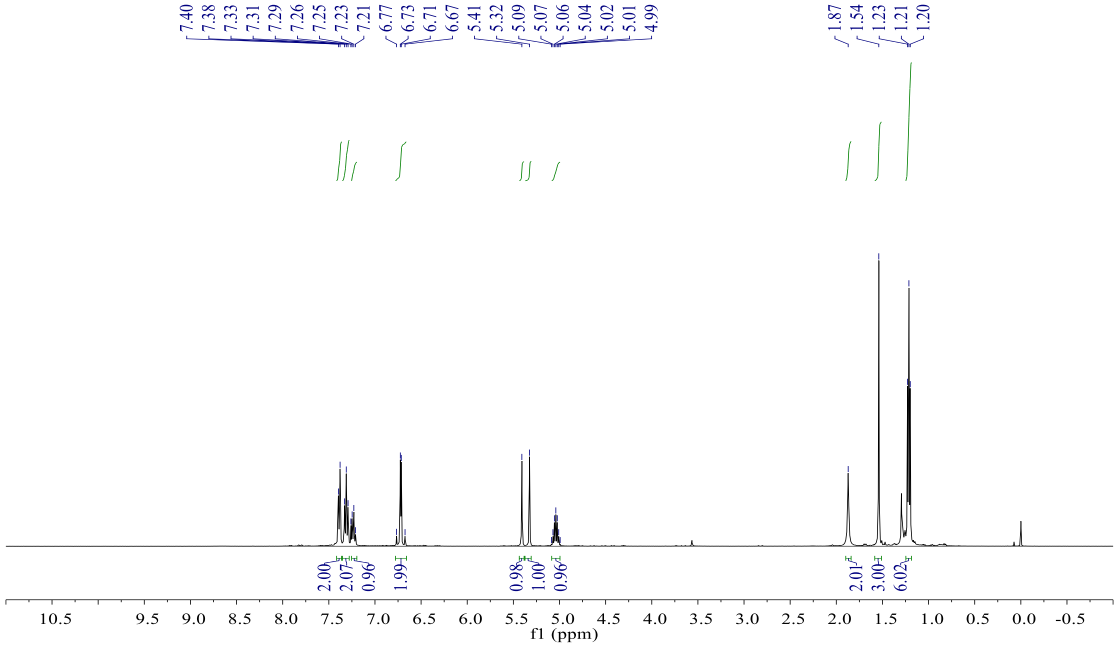

Supplementary Fig. 58 ^{1}H NMR (400 MHz, $CDCl_3$) spectrum of (*S*)-3ai

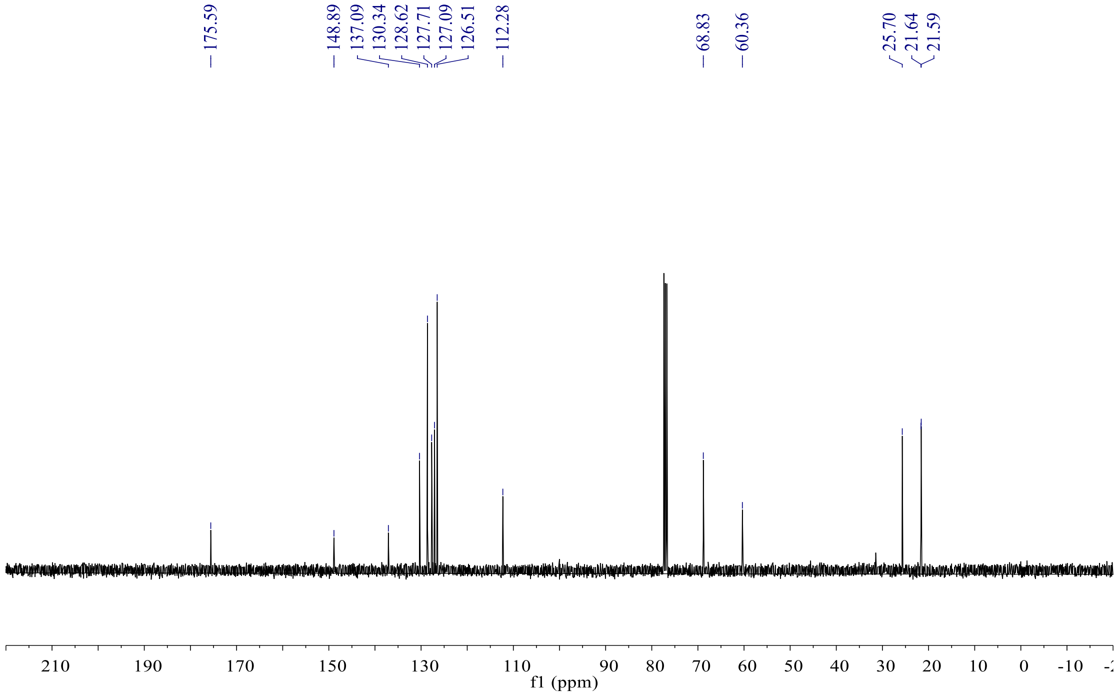

Supplementary Fig. 59 ^{13}C NMR (100 MHz, $CDCl_3$) spectrum of (*S*)-3ai

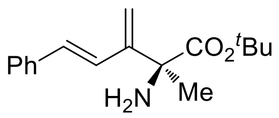

(S)-**3aj**

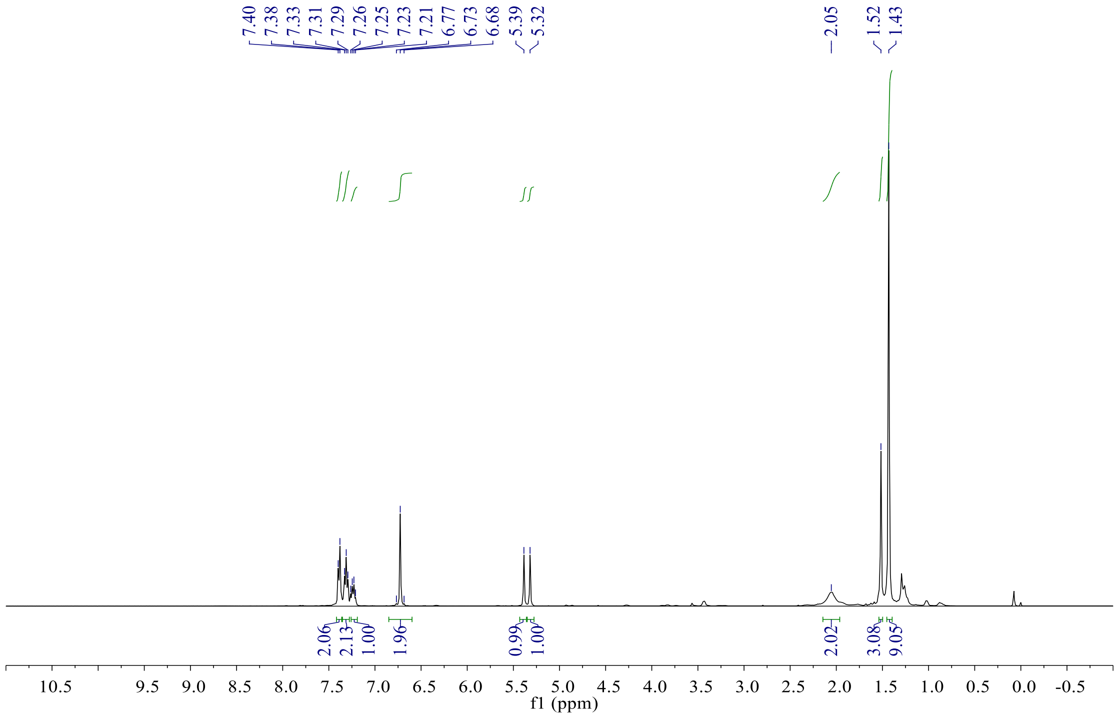

Supplementary Fig. 60 ^{1}H NMR (400 MHz, CDCl$_3$) spectrum of (*S*)-3aj

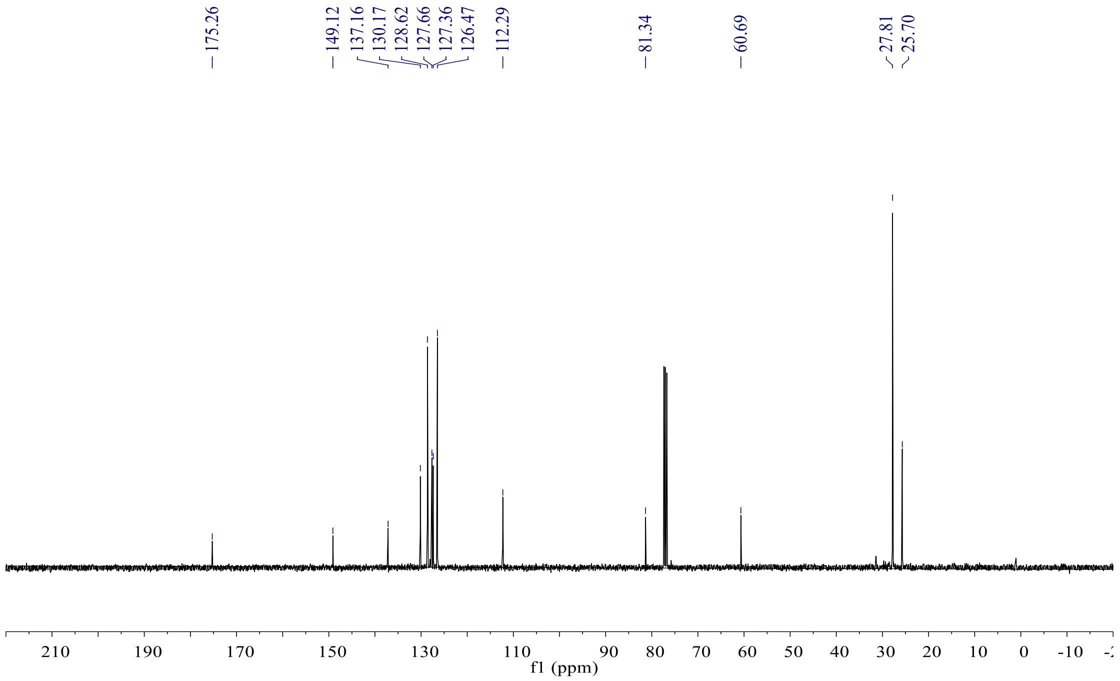

Supplementary Fig. 61 ^{13}C NMR (100 MHz, CDCl$_3$) spectrum of (*S*)-3aj

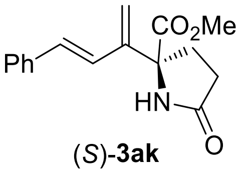

(*S*)-**3ak**

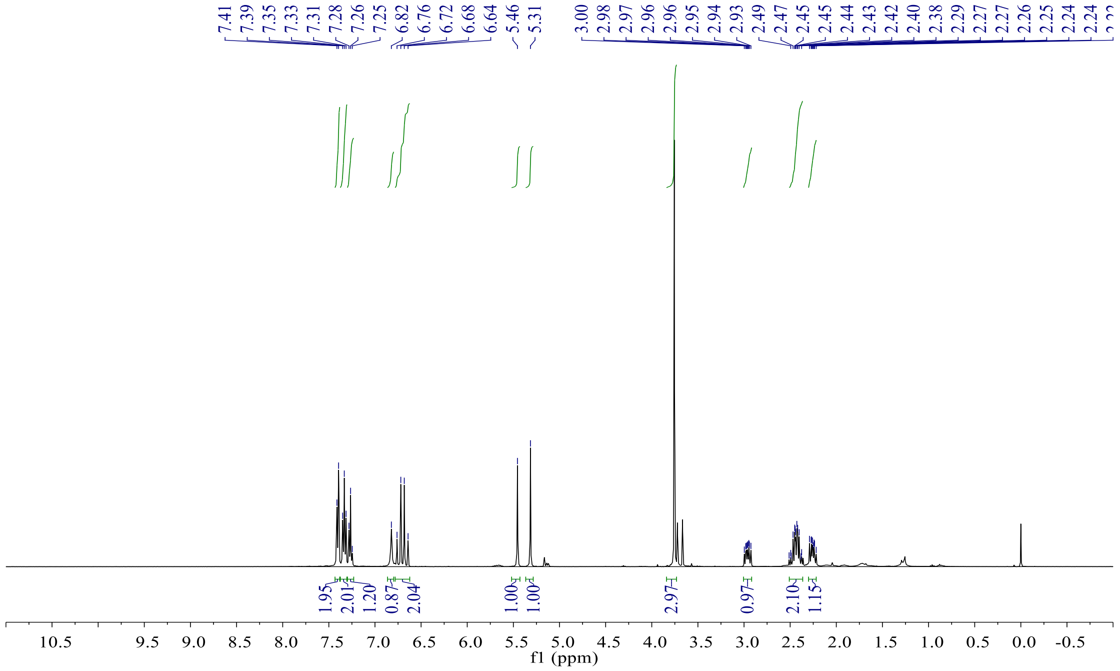

Supplementary Fig. 62 ^{1}H NMR (400 MHz, CDCl$_3$) spectrum of (*S*)-3ak

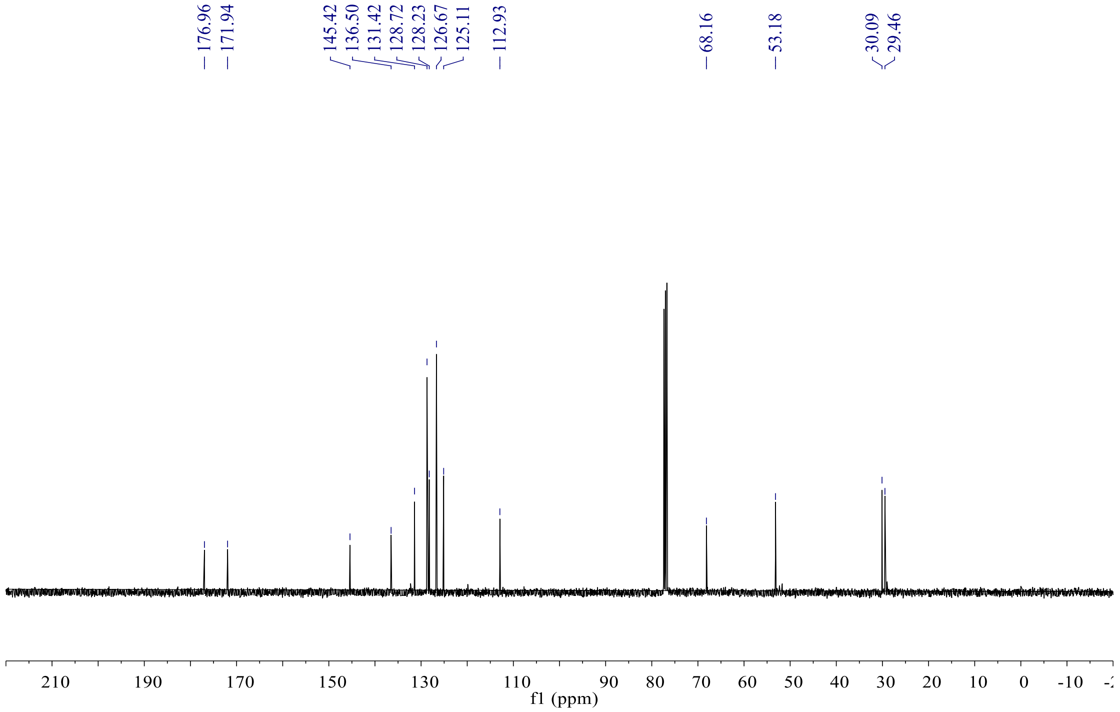

Supplementary Fig. 63 ^{13}C NMR (100 MHz, CDCl$_3$) spectrum of (*S*)-3ak

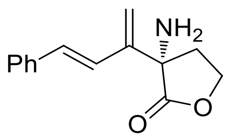

(*S*)-**3al**

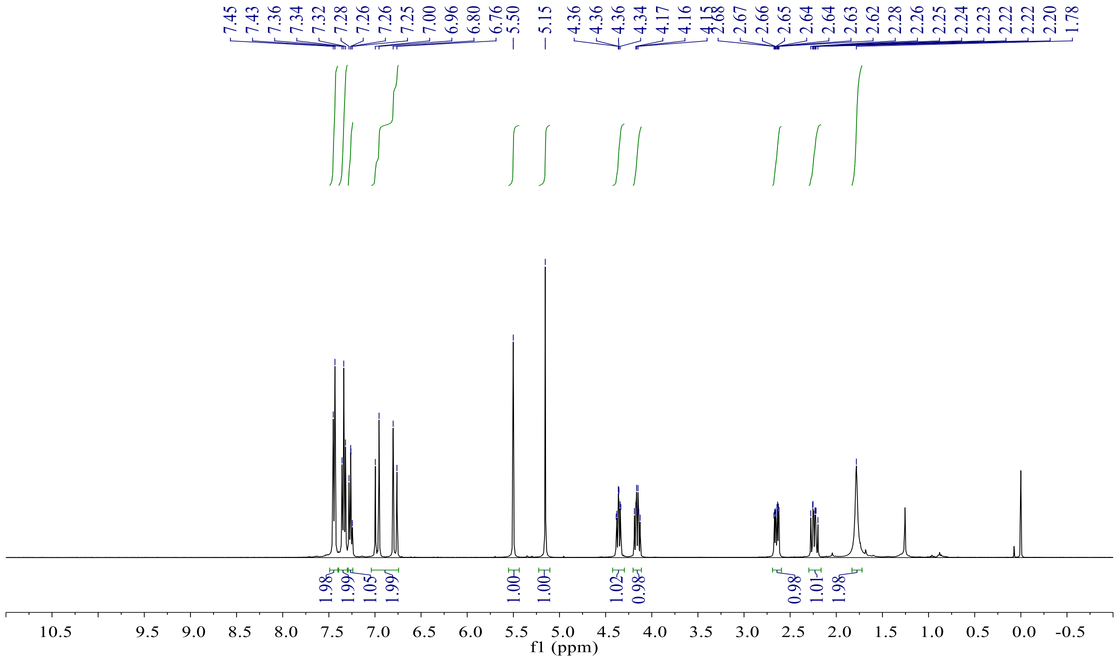

Supplementary Fig. 64 ^{1}H NMR (400 MHz, CDCl$_3$) spectrum of (*S*)-3al

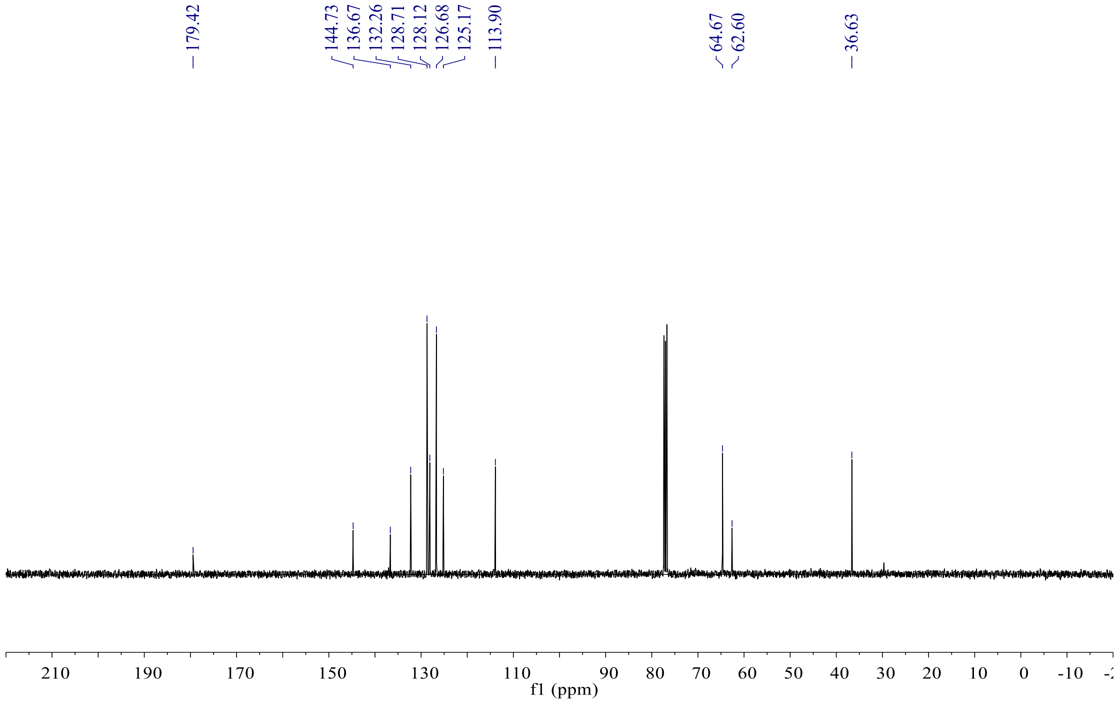

Supplementary Fig. 65 ^{13}C NMR (100 MHz, CDCl$_3$) spectrum of (*S*)-3al

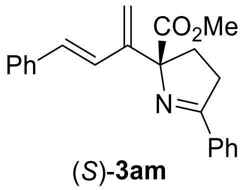

(*S*)-**3am**

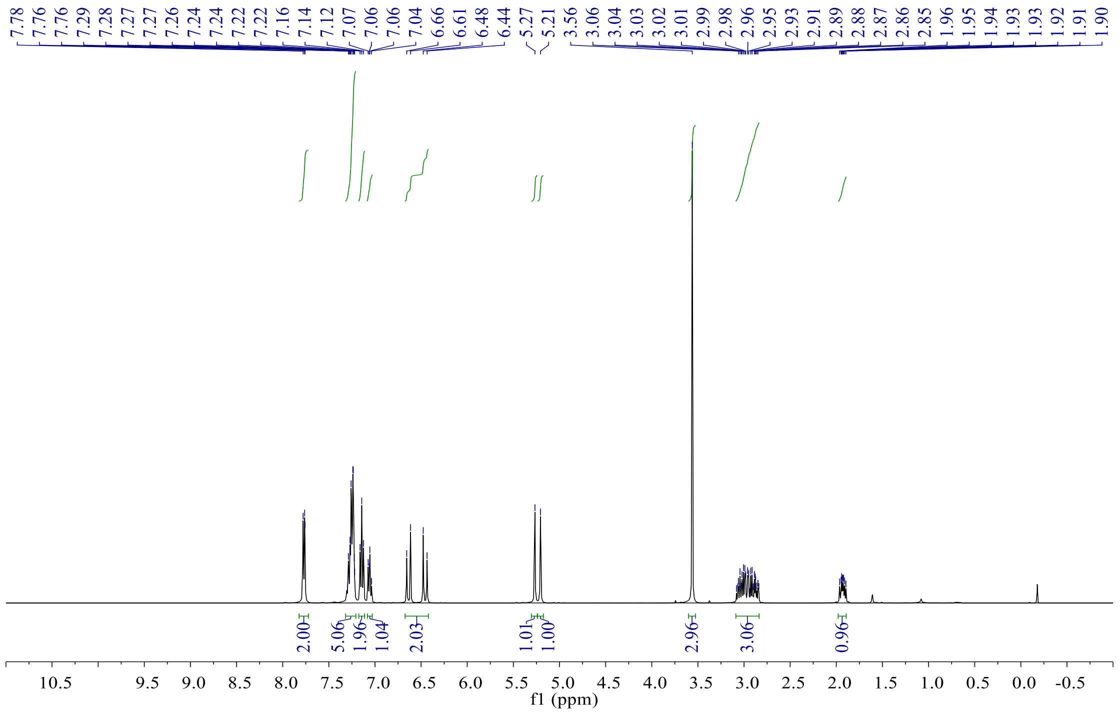

Supplementary Fig. 66 ^{1}H NMR (400 MHz, CDCl$_3$) spectrum of (*S*)-3am

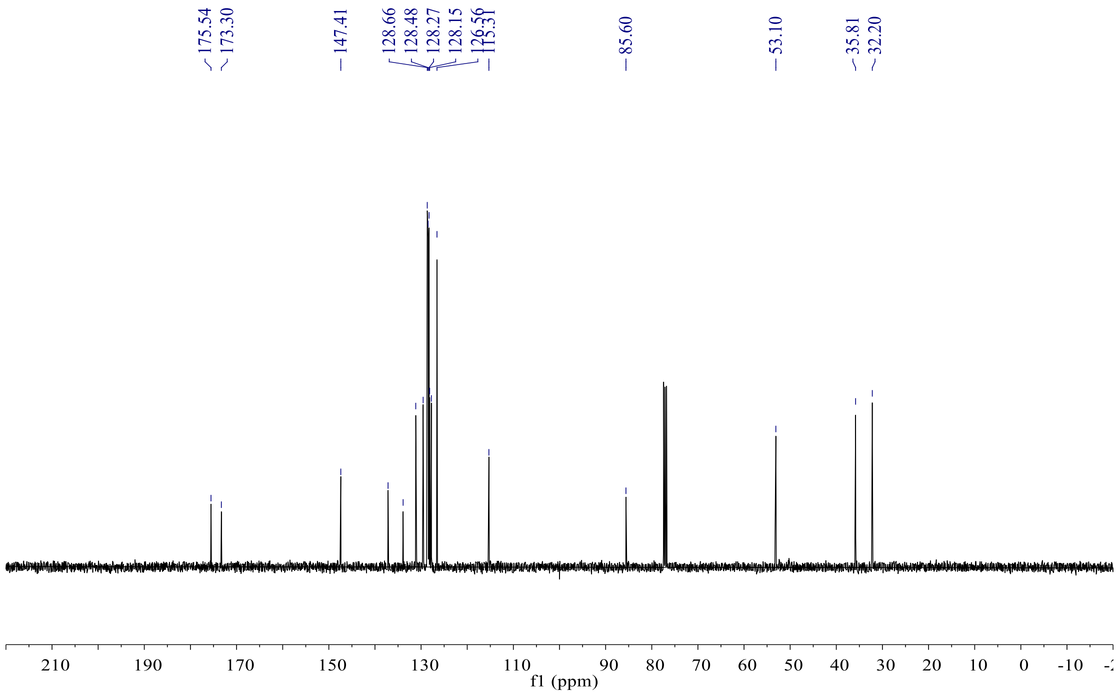

Supplementary Fig. 67 ^{13}C NMR (100 MHz, CDCl$_3$) spectrum of (*S*)-3am

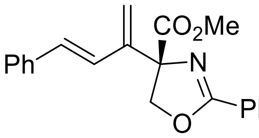

(*S*)-**3an**

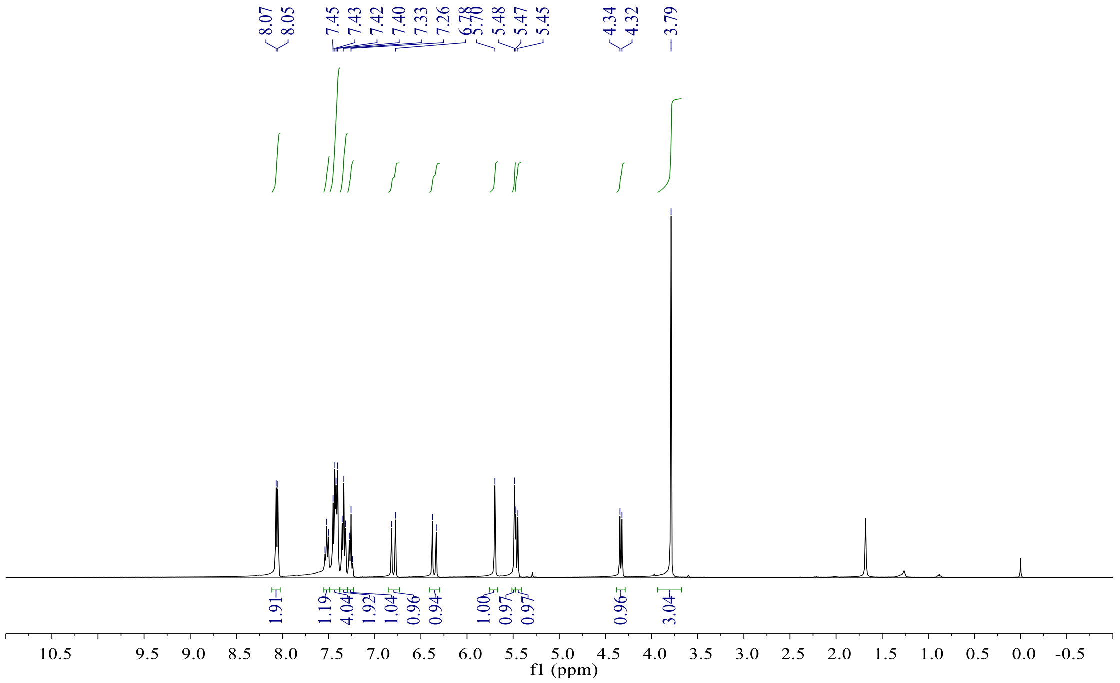

Supplementary Fig. 68 ^{1}H NMR (400 MHz, CDCl$_3$) spectrum of (*S*)-3an

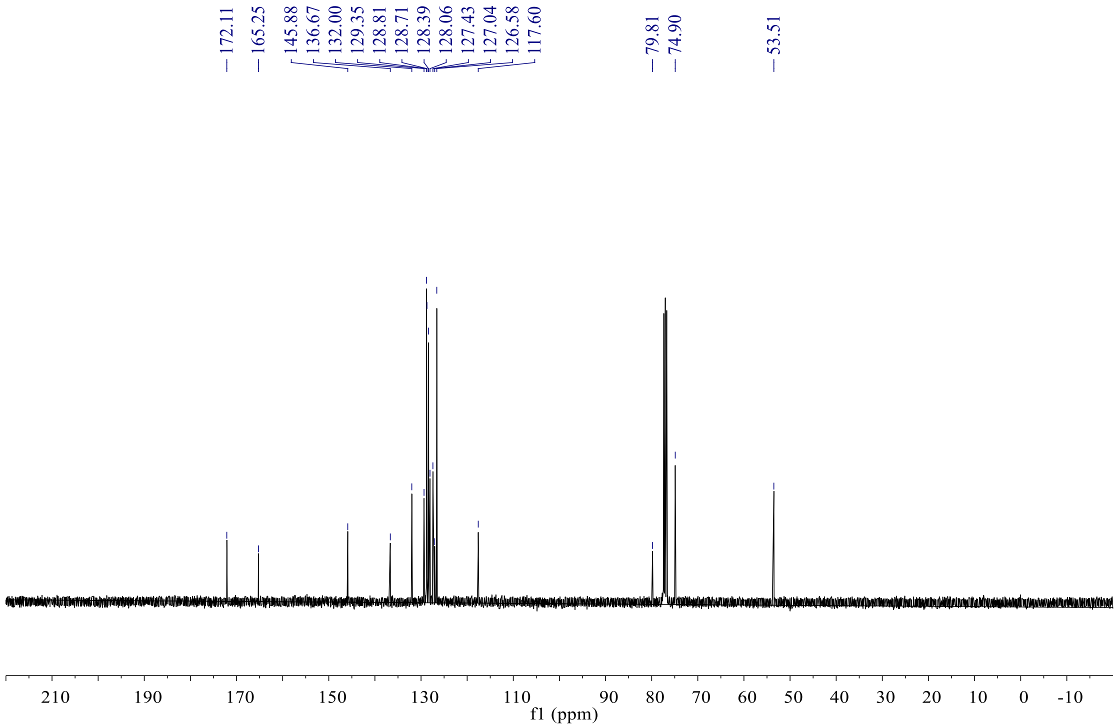

Supplementary Fig. 69 ^{13}C NMR (100 MHz, CDCl$_3$) spectrum of (*S*)-3an

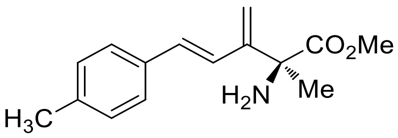

(S)-**3ba**

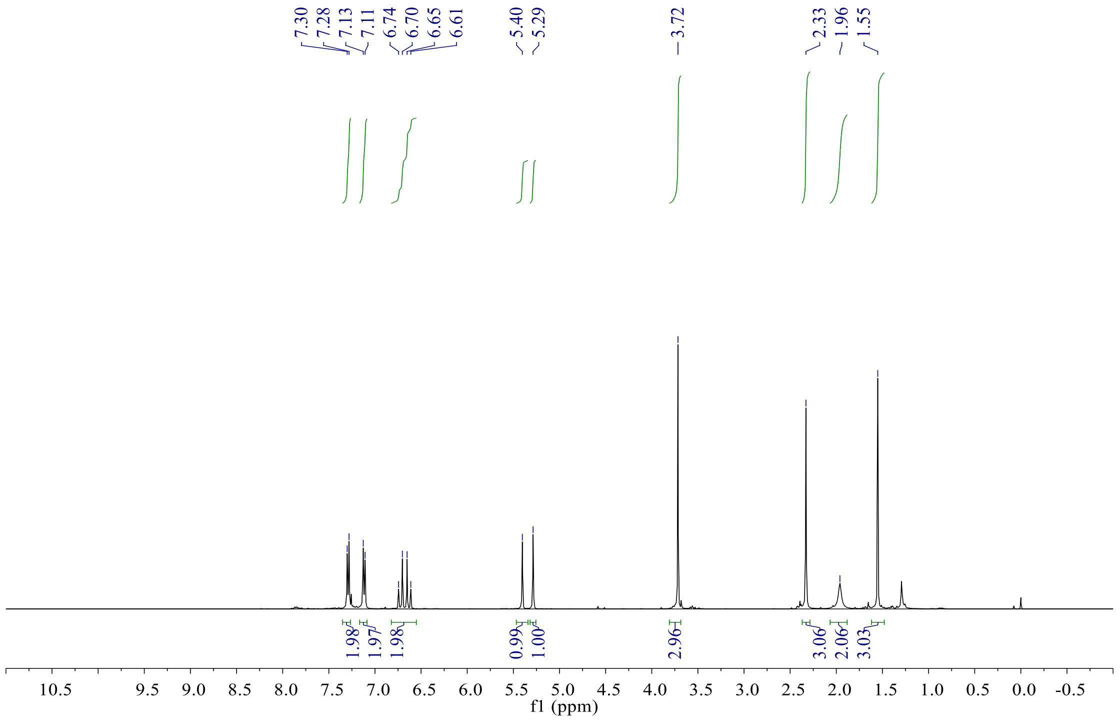

Supplementary Fig. 70 ^{1}H NMR (400 MHz, $CDCl_3$) spectrum of (*S*)-3ba

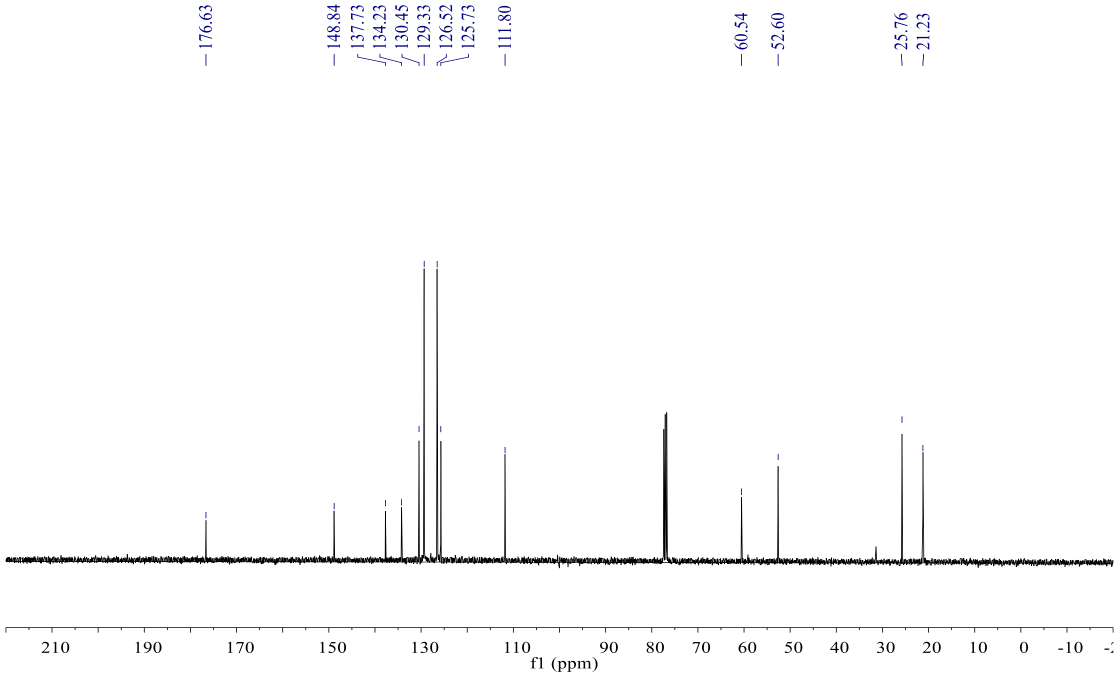

Supplementary Fig. 71 ^{13}C NMR (100 MHz, $CDCl_3$) spectrum of (*S*)-3ba

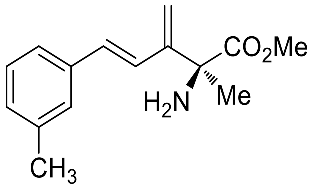

(*S*)-**3ca**

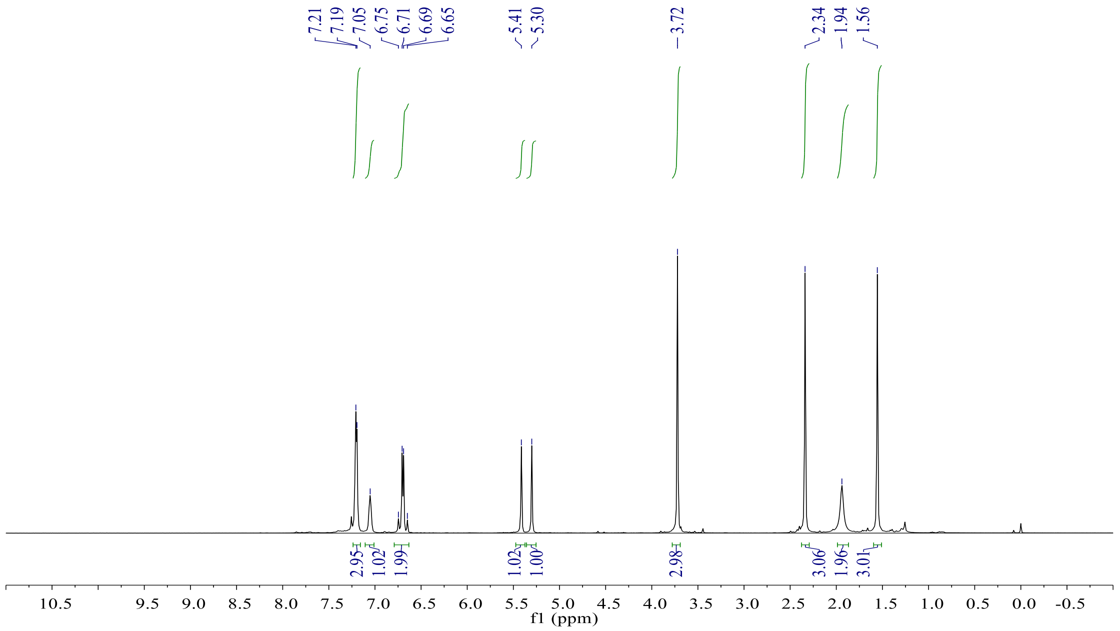

Supplementary Fig. 72 ^{1}H NMR (400 MHz, $CDCl_3$) spectrum of (*S*)-3ca

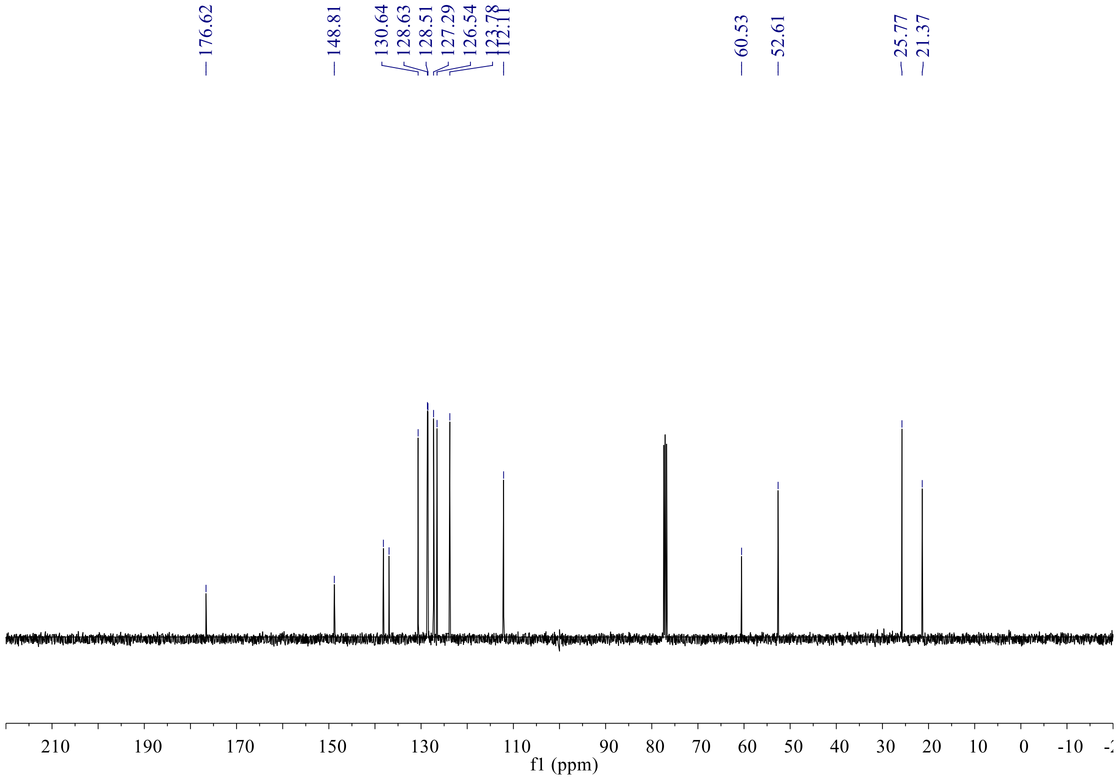

Supplementary Fig. 73 ^{13}C NMR (100 MHz, $CDCl_3$) spectrum of (*S*)-3ca

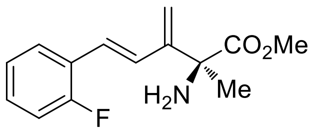

(S)-**3da**

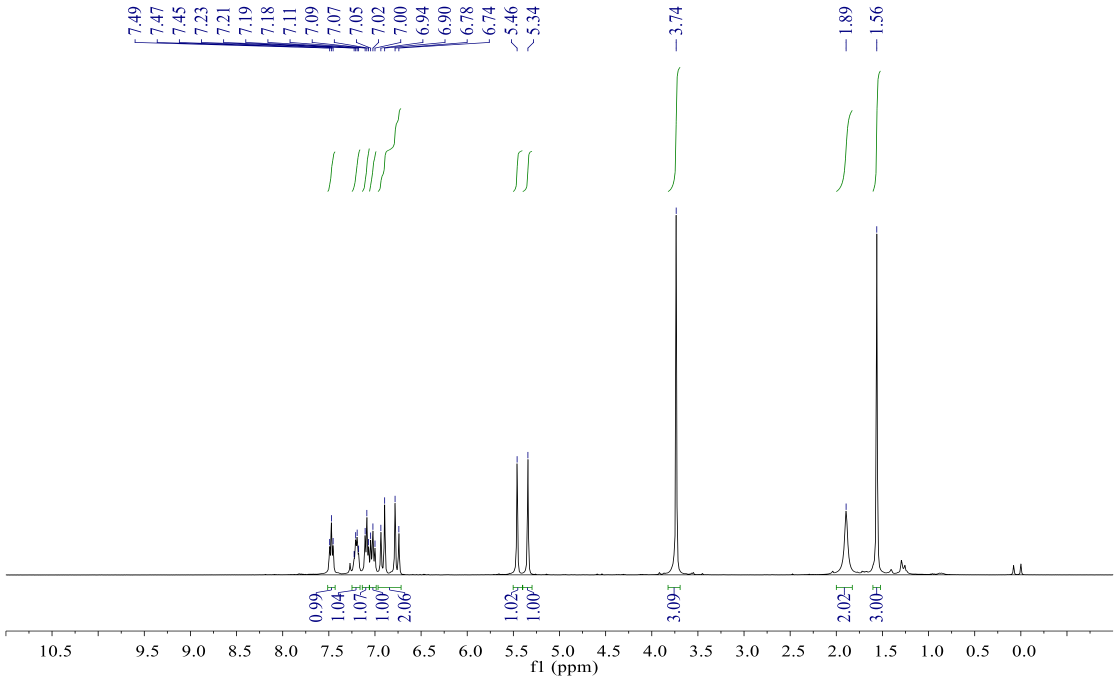

Supplementary Fig. 74 ^{1}H NMR (400 MHz, CDCl$_3$) spectrum of (*S*)-3da

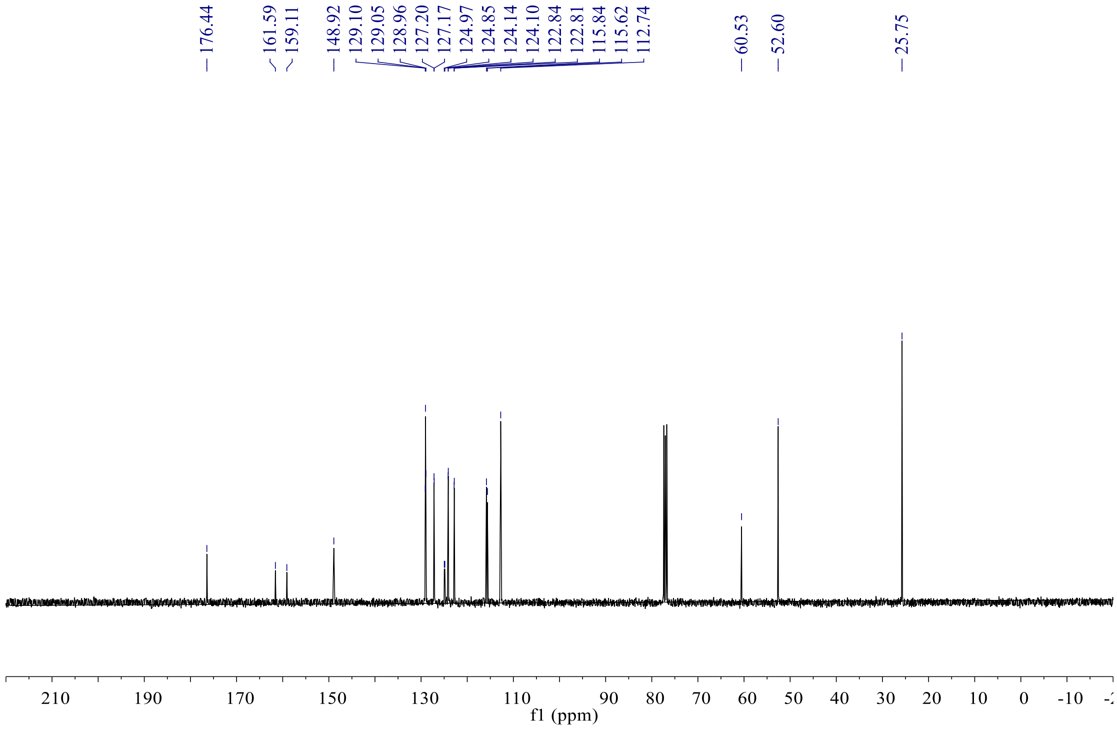

Supplementary Fig. 75 ^{13}C NMR (100 MHz, CDCl$_3$) spectrum of (*S*)-3da

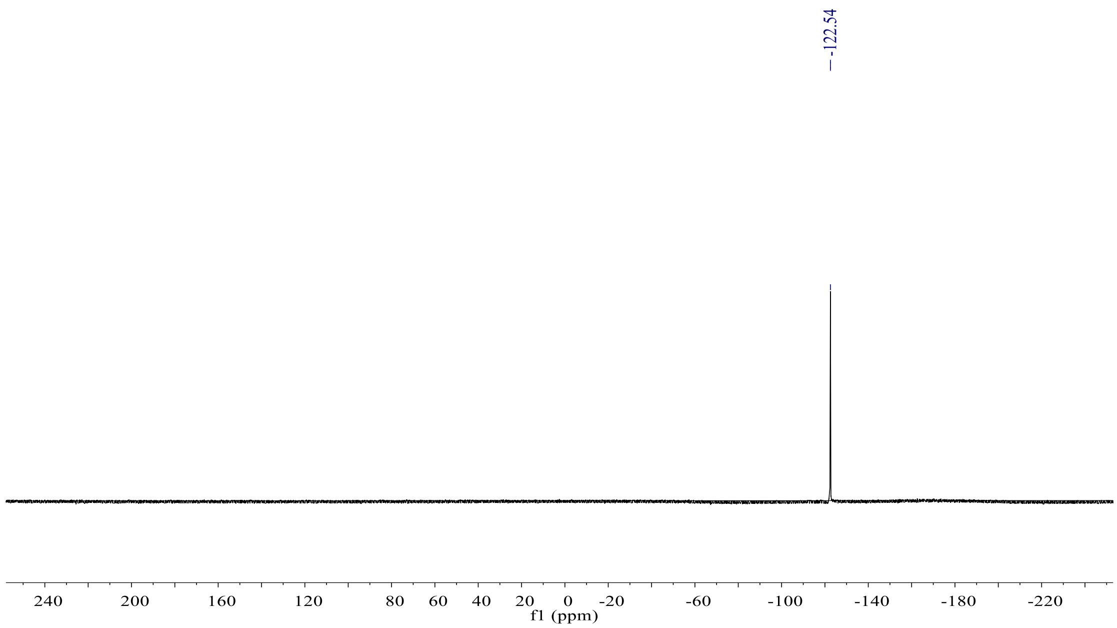

Supplementary Fig. 76 ^{19}C NMR (375 MHz, $CDCl_3$) spectrum of (*S*)-3da

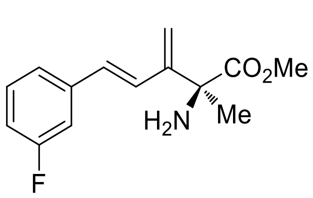

(S)-**3ea**

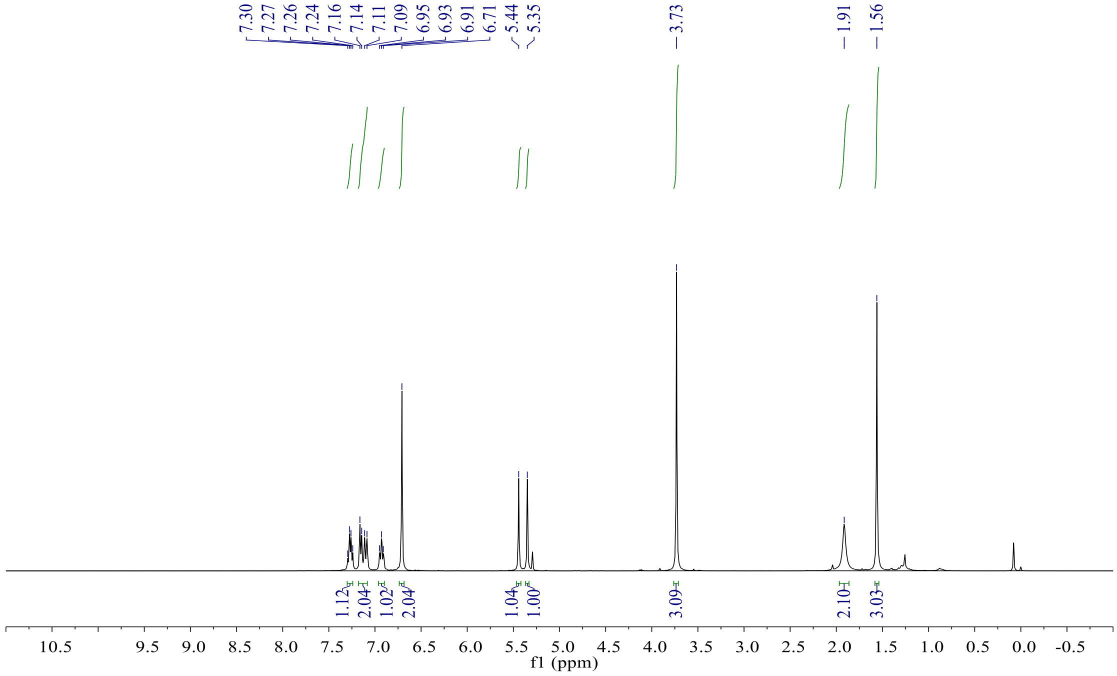

Supplementary Fig. 77 ^{1}H NMR (400 MHz, $CDCl_3$) spectrum of (*S*)-3ea

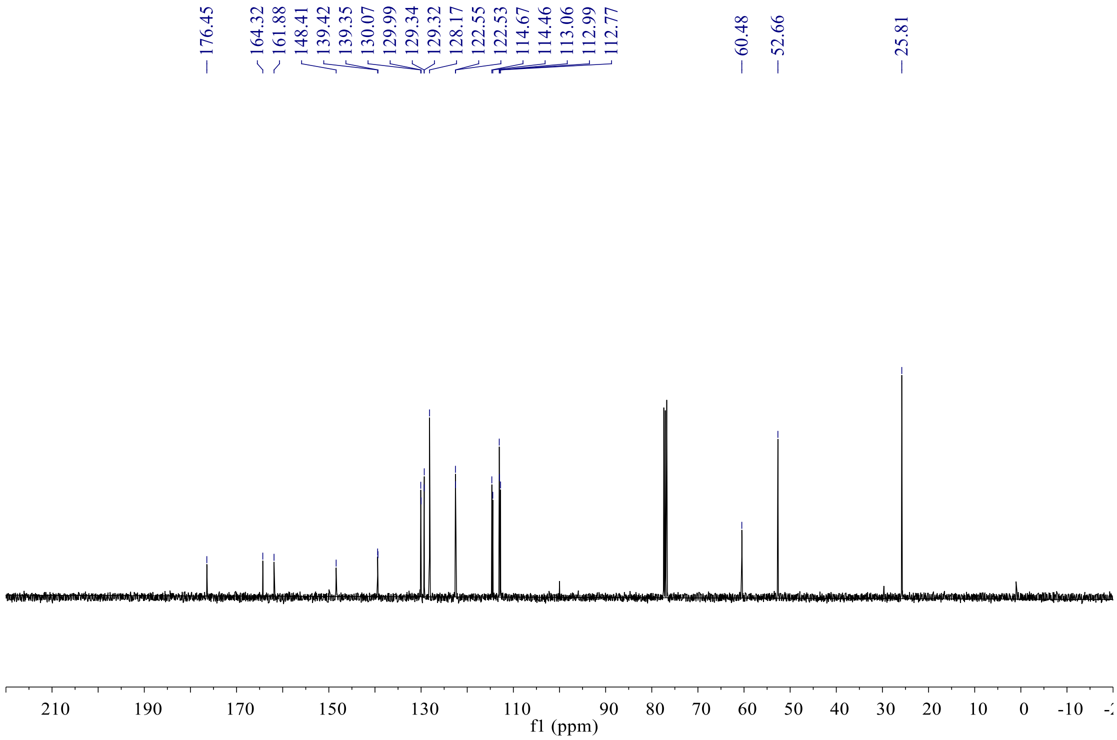

Supplementary Fig. 78 ^{13}C NMR (100 MHz, $CDCl_3$) spectrum of (*S*)-3ea

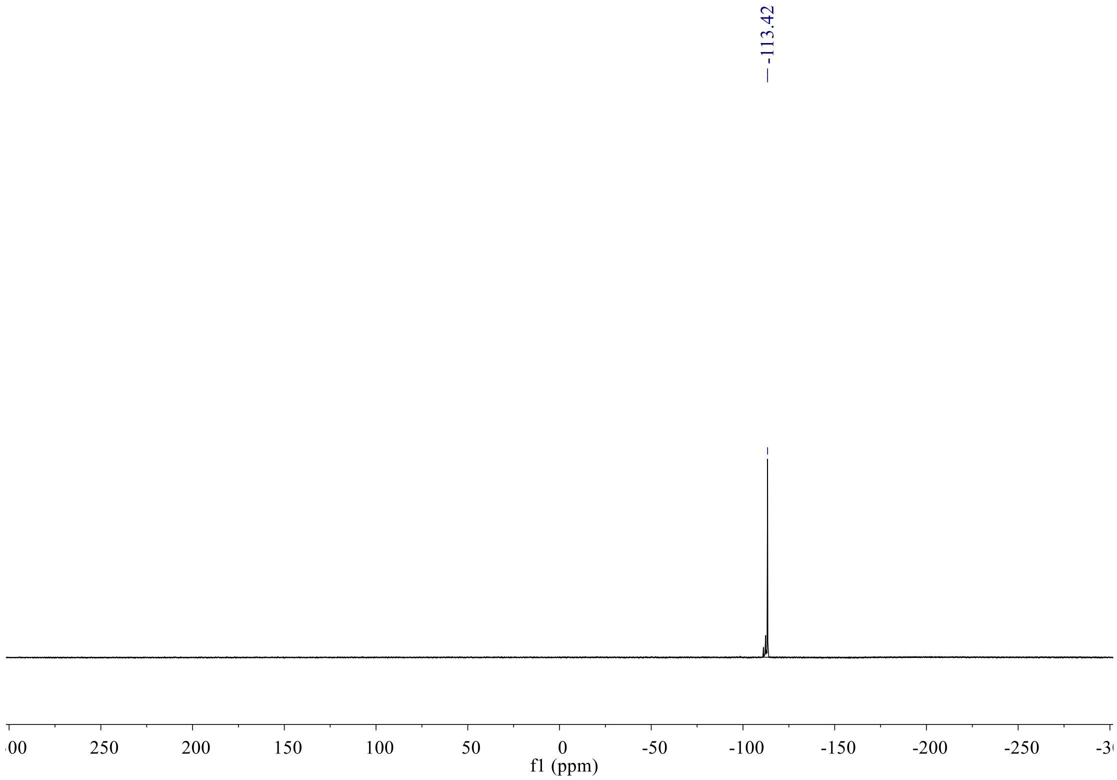

Supplementary Fig. 79 ^{19}F NMR (375 MHz, $CDCl_3$) spectrum of (*S*)-3ea

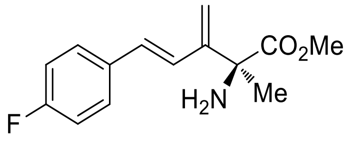

(*S*)-**3fa**

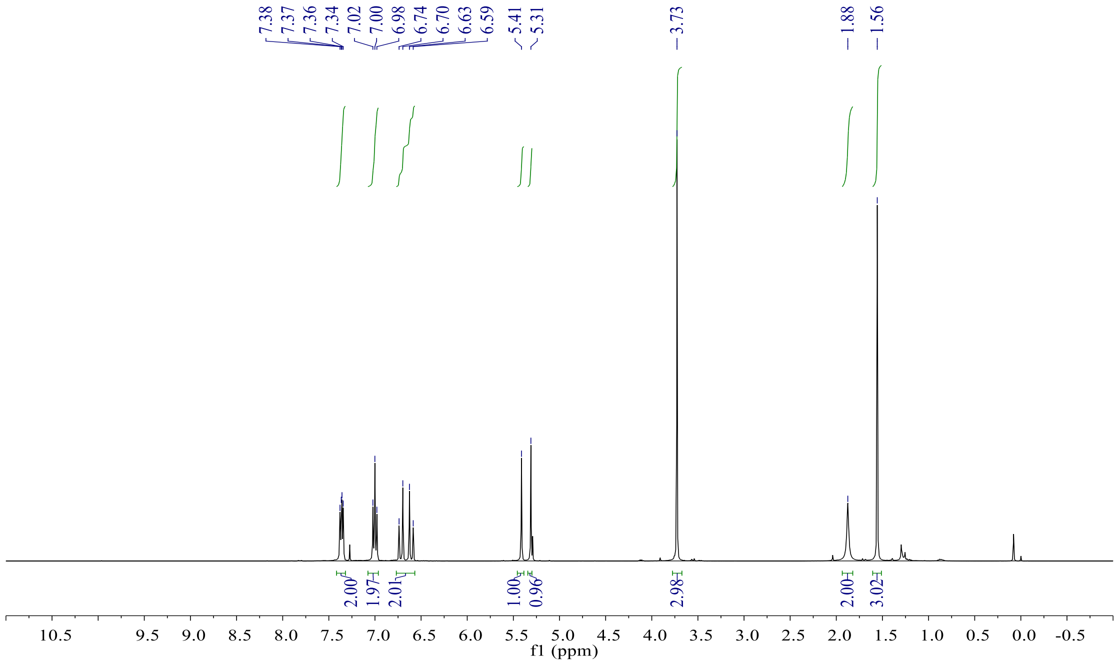

Supplementary Fig. 80 ^{1}H NMR (400 MHz, CDCl$_3$) spectrum of (*S*)-3fa

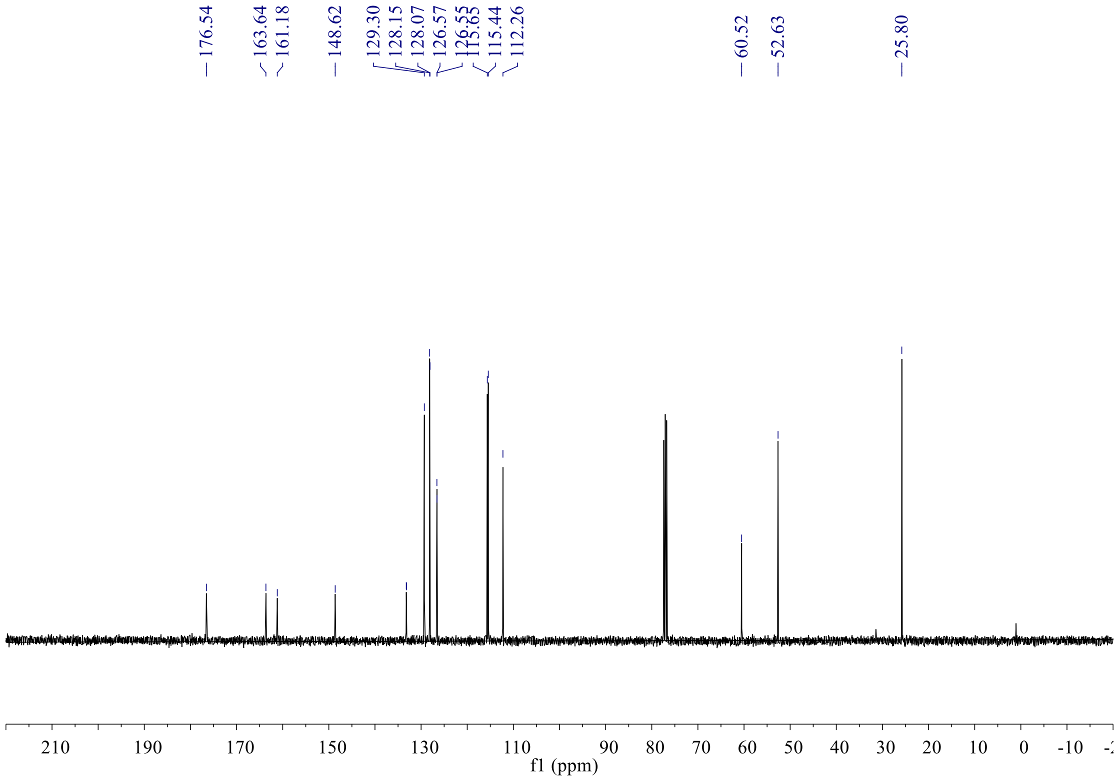

Supplementary Fig. 81 ^{13}C NMR (100 MHz, CDCl$_3$) spectrum of (*S*)-3fa

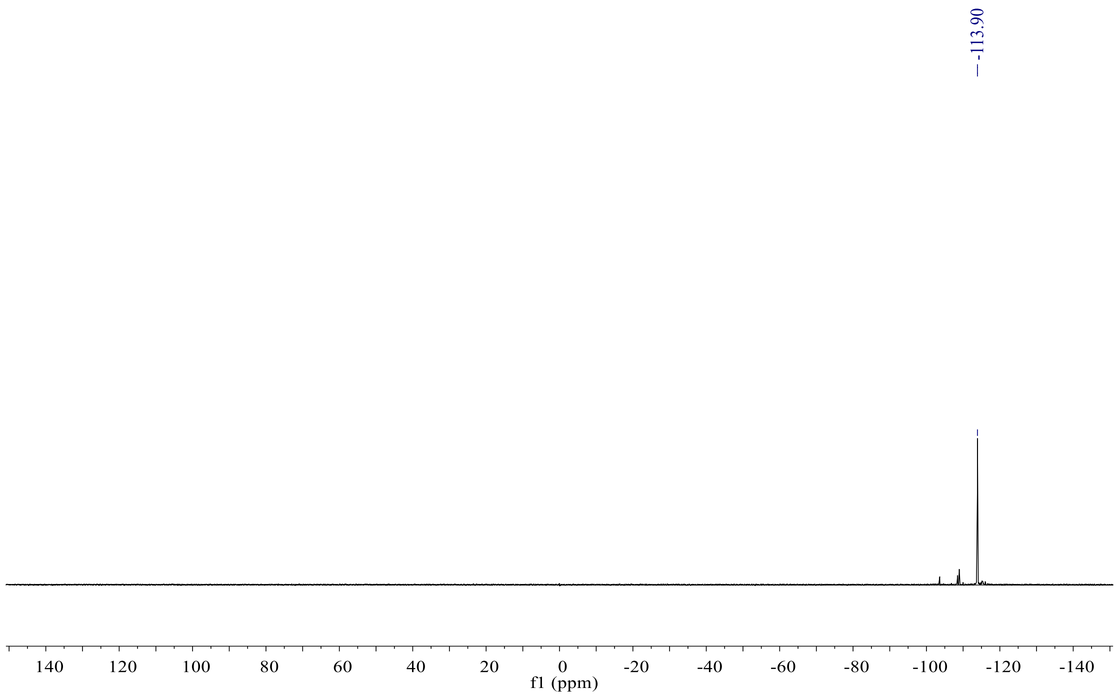

Supplementary Fig. 82 ^{19}F NMR (375 MHz, $CDCl_3$) spectrum of (*S*)-3fa

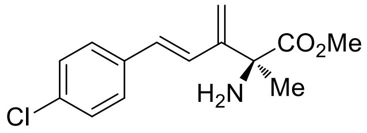

(S)-**3ga**

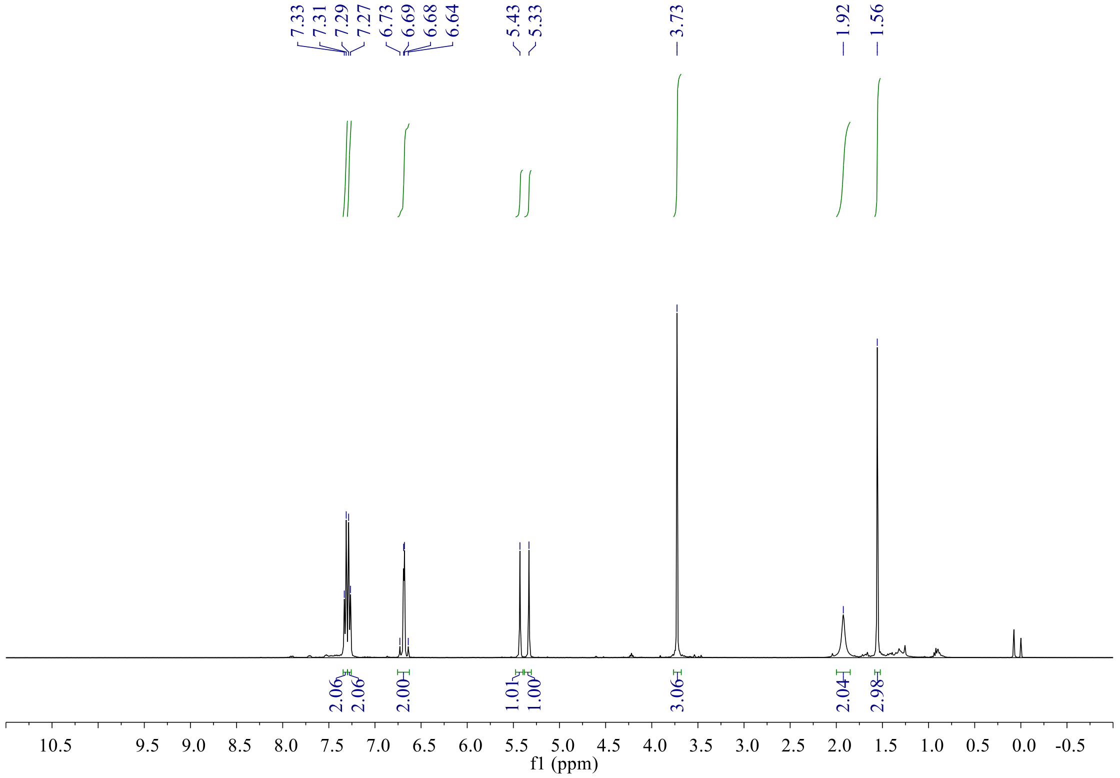

Supplementary Fig. 83 ^{1}H NMR (400 MHz, $CDCl_3$) spectrum of (*S*)-3ga

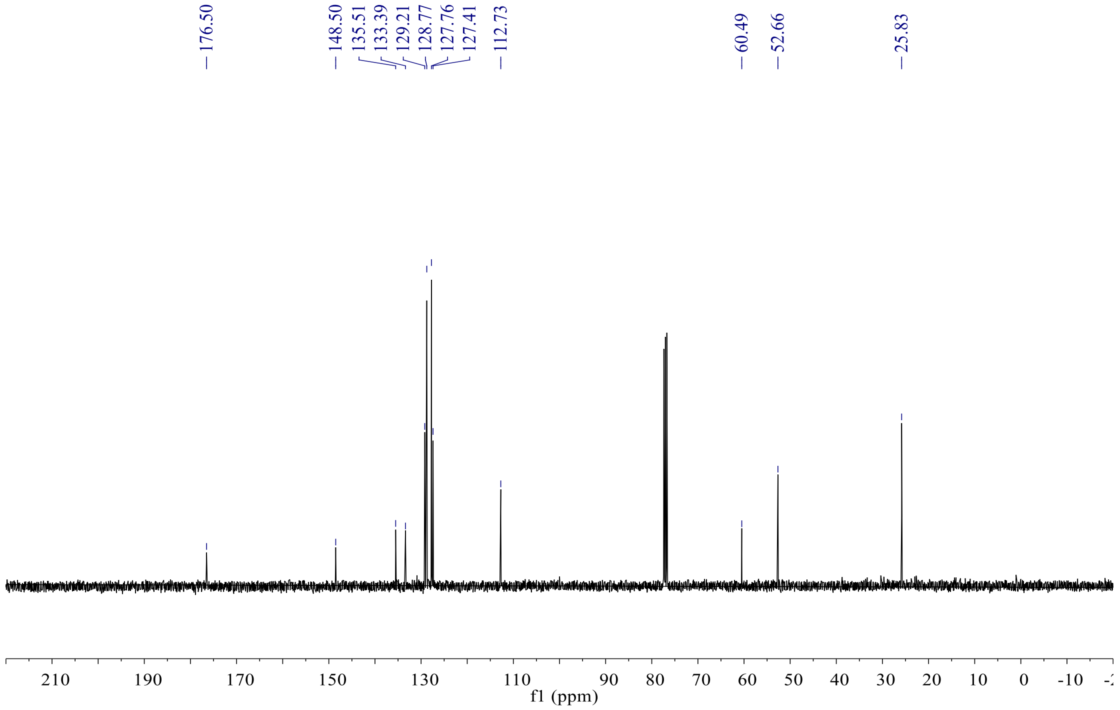

Supplementary Fig. 84 ^{13}C NMR (100 MHz, $CDCl_3$) spectrum of (*S*)-3ga

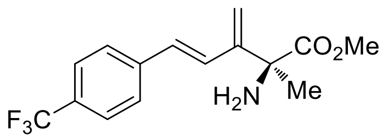

(S)-**3ha**

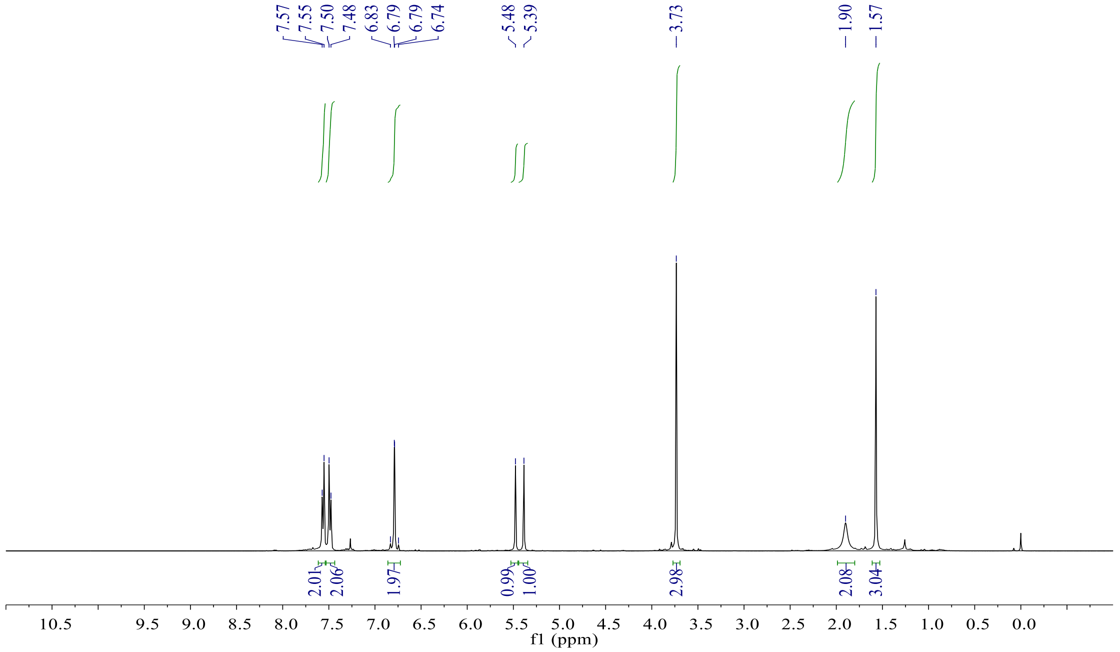

Supplementary Fig. 85 ^{1}H NMR (400 MHz, $CDCl_3$) spectrum of (*S*)-3ha

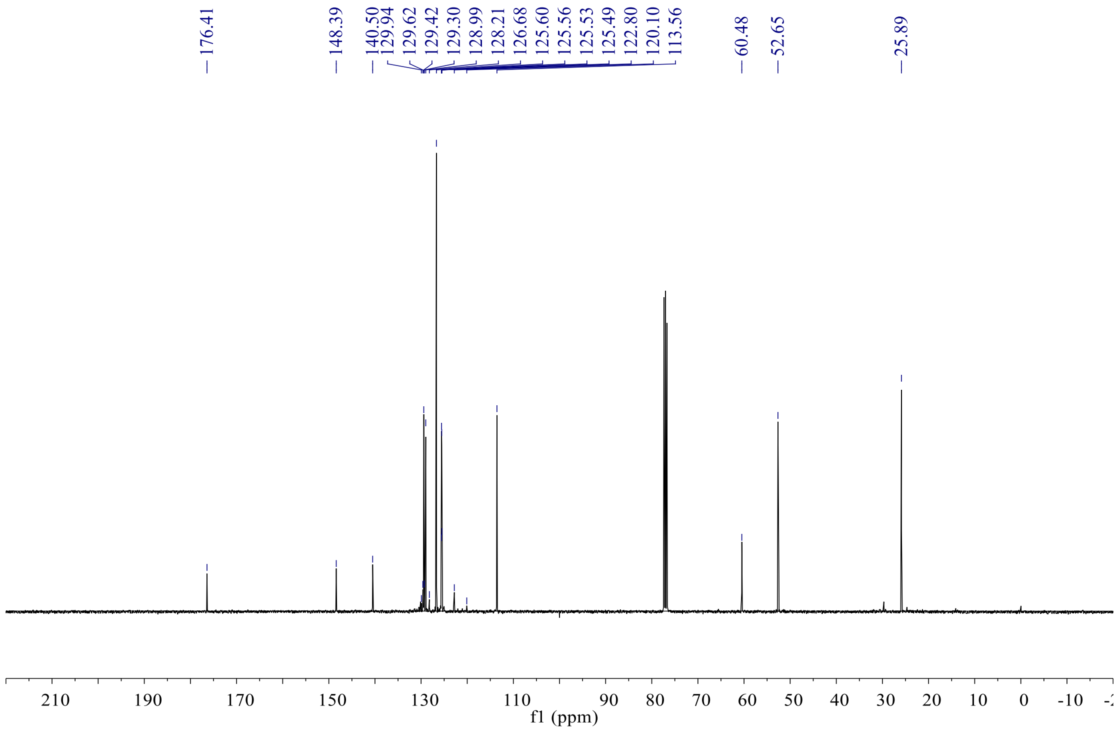

Supplementary Fig. 86 ^{13}C NMR (100 MHz, $CDCl_3$) spectrum of (*S*)-3ha

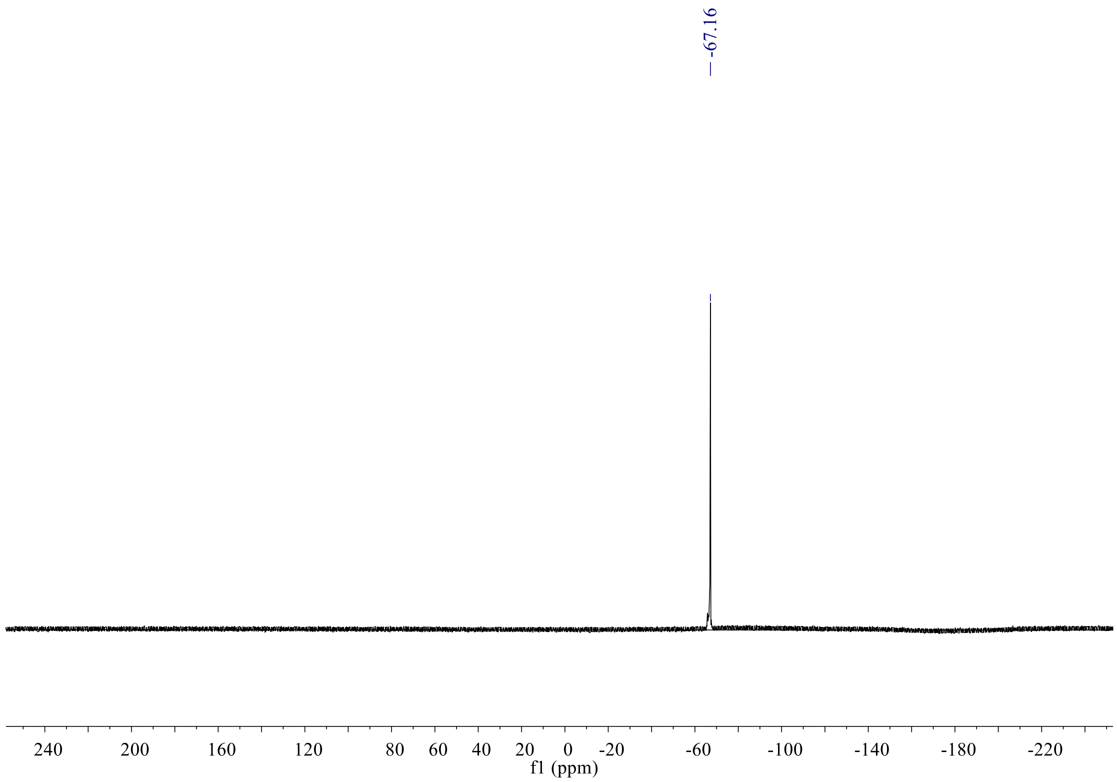

Supplementary Fig. 87 ^{19}F NMR (375 MHz, $CDCl_3$) spectrum of (*S*)-3ha

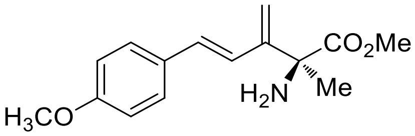

(S)-**3ia**

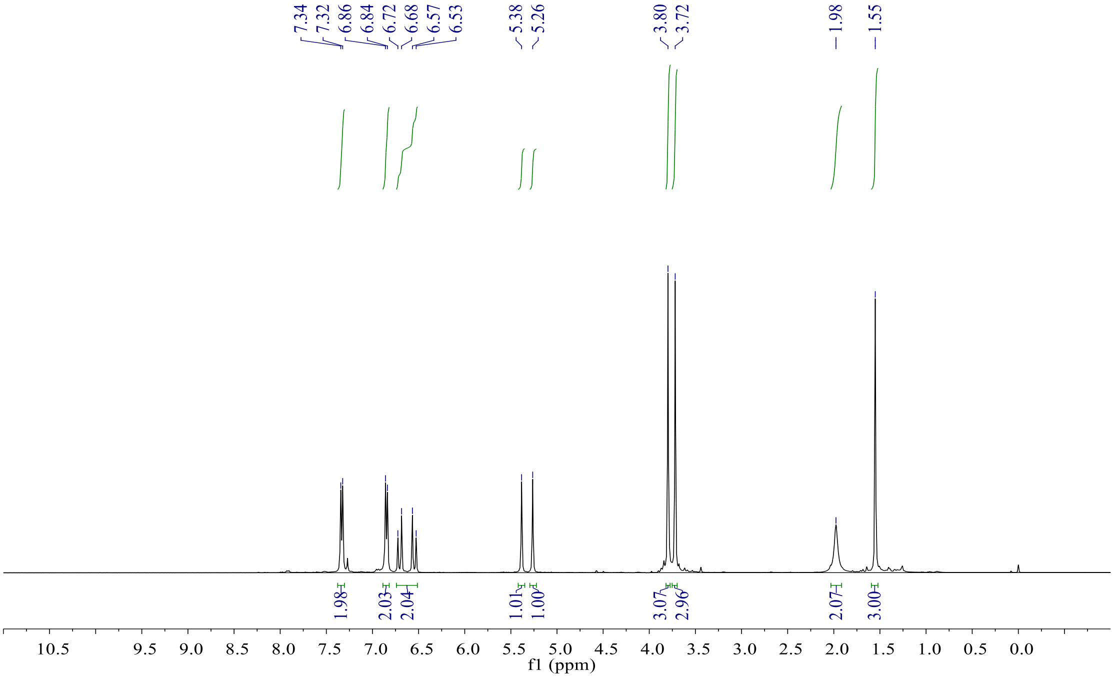

Supplementary Fig. 88 ^{1}H NMR (400 MHz, CDCl$_3$) spectrum of (*S*)-3ia

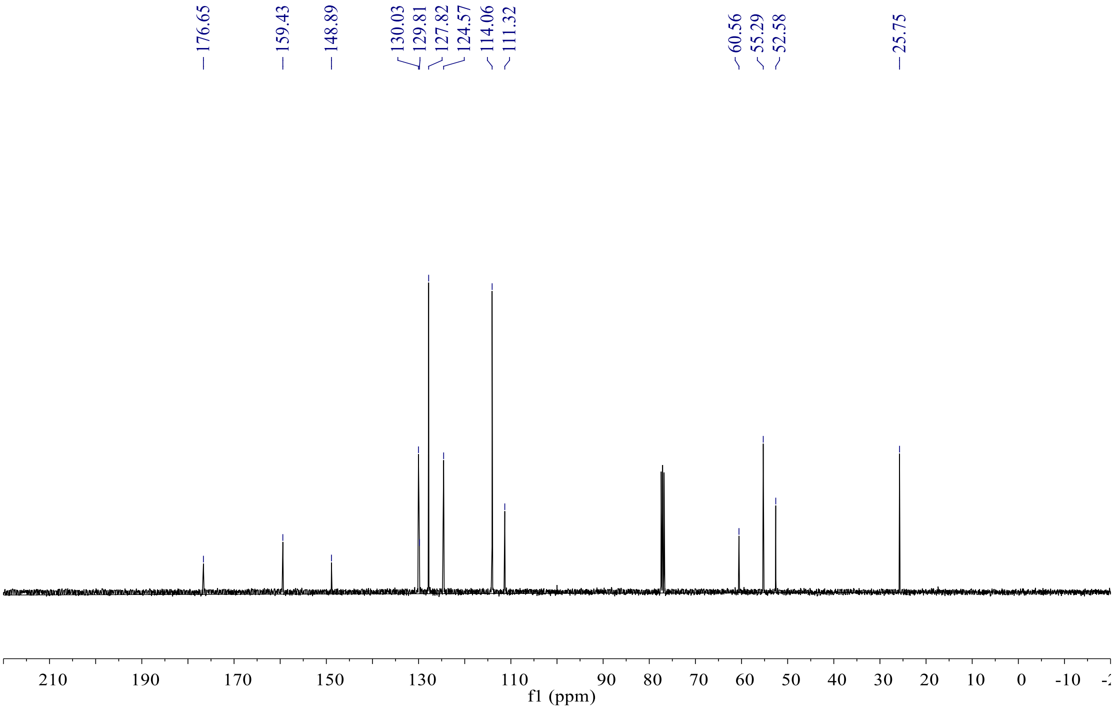

Supplementary Fig. 89 ^{13}C NMR (100 MHz, CDCl$_3$) spectrum of (*S*)-3ia

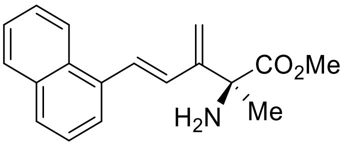

(S)-**3ja**

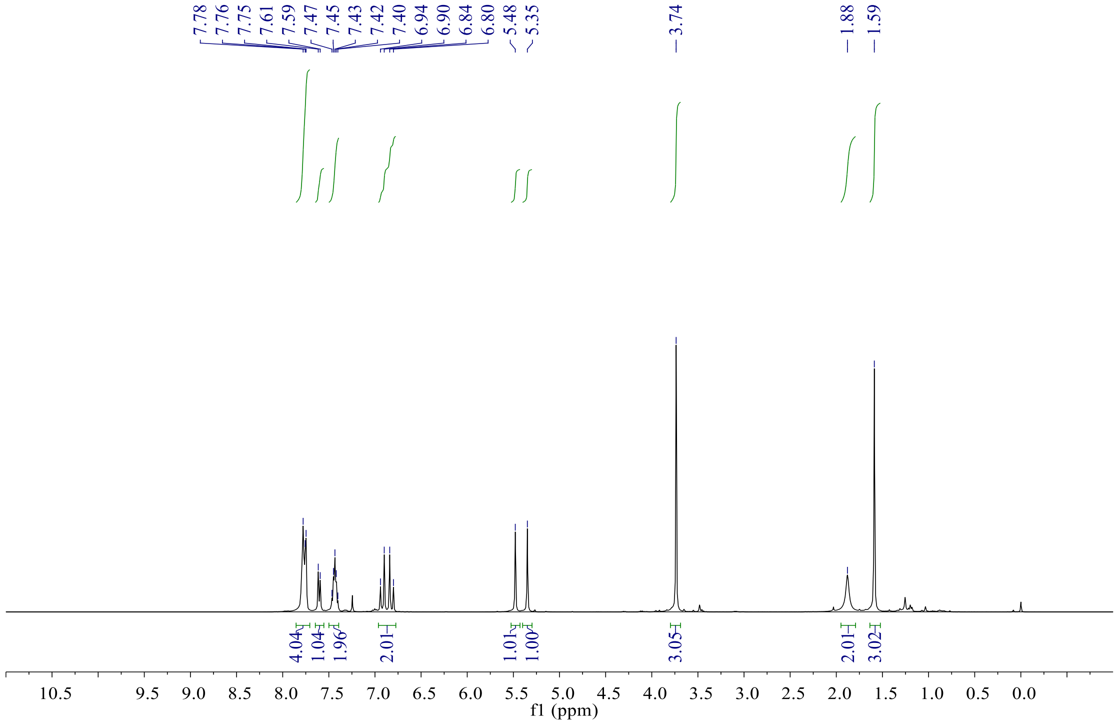

Supplementary Fig. 90 ^{1}H NMR (400 MHz, CDCl$_3$) spectrum of (*S*)-3ja

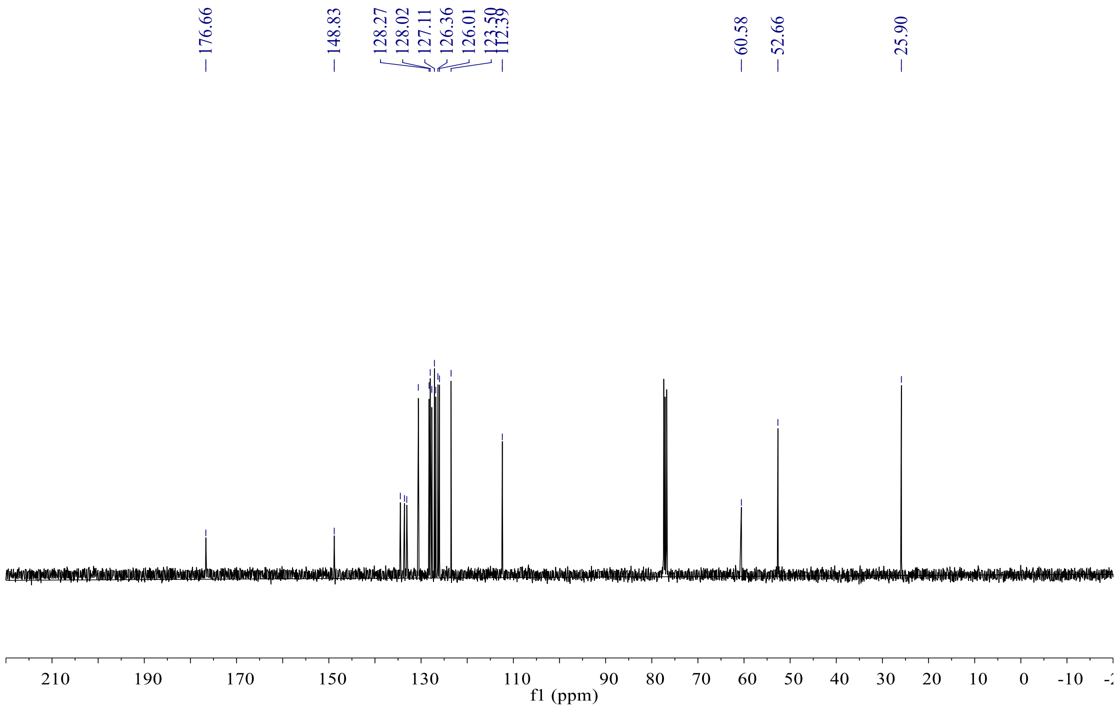

Supplementary Fig. 91 ^{13}C NMR (100 MHz, CDCl$_3$) spectrum of (*S*)-3ja

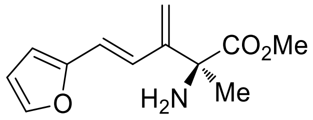

(S)-**3ka**

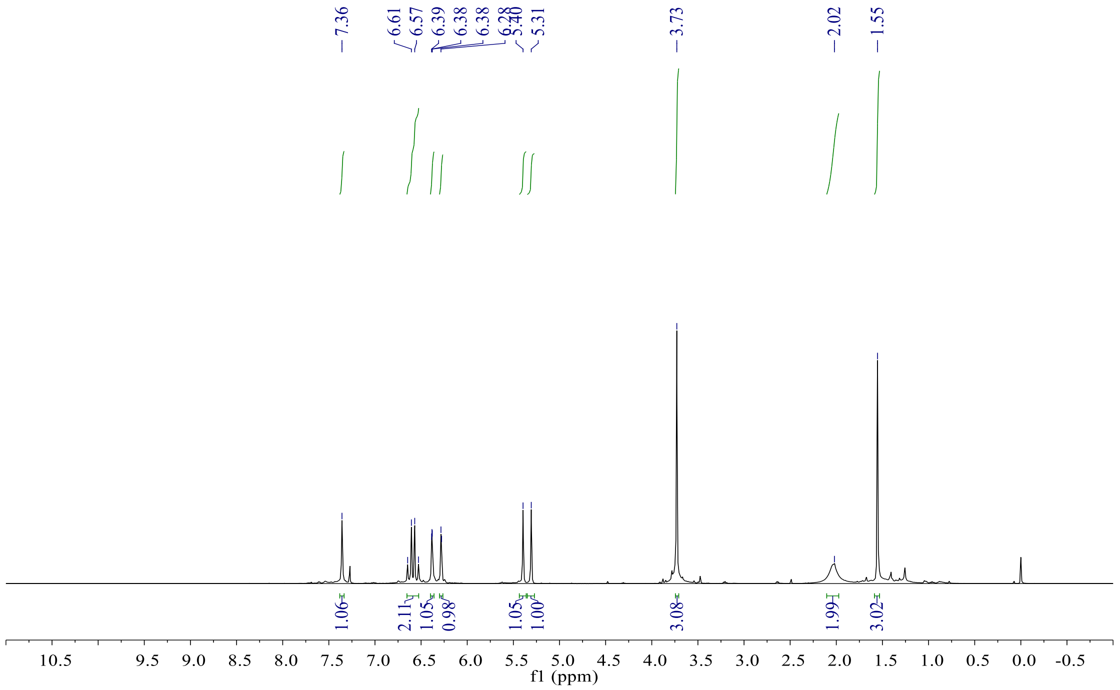

Supplementary Fig. 92 ^{1}H NMR (400 MHz, CDCl$_3$) spectrum of (*S*)-3ka

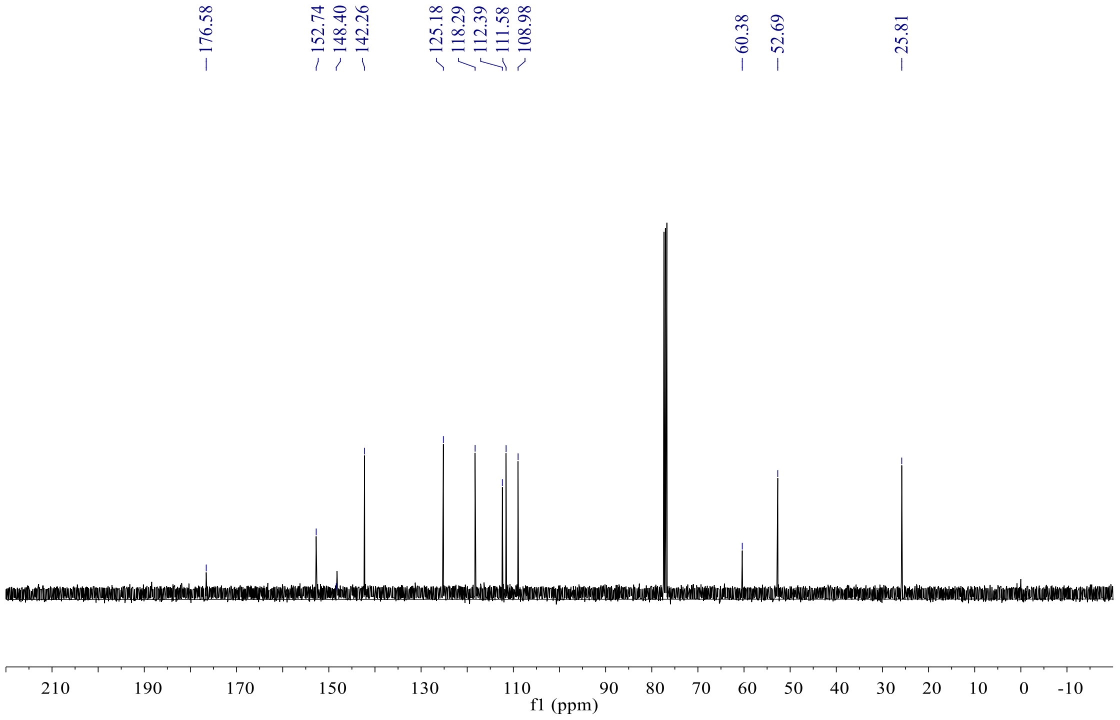

Supplementary Fig. 93 ^{13}C NMR (100 MHz, CDCl$_3$) spectrum of (*S*)-3ka

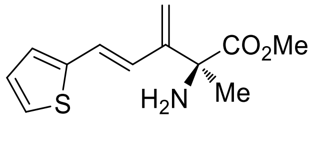

(S)-**3la**

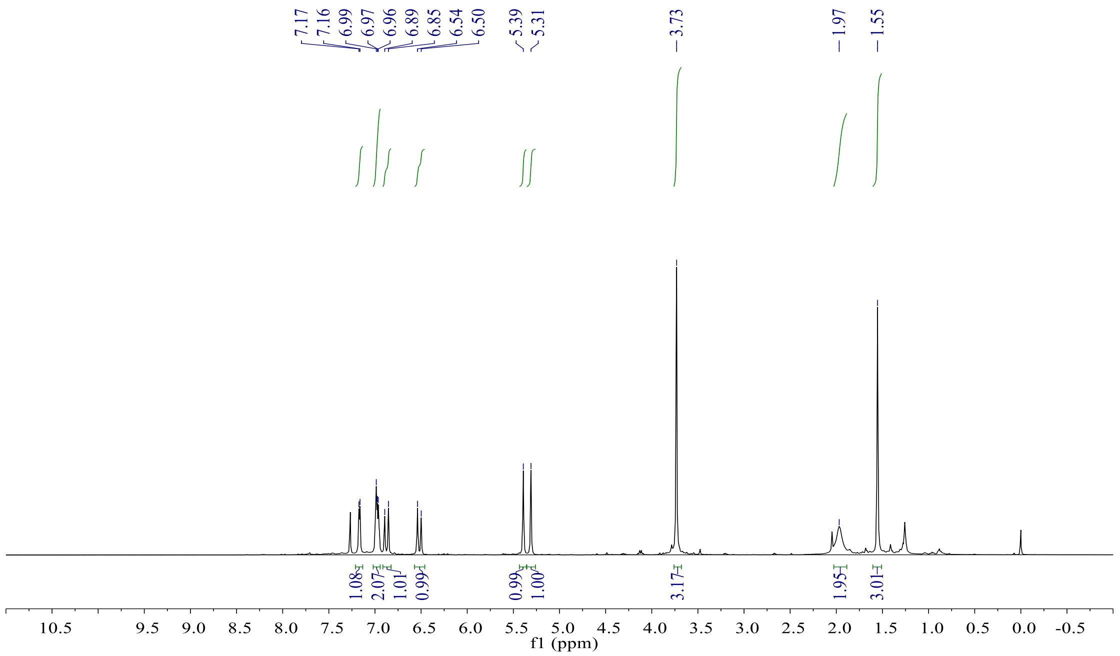

Supplementary Fig. 94 ^{1}H NMR (400 MHz, CDCl$_3$) spectrum of (*S*)-3la

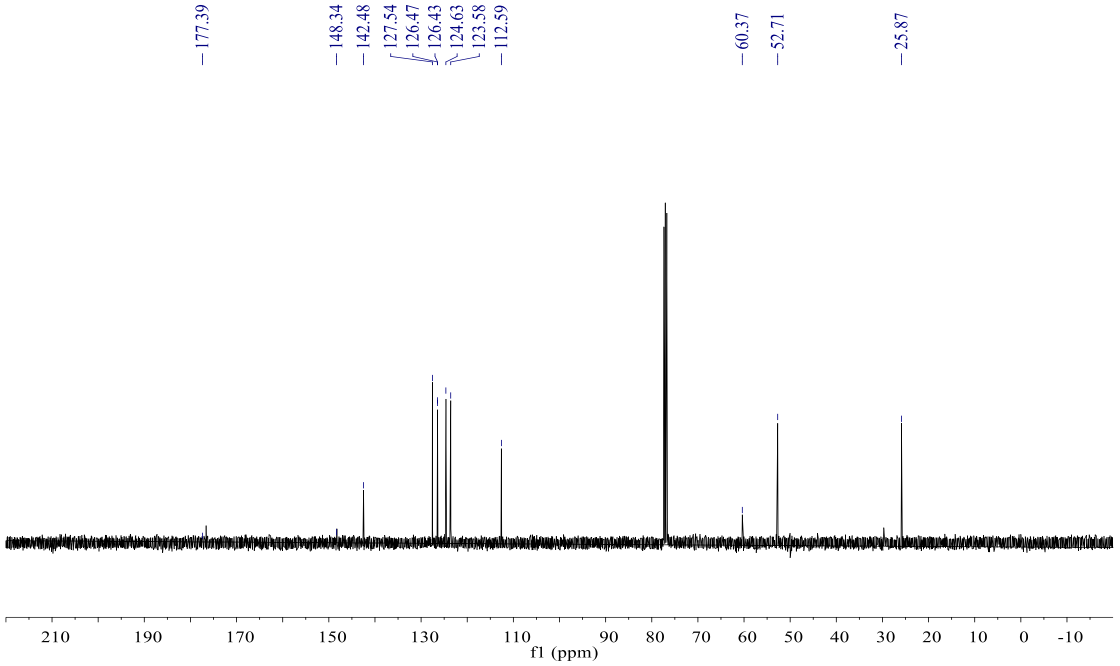

Supplementary Fig. 95 ^{13}C NMR (100 MHz, CDCl$_3$) spectrum of (*S*)-3la

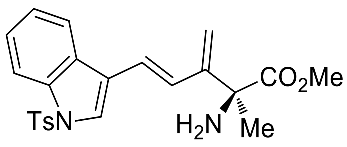

(*S*)-**3ma**

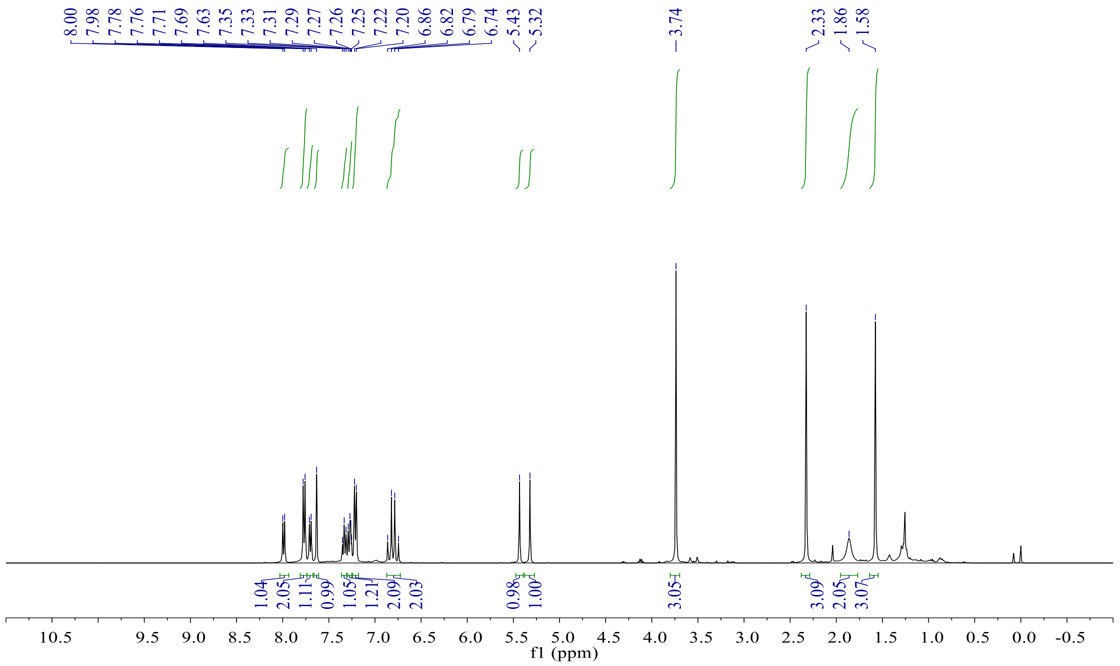

Supplementary Fig. 96 ^{1}H NMR (400 MHz, CDCl$_3$) spectrum of (*S*)-3ma

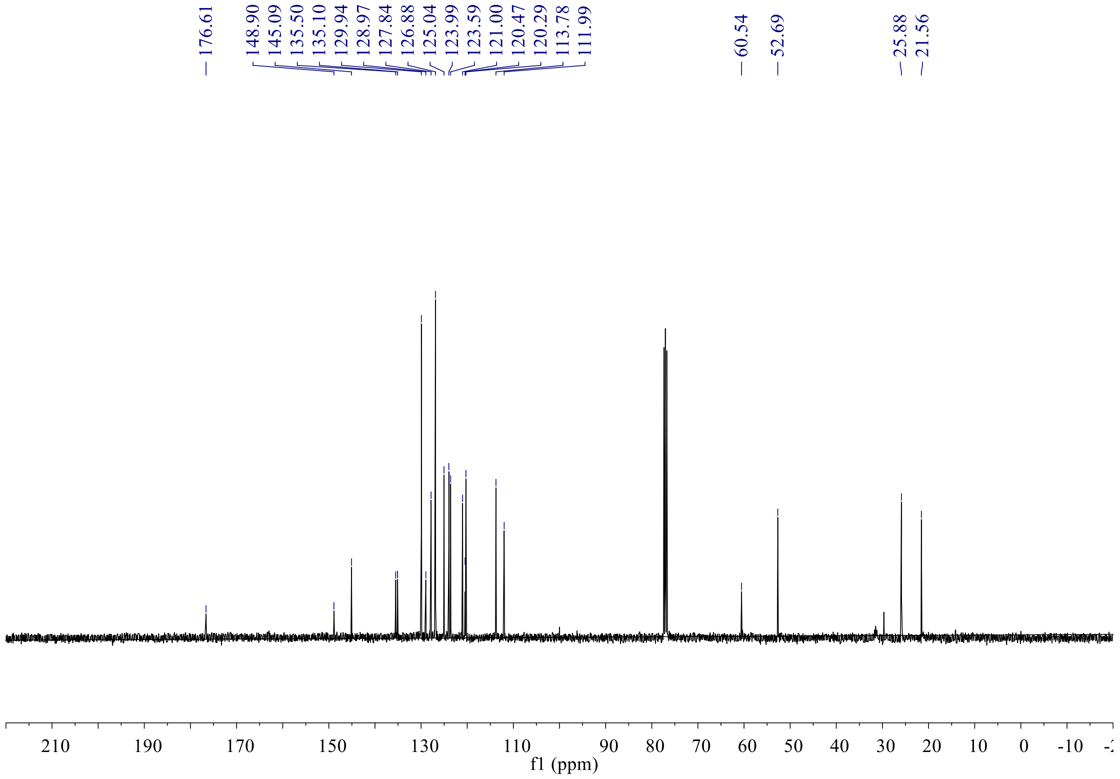

Supplementary Fig. 97 ^{13}C NMR (100 MHz, CDCl$_3$) spectrum of (*S*)-3ma

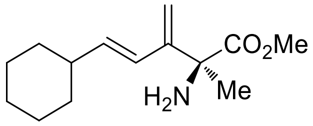

(S)-**3na**

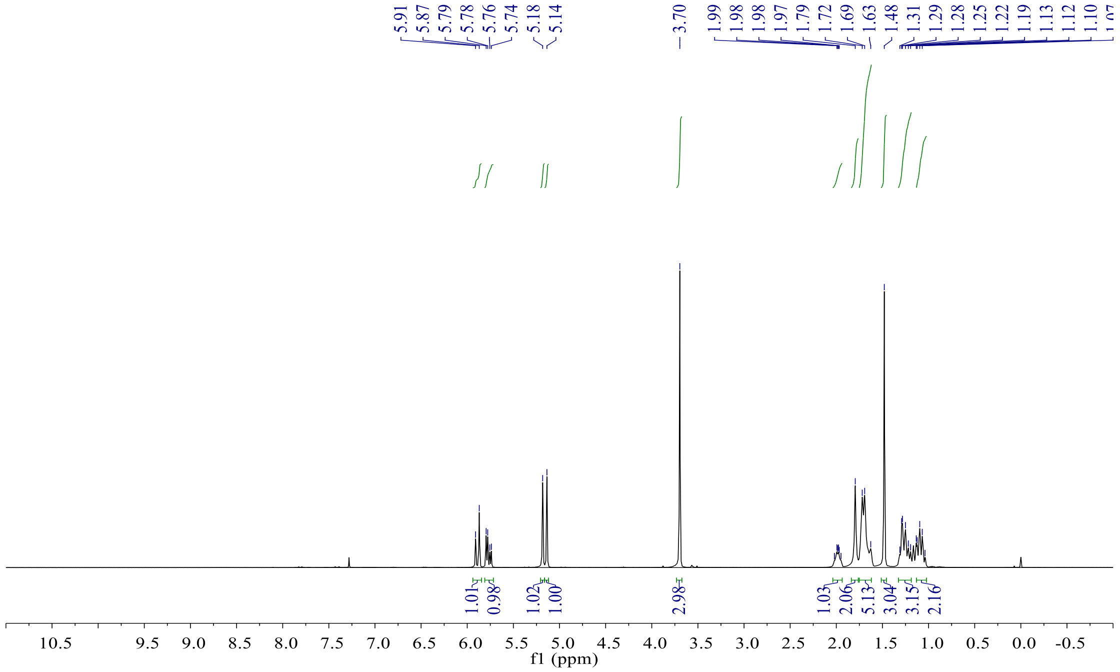

Supplementary Fig. 98 ^{1}H NMR (400 MHz, $CDCl_3$) spectrum of (*S*)-3na

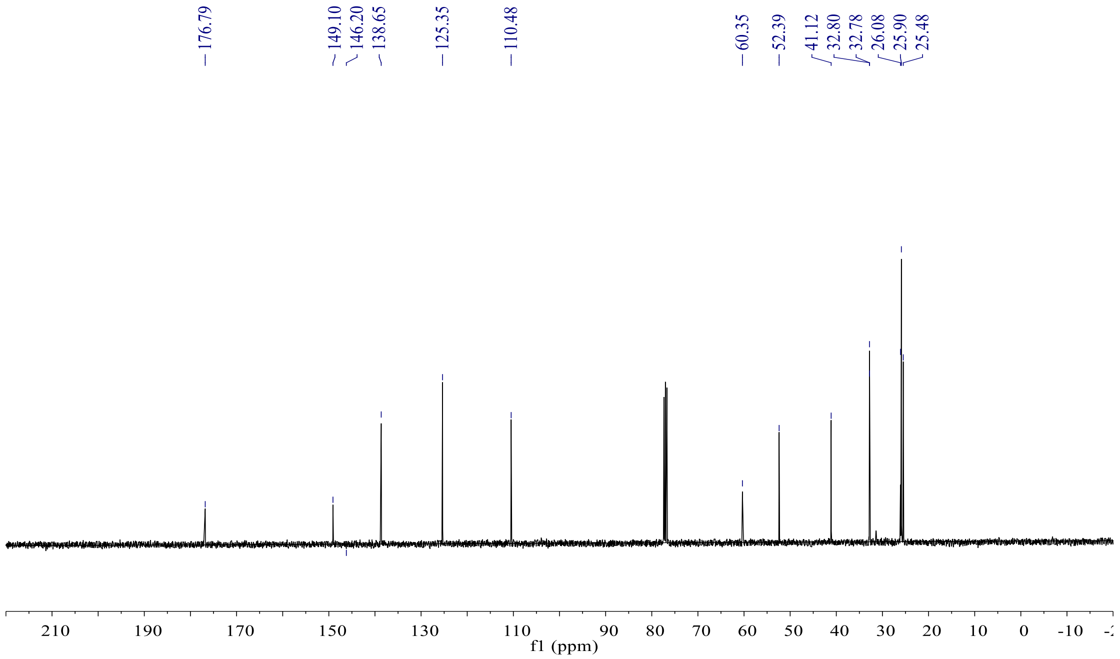

Supplementary Fig. 99 ^{13}C NMR (100 MHz, $CDCl_3$) spectrum of (*S*)-3na

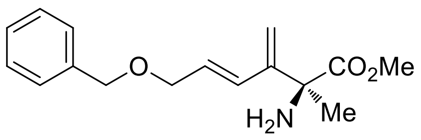

(*S*)-**3oa**

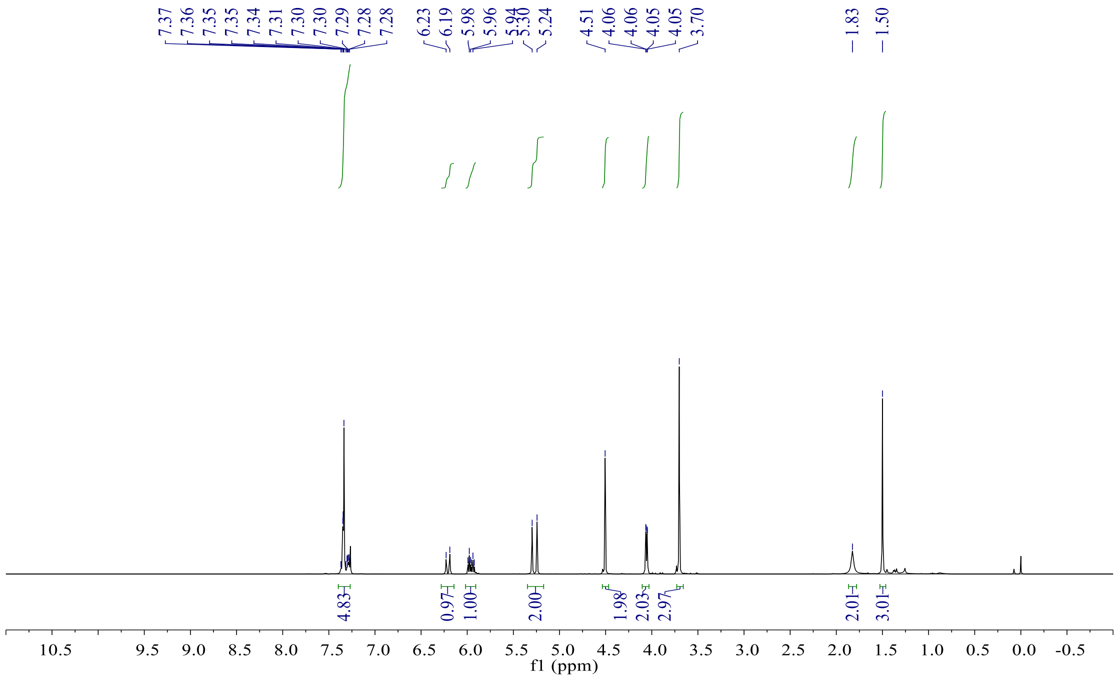

Supplementary Fig. 100 ^{1}H NMR (400 MHz, CDCl$_3$) spectrum of (*S*)-3oa

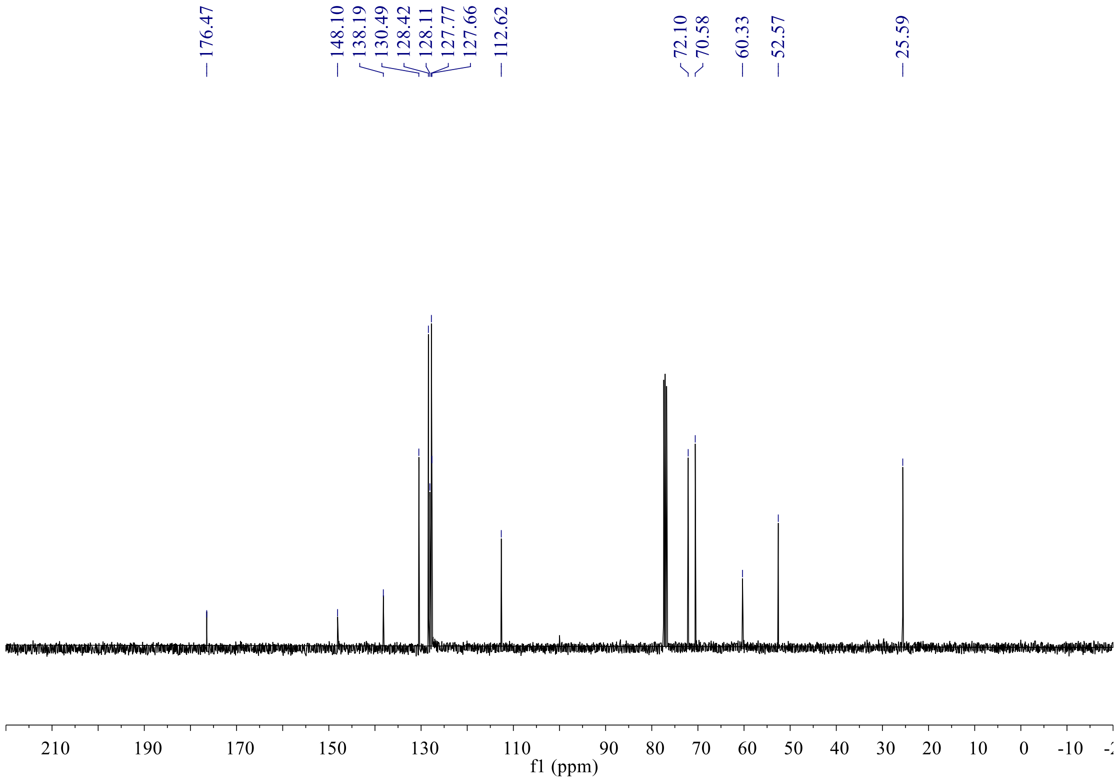

Supplementary Fig. 101 ^{13}C NMR (100 MHz, CDCl$_3$) spectrum of (*S*)-3oa

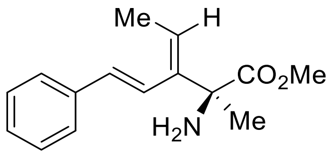

(S)-**3pa**

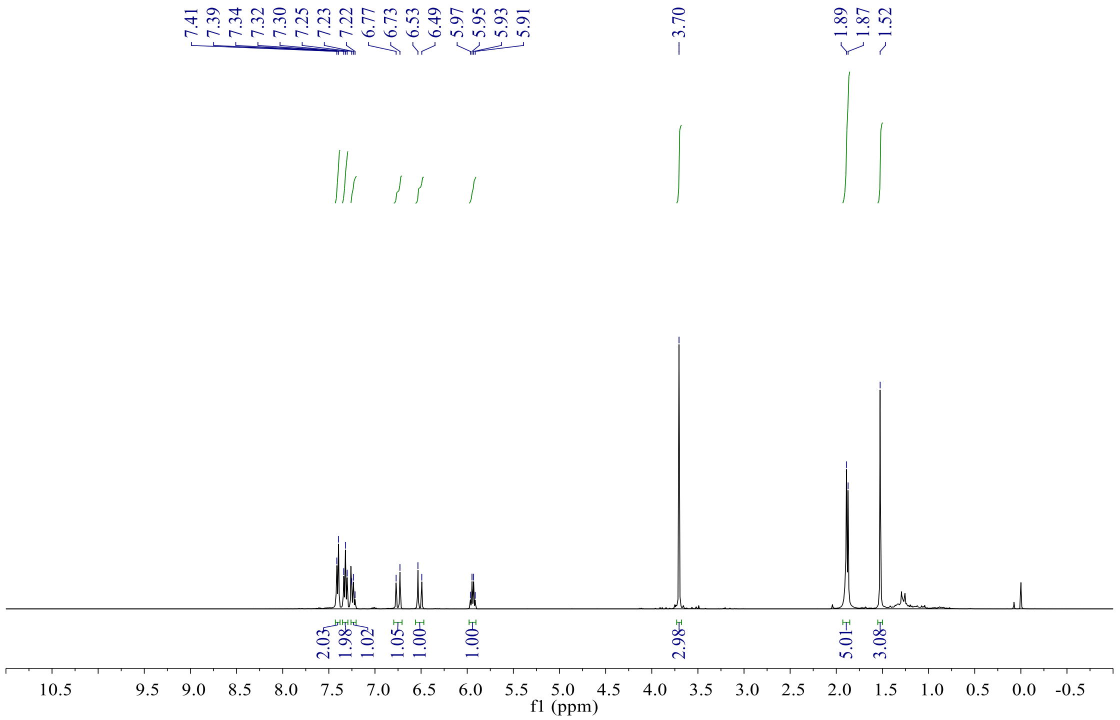

Supplementary Fig. 102 ^{1}H NMR (400 MHz, $CDCl_3$) spectrum of (*S*)-3pa

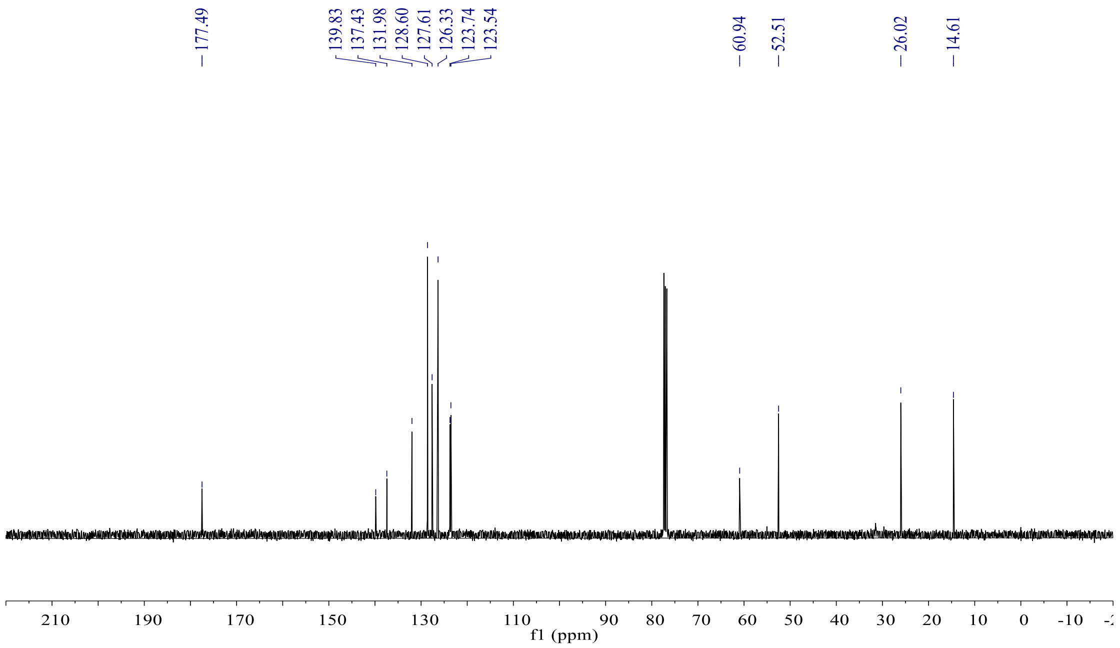

Supplementary Fig. 103 ^{13}C NMR (100 MHz, $CDCl_3$) spectrum of (*S*)-3pa

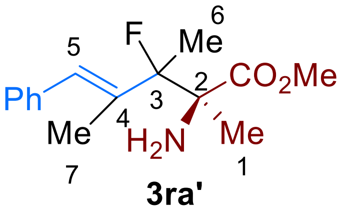

3ra'

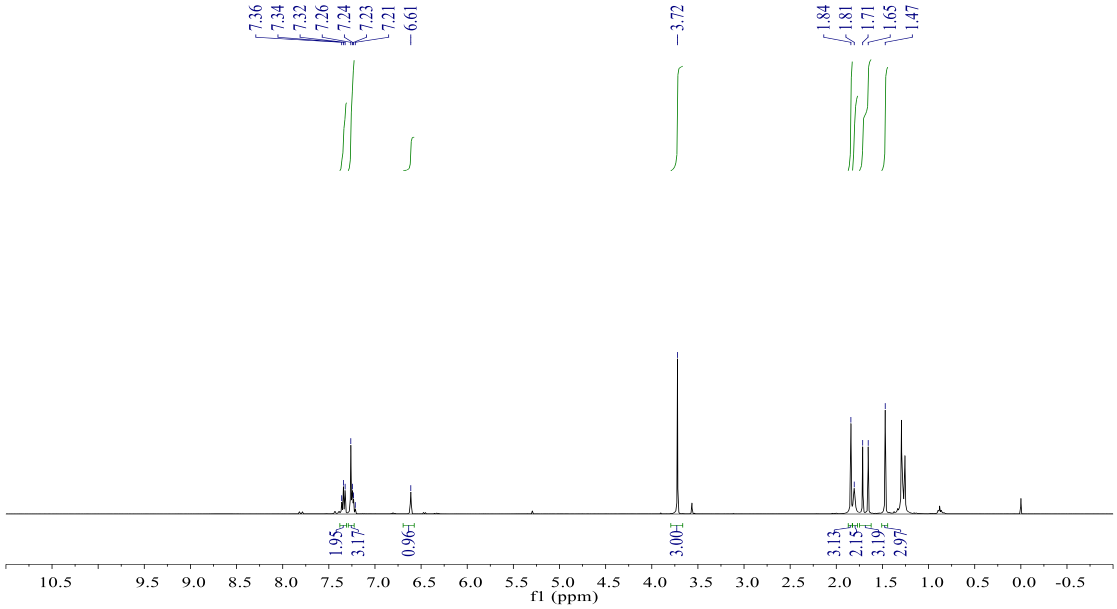

Supplementary Fig. 104 ^{1}H NMR (400 MHz, CDCl₃) spectrum of (*S*)-3ra′

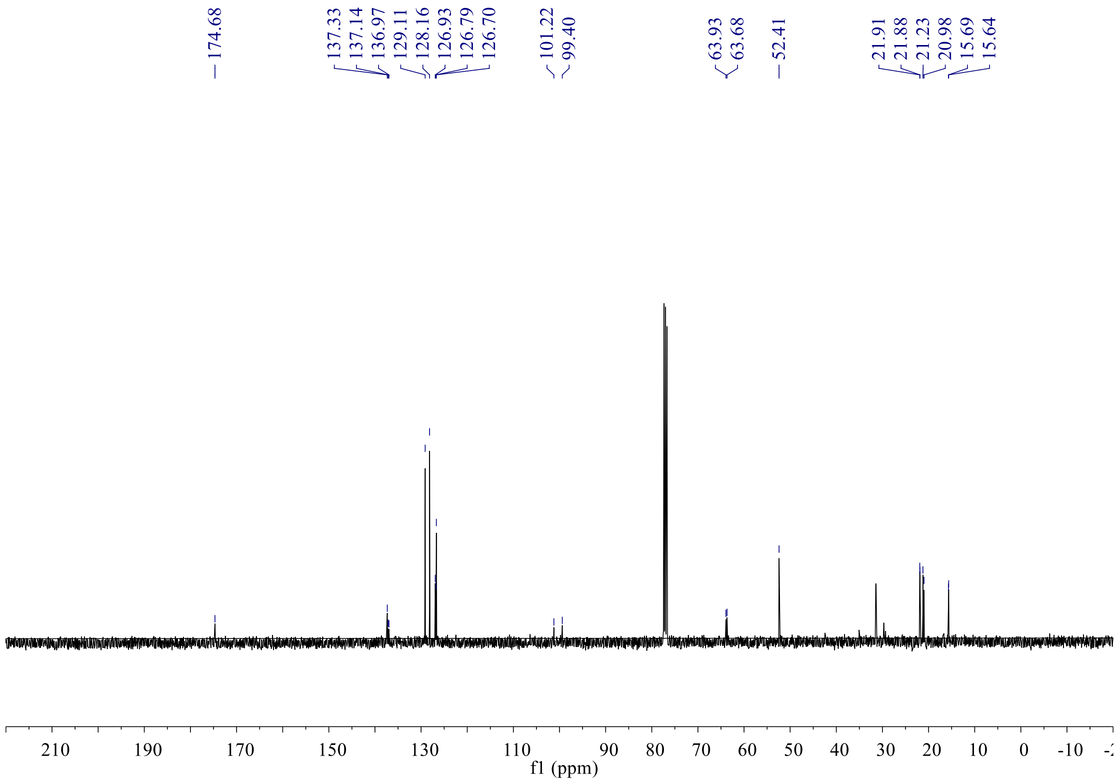

Supplementary Fig. 105 ^{13}C NMR (100 MHz, CDCl₃) spectrum of (*S*)-3ra′

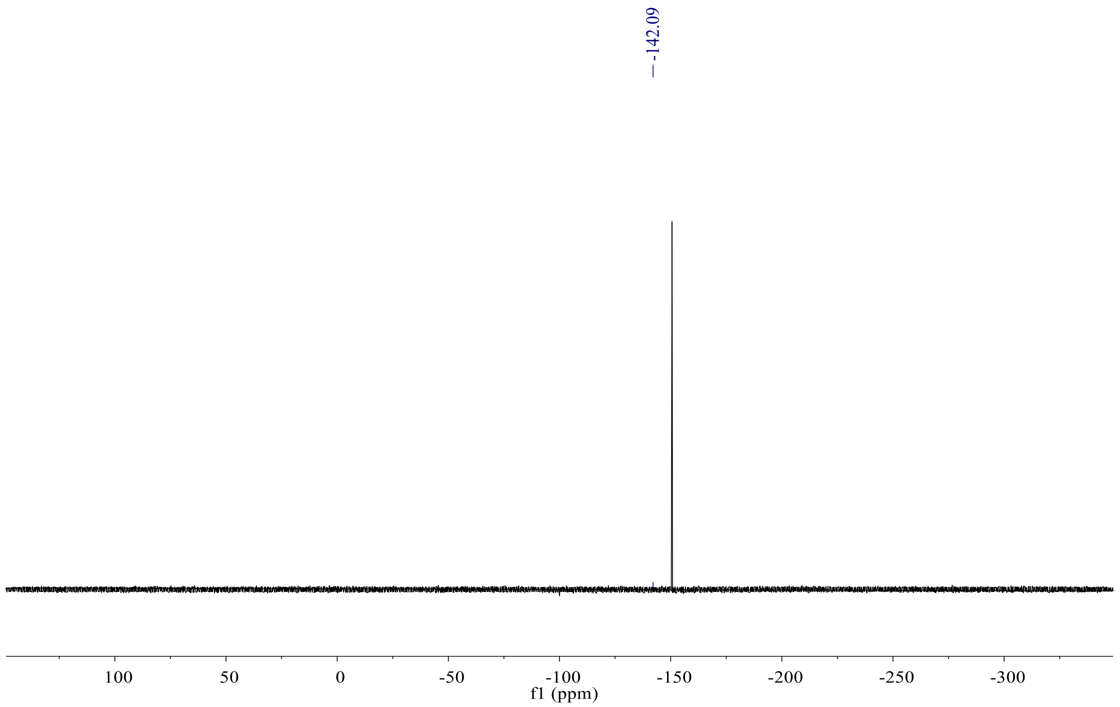

Supplementary Fig. 106 ^{19}F NMR (100 MHz, CDCl$_3$) spectrum of (*S*)-3ra′

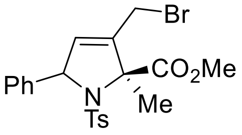

5aa

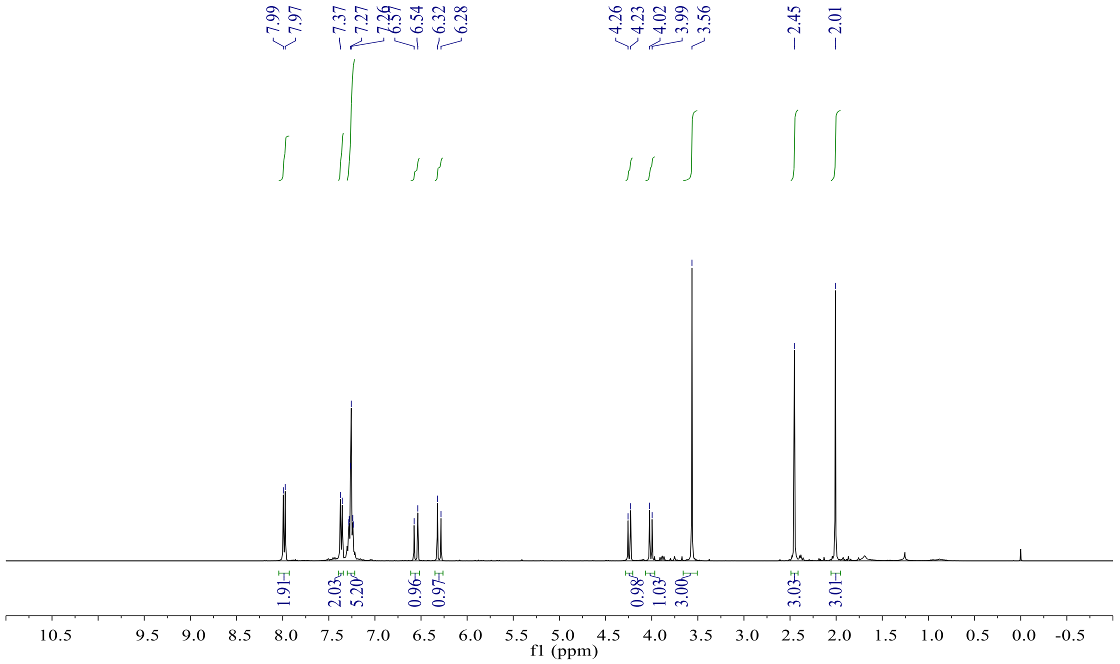

Supplementary Fig. 107 ^{1}H NMR (400 MHz, CDCl$_3$) spectrum of 5aa

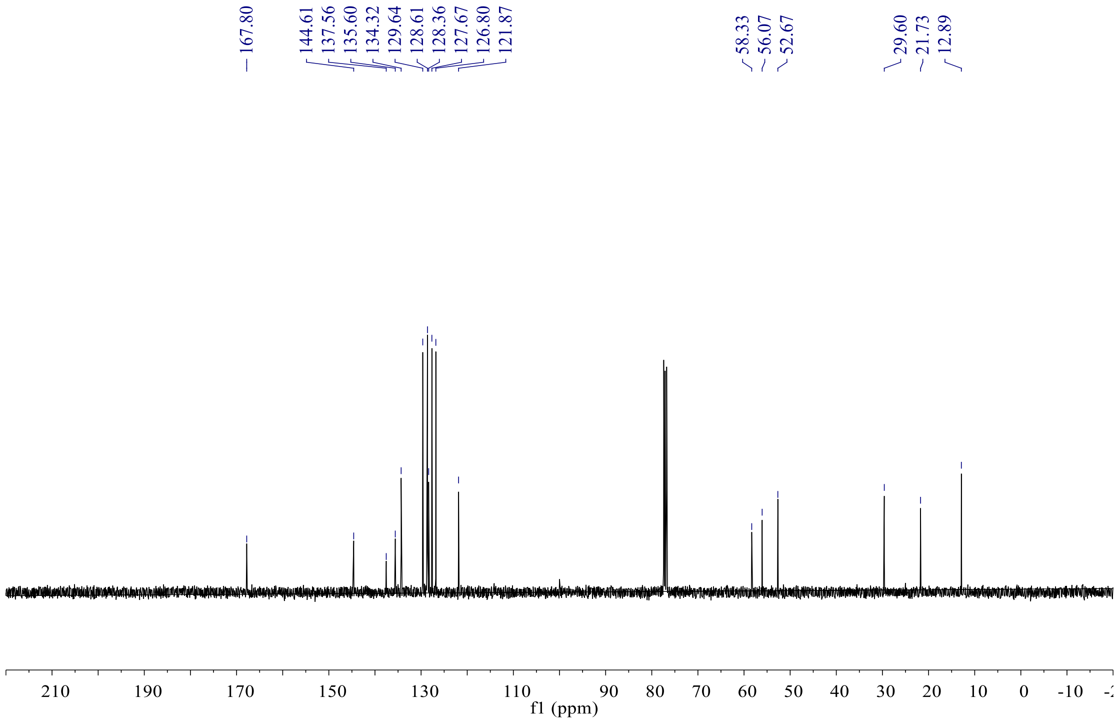

Supplementary Fig. 108 ^{13}C NMR (100 MHz, $CDCl_3$) spectrum of 5aa

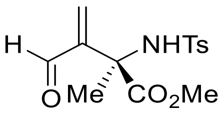

(S)-6aa

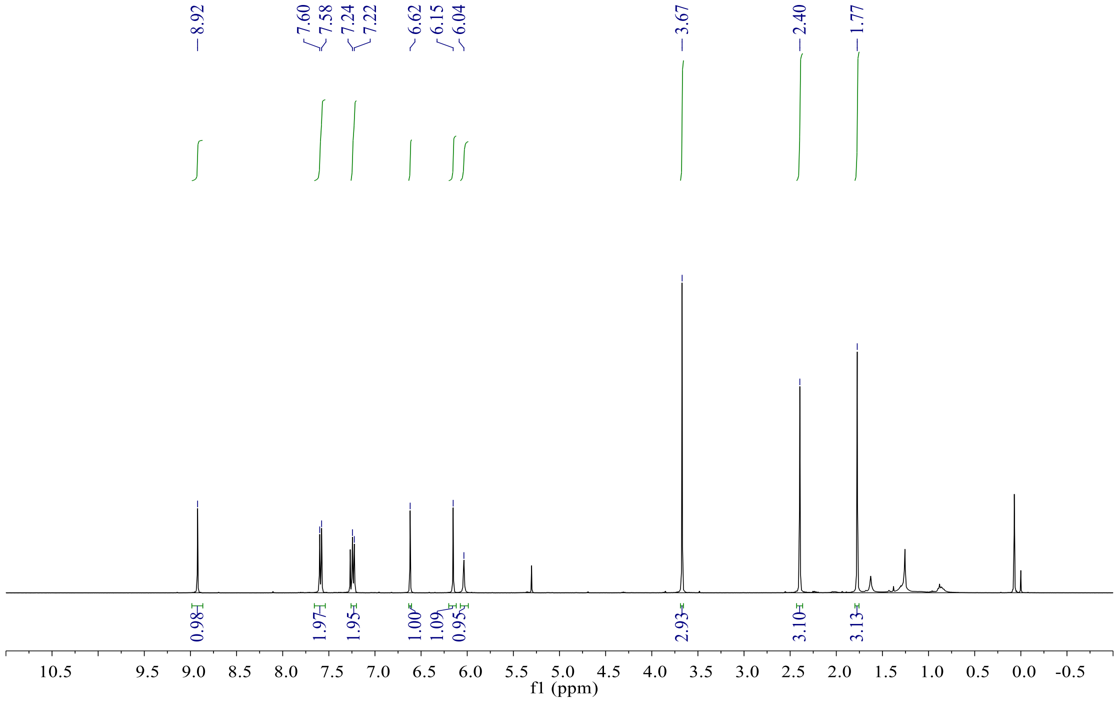

Supplementary Fig. 109 ^{1}H NMR (400 MHz, $CDCl_3$) spectrum of (*S*)-6aa

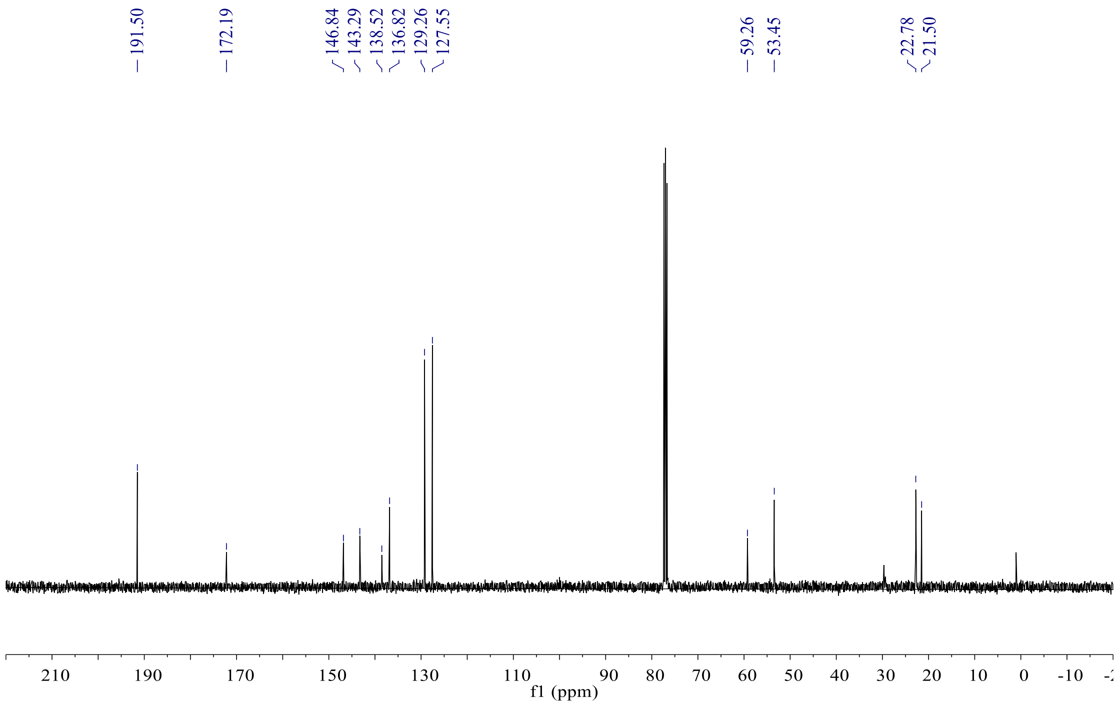

Supplementary Fig. 110 ^{13}C NMR (100 MHz, CDCl$_3$) spectrum of (*S*)-6aa

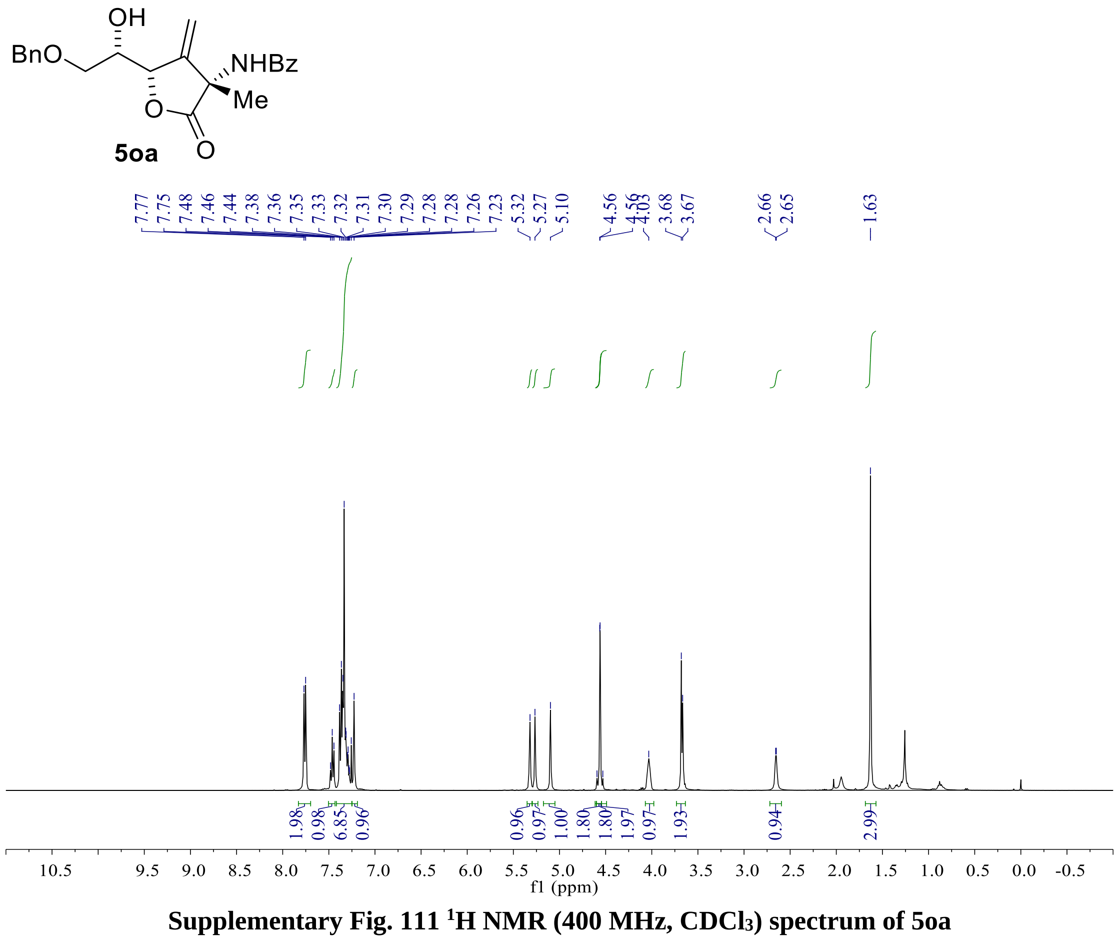

Supplementary Fig. 111 ^{1}H NMR (400 MHz, CDCl$_3$) spectrum of 5oa

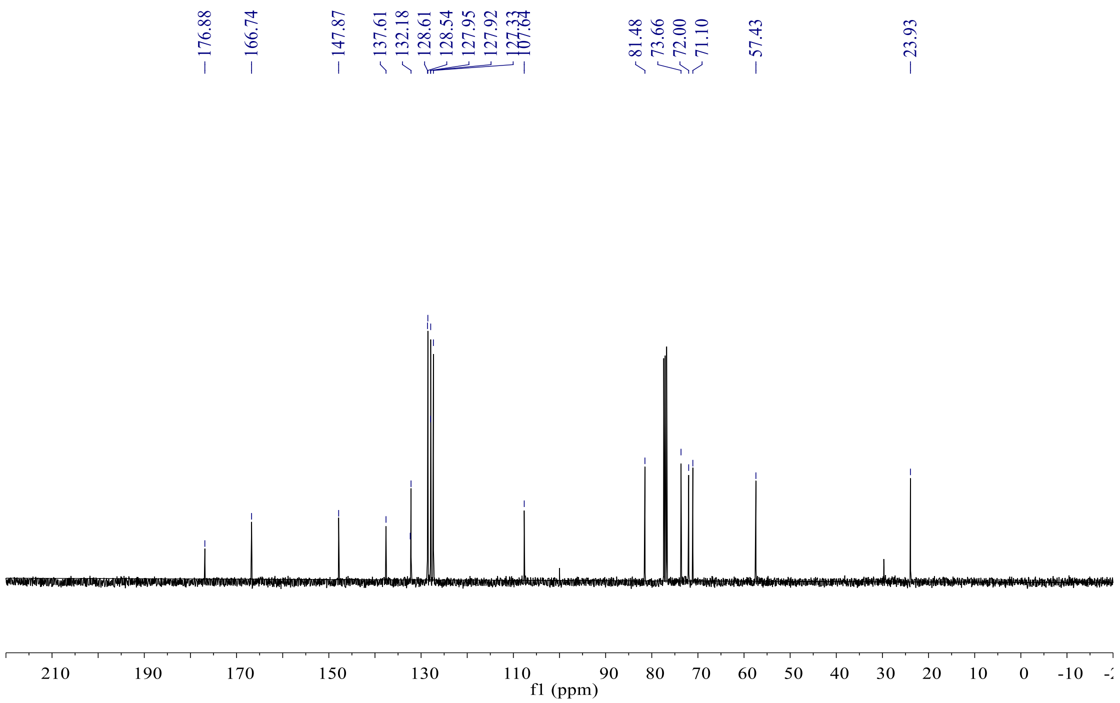

Supplementary Fig. 112 ^{13}C NMR (100 MHz, CDCl$_3$) spectrum of 3oa

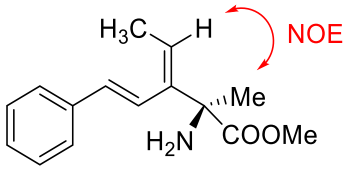

(S)-**3pa**

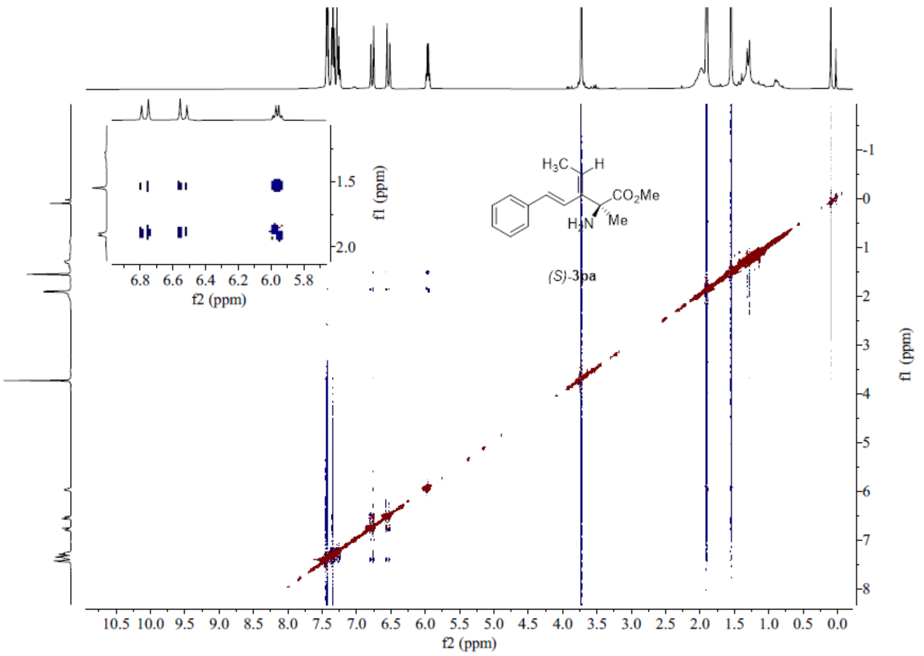

Supplementary Fig. 113 2D NOSEY (400 MHz, CDCl$_3$) spectrum of (*S*)-3pa

4. Supplementary References

1. A. Hedhli, A. Baklouti. *Tetrahedron. Letters.* **1995**, *25*, 4433-4436.
2. De, S.; Day, C.; Welker, M. E. *Tetrahedron*. **2007**, *63*, 10939-10948.
3. a) Huo, X.; He, R.; Fu, J.; Zhang, J.; Yang, G.; Zhang, W. *J. Am. Chem. Soc.* **2017**, *139*, 9819-9822. b) Liu, P.; Huo, X.; Li, B.; He, R.; Zhang, J.; Xie, F.; Zhang, W. *Org. Lett.* **2018**, *20*, 6564-6568. c) Guerrero-Corella, A.; Esterban, F.; Iniesta, M.; Martín-Somer, A.; Parra, M.; Díaz-Tendero, S.; Fraile, A.; Alemán, J. *Angew. Chem., Int. Ed.* **2018**, *57*, 5350- 5354.
