# Supplementary material for: Synergistic Pd/Cu-catalyzed enantioselective Csp2–F bond alkylation of fluoro-1,3-dienes with aldimine esters
Source: Nat Commun. 2022 May 5;13:2470. doi: 10.1038/s41467-022-30152-7 (PMC9072389; doi:10.1038/s41467-022-30152-7)
Supplement: Supplementary file 2 — Description of Additional Supplementary Files [file 41467_2022_30152_MOESM2_ESM.docx]

**Description of Additional Supplementary Files**

**File Name:** Supplementary Data 1

**Description:** Coordinates and Energies of Stationary Points
