# Supplementary material for: Synergistic Pd/Cu-catalyzed enantioselective Csp2–F bond alkylation of fluoro-1,3-dienes with aldimine esters
Source: Nat Commun. 2022 May 5;13:2470. doi: 10.1038/s41467-022-30152-7 (PMC9072389; doi:10.1038/s41467-022-30152-7)
Supplement: Supplementary file 3 — Supplementary Data 1 [file 41467_2022_30152_MOESM3_ESM.pdf]

Supplementary Data

Coordinates and Energies of Stationary Points

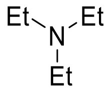

Total SCF energy (M06-2X/def2TZVP/SMD(THF)): -292.373547 a.u.

Thermal correction to Gibbs Free Energy at 298.15 K: 0.173245 a.u

Gibbs free energy at 298.15 K (M06-2X/def2TZVP/SMD(THF)): -292.200302 a.u.

N	-0.00002300	-0.00002700	0.01868900
C	-0.05205400	1.40072800	0.44709700
H	-1.09371500	1.73179300	0.39399400
H	0.25075500	1.50746100	1.50756600
C	0.78955400	2.31857800	-0.43841200
H	0.45328200	2.24825300	-1.47767600
H	0.69841400	3.35968400	-0.10795600
H	1.85263700	2.05613100	-0.41158400
C	1.23900000	-0.65538700	0.44726200
H	2.04651400	0.08125900	0.39469400
H	1.17970200	-0.97133900	1.50761300
C	1.61350600	-1.84283200	-0.43850900
H	1.72098800	-1.51610500	-1.47763900
H	2.56067400	-2.28440800	-0.10791700
H	0.85481100	-2.63239900	-0.41213800
C	-1.18711000	-0.74549000	0.44699400
H	-0.95298600	-1.81312900	0.39374900
H	-1.43093700	-0.53675600	1.50750100
C	-2.40288300	-0.47556600	-0.43841800
H	-3.25884100	-1.07515900	-0.10791500
H	-2.70726100	0.57627300	-0.41146500
H	-2.17395800	-0.73154800	-1.47772800

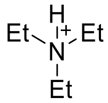

Total SCF energy (M06-2X/def2TZVP/SMD(THF)): -292.836445 a.u.

Thermal correction to Gibbs Free Energy at 298.15 K: 0.188695 a.u

Gibbs free energy at 298.15 K (M06-2X/def2TZVP/SMD(THF)): --292.647550 a.u.

N	-0.00004100	-0.00006000	0.01484000
C	-0.19412300	1.44193500	0.45782500
H	-1.26491000	1.64351000	0.40957200
H	0.11440100	1.47679200	1.50579500
C	0.57882900	2.42077800	-0.41236100
H	0.25237800	2.37150800	-1.45724400
H	0.38199000	3.43576600	-0.05580200
H	1.66062200	2.26527700	-0.37217200
C	1.34577000	-0.55297400	0.45796200
H	2.05566000	0.27366000	0.41015300
H	1.22148900	-0.83793700	1.50581400
C	1.80738700	-1.71144100	-0.41248700

H	1.92813500	-1.40371900	-1.45723900
H	2.78478800	-2.04841800	-0.05581600
H	1.13197000	-2.57068500	-0.37274200
C	-1.15181800	-0.88912800	0.45777000
H	-0.79102500	-1.91725100	0.40945200
H	-1.33628100	-0.63944900	1.50576000
C	-2.38603200	-0.70912700	-0.41239100
H	-3.16657900	-1.38715700	-0.05588200
H	-2.79230800	0.30546600	-0.37210800
H	-2.18013500	-0.96710300	-1.45730100
H	0.00001400	-0.00008900	-1.01202200

Et_3NHF

Total SCF energy (M06-2X/def2TZVP/SMD(THF)): -392.857439 a.u.

Thermal correction to Gibbs Free Energy at 298.15 K: 0.184634 a.u

Gibbs free energy at 298.15 K (M06-2X/def2TZVP/SMD(THF)): --392.672805 a.u.

N	-0.00000900	-0.00025900	-0.38080900
C	-1.37558600	-0.33737000	-0.80502100
H	-1.97504800	0.57282400	-0.72164400
H	-1.38411400	-0.62853100	-1.86884600
C	-2.00154200	-1.41989000	0.07395000
H	-1.91269800	-1.13520500	1.12590900
H	-3.05976000	-1.54494000	-0.17816200
H	-1.51482300	-2.39214800	-0.05597800
C	0.97971600	-1.02327900	-0.80432900
H	0.49143500	-1.99749900	-0.71918500
H	1.23530100	-0.88653300	-1.86855000
C	2.23087900	-1.02258000	0.07365100
H	1.94076200	-1.08619800	1.12596000
H	2.86806300	-1.87694100	-0.17752700
H	2.82943100	-0.11518500	-0.05835300
C	0.39609300	1.35950300	-0.80499700
H	1.48397500	1.42365100	-0.72040200
H	0.14937100	1.51213300	-1.86914300
C	-0.22955500	2.44297300	0.07300300
H	0.19163600	3.42188900	-0.17868300
H	-1.31472700	2.50762800	-0.05840800
H	-0.02881300	2.22379100	1.12525300
H	-0.00008200	0.00027600	1.20208900
F	0.00001300	0.00074000	2.19153300

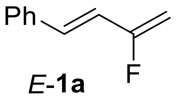

E-**1a**

Total SCF energy (M06-2X/def2TZVP/SMD(THF)): -486.281668 a.u.

Thermal correction to Gibbs Free Energy at 298.15 K: 0.123624 a.u

Gibbs free energy at 298.15 K (M06-2X/def2TZVP/SMD(THF)): -486.158044 a.u.

C	2.77259600	1.33224200	-0.00000900
C	1.39695800	1.13272800	-0.00000700
C	0.85623300	-0.16710900	0.00000200
C	1.74898700	-1.25354500	0.00001300
C	3.12732200	-1.05459100	0.00001100

C	3.64586700	0.24027100	-0.00000200
H	3.16856000	2.34397600	-0.00001600
H	0.73566300	1.99380000	-0.00000800
H	1.34796300	-2.26400200	0.00002300
H	3.79608700	-1.91068500	0.00001800
H	4.72018600	0.40015300	-0.00000800
C	-0.57941100	-0.43962900	0.00000700
H	-0.85568600	-1.49123200	0.00004400
C	-1.57804900	0.46549600	-0.00001600
H	-1.37758000	1.53323100	-0.00004600
C	-2.98111500	0.10756800	-0.00000100
C	-4.02221400	0.94728900	0.00003100
H	-3.85796200	2.01797400	0.00005100
H	-5.03987500	0.57652300	0.00003700
F	-3.21782300	-1.22934000	-0.00003000

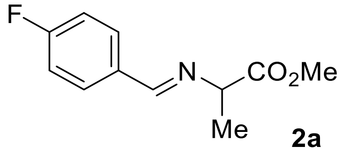

2a

Total SCF energy (M06-2X/def2TZVP/SMD(THF)): -731.432683 a.u.

Thermal correction to Gibbs Free Energy at 298.15 K: 0.170752 a.u

Gibbs free energy at 298.15 K (M06-2X/def2TZVP/SMD(THF)): -731.261931 a.u.

C	4.06128000	-0.24294900	0.07788500
C	3.44246700	0.85355100	0.67550700
C	2.06060900	0.96453400	0.59680900
C	1.30152900	-0.00997100	-0.07234000
C	1.95691600	-1.09948700	-0.66196200
C	3.34267800	-1.22517200	-0.59203300
H	4.04815600	1.59389500	1.18717600
H	1.54168000	1.80299200	1.04853000
H	1.37447200	-1.85540900	-1.18250400
H	3.86656200	-2.06085100	-1.04293300
C	-0.16220500	0.08847700	-0.16812300
H	-0.65270600	-0.72824600	-0.71248100
N	-0.80187400	1.06478200	0.34721400
C	-2.25513900	1.18497800	0.21756800
C	-2.56639500	2.24435200	-0.84762100
H	-3.63346800	2.49074100	-0.85331100
H	-1.99222200	3.15082600	-0.63782100
H	-2.28871700	1.86467700	-1.83478000
C	-2.97160500	-0.11912600	-0.11982700
O	-2.92467500	-0.68275000	-1.19524800
O	-3.64353100	-0.60356900	0.94421000
C	-4.31773600	-1.85439600	0.72535200
H	-4.80928200	-2.09094400	1.66885700
H	-5.05036300	-1.75765300	-0.07993300
H	-3.59854300	-2.63299400	0.45820800
F	5.40258900	-0.35269700	0.15351700
H	-2.62449800	1.52556300	1.18986500

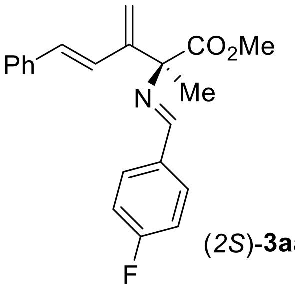

(2*S*)-**3aa**

Total SCF energy (M06-2X/def2TZVP/SMD(THF)): -1117.262544 a.u.

Thermal correction to Gibbs Free Energy at 298.15 K: 0.304616 a.u

Gibbs free energy at 298.15 K (M06-2X/def2TZVP/SMD(THF)): -1116.957928 a.u.

C	-0.71142900	-0.90460100	-0.51135600
C	-2.14831000	-0.60151600	-0.56976900
H	-2.46582500	0.13282100	-1.30564100
C	-3.06356600	-1.12232400	0.26961600
H	-2.71265900	-1.77486900	1.06758300
C	-4.50574500	-0.86806400	0.25517700
C	-5.28139800	-1.28869700	1.34949800
C	-5.15990100	-0.21399100	-0.80558900
C	-6.65348400	-1.05268100	1.39447800
H	-4.79310800	-1.80102100	2.17480700
C	-6.52925900	0.02413700	-0.76068000
H	-4.59283000	0.09629000	-1.67793400
C	-7.28400200	-0.39195900	0.34001500
H	-7.23027000	-1.38552500	2.25292000
H	-7.01419400	0.52891800	-1.59179600
H	-8.35417300	-0.20843100	0.36984500
C	6.54517700	-0.63376900	0.32942500
C	5.67077600	-1.44654000	-0.38971400
C	4.34255500	-1.05863700	-0.50556200
C	3.88912000	0.12952900	0.09119400
C	4.79657400	0.92358800	0.80528000
C	6.13231200	0.54830000	0.93125400
H	6.04244200	-2.35932200	-0.84300800
H	3.63215300	-1.66236300	-1.05981300
H	4.45050000	1.84283600	1.27080600
H	6.84774500	1.14922400	1.48185700
C	2.48662800	0.55738600	-0.01857300
H	2.25468000	1.51658100	0.45739500
N	1.61812600	-0.15725400	-0.61603300
C	0.24143200	0.27491300	-0.80482800
C	0.12969500	0.68951800	-2.28928400
H	-0.89676300	0.92200400	-2.57548300
H	0.74692400	1.57256700	-2.47973700
H	0.49796900	-0.13635300	-2.90249100
C	-0.21248500	1.39874700	0.15540200
O	0.08739300	1.46915000	1.32693700
O	-1.04441200	2.27577400	-0.44593300
C	-1.63598700	3.25138700	0.42943600
H	-2.26825100	3.86929900	-0.20796300
H	-2.23120900	2.75211200	1.19804500
H	-0.86183500	3.85401700	0.91019800
F	7.83515200	-1.00797500	0.44252300
C	-0.23946500	-2.12081700	-0.20812200
H	0.82410200	-2.30366200	-0.12158900

H	-0.91791000	-2.95542500	-0.06030900

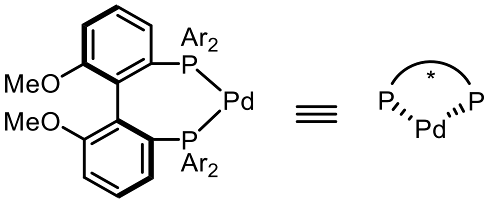

Ar = 3,5-^{t}Bu-C_6H_3

Total SCF energy (M06-2X/def2TZVP/SMD(THF)): -3686.139686 a.u.

Thermal correction to Gibbs Free Energy at 298.15 K: 1.382068 a.u

Gibbs free energy at 298.15 K (M06-2X/def2TZVP/SMD(THF)): -3684.757618 a.u.

Pd	-0.01273000	0.01409000	-1.92441800
P	-1.89323300	0.03969400	-0.63358300
P	1.86338800	-0.02045600	-0.62815200
O	0.96244800	1.16021300	3.61206500
O	-0.99146000	-1.02613600	3.64420200
C	-3.55319000	0.71091500	-1.07030500
C	-4.77066900	0.20884200	-0.59144100
H	-4.76056500	-0.64243800	0.07338800
C	-5.98348200	0.78337000	-0.97564400
C	-5.95113700	1.88964200	-1.84169800
C	-4.75643400	2.41178400	-2.34391600
C	-3.56143900	1.78794400	-1.95972000
H	-2.60999400	2.14026900	-2.34955500
C	-7.33507500	0.25056600	-0.47918100
C	-7.17408400	-0.95397400	0.46376700
H	-6.60271400	-0.69145000	1.36097600
H	-8.16016400	-1.30425000	0.78816300
H	-6.66727400	-1.79052900	-0.02944200
C	-8.18455400	-0.19398400	-1.68956200
H	-7.67725100	-0.98856600	-2.24770900
H	-9.15669500	-0.57597300	-1.35507900
H	-8.36929600	0.63506100	-2.38022100
C	-8.07564200	1.36738500	0.28800700
H	-8.25887400	2.24136500	-0.34523700
H	-9.04588800	1.00491100	0.64856800
H	-7.48976700	1.69722000	1.15321700
C	-4.70238800	3.62120600	-3.28713100
C	-4.03196800	3.20460600	-4.61441300
H	-4.60399300	2.40955200	-5.10536700
H	-3.97591200	4.06007800	-5.29833700
H	-3.01418200	2.83478900	-4.45487900
C	-3.87103400	4.74056900	-2.62188100
H	-2.84869300	4.41372300	-2.40933000
H	-3.81398700	5.61680500	-3.27903500
H	-4.32655600	5.05215000	-1.67500700
C	-6.09887600	4.17981000	-3.60483400
H	-6.61659300	4.51637000	-2.69957200
H	-6.00754200	5.04139200	-4.27528500
H	-6.72910700	3.43547600	-4.10428000
C	-2.22552100	-1.61110900	0.08926300
C	-1.69554600	-2.70658500	-0.58990600

H	-1.09543700	-2.51345500	-1.47447600
C	-1.89724600	-4.01544400	-0.13263500
C	-2.67762300	-4.18670700	1.01175000
C	-3.22221000	-3.10437100	1.72654900
C	-2.96789800	-1.81359100	1.26203700
H	-3.32101800	-0.95108200	1.81206600
C	-4.09298800	-3.37723700	2.96056400
C	-3.35319700	-4.31993600	3.93329800
H	-2.38422300	-3.89984700	4.21862200
H	-3.94628600	-4.47151200	4.84291200
H	-3.16981900	-5.30380000	3.49076300
C	-5.40408700	-4.05382100	2.50305900
H	-5.20560800	-4.99666200	1.98232700
H	-6.04685300	-4.27064100	3.36528500
H	-5.95783300	-3.40238600	1.81850800
C	-4.44754800	-2.08274100	3.71182500
H	-5.06930800	-1.41991100	3.10022200
H	-5.01560500	-2.32262500	4.61755900
H	-3.54889000	-1.53027200	4.00062300
C	-1.25816900	-5.18056600	-0.89935400
C	0.27311900	-4.98042000	-0.93575400
H	0.68712700	-4.97226600	0.07696500
H	0.74915500	-5.79017500	-1.50257000
H	0.54574600	-4.03016000	-1.40510300
C	-1.80587700	-5.20020200	-2.34293200
H	-1.58563800	-4.26565000	-2.86854100
H	-1.35393700	-6.02176000	-2.91187600
H	-2.89294100	-5.33838900	-2.34514500
C	-1.55230400	-6.54116700	-0.24798200
H	-2.62659500	-6.75656500	-0.22474700
H	-1.06656800	-7.33834100	-0.82147200
H	-1.17028200	-6.58809200	0.77808600
C	2.23563200	1.63271900	0.06907900
C	2.98368400	1.83448500	1.23825400
H	3.31746900	0.97176000	1.79989500
C	3.26813800	3.12550000	1.68459300
C	2.74630500	4.20975800	0.95602800
C	1.96041600	4.04031100	-0.18501400
C	1.72950600	2.73031000	-0.62467100
H	1.12474100	2.53861000	-1.50629500
C	3.50257000	-0.73540400	-1.07040000
C	4.73014700	-0.26726500	-0.59407500
H	4.74485000	0.58524700	0.06930200
C	5.92949900	-0.87906000	-0.97621200
C	5.86092600	-1.98159600	-1.83659900
C	4.64883600	-2.47300100	-2.34135100
C	3.47470000	-1.81909200	-1.95932500
H	2.51179100	-2.13946600	-2.34225600
C	4.15092600	3.39434300	2.91096900
C	5.48292100	4.01547500	2.43530000
H	5.31435400	4.95319600	1.89507100
H	6.13604000	4.22847300	3.29069300
H	6.01149700	3.33114900	1.76307100
C	4.46268200	2.10410300	3.68827100
H	5.06232000	1.40885600	3.09075100
H	5.03825400	2.34307100	4.58947900
H	3.54611200	1.58745400	3.98722500

C	3.44887100	4.38257900	3.86616500
H	2.46628200	4.00403900	4.16278300
H	4.05019500	4.53040000	4.77093300
H	3.30035800	5.36368700	3.40490100
C	1.34738500	5.20902400	-0.96717600
C	1.90928900	5.20633700	-2.40546500
H	1.68021300	4.26964200	-2.92359000
H	1.47553700	6.02891400	-2.98700800
H	2.99831700	5.32799400	-2.39832900
C	-0.18664300	5.03468400	-1.01660700
H	-0.61047000	5.03746000	-0.00790900
H	-0.64448200	5.84934000	-1.59091600
H	-0.47125800	4.08715500	-1.48465000
C	1.65814400	6.57023400	-0.32478700
H	2.73534800	6.76989900	-0.29665700
H	1.18800800	7.36999600	-0.90757400
H	1.27066200	6.63081500	0.69852100
C	7.29532300	-0.38478600	-0.47847700
C	8.15944600	0.03187500	-1.68828200
H	7.67666800	0.83965200	-2.24920400
H	9.14188900	0.38585900	-1.35313100
H	8.32060700	-0.80380600	-2.37685800
C	7.16761300	0.82620300	0.46139200
H	6.58693900	0.58296900	1.35810800
H	8.16283600	1.14829700	0.78701000
H	6.68666500	1.67598200	-0.03508200
C	8.00091700	-1.52133900	0.29253500
H	8.15950700	-2.40153400	-0.33878800
H	8.98064500	-1.18710800	0.65470200
H	7.40358300	-1.83235300	1.15685600
C	4.64875400	-3.68720000	-3.28033500
C	5.49844100	-3.37135000	-4.53004800
H	6.53494000	-3.13608100	-4.26767300
H	5.51160300	-4.23155300	-5.21012800
H	5.08658600	-2.51256300	-5.07142900
C	3.23168300	-4.06562000	-3.74318800
H	2.75353400	-3.24892900	-4.29527400
H	3.28213900	-4.93361100	-4.40988000
H	2.58587600	-4.33151400	-2.89956900
C	5.25331400	-4.89870400	-2.53763200
H	5.26924600	-5.77910400	-3.19133300
H	6.28013700	-4.70299200	-2.21198400
H	4.66041900	-5.14275300	-1.64894100
C	-0.35539900	0.70851800	1.69830900
C	-1.39290100	1.08946800	0.81556000
C	-1.99254600	2.35142000	0.96322100
H	-2.78807800	2.64947200	0.29235200
C	-1.58414800	3.22770300	1.96112500
H	-2.05731500	4.20167100	2.05300100
C	-0.58251000	2.85861100	2.85449300
C	0.00537800	1.60078100	2.73129300
C	0.86481200	1.61983100	4.95002700
H	-0.16538100	1.53871600	5.31730100
H	1.52001000	0.97485100	5.54011700
C	1.36239500	-1.03413300	0.84584400
C	1.96747500	-2.28815600	1.03244400
H	2.76211800	-2.60449600	0.36887100

C	1.56496800	-3.13320500	2.05951400
H	2.04252500	-4.10160700	2.18245100
C	0.56311600	-2.73971000	2.94201600
C	-0.03117900	-1.48942300	2.77876800
C	0.32535300	-0.62895500	1.71812100
C	-0.88950800	-1.43666000	4.99790400
H	-1.54485500	-0.77210500	5.56572500
H	0.14131700	-1.33949500	5.35926400
H	1.20430000	2.65594100	5.06202200
H	0.24999000	-3.39630600	3.74546000
H	-1.22636500	-2.46882600	5.14813500
H	-0.26484800	3.53972300	3.63539000
H	-2.86791900	-5.18999400	1.37253500
H	2.95929200	5.21343300	1.30264200
H	-6.88961400	2.34423300	-2.13474200
H	6.78412100	-2.46985100	-2.13271600

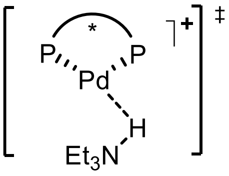

TS-1

Total SCF energy (M06-2X/def2TZVP/SMD(THF)): -3978.967124 a.u.

Thermal correction to Gibbs Free Energy at 298.15 K: 1.588981 a.u

Gibbs free energy at 298.15 K (M06-2X/def2TZVP/SMD(THF)): -3977.378143 a.u.

Pd	0.08513500	0.89991200	-1.32875400
P	-1.82141500	-0.04423900	-0.11582900
P	1.81338600	-0.11710400	-0.30487700
O	1.06628000	-2.50381000	3.38389900
O	-1.06347000	-3.91153300	1.80766300
C	-3.20601400	1.14623400	0.04006000
C	-4.54084600	0.74974800	0.16218400
H	-4.78010400	-0.30374500	0.13270800
C	-5.55864400	1.69820600	0.31375100
C	-5.19474100	3.04974300	0.33656100
C	-3.87087900	3.48797500	0.19948200
C	-2.87993900	2.51177400	0.04678600
H	-1.83717500	2.79672200	-0.05495800
C	-7.03468900	1.30605000	0.46083300
C	-7.23718700	-0.21609200	0.39192300
H	-6.70412900	-0.73470600	1.19688100
H	-8.30117300	-0.45264600	0.49462100
H	-6.89621400	-0.62513200	-0.56538200
C	-7.85187900	1.95484100	-0.67767200
H	-7.49485600	1.61510600	-1.65615200
H	-8.90968700	1.68372700	-0.58655100
H	-7.78494100	3.04719300	-0.65574900
C	-7.55817700	1.80658100	1.82475400
H	-7.47874200	2.89457900	1.91608800
H	-8.61313700	1.53705900	1.94824500
H	-6.99187000	1.35759800	2.64831200
C	-3.57201600	4.99351200	0.23583700

C	-4.40831200	5.71515300	-0.84334500
H	-5.48306300	5.58853700	-0.68405600
H	-4.19554100	6.79009000	-0.83042200
H	-4.17017200	5.33098400	-1.84194200
C	-2.09168200	5.30445800	-0.03237500
H	-1.79201300	4.97170700	-1.03182000
H	-1.92652000	6.38584000	0.01674100
H	-1.43183500	4.83771700	0.70442600
C	-3.94081300	5.54193800	1.63176900
H	-3.35055700	5.04844300	2.41140400
H	-3.74574800	6.61928900	1.68279300
H	-4.99902100	5.38099700	1.86019900
C	-2.54614900	-1.60202200	-0.72638300
C	-2.25778400	-1.97190300	-2.03869700
H	-1.56398900	-1.36249600	-2.61063300
C	-2.83811800	-3.11307700	-2.61157700
C	-3.72586900	-3.85011300	-1.82489600
C	-4.03081600	-3.51240200	-0.49295600
C	-3.40634800	-2.39014300	0.05510700
H	-3.57997500	-2.10925200	1.08626300
C	-5.09591800	-4.31425300	0.27140100
C	-4.84220300	-5.83250000	0.16615500
H	-3.88778300	-6.11264800	0.62111700
H	-5.63492000	-6.37853600	0.68928400
H	-4.83568100	-6.18059500	-0.87086100
C	-6.47172900	-4.00376200	-0.36214300
H	-6.49881800	-4.29685400	-1.41692300
H	-7.26508500	-4.54942400	0.16181500
H	-6.69618400	-2.93449500	-0.30382800
C	-5.14352900	-3.93121800	1.76055100
H	-5.45574800	-2.89096400	1.90312100
H	-5.86860800	-4.56371300	2.28296100
H	-4.16864400	-4.06069100	2.24308200
C	-2.48940900	-3.49132800	-4.05717600
C	-0.97534500	-3.77909800	-4.15026500
H	-0.70371000	-4.62483200	-3.51017400
H	-0.69725700	-4.02407400	-5.18191000
H	-0.37808300	-2.91925600	-3.83017100
C	-2.84750100	-2.31543000	-4.99254300
H	-2.29191300	-1.40767900	-4.73383600
H	-2.60727000	-2.56993600	-6.03111800
H	-3.91654800	-2.08316900	-4.93568200
C	-3.25023800	-4.73800600	-4.53662000
H	-4.33492400	-4.58521300	-4.51553700
H	-2.96818100	-4.96692700	-5.56955000
H	-3.01416000	-5.61724400	-3.92702700
C	2.03893300	0.60200700	1.36164000
C	2.88091200	-0.00551700	2.30379100
H	3.35586200	-0.94421500	2.06370200
C	3.09338300	0.58309500	3.54748400
C	2.45032400	1.80337800	3.82101600
C	1.60475700	2.43329200	2.90709800
C	1.40596500	1.80660000	1.66745000
H	0.74865100	2.24872600	0.92446800
C	3.51929800	-0.37244000	-0.91555500
C	4.29240600	-1.41356200	-0.37065300
H	3.83404600	-2.10072300	0.32646000

C	5.62043500	-1.58952000	-0.74544400
C	6.15926800	-0.69631600	-1.68901400
C	5.41580800	0.33215300	-2.26805600
C	4.07619000	0.48252900	-1.86262200
H	3.45537100	1.26293800	-2.29022600
C	4.01093600	-0.04494600	4.60379200
C	5.21152000	0.89396300	4.85139400
H	4.88814100	1.87820200	5.20560800
H	5.87984000	0.46722100	5.60813900
H	5.78625200	1.04013500	3.93016500
C	4.54650800	-1.41582300	4.15739500
H	5.17807300	-1.32919300	3.26586900
H	5.15995000	-1.85108700	4.95303500
H	3.73076000	-2.11153800	3.93436500
C	3.22595800	-0.23308900	5.91937600
H	2.34906900	-0.86725400	5.75650100
H	3.85969300	-0.70799300	6.67665700
H	2.87697700	0.72007800	6.32841800
C	0.90345700	3.76599400	3.20689200
C	1.25523600	4.78720600	2.10422000
H	0.92911900	4.44307200	1.11841900
H	0.76391100	5.74666900	2.30208600
H	2.33659200	4.95747500	2.06015500
C	-0.62302700	3.54319200	3.23026800
H	-0.90049300	2.85288200	4.03205200
H	-1.14317800	4.49393900	3.39682800
H	-0.98745200	3.11396300	2.29247600
C	1.32560200	4.35882700	4.56101200
H	2.40390600	4.54764700	4.60321700
H	0.81371600	5.31373600	4.71988700
H	1.05797200	3.69967700	5.39387200
C	6.49459500	-2.70759900	-0.16318200
C	7.64589800	-2.06875700	0.64402600
H	7.25396000	-1.46411600	1.46984300
H	8.29228200	-2.84649900	1.06624600
H	8.26647800	-1.41939800	0.01795600
C	5.70102200	-3.63882000	0.76932400
H	4.86223900	-4.11535200	0.24920000
H	6.35800000	-4.43252100	1.13949700
H	5.30648000	-3.10669000	1.64172300
C	7.07717800	-3.56012600	-1.31136900
H	7.69999400	-2.96713000	-1.98819800
H	7.70155900	-4.36354900	-0.90495200
H	6.27612200	-4.01656700	-1.90335600
C	6.00574400	1.27304400	-3.32771900
C	5.91837300	2.72795500	-2.82172400
H	6.49543200	2.85624500	-1.89963100
H	6.31580900	3.41912600	-3.57374500
H	4.88602300	3.01791800	-2.60841100
C	5.19504400	1.12732000	-4.63339900
H	4.13618600	1.35121300	-4.47651800
H	5.57696200	1.81201300	-5.39946900
H	5.26407200	0.10569100	-5.02247900
C	7.47870500	0.96191300	-3.63999000
H	7.84745000	1.66088200	-4.39784600
H	8.11385400	1.06976000	-2.75407200
H	7.60598300	-0.05150300	-4.03568500

C	-0.31921800	-1.34676200	1.87796700
C	-1.33382500	-0.40191400	1.62302200
C	-1.91902600	0.29785300	2.68561300
H	-2.70182600	1.02123000	2.49235600
C	-1.50272700	0.06002300	3.99364300
H	-1.96512700	0.60127400	4.81395500
C	-0.50075700	-0.86878000	4.26224000
C	0.08184300	-1.57268100	3.20628300
C	1.01062200	-3.29615800	4.56583200
H	-0.00257400	-3.68332300	4.72205200
H	1.70302000	-4.12427300	4.40378200
C	1.17101100	-1.83227200	-0.16148900
C	1.56227900	-2.72999700	-1.17378400
H	2.26113300	-2.41282800	-1.93869200
C	1.07596000	-4.02856900	-1.18260200
H	1.38624000	-4.71695700	-1.96247500
C	0.19783400	-4.46358500	-0.19007500
C	-0.21165100	-3.57264500	0.80203500
C	0.24264100	-2.23134900	0.81392600
C	-1.40884300	-5.28061600	1.94977200
H	-2.05651000	-5.33655200	2.82559000
H	-0.51675400	-5.89929500	2.10855500
H	1.33097200	-2.73749300	5.45231900
H	-0.16341900	-5.48386900	-0.20389100
H	-1.95416800	-5.64550200	1.07350700
H	-0.17813300	-1.04383600	5.28197600
H	-4.21009700	-4.71603300	-2.25755400
H	2.62531600	2.27102900	4.78151000
H	-5.97504100	3.79275300	0.46030800
H	7.19230900	-0.82567300	-1.98452800
H	1.09980000	1.90796300	-2.08657200
N	0.78821200	3.24219000	-2.93902100
C	1.68958000	3.13671400	-4.10406300
H	1.64632300	4.06365800	-4.69505700
H	2.70604400	3.05893500	-3.71327500
C	1.13136400	4.35074300	-2.02040100
H	0.37886600	4.33391500	-1.22769400
H	1.03049400	5.31207200	-2.54446600
C	-0.65976600	3.24305200	-3.29306500
H	-1.21297700	3.24462000	-2.34924700
H	-0.87762800	2.29350200	-3.78804400
C	2.51888700	4.24170400	-1.39797600
H	3.31738800	4.40105000	-2.12626400
H	2.66430300	3.26915800	-0.91838900
H	2.62353800	5.01277800	-0.62943800
C	-1.13055200	4.40640800	-4.16810000
H	-0.96499900	5.37541500	-3.68793600
H	-2.20632600	4.30462200	-4.34516200
H	-0.63445800	4.41553400	-5.14346700
C	1.39605700	1.92246900	-4.97975800
H	2.18720700	1.81478600	-5.72717800
H	0.44786500	2.01225000	-5.51743000
H	1.36958800	1.00871300	-4.37510200

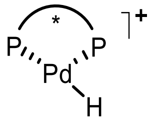

Total SCF energy (M06-2X/def2TZVP/SMD(THF)): -3686.584807 a.u.

Thermal correction to Gibbs Free Energy at 298.15 K: 1.390238 a.u

Gibbs free energy at 298.15 K (M06-2X/def2TZVP/SMD(THF)): -3685.194569 a.u.

Pd	-0.04570600	0.17603000	-1.91267100
P	-1.72780400	0.17131000	-0.22229700
P	1.69926600	-0.03641100	-0.51951400
O	1.32233600	1.55165400	3.69134700
O	-0.68541300	-0.54763000	4.05970800
C	-3.24482100	0.85362000	-0.95624000
C	-4.52051600	0.40611700	-0.60522900
H	-4.62033900	-0.36965700	0.14106700
C	-5.65433200	0.94781800	-1.21998100
C	-5.46195400	1.93835800	-2.19282300
C	-4.19579100	2.40334600	-2.57615600
C	-3.08446400	1.83996600	-1.94236600
H	-2.08271200	2.17832800	-2.19282100
C	-7.07437500	0.49133600	-0.86071300
C	-7.07544100	-0.58970300	0.23432200
H	-6.62450000	-0.22571400	1.16491800
H	-8.10585300	-0.88291400	0.45923200
H	-6.53576000	-1.48990400	-0.08066400
C	-7.75381900	-0.08994800	-2.11984500
H	-7.19621300	-0.95222900	-2.50156800
H	-8.77201500	-0.41828500	-1.88298000
H	-7.82044500	0.65017700	-2.92359100
C	-7.88308500	1.70293900	-0.34779300
H	-7.95658300	2.49250300	-1.10234800
H	-8.90186900	1.39474400	-0.08772900
H	-7.41758800	2.13349900	0.54573800
C	-4.07087100	3.49159300	-3.65019200
C	-4.72774700	2.99679600	-4.95708500
H	-5.78953100	2.77000200	-4.81951800
H	-4.64898500	3.76547200	-5.73384000
H	-4.23467000	2.08930300	-5.32286100
C	-2.60483900	3.84745400	-3.94935800
H	-2.04267900	2.98247600	-4.32081900
H	-2.56553400	4.62122900	-4.72259100
H	-2.09101900	4.23981500	-3.06505300
C	-4.78960700	4.76748400	-3.15900100
H	-4.33666700	5.13844700	-2.23267900
H	-4.71800200	5.55753700	-3.91481000
H	-5.85131700	4.58586100	-2.96445200
C	-2.15390700	-1.40956400	0.55081000
C	-1.73050700	-2.56873100	-0.09726100
H	-1.11004800	-2.47569100	-0.98356300
C	-2.09000800	-3.83510700	0.38437100
C	-2.88880200	-3.88398200	1.52882000
C	-3.31859800	-2.73181800	2.21487200
C	-2.93048600	-1.48849600	1.71605000
H	-3.21525100	-0.57401600	2.22105500
C	-4.22562200	-2.87358600	3.44386100
C	-3.60204400	-3.86110100	4.45299500

H	-2.60479800	-3.53011400	4.76225800
H	-4.23026500	-3.93450400	5.34731700
H	-3.50293400	-4.86816800	4.03711400
C	-5.59633500	-3.41738100	2.98244000
H	-5.49708100	-4.39298700	2.49533000
H	-6.26894300	-3.53450100	3.83984300
H	-6.06694200	-2.73140300	2.26961100
C	-4.44633700	-1.52597900	4.15187500
H	-4.98668600	-0.81732300	3.51437900
H	-5.04688400	-1.67617300	5.05475200
H	-3.49809600	-1.06311500	4.44515700
C	-1.60615200	-5.08751500	-0.35977000
C	-0.06258400	-5.12883200	-0.33271600
H	0.30586300	-5.21126600	0.69472500
H	0.30374900	-5.99182900	-0.90058700
H	0.37604200	-4.22553700	-0.76866600
C	-2.08842500	-5.02679000	-1.82606600
H	-1.68972400	-4.15218100	-2.35086700
H	-1.75873800	-5.91932100	-2.36952300
H	-3.18155000	-4.98009900	-1.87676300
C	-2.13715500	-6.38375900	0.27259200
H	-3.23189000	-6.42257700	0.25930400
H	-1.76860300	-7.24652900	-0.29166400
H	-1.80032900	-6.49831300	1.30889400
C	2.27462200	1.59268800	0.01029100
C	3.20467800	1.69738900	1.05229100
H	3.53083200	0.80405600	1.56791200
C	3.68831500	2.95141800	1.43688500
C	3.15932700	4.07915700	0.79403900
C	2.17698000	4.00528300	-0.20308400
C	1.76360700	2.73587100	-0.61047800
H	1.02795800	2.61461400	-1.39547900
C	3.05057300	-1.00443500	-1.24800200
C	4.37978400	-0.79868300	-0.87112100
H	4.62898300	0.02775800	-0.22155300
C	5.38519300	-1.64803500	-1.34724400
C	5.00725500	-2.70316400	-2.18752000
C	3.68234600	-2.92671900	-2.59037300
C	2.70632600	-2.04911800	-2.11367200
H	1.67050800	-2.16125600	-2.41053500
C	4.79674400	3.10316700	2.48734600
C	6.11088100	3.44594900	1.74990400
H	6.01594300	4.37758200	1.18198600
H	6.93144700	3.56626200	2.46656100
H	6.38375700	2.65008400	1.04843100
C	5.00212100	1.80701100	3.29153200
H	5.38337500	0.99400300	2.66389500
H	5.73926600	1.97581000	4.08298100
H	4.06844900	1.47149100	3.75476900
C	4.46314700	4.24002900	3.47516300
H	3.50514100	4.06119600	3.97285100
H	5.23993400	4.30667200	4.24430600
H	4.40926900	5.21561100	2.98323600
C	1.59740100	5.28633300	-0.81541000
C	0.40889500	4.98676100	-1.74598700
H	-0.38733800	4.45308000	-1.21354900
H	-0.00888000	5.92546700	-2.12403400

H	0.70631000	4.38846000	-2.61426100
C	1.09645300	6.21516900	0.31182200
H	1.90542900	6.52264500	0.98154000
H	0.65930200	7.12396000	-0.11631700
H	0.33138000	5.71385500	0.91346500
C	2.69970100	6.00107500	-1.62582700
H	3.06317900	5.36243100	-2.43826200
H	2.31101300	6.92682100	-2.06507600
H	3.55554500	6.26191800	-0.99433900
C	6.86145600	-1.45523000	-0.97551200
C	7.68052700	-1.21562300	-2.26267100
H	7.33123300	-0.31919800	-2.78645500
H	8.73959100	-1.07839100	-2.01741300
H	7.60470800	-2.06017900	-2.95482600
C	7.07086800	-0.25431500	-0.03728800
H	6.53324800	-0.37945000	0.90992100
H	8.13484300	-0.15365600	0.19913600
H	6.74424200	0.68452000	-0.49863800
C	7.37575300	-2.72435800	-0.26161500
H	7.29480600	-3.61094400	-0.89838000
H	8.43054200	-2.60437000	0.00941900
H	6.80670500	-2.91489000	0.65520300
C	3.34456400	-4.10813500	-3.50876500
C	4.18776800	-4.01881400	-4.79894900
H	5.26151700	-4.05324300	-4.59028000
H	3.95189000	-4.85872900	-5.46165300
H	3.97889100	-3.08845800	-5.33812400
C	1.85823500	-4.12930000	-3.90534300
H	1.56638700	-3.21507500	-4.43480000
H	1.67043100	-4.97469000	-4.57496600
H	1.20393300	-4.25016900	-3.03494700
C	3.66975900	-5.42201300	-2.76425200
H	3.44672900	-6.28496300	-3.40170200
H	4.72634700	-5.47692200	-2.48276600
H	3.07228200	-5.50935200	-1.84941600
C	-0.14373800	1.00063600	1.93757100
C	-1.19912700	1.35004200	1.07524000
C	-1.74957500	2.63821300	1.11328800
H	-2.56528100	2.90094000	0.45030300
C	-1.25503400	3.57598700	2.01588900
H	-1.68578900	4.57218800	2.04973900
C	-0.22090200	3.24515400	2.88907300
C	0.32443100	1.95920300	2.85809600
C	1.42044100	2.19024300	4.95968400
H	0.44701200	2.19986400	5.46358700
H	2.13318600	1.59977400	5.53737200
C	1.23909400	-0.95347900	0.99998100
C	1.71074800	-2.26902500	1.14200600
H	2.33710300	-2.71045800	0.37848700
C	1.38530200	-3.00623200	2.27432300
H	1.75867100	-4.01964700	2.38235200
C	0.58812600	-2.45679700	3.27446900
C	0.11033600	-1.15139400	3.13685500
C	0.42236900	-0.37955000	1.99608200
C	-0.71389700	-1.08563300	5.37654100
H	-1.26704300	-0.36413100	5.97936400
H	0.30161300	-1.20140000	5.77360100

H	1.79741000	3.21499200	4.87523600
H	0.33655900	-3.04562300	4.14803100
H	-1.23268900	-2.04955800	5.41069400
H	0.15418300	3.98335400	3.58755000
H	-3.19575900	-4.84983800	1.90910700
H	3.51920100	5.05815900	1.08894000
H	-6.33485500	2.36340000	-2.67645900
H	5.77770700	-3.37365100	-2.55255400
H	1.15080600	0.33162500	-2.96638400

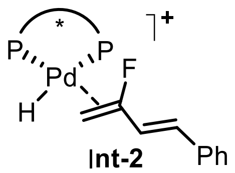

Int-2

Total SCF energy (M06-2X/def2TZVP/SMD(THF)): -4172.897087 a.u.

Thermal correction to Gibbs Free Energy at 298.15 K: 1.546972 a.u

Gibbs free energy at 298.15 K (M06-2X/def2TZVP/SMD(THF)): -4171.350115 a.u.

Pd	-0.33537800	0.04159400	-1.82261000
P	1.20409500	-0.49181200	-0.03177100
P	-2.13384300	0.16810300	-0.39665800
O	-2.13132600	-1.64343800	3.78084000
O	0.06980600	0.16133000	4.30680600
C	2.74423200	-1.36614300	-0.48615200
C	3.97534700	-1.11763200	0.13299600
H	4.05913100	-0.30520300	0.83884900
C	5.09179300	-1.90204800	-0.16429000
C	4.94196400	-2.92997400	-1.10979800
C	3.73193900	-3.19523300	-1.75837100
C	2.63295300	-2.38609200	-1.43538900
H	1.67264800	-2.56314700	-1.90991900
C	6.42794600	-1.73007600	0.57202400
C	6.47178300	-0.42964800	1.39270300
H	5.72794300	-0.43356000	2.19701900
H	7.45548800	-0.32439400	1.86190100
H	6.30027500	0.45140100	0.76829900
C	7.60591100	-1.71348900	-0.42388600
H	7.50846000	-0.88343800	-1.12831300
H	8.54961200	-1.58946800	0.11874600
H	7.67852900	-2.64347300	-0.99587900
C	6.59535300	-2.92715500	1.53548800
H	6.60581600	-3.87828100	0.99269800
H	7.53778700	-2.84229600	2.08880700
H	5.77393200	-2.96208500	2.25992700
C	3.55831500	-4.37329300	-2.72763400
C	2.86541900	-3.91134800	-4.02639000
H	3.45562200	-3.14291000	-4.53575200
H	2.74936200	-4.76138000	-4.70761600
H	1.86926200	-3.49939600	-3.84050300
C	2.68062400	-5.43724200	-2.03039900
H	1.69832200	-5.03349700	-1.76651500
H	2.52521600	-6.29839000	-2.69062400
H	3.15827700	-5.79367500	-1.11085100

C	4.90150800	-5.01960700	-3.10672300
H	5.40405200	-5.46187400	-2.23998800
H	4.72986400	-5.82423700	-3.82895400
H	5.58229200	-4.29556900	-3.56833500
C	1.66813100	1.03091800	0.85625700
C	1.30911600	2.24495200	0.26760900
H	0.70460500	2.22480400	-0.63397400
C	1.71005900	3.46921900	0.82044600
C	2.46847900	3.42507300	1.99317000
C	2.80723100	2.22142500	2.63880000
C	2.39145400	1.02340600	2.05720500
H	2.60652200	0.07668400	2.53472300
C	3.63481200	2.26016800	3.92989600
C	2.96991200	3.21110000	4.94808900
H	1.94186200	2.89925300	5.15959400
H	3.53010400	3.20963500	5.88945000
H	2.93558500	4.24328300	4.58659300
C	5.05154500	2.77892700	3.59853900
H	5.01902000	3.79466700	3.18955800
H	5.67019200	2.80209300	4.50291700
H	5.54224000	2.13436000	2.86197100
C	3.75803600	0.86873100	4.57380300
H	4.31725900	0.17461200	3.93720000
H	4.29799900	0.94742300	5.52284100
H	2.77519700	0.42976300	4.77311300
C	1.31585700	4.78186100	0.12583800
C	-0.21476900	4.95828900	0.21363400
H	-0.53898500	5.02931400	1.25625600
H	-0.52267200	5.87221900	-0.30765200
H	-0.74318100	4.11306600	-0.23811600
C	1.73010100	4.73374400	-1.36159600
H	1.20889400	3.93625200	-1.90255500
H	1.47575800	5.67846400	-1.85428200
H	2.80828200	4.57393000	-1.46961300
C	1.98984700	6.00382600	0.77030700
H	3.08253100	5.92827000	0.73466400
H	1.70146400	6.91142700	0.23038600
H	1.68659400	6.13283900	1.81480300
C	-2.87270600	-1.45464700	-0.08634900
C	-3.80319300	-1.64722900	0.94664400
H	-4.02455200	-0.82914300	1.61943300
C	-4.41091500	-2.88999000	1.11639200
C	-4.02937500	-3.93626000	0.25576400
C	-3.07821000	-3.78078200	-0.75401500
C	-2.51385100	-2.50916400	-0.92233100
H	-1.78071200	-2.32910900	-1.70227200
C	-3.42790100	1.27719900	-1.04717000
C	-4.77086200	1.11535300	-0.70046000
H	-5.06971900	0.25331500	-0.12148700
C	-5.72601300	2.05220100	-1.11170900
C	-5.28601200	3.15202500	-1.86006700
C	-3.94614900	3.33716900	-2.22978400
C	-3.02321800	2.37432100	-1.81554100
H	-1.97832000	2.46181900	-2.08477100
C	-5.48424400	-3.13890800	2.18330400
C	-6.82804900	-3.42118300	1.47564600
H	-6.76204900	-4.29525500	0.81954200

H	-7.61513000	-3.61185300	2.21422600
H	-7.13301700	-2.56398100	0.86515700
C	-5.66595900	-1.92588500	3.11121400
H	-6.03740600	-1.05202800	2.56427400
H	-6.40154800	-2.16173800	3.88711900
H	-4.72667700	-1.64887400	3.59939900
C	-5.09307400	-4.36035600	3.04214200
H	-4.11232100	-4.21132600	3.50483500
H	-5.82971700	-4.51641200	3.83789700
H	-5.04545500	-5.28027900	2.45163000
C	-2.63152800	-4.92348000	-1.67371000
C	-2.99500800	-4.57006600	-3.13254000
H	-2.51157200	-3.64188200	-3.45578400
H	-2.67328400	-5.37015300	-3.80891500
H	-4.07700400	-4.44182400	-3.24470400
C	-1.10126900	-5.09402300	-1.55413900
H	-0.81221900	-5.32700200	-0.52401400
H	-0.75748800	-5.90677200	-2.20360400
H	-0.56941900	-4.18306300	-1.84838600
C	-3.29824300	-6.25997100	-1.31167600
H	-4.38757100	-6.21269300	-1.41739400
H	-2.93636100	-7.04560400	-1.98285800
H	-3.06389700	-6.56489500	-0.28557000
C	-7.21396800	1.90618900	-0.76604800
C	-8.03259100	1.82078900	-2.07266300
H	-7.72605800	0.95445100	-2.66893900
H	-9.09983400	1.72000500	-1.84545100
H	-7.90558800	2.71567100	-2.69018400
C	-7.49493500	0.64254000	0.06544100
H	-6.96231800	0.66013800	1.02333900
H	-8.56549100	0.57748200	0.28455900
H	-7.20983100	-0.26989400	-0.47041800
C	-7.66833400	3.13524900	0.05123600
H	-7.53470000	4.06727700	-0.50704900
H	-8.73051000	3.04892300	0.30619100
H	-7.09838100	3.21639400	0.98357300
C	-3.53153700	4.56806500	-3.04631700
C	-4.37428700	4.64411900	-4.33746000
H	-5.44388100	4.73185600	-4.12400100
H	-4.08157800	5.51920600	-4.92829700
H	-4.22655500	3.75009700	-4.95316600
C	-2.04581200	4.52588900	-3.44607400
H	-1.81568300	3.63911800	-4.04874600
H	-1.80247600	5.40806700	-4.04700400
H	-1.38625000	4.53263200	-2.57165200
C	-3.77176600	5.83298900	-2.19319200
H	-3.49257700	6.73152100	-2.75520100
H	-4.82346700	5.92991700	-1.90500300
H	-3.17300200	5.80290600	-1.27557000
C	-0.57217700	-1.20141600	2.07001100
C	0.42769800	-1.64410600	1.18504200
C	0.77262600	-3.00439900	1.15189800
H	1.54944100	-3.35489100	0.48655500
C	0.13182000	-3.91433900	1.98579800
H	0.40702700	-4.96416500	1.94792500
C	-0.84852400	-3.49033400	2.87874500
C	-1.18828800	-2.13872500	2.92708500

C	-2.30989300	-2.31418800	5.02395700
H	-1.34259300	-2.50492800	5.50279300
H	-2.90549300	-1.64137100	5.64340100
C	-1.73291000	0.91737800	1.23585800
C	-2.14527200	2.23726900	1.48006600
H	-2.72728300	2.77274700	0.74244100
C	-1.82023200	2.86163400	2.67890900
H	-2.15107900	3.87918700	2.86258900
C	-1.07580400	2.19464000	3.64791600
C	-0.66465300	0.88171800	3.41317600
C	-0.98673600	0.22335000	2.20697400
C	0.00388400	0.53534400	5.67798700
H	0.47975600	-0.27605500	6.23091900
H	-1.03765000	0.64603200	6.00189200
H	-2.85266600	-3.25828400	4.90810300
H	-0.82012200	2.69453500	4.57442200
H	0.54844400	1.46504200	5.87567800
H	-1.34185500	-4.20446900	3.52737500
H	2.80556600	4.35477300	2.43333100
H	-4.49277600	-4.90520600	0.39102300
H	5.79928200	-3.55119000	-1.33267100
H	-6.01696800	3.88918400	-2.17470200
H	-1.51733800	0.25381900	-2.85946200
C	0.63258300	0.74691100	-3.89567600
H	0.34271200	1.78469000	-3.77375900
H	0.10805000	0.16095100	-4.64061100
C	1.87919900	0.35337100	-3.48428300
C	2.84035900	1.13157600	-2.74830300
H	2.49139000	2.09283200	-2.38994600
C	4.11544100	0.73301700	-2.54738900
H	4.43611200	-0.21597100	-2.96920200
C	5.12002500	1.50067200	-1.81951500
C	4.76552500	2.39873100	-0.79652000
C	6.47964300	1.34286700	-2.13819400
C	5.74143800	3.13345900	-0.13137300
H	3.72970800	2.48890300	-0.49747100
C	7.45528700	2.08646600	-1.47745700
H	6.76470800	0.64564600	-2.92131800
C	7.08929400	2.98509000	-0.47277800
H	5.44943800	3.81088700	0.66479500
H	8.50076400	1.96059900	-1.74171500
H	7.85044400	3.55720200	0.04900200
F	2.31265400	-0.85228500	-3.89617600

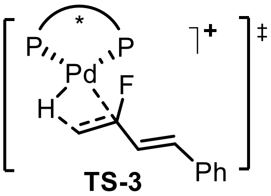

TS-3

Total SCF energy (M06-2X/def2TZVP/SMD(THF)): -4172.893826 a.u.

Thermal correction to Gibbs Free Energy at 298.15 K: 1.545518 a.u

Gibbs free energy at 298.15 K (M06-2X/def2TZVP/SMD(THF)): -4171.348308 a.u.

Pd	0.23680300	-0.12867600	-1.73133100
P	-1.21215800	0.49335900	0.06242500

P	2.10361200	-0.21015300	-0.32239200
O	2.13905600	1.58502800	3.86293600
O	-0.11492500	-0.20846500	4.36104500
C	-2.73731600	1.36732500	-0.42997000
C	-3.99547700	1.08345400	0.11198800
H	-4.10504000	0.25317700	0.79320500
C	-5.10832200	1.85427700	-0.23553900
C	-4.92490800	2.89779500	-1.15560000
C	-3.68546900	3.19446400	-1.73375700
C	-2.59125900	2.40557100	-1.35630300
H	-1.61124900	2.60182200	-1.77628100
C	-6.48121200	1.64524400	0.41876000
C	-6.54885200	0.32579100	1.20606100
H	-5.85216000	0.32327000	2.05156700
H	-7.55548000	0.19368800	1.61622800
H	-6.32694300	-0.53720900	0.57214600
C	-7.60087900	1.63249100	-0.64231800
H	-7.44758200	0.82204500	-1.35936700
H	-8.57065400	1.47875500	-0.15618400
H	-7.65973700	2.57518700	-1.19485900
C	-6.72205100	2.81726900	1.39735400
H	-6.71677200	3.78035400	0.87586600
H	-7.69291100	2.70594200	1.89393500
H	-5.94384900	2.84783400	2.16832700
C	-3.49048700	4.39060300	-2.67658400
C	-2.56809900	4.01998200	-3.85638100
H	-2.97598600	3.18082000	-4.42872300
H	-2.46789500	4.87788500	-4.52998700
H	-1.56132100	3.74537200	-3.52829600
C	-2.84264200	5.53236300	-1.86080200
H	-1.87916500	5.22307900	-1.44341000
H	-2.67168800	6.40958700	-2.49580500
H	-3.49056900	5.83298500	-1.02996300
C	-4.82430600	4.89375700	-3.25686100
H	-5.48521500	5.29843900	-2.48350100
H	-4.63247400	5.70055300	-3.97159700
H	-5.35941300	4.09629900	-3.78460900
C	-1.68586400	-1.03496600	0.93541500
C	-1.30255800	-2.25046400	0.36480600
H	-0.66269100	-2.23322700	-0.51158500
C	-1.73012200	-3.47371600	0.89812100
C	-2.54317600	-3.42744500	2.03321000
C	-2.90881700	-2.22329400	2.66256400
C	-2.46070100	-1.02556900	2.10402100
H	-2.69953200	-0.07817100	2.56895900
C	-3.81537400	-2.26158000	3.89931500
C	-3.23200400	-3.22672500	4.95316100
H	-2.22143000	-2.92294400	5.24581400
H	-3.86096900	-3.23145400	5.84997900
H	-3.17617500	-4.25546600	4.58479500
C	-5.21219100	-2.76294100	3.46853700
H	-5.16157500	-3.77627800	3.05556600
H	-5.89186700	-2.78448500	4.32805000
H	-5.64442900	-2.10967000	2.70378800
C	-3.96434900	-0.87323100	4.54440200
H	-4.47083200	-0.16633100	3.87843900
H	-4.56660300	-0.95153500	5.45515800

H	-2.99125500	-0.44863500	4.81352400
C	-1.31018000	-4.78784400	0.22091000
C	0.21286200	-4.97443500	0.38312200
H	0.48673200	-5.04288400	1.44021900
H	0.53881700	-5.89274900	-0.11937000
H	0.76769600	-4.13412300	-0.04535000
C	-1.64939200	-4.73672100	-1.28537000
H	-1.09821200	-3.94058700	-1.79800500
H	-1.37335600	-5.68211600	-1.76504900
H	-2.71946200	-4.57168800	-1.44874200
C	-2.02288200	-6.00624600	0.82961400
H	-3.11196800	-5.92324500	0.74108900
H	-1.71456200	-6.91488900	0.30258900
H	-1.77149300	-6.13902600	1.88731300
C	2.81071000	1.43403100	-0.04398800
C	3.76054600	1.66743300	0.96195900
H	4.02281700	0.86321400	1.63698500
C	4.33338200	2.93010400	1.10410900
C	3.90401000	3.95304500	0.23776100
C	2.93468100	3.75682100	-0.74739300
C	2.39991700	2.46828300	-0.88164000
H	1.64522400	2.26108400	-1.63467300
C	3.41915700	-1.28042000	-0.99587700
C	4.76991100	-1.05110800	-0.72521300
H	5.05695200	-0.16618100	-0.17545800
C	5.74574900	-1.94920800	-1.17242300
C	5.32052800	-3.07958400	-1.88293800
C	3.97351600	-3.33342300	-2.17819600
C	3.02888800	-2.40875900	-1.72662000
H	1.97591000	-2.55788400	-1.93075000
C	5.41518100	3.22655700	2.15000800
C	6.73396500	3.56209700	1.41926000
H	6.62160400	4.43084800	0.76242400
H	7.52468400	3.78723600	2.14408900
H	7.06386800	2.71628400	0.80579800
C	5.66302400	2.02555400	3.07816800
H	6.05595300	1.16403200	2.52654600
H	6.40503000	2.29301900	3.83755300
H	4.74590300	1.71462700	3.58779900
C	4.98638900	4.43291100	3.01241000
H	4.02304000	4.24080100	3.49567900
H	5.73120100	4.62647500	3.79221800
H	4.88377700	5.34661100	2.41914100
C	2.43671200	4.87300900	-1.67352000
C	2.75072100	4.49516900	-3.13778200
H	2.26550800	3.55620900	-3.42573700
H	2.39515900	5.27839300	-3.81689100
H	3.82918800	4.37601900	-3.28759000
C	0.90968800	5.02373000	-1.50181400
H	0.65496100	5.27747300	-0.46752600
H	0.52949900	5.81486000	-2.15752600
H	0.38142900	4.09830200	-1.75407800
C	3.09551700	6.22668000	-1.36486500
H	4.18142100	6.19129500	-1.50486200
H	2.70066600	6.99131200	-2.04170500
H	2.89016800	6.55273900	-0.33912300
C	7.24082400	-1.72880200	-0.90604500

C	7.98562300	-1.62072300	-2.25452600
H	7.60785800	-0.77691800	-2.84226300
H	9.05718700	-1.46705700	-2.08453200
H	7.86956400	-2.52804600	-2.85590800
C	7.50340900	-0.44314400	-0.10254100
H	7.02127500	-0.47372900	0.88140600
H	8.57944200	-0.32566700	0.06084100
H	7.14886500	0.44846800	-0.63194000
C	7.79545900	-2.92486700	-0.10186000
H	7.67830900	-3.86898300	-0.64329300
H	8.86399500	-2.78488800	0.09628900
H	7.27917100	-3.02176900	0.85972000
C	3.57596700	-4.59689100	-2.95257900
C	4.36356200	-4.66642300	-4.27837500
H	5.44408400	-4.70810500	-4.11175700
H	4.08041700	-5.56421100	-4.83899200
H	4.15374600	-3.79169000	-4.90392400
C	2.07386300	-4.62484900	-3.28662500
H	1.78158300	-3.76091800	-3.89620200
H	1.84154500	-5.52798400	-3.86003000
H	1.45284400	-4.64000400	-2.38479200
C	3.90549400	-5.83263700	-2.08651300
H	3.64157000	-6.75382900	-2.61839500
H	4.97160600	-5.87863000	-1.84211000
H	3.34533100	-5.80804200	-1.14471000
C	0.56956700	1.16875600	2.15743200
C	-0.43190200	1.62839900	1.28349600
C	-0.77951100	2.98861300	1.27282400
H	-1.56001900	3.34719800	0.61604400
C	-0.13665100	3.88467400	2.11932600
H	-0.41338800	4.93461600	2.10174400
C	0.84918600	3.44608000	2.99954800
C	1.19082100	2.09419200	3.02436700
C	2.34835600	2.24905600	5.10472500
H	1.39227800	2.45204900	5.60093200
H	2.94430500	1.56515900	5.71149500
C	1.75016200	-0.94482800	1.32560800
C	2.18376900	-2.25515500	1.58117800
H	2.78722000	-2.78105300	0.85318700
C	1.84942900	-2.88222500	2.77617500
H	2.19677400	-3.89251800	2.96950700
C	1.07132200	-2.22899800	3.72837300
C	0.64495200	-0.92232600	3.48479100
C	0.98196700	-0.25893300	2.28495600
C	-0.14334800	-0.62889100	5.71882400
H	-0.65560200	0.16454100	6.26514400
H	0.87290900	-0.75449300	6.11121900
H	2.90294500	3.18557900	4.98171300
H	0.80612800	-2.73382700	4.64929300
H	-0.70045600	-1.56384100	5.84497400
H	1.34460800	4.15011200	3.65751300
H	-2.90717400	-4.35578100	2.45406300
H	4.34302200	4.93629500	0.34934700
H	-5.78078800	3.50445800	-1.42030500
H	6.06835100	-3.78663200	-2.22564800
H	1.20318500	-0.55210900	-2.95044000
C	-0.22080400	-0.81260300	-3.88850000

H	-0.08622000	-1.88906700	-3.89984100
H	0.25241800	-0.26130600	-4.69433000
C	-1.42503300	-0.30407600	-3.37196500
C	-2.50422800	-1.08244500	-2.80512200
H	-2.23548500	-2.08484800	-2.48949100
C	-3.77193900	-0.63776500	-2.67588500
H	-4.02407700	0.34936200	-3.05364900
C	-4.85386400	-1.41343100	-2.06981700
C	-4.60652500	-2.33681400	-1.03820000
C	-6.17664700	-1.23525400	-2.50768300
C	-5.64529700	-3.07829900	-0.48318500
H	-3.60443200	-2.44007400	-0.64383400
C	-7.21516300	-1.98557300	-1.95862500
H	-6.38389100	-0.51526200	-3.29480700
C	-6.95376300	-2.91151300	-0.94599700
H	-5.43323700	-3.77344500	0.32384200
H	-8.23028900	-1.84334700	-2.31698700
H	-7.76525500	-3.48768500	-0.51190900
F	-1.75956300	0.94740600	-3.78140500

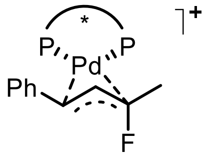

Int-4

Total SCF energy (M06-2X/def2TZVP/SMD(THF)): -4172.943991 a.u.

Thermal correction to Gibbs Free Energy at 298.15 K: 1.548342 a.u

Gibbs free energy at 298.15 K (M06-2X/def2TZVP/SMD(THF)): -4172.395649 a.u.

Pd	-0.09246600	0.10422700	-1.40060600
P	-1.80346700	0.36940400	0.16287900
P	1.50993200	-0.50047600	0.17744300
O	0.93420800	0.32724300	4.51880100
O	-1.54294300	-1.30166500	4.26859100
C	-3.03438900	1.49869700	-0.56821700
C	-4.39142000	1.16481700	-0.64081900
H	-4.73734300	0.26350100	-0.15472500
C	-5.28370900	1.97098100	-1.35253700
C	-4.77976700	3.12294700	-1.97860800
C	-3.43117600	3.49295900	-1.91138900
C	-2.56312000	2.65318700	-1.20196000
H	-1.50363900	2.88590000	-1.15111300
C	-6.77134000	1.61958200	-1.48379000
C	-7.14066100	0.35648900	-0.68670600
H	-6.93518500	0.47941400	0.38278900
H	-8.21033700	0.15200900	-0.79773300
H	-6.59689200	-0.52657000	-1.04054400
C	-7.09675200	1.37048200	-2.97301800
H	-6.50175900	0.53900300	-3.36793200
H	-8.15636400	1.11908200	-3.09408400
H	-6.89006700	2.25371400	-3.58595700
C	-7.62633900	2.79193700	-0.95564600
H	-7.44595400	3.71458500	-1.51605500
H	-8.69170400	2.55207600	-1.04437200
H	-7.40750800	2.99023600	0.09934000
C	-2.88450000	4.77120900	-2.56212100

C	-1.65008100	4.43483500	-3.42743900
H	-1.90739200	3.71893200	-4.21734900
H	-1.26502500	5.34115900	-3.90706600
H	-0.83661900	4.01218400	-2.82874600
C	-2.46849600	5.74980600	-1.44143000
H	-1.70076500	5.31391100	-0.79449300
H	-2.06381500	6.67353600	-1.87084300
H	-3.32894800	6.01230800	-0.81651200
C	-3.92524700	5.46170000	-3.45853700
H	-4.79698100	5.79661300	-2.88672700
H	-3.48033100	6.34681700	-3.92442700
H	-4.27213300	4.80034100	-4.26046500
C	-2.69005800	-1.14698600	0.60347500
C	-2.48438200	-2.26762600	-0.19894800
H	-1.75928600	-2.21078800	-1.00406300
C	-3.19779100	-3.45276700	0.03029100
C	-4.12295500	-3.45732000	1.07570500
C	-4.34430800	-2.34405000	1.90882600
C	-3.60511100	-1.18678300	1.66572900
H	-3.72275100	-0.31068100	2.29072000
C	-5.40540500	-2.42175000	3.01384800
C	-5.17278900	-3.67320900	3.88670300
H	-4.17324300	-3.65936100	4.33336500
H	-5.90885500	-3.71013000	4.69717900
H	-5.26817300	-4.60001800	3.31321200
C	-6.79799500	-2.51784600	2.35162000
H	-6.87835100	-3.40497800	1.71471800
H	-7.58130400	-2.57823300	3.11589300
H	-6.99474500	-1.63833700	1.72874600
C	-5.37660900	-1.18146200	3.92294400
H	-5.64171300	-0.27066500	3.37466200
H	-6.10397000	-1.29891300	4.73271000
H	-4.38774700	-1.03305000	4.36935500
C	-2.92473100	-4.67309200	-0.85727700
C	-1.45208300	-5.09827200	-0.66909200
H	-1.26166200	-5.38856300	0.36932900
H	-1.21580100	-5.95040200	-1.31677500
H	-0.76399000	-4.28324400	-0.91361200
C	-3.16784000	-4.29698200	-2.33580000
H	-2.51542800	-3.48352100	-2.66570000
H	-2.97491600	-5.16135100	-2.98127600
H	-4.20659100	-3.98353700	-2.49024900
C	-3.82556300	-5.86715000	-0.50488100
H	-4.88678500	-5.62632100	-0.63267500
H	-3.59535700	-6.70931500	-1.16553600
H	-3.67008200	-6.20403400	0.52601900
C	2.31113200	0.87633000	1.04065000
C	3.18681100	0.65757000	2.11062500
H	3.35062800	-0.35407200	2.45966200
C	3.83498000	1.73590200	2.72119200
C	3.53265800	3.02450200	2.25827100
C	2.61873700	3.27541800	1.22676300
C	2.02886500	2.17346100	0.60345000
H	1.33387100	2.30201700	-0.21644100
C	2.80407200	-1.51086700	-0.61469500
C	4.16529900	-1.30436600	-0.38791500
H	4.47390000	-0.53859900	0.30880500

C	5.12402300	-2.06022000	-1.07666900
C	4.67098300	-3.00964500	-1.99933600
C	3.30936000	-3.23267400	-2.26051500
C	2.38286000	-2.46904400	-1.54936800
H	1.32102700	-2.59372700	-1.72882800
C	4.88973600	1.53962000	3.81888800
C	6.27870600	1.83837400	3.21073500
H	6.33638800	2.86742300	2.84029600
H	7.06357500	1.70406900	3.96408000
H	6.49078800	1.16784600	2.37159200
C	4.88409000	0.10115600	4.36460200
H	5.17969900	-0.62647200	3.60065700
H	5.59867400	0.01463500	5.18945100
H	3.89366600	-0.18167000	4.73814700
C	4.64347300	2.50548000	4.99627700
H	3.65373300	2.34819600	5.43562900
H	5.39204200	2.34025200	5.77871900
H	4.71333400	3.55452900	4.69415800
C	2.31208000	4.71712300	0.80354900
C	3.57217900	5.32206100	0.14798300
H	3.87679000	4.73091300	-0.72135100
H	3.37821100	6.35009100	-0.17998800
H	4.41279400	5.34620400	0.84955100
C	1.14715900	4.77926200	-0.19984400
H	0.24157600	4.32780000	0.22143700
H	0.92273600	5.82300700	-0.44319000
H	1.38839100	4.26450500	-1.13457500
C	1.91876500	5.55296600	2.04009100
H	2.72795600	5.60663300	2.77471100
H	1.67739000	6.57885100	1.74001900
H	1.04210500	5.12224100	2.53423600
C	6.62913100	-1.88828000	-0.83008300
C	7.36465500	-1.65195400	-2.16630500
H	6.99753800	-0.74836900	-2.66443400
H	8.43827300	-1.52786300	-1.98745100
H	7.24073900	-2.48948600	-2.85933800
C	6.92759200	-0.70121600	0.10126000
H	6.49487900	-0.84937500	1.09678800
H	8.00976600	-0.59384700	0.22709900
H	6.54075700	0.24089200	-0.30387100
C	7.16834100	-3.17790000	-0.17203500
H	7.01130500	-4.05116700	-0.81363400
H	8.24381300	-3.08791100	0.01854700
H	6.66587800	-3.36754000	0.78291600
C	2.88982700	-4.25309900	-3.32637700
C	3.44223600	-3.79540400	-4.69435200
H	4.53418200	-3.71978500	-4.68643800
H	3.16048600	-4.50911200	-5.47656400
H	3.03972000	-2.81321300	-4.96835000
C	1.36141900	-4.38329600	-3.43847000
H	0.88312400	-3.43961200	-3.72290700
H	1.11284700	-5.12128200	-4.20806600
H	0.91402300	-4.72327600	-2.49864800
C	3.46796800	-5.63937500	-2.96967800
H	3.17920500	-6.37574500	-3.72785600
H	4.56114200	-5.62630400	-2.91874100
H	3.08994900	-5.98161400	-1.99987300

C	-0.38041400	0.43284300	2.57807600
C	-1.21110200	1.15819200	1.70447000
C	-1.49140900	2.51010900	1.95240300
H	-2.14551300	3.06214100	1.28920300
C	-0.94408700	3.13895900	3.06614700
H	-1.16984500	4.18300500	3.26120800
C	-0.11738700	2.43976500	3.94322800
C	0.15442500	1.09013700	3.70565300
C	1.13468400	0.77040800	5.85430400
H	0.17558300	0.97111100	6.34684500
H	1.65065600	-0.04426300	6.36448100
C	0.76045300	-1.57604200	1.46493400
C	0.98372700	-2.96035500	1.42525600
H	1.66002700	-3.38235500	0.69317800
C	0.34702300	-3.79657200	2.33649300
H	0.52565600	-4.86699400	2.30117500
C	-0.51064400	-3.27450500	3.30152000
C	-0.72866100	-1.89611200	3.35241800
C	-0.10784900	-1.02852400	2.42872600
C	-1.81464400	-2.00673200	5.47316000
H	-2.31704600	-1.29380000	6.12873400
H	-0.88490100	-2.35201800	5.94083100
H	1.75959800	1.66878900	5.89634800
H	-1.00141300	-3.93636800	4.00472800
H	-2.47784700	-2.86229400	5.30529000
H	0.30507500	2.94427400	4.80343100
H	-4.69823400	-4.35587800	1.25851800
H	4.02850000	3.86935400	2.72289400
H	-5.46573700	3.74552100	-2.53868800
H	5.40363600	-3.59456300	-2.54450000
C	-2.72045400	0.04401700	-3.61611600
H	-2.90357400	1.11347000	-3.50142600
H	-3.03746900	-0.27383400	-4.61779200
C	-1.27948900	-0.26899700	-3.41756500
C	-0.19162600	0.58624200	-3.57069100
H	-0.43853800	1.63394800	-3.70908700
C	1.14019600	0.18228200	-3.26394200
H	1.38955100	-0.86562800	-3.39686000
C	2.28218900	1.10425100	-3.22499700
C	2.13246400	2.50344400	-3.22636200
C	3.58166800	0.57346600	-3.14184000
C	3.24104600	3.33929800	-3.14167000
H	1.14435500	2.94737700	-3.29292500
C	4.68817100	1.41285400	-3.03928100
H	3.71904600	-0.50244800	-3.15060800
C	4.52595400	2.79877800	-3.03683500
H	3.10296400	4.41633400	-3.15494700
H	5.68200400	0.98190400	-2.97009600
H	5.38969800	3.45262600	-2.96458600
F	-1.02751100	-1.61617900	-3.45069000
H	-3.33021100	-0.49073000	-2.88285100

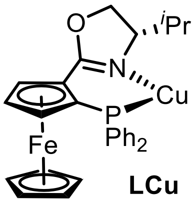

LCu

Total SCF energy (M06-2X/def2TZVP/SMD(THF)): -4459.180587 a.u.

Thermal correction to Gibbs Free Energy at 298.15 K: 0.428591 a.u

Gibbs free energy at 298.15 K (M06-2X/def2TZVP/SMD(THF)): -4458.751996 a.u.

Fe	-0.35690400	-2.11283400	0.84455600
P	1.22420600	0.69920200	-0.34756800
N	-2.16758000	1.13846100	0.06125600
C	-0.79989700	-0.26934900	1.58227900
C	-0.83366700	-1.22101100	2.65886000
H	-1.70930200	-1.45828000	3.24451000
C	0.44468500	-1.82079100	2.76594100
H	0.71448400	-2.61356000	3.44975000
C	1.28982500	-1.25890400	1.77073600
H	2.31136800	-1.54534100	1.56792700
C	0.54798600	-0.29252100	1.01781900
C	-0.24998000	-4.14140900	0.42953600
H	0.28485200	-4.85282400	1.04364400
C	0.30110200	-3.38559200	-0.64682900
H	1.32384900	-3.41910500	-0.99459400
C	-0.72378600	-2.52422600	-1.14568500
H	-0.61841200	-1.80894300	-1.94887800
C	-1.90373100	-2.74897900	-0.37568800
H	-2.84261300	-2.22262400	-0.48455200
C	-1.61160200	-3.74776900	0.59913800
H	-2.28827200	-4.11033500	1.36060600
C	2.53202200	-0.32507400	-1.08659800
C	2.27978000	-0.98748900	-2.29496700
H	1.33039000	-0.84201600	-2.80209200
C	3.24769100	-1.82378500	-2.85086200
H	3.04880100	-2.33346400	-3.78859400
C	4.47542600	-1.99183300	-2.20648900
H	5.23095600	-2.63920300	-2.64082200
C	4.74178300	-1.31131900	-1.01525800
H	5.70451100	-1.42583500	-0.52671100
C	3.77680200	-0.47421300	-0.45697100
H	3.99310900	0.07263900	0.45595600
C	2.06808500	2.09933500	0.45953000
C	2.36852300	2.11014200	1.82837000
H	2.08490000	1.27262900	2.45700800
C	3.03012700	3.20511000	2.38621400
H	3.25947200	3.21103800	3.44745000
C	3.39487200	4.28805000	1.58499500
H	3.90917000	5.13791100	2.02293100
C	3.09367100	4.28244200	0.22051000
H	3.37293100	5.12584600	-0.40338800
C	2.42643900	3.19582500	-0.34043600
H	2.18752600	3.19585100	-1.40142200
Cu	-0.71043600	1.32285700	-1.23153000
C	-2.00521700	0.40924600	1.12196700
O	-3.09891700	0.22813100	1.87787700

C	-4.22048700	0.84015800	1.17816500
H	-4.89058400	0.03259200	0.86862200
H	-4.73352600	1.49199500	1.88491100
C	-3.57116800	1.57902700	-0.01710200
H	-3.59701800	2.66683400	0.13094500
C	-4.20268700	1.26008000	-1.38244400
H	-4.21314700	0.16534700	-1.49090200
C	-5.64699900	1.77695700	-1.42612500
H	-6.12244100	1.51776400	-2.37675400
H	-6.25950300	1.34931100	-0.62449600
H	-5.67380400	2.86853700	-1.32409800
C	-3.38433300	1.85317800	-2.53672800
H	-2.39250600	1.37405500	-2.63464700
H	-3.88724900	1.69344500	-3.49549900
H	-3.23607800	2.93254800	-2.41045100

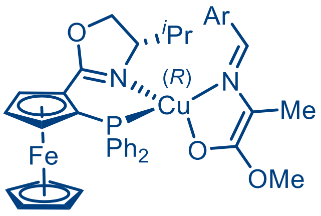

Cu(*R*)-Nu

Total SCF energy (M06-2X/def2TZVP/SMD(THF)): -5190.179708 a.u.

Thermal correction to Gibbs Free Energy at 298.15 K: 0.616009 a.u

Gibbs free energy at 298.15 K (M06-2X/def2TZVP/SMD(THF)): -5189.563699 a.u.

Fe	2.98113200	-0.91068700	-1.32152400
P	0.09917900	-1.28983000	0.50298000
N	0.01151200	1.25714700	-1.61631500
C	1.12974300	-0.87994000	-2.17099000
C	2.04517200	-1.49855700	-3.08399800
H	2.29694200	-1.11847300	-4.06338700
C	2.60585500	-2.63543300	-2.44289300
H	3.37629300	-3.27497000	-2.85168800
C	2.03233400	-2.74517300	-1.14446600
H	2.28164400	-3.48235200	-0.39527900
C	1.11319400	-1.66602100	-0.95243100
C	4.99280400	-0.84717200	-0.84978900
H	5.66597700	-1.68081100	-0.99818400
C	4.20620700	-0.59972600	0.31608200
H	4.17095000	-1.21411500	1.20484900
C	3.41021300	0.56223800	0.07854700
H	2.68364700	1.00195400	0.74820800
C	3.70255700	1.02621300	-1.23798200
H	3.21690700	1.85890500	-1.72893800
C	4.68030100	0.15988100	-1.81369600
H	5.07594100	0.22306100	-2.81853300
C	1.05921300	-2.01251600	1.88312000
C	1.73326000	-1.13178900	2.74141900
H	1.63047700	-0.05948000	2.59487800
C	2.52134900	-1.63687200	3.77738600
H	3.04298500	-0.95176200	4.44007600
C	2.62979000	-3.01579800	3.96960600

H	3.24080500	-3.40612600	4.77877500
C	1.93609400	-3.89468100	3.13303300
H	2.00371500	-4.96714100	3.29380300
C	1.14874000	-3.39534800	2.09595400
H	0.59223600	-4.07553900	1.45719400
C	-1.34473100	-2.40214400	0.32806000
C	-1.49526800	-3.31211700	-0.72427000
H	-0.69730600	-3.43958100	-1.44909800
C	-2.67766300	-4.04582100	-0.85393200
H	-2.79191400	-4.74083100	-1.68129700
C	-3.70959600	-3.88244900	0.06910600
H	-4.63473900	-4.43968200	-0.04417100
C	-3.56049600	-2.98076300	1.12684500
H	-4.36974000	-2.83193200	1.83486500
C	-2.39081600	-2.23759300	1.25022800
H	-2.29819100	-1.49860100	2.04140700
Cu	-0.26673900	0.92137800	0.46332700
C	-5.12921900	-0.88815300	-1.00033300
C	-3.83982600	-0.53060100	-1.37285000
C	-3.14487600	0.38902100	-0.59633700
C	-3.71143900	0.95818000	0.56275200
C	-5.03865800	0.59712900	0.87690900
C	-5.74675600	-0.32374600	0.11009400
H	-3.38482700	-0.99384700	-2.24178300
H	-2.13389700	0.65324000	-0.87655700
H	-5.50920000	1.03093300	1.75583500
H	-6.75936100	-0.61720300	0.36721100
C	-3.00762500	1.86803400	1.45532700
H	-3.63797300	2.40869800	2.16302500
N	-1.70535700	2.00222200	1.51140800
C	-1.09379200	2.84615600	2.40308100
C	-1.89141300	3.74288700	3.30267400
H	-1.23225800	4.37915900	3.89368000
H	-2.56868800	4.39080100	2.72452200
H	-2.52547500	3.17261600	4.00036900
C	0.31137800	2.77922600	2.44759800
O	1.04698400	2.02494100	1.73885800
O	0.91139600	3.61733900	3.34611900
C	2.33277400	3.55509500	3.39613800
H	2.63178400	4.29545200	4.14077100
H	2.67766800	2.56057500	3.69908100
H	2.77755300	3.79620600	2.42560000
C	0.43448700	0.37328200	-2.45190700
O	0.23808900	0.62733300	-3.76869500
C	-0.53873000	1.85082600	-3.84244600
H	-0.03964700	2.51512200	-4.55065400
H	-1.53092100	1.59095900	-4.22178500
C	-0.55228300	2.38581100	-2.38844000
H	-1.57505700	2.56506600	-2.03751000
C	0.25779200	3.68632500	-2.21841900
H	1.24249600	3.51399800	-2.68167800
C	-0.44241300	4.83045100	-2.96543400
H	0.14865700	5.74976100	-2.89850400
H	-0.59658500	4.61334800	-4.02910100
H	-1.42410700	5.03271000	-2.51882800
C	0.46717200	4.05405500	-0.74828100
H	1.05084000	3.30237100	-0.21503400

H	0.98741300	5.01616400	-0.67148900
H	-0.49178600	4.14780300	-0.22520900
F	-5.79764000	-1.81199800	-1.73036500

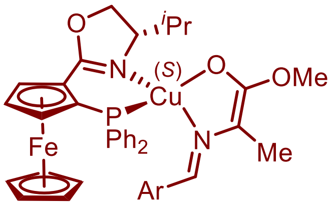

Cu(*S*)-Nu

Total SCF energy (M06-2X/def2TZVP/SMD(THF)): -5197.173440 a.u.

Thermal correction to Gibbs Free Energy at 298.15 K: 0.614379 a.u

Gibbs free energy at 298.15 K (M06-2X/def2TZVP/SMD(THF)): -5196.559061 a.u.

Fe	-2.74208100	1.30843300	-1.23791100
P	0.38518800	1.36804900	0.22679200
N	0.23855500	-1.09637500	-2.02771200
C	-1.00905800	1.04964300	-2.29499200
C	-1.95895200	1.76646800	-3.09934400
H	-2.36568300	1.41478700	-4.03541100
C	-2.29896100	2.96618100	-2.42226500
H	-3.03141500	3.69115800	-2.75030300
C	-1.57107500	3.00899800	-1.19986700
H	-1.65158300	3.77036800	-0.43810900
C	-0.75988700	1.83408900	-1.09980700
C	-3.83831000	1.22925400	0.51330000
H	-3.64673100	1.87027000	1.36266200
C	-3.21309500	-0.02900700	0.26819600
H	-2.48536200	-0.51343700	0.89758200
C	-3.67930300	-0.51749000	-0.98760000
H	-3.35055300	-1.42709200	-1.47201200
C	-4.59539700	0.43670300	-1.52268000
H	-5.08830200	0.37784000	-2.48365300
C	-4.69388100	1.51723100	-0.59260800
H	-5.27465600	2.41945700	-0.72959300
C	-0.47495000	1.89408400	1.75652600
C	-0.96570800	0.89108400	2.60209000
H	-0.77511800	-0.14914100	2.36549500
C	-1.68969700	1.22472800	3.74727200
H	-2.06852200	0.43350600	4.38771100
C	-1.91453200	2.56577900	4.06308000
H	-2.47767900	2.82915700	4.95406900
C	-1.39443700	3.57285100	3.24336200
H	-1.54679000	4.61713000	3.50216200
C	-0.67008900	3.23959400	2.09921200
H	-0.24126400	4.02233200	1.47983500
C	1.76596200	2.56422200	0.08117100
C	1.77943500	3.65766500	-0.79237900
H	0.91923100	3.86144200	-1.42167700
C	2.90504800	4.48186900	-0.86886800
H	2.90743000	5.32671800	-1.55231200
C	4.02238000	4.22065000	-0.07566200
H	4.89758300	4.86116400	-0.13998800

C	4.01703500	3.12580400	0.79374000
H	4.88960800	2.90391400	1.40140100
C	2.89940100	2.29868700	0.86743100
H	2.91386500	1.42603300	1.51482100
Cu	1.12683100	-0.72119200	-0.19963400
C	-2.72281300	-3.20855000	2.54053700
C	-2.02781100	-3.04980500	3.73544600
C	-0.67481300	-2.72702200	3.68989400
C	0.00543500	-2.54074100	2.46537100
C	-0.73893100	-2.72404100	1.27824600
C	-2.08977300	-3.05756300	1.31181900
H	-2.54802800	-3.18204700	4.67864600
H	-0.12636500	-2.59822800	4.61955400
H	-0.24555300	-2.61851600	0.31968900
H	-2.65705200	-3.20453900	0.39839100
C	1.40914900	-2.17697100	2.47978500
H	1.93685300	-2.32414100	3.42273400
N	2.03508000	-1.67322500	1.43972800
C	3.36414200	-1.37551000	1.44509500
C	4.22081400	-1.64641400	2.64547200
H	5.25908200	-1.37938700	2.44752900
H	3.88388100	-1.07645400	3.52687500
H	4.18681900	-2.70909400	2.93287400
C	3.86970100	-0.80815100	0.24493400
O	3.20587500	-0.56544500	-0.79398700
O	5.20055100	-0.49400100	0.27830400
C	5.70370300	0.15304500	-0.88916400
H	6.76202200	0.33313300	-0.68861900
H	5.59129100	-0.48125300	-1.77372200
H	5.18469000	1.09994400	-1.06515200
C	-0.48479300	-0.25617500	-2.68691000
O	-0.80968800	-0.60888800	-3.96094200
C	-0.39318100	-1.98581600	-4.10738900
H	0.01866400	-2.11144400	-5.10864300
H	-1.28287500	-2.61774200	-3.99143200
C	0.61506700	-2.18741000	-2.96374000
H	1.63648400	-1.97019000	-3.30794500
C	0.60340900	-3.59487500	-2.34727200
H	-0.34206900	-3.70056700	-1.79481200
C	1.77804800	-3.80739400	-1.38519700
H	1.82316900	-3.05300000	-0.59842100
H	1.70430400	-4.79135200	-0.90866800
H	2.72822100	-3.76363200	-1.93155900
C	0.64525400	-4.66314300	-3.45212000
H	1.54623000	-4.54749100	-4.06811000
H	0.67663000	-5.66370600	-3.00865500
H	-0.22599400	-4.62497000	-4.11556300
F	-4.03920300	-3.52197600	2.57441100

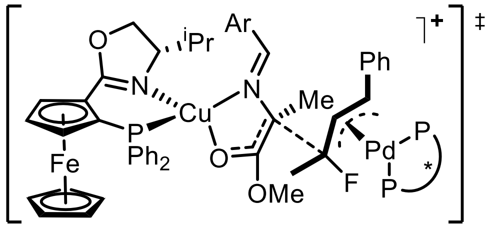

Total SCF energy (M06-2X/def2TZVP/SMD(THF)): -9363.130578 a.u.

Thermal correction to Gibbs Free Energy at 298.15 K: 2.200618 a.u

Gibbs free energy at 298.15 K (M06-2X/def2TZVP/SMD(THF)): -9360.92996 a.u.

Pd	-1.88360900	0.19479700	-0.59241600
P	-2.17555000	-2.01501200	0.18189600
P	-3.85075900	1.04396500	0.31659000
O	-7.08853400	-1.71330200	1.75917600
O	-4.88897400	-2.89161700	3.49742400
C	-1.26809700	-3.15492900	-0.93344700
C	-0.27497200	-4.03619000	-0.49217000
H	-0.11729400	-4.15915300	0.56951700
C	0.54005300	-4.71834200	-1.40478700
C	0.31000200	-4.51533200	-2.77220400
C	-0.69783400	-3.66706200	-3.25118300
C	-1.46577600	-2.97461400	-2.30949600
H	-2.22225700	-2.26974700	-2.63876200
C	1.70741500	-5.59822400	-0.93429200
C	1.48396500	-6.11167300	0.49975800
H	0.54396500	-6.66913400	0.58449600
H	2.30250000	-6.78059300	0.78608300
H	1.46663900	-5.29482900	1.22448800
C	2.99231600	-4.73964700	-0.96580900
H	2.90224200	-3.87276400	-0.30447400
H	3.85817700	-5.32527500	-0.63533200
H	3.19835000	-4.37395500	-1.97705300
C	1.89277200	-6.82417700	-1.85118800
H	2.18959700	-6.54419000	-2.86650000
H	2.68423500	-7.46750300	-1.45184900
H	0.97265600	-7.41548800	-1.91527400
C	-1.00208900	-3.52080000	-4.74926100
C	-1.14879900	-2.03423700	-5.13280000
H	-0.21734700	-1.48894400	-4.94750400
H	-1.38593000	-1.94091100	-6.19834300
H	-1.94952800	-1.54065400	-4.57718700
C	-2.33069700	-4.25463200	-5.04016900
H	-3.15466000	-3.82763900	-4.46026000
H	-2.58804300	-4.17648600	-6.10303200
H	-2.25085500	-5.31673200	-4.78415900
C	0.10132400	-4.12992200	-5.63096900
H	0.19042800	-5.21185600	-5.48895100
H	-0.13390700	-3.95747800	-6.68636200
H	1.07754100	-3.67477600	-5.42558800
C	-1.61304700	-2.38320400	1.88076800
C	-1.07024300	-1.33220700	2.62974700
H	-1.02542000	-0.34932600	2.17513100
C	-0.63161500	-1.54220300	3.94180500
C	-0.70616800	-2.84665600	4.44883000

C	-1.22826200	-3.92615500	3.72077200
C	-1.71647100	-3.66515400	2.43491900
H	-2.16759200	-4.45467400	1.84680100
C	-1.16568800	-5.34754300	4.30196000
C	-1.72566200	-5.39327500	5.73850100
H	-2.78752500	-5.13188200	5.75734300
H	-1.62223500	-6.40482200	6.14640700
H	-1.19717100	-4.71271200	6.41275300
C	0.31784600	-5.78207000	4.33235700
H	0.92052100	-5.10142900	4.94318700
H	0.41384100	-6.79086700	4.75028100
H	0.73759300	-5.79226900	3.32123500
C	-1.95299500	-6.35500400	3.44704000
H	-1.53121800	-6.45443200	2.44101300
H	-1.91852300	-7.34403800	3.91553100
H	-3.00521800	-6.06465700	3.34914000
C	-0.07907400	-0.41196700	4.82185000
C	-0.95264900	-0.28468000	6.08888700
H	-0.94470300	-1.20726400	6.67876700
H	-0.57480200	0.52259900	6.72712300
H	-1.98835800	-0.06128400	5.82390000
C	-0.08736500	0.93985200	4.08710200
H	-1.09969400	1.21556700	3.77230100
H	0.28372800	1.72425800	4.75271100
H	0.56318700	0.93186000	3.20550000
C	1.36683200	-0.74077100	5.24977800
H	2.02676300	-0.82009700	4.38349000
H	1.75073900	0.04912900	5.90491000
H	1.42006500	-1.68572400	5.80030800
C	-5.34414000	0.64701600	-0.64319800
C	-6.61952800	0.83704700	-0.09851900
H	-6.70762500	1.18801800	0.92146700
C	-7.75969700	0.55372000	-0.85197900
C	-7.57661400	0.04582500	-2.14600100
C	-6.31448000	-0.19780600	-2.69985400
C	-5.19291300	0.13330300	-1.93263200
H	-4.18976400	-0.01449200	-2.31261700
C	-3.87927100	2.85791500	0.54829600
C	-4.97829600	3.64092600	0.17749600
H	-5.84997700	3.15703700	-0.23656100
C	-4.94915800	5.02896000	0.31955500
C	-3.78568800	5.61430700	0.84349100
C	-2.66603900	4.86218200	1.21402000
C	-2.72718500	3.47183800	1.04740600
H	-1.86526300	2.85452600	1.28534100
C	-9.17788500	0.77239700	-0.31099700
C	-9.88295100	1.84730000	-1.16604600
H	-9.94568400	1.54862800	-2.21767400
H	-10.90336300	2.01610800	-0.80259700
H	-9.34139200	2.79850300	-1.11959900
C	-9.16835000	1.23613700	1.15510600
H	-8.67040000	2.20573600	1.27056300
H	-10.19704900	1.35068000	1.51298700
H	-8.65994200	0.51035400	1.79796800
C	-9.96707000	-0.55155300	-0.39342700
H	-9.45968600	-1.33200900	0.18094800
H	-10.97559900	-0.42093700	0.01524600

H	-10.06905000	-0.90416900	-1.42446200
C	-6.19347100	-0.79111200	-4.10861800
C	-6.60786800	0.28399400	-5.13600000
H	-5.96530600	1.16662800	-5.04912000
H	-6.52616800	-0.10661900	-6.15757100
H	-7.64341400	0.60401400	-4.97799700
C	-4.75376800	-1.23875100	-4.40856200
H	-4.40991100	-1.96791200	-3.66608300
H	-4.70672300	-1.71180000	-5.39523500
H	-4.05993900	-0.39391700	-4.41105500
C	-7.10844300	-2.02633600	-4.24515300
H	-8.16612200	-1.77438900	-4.12308600
H	-6.99039300	-2.47399500	-5.23854900
H	-6.85195000	-2.78043400	-3.49348700
C	-6.14391100	5.91278500	-0.06354900
C	-5.70854400	6.93933800	-1.13099500
H	-5.37874900	6.43275200	-2.04450000
H	-6.54680100	7.59556300	-1.39130200
H	-4.88666000	7.57104100	-0.77928700
C	-7.31250300	5.09057800	-0.63384400
H	-7.69500700	4.36948900	0.09718000
H	-8.13705800	5.75970200	-0.90124400
H	-7.02164700	4.54065900	-1.53571700
C	-6.64144300	6.65846400	1.19422800
H	-5.86268500	7.29904700	1.62060900
H	-7.49898300	7.29394300	0.94516000
H	-6.95454900	5.94905000	1.96822800
C	-1.37479400	5.50699800	1.73469700
C	-0.26344600	5.26963100	0.68944500
H	-0.56222500	5.66270600	-0.28844200
H	0.66203300	5.77720500	0.98655100
H	-0.04457800	4.20280400	0.57455600
C	-0.97281300	4.86225800	3.07854900
H	-0.77039000	3.79164400	2.97866400
H	-0.06436100	5.33499700	3.46922400
H	-1.76693700	4.99049700	3.82229300
C	-1.52119700	7.02216500	1.95048300
H	-0.58520200	7.42937900	2.34743300
H	-1.73840700	7.54889100	1.01500500
H	-2.31609000	7.25308900	2.66793800
C	-4.86073600	-1.94688500	0.99904100
C	-3.93164100	-2.54788600	0.12509900
C	-4.36948600	-3.46053600	-0.84475300
H	-3.65354700	-3.93711300	-1.50265700
C	-5.72169900	-3.77173300	-0.96088400
H	-6.05094100	-4.47844500	-1.71710700
C	-6.65271600	-3.19024800	-0.10509700
C	-6.21892400	-2.30069000	0.87835800
C	-8.05620200	-2.56659800	2.36503600
H	-7.57542900	-3.46725800	2.76161100
H	-8.49468100	-1.98933600	3.18128100
C	-4.18013700	0.33738300	1.98249100
C	-3.98667900	1.13785100	3.11939600
H	-3.75850700	2.19043800	3.00704800
C	-4.11119400	0.59151400	4.39048900
H	-3.98211800	1.22283600	5.26477200
C	-4.40096700	-0.76145000	4.55706400

C	-4.59430300	-1.56275400	3.43020300
C	-4.50089400	-1.02720700	2.12666200
C	-4.94074900	-3.49641800	4.77865300
H	-5.11880800	-4.55807800	4.60032600
H	-5.75825700	-3.08554800	5.38476700
H	-8.85280100	-2.85050700	1.66778600
H	-4.48261200	-1.17428500	5.55443300
H	-3.99172600	-3.36938900	5.31107400
H	-7.70720300	-3.42389800	-0.19874200
H	-0.33000600	-3.03175000	5.44919700
H	-8.45531300	-0.18124100	-2.73992100
H	0.93756800	-5.03266900	-3.48431000
H	-3.75718200	6.69053700	0.95767800
C	1.44364200	-1.20062600	-1.24060700
H	1.28224100	-1.76502600	-2.16066400
H	2.49958500	-1.21732100	-0.98254200
C	0.94837900	0.19528700	-1.36888300
C	-0.25002400	0.55647900	-2.01999800
H	-0.48675200	-0.06534800	-2.87522300
C	-0.98100100	1.76144700	-1.78024600
H	-0.55288800	2.46475100	-1.07178600
C	-1.91656400	2.35177800	-2.75086600
C	-2.26511800	1.71956300	-3.95856900
C	-2.51011400	3.59546800	-2.46504100
C	-3.18918800	2.29362200	-4.82839800
H	-1.80774600	0.77310500	-4.22660800
C	-3.43932900	4.16413700	-3.33152700
H	-2.25776600	4.10245200	-1.53988700
C	-3.79111100	3.51530900	-4.51711300
H	-3.43739400	1.78587500	-5.75631500
H	-3.88516600	5.12158900	-3.07947700
H	-4.51315100	3.96010600	-5.19524600
F	1.31877100	0.97114300	-0.31135300
H	0.89356600	-1.70822300	-0.44649500
Fe	7.41721100	2.37133800	1.75122800
P	4.62643400	0.49063200	1.27158000
N	6.64419300	0.74235900	-1.42093000
C	7.51260400	0.53241400	0.88436600
C	8.69651900	0.72269400	1.66855800
H	9.70178000	0.74533200	1.27401800
C	8.30614200	0.94701200	3.01375100
H	8.97036500	1.18126400	3.83423300
C	6.88810400	0.88109000	3.08481900
H	6.29481500	1.05119700	3.96947700
C	6.36547900	0.63074400	1.77339500
C	7.72280900	4.26805000	2.52630000
H	8.10804800	4.45637900	3.51918600
C	6.34995900	4.08837300	2.17992600
H	5.51095500	4.11168000	2.86074100
C	6.28274800	3.79981800	0.78181100
H	5.39117800	3.57005200	0.21592600
C	7.61380700	3.80030700	0.26922300
H	7.89486100	3.56132100	-0.74727900
C	8.50495300	4.08977100	1.34500500
H	9.58446700	4.11992200	1.28662000
C	3.74325900	1.64963900	2.37736800
C	3.13317700	2.77627800	1.80988800

H	3.12290500	2.89438100	0.73091100
C	2.54762200	3.73929000	2.63405100
H	2.08487000	4.61389600	2.19156500
C	2.56102900	3.58279200	4.02042600
H	2.11008600	4.33785900	4.65770500
C	3.13505400	2.44171800	4.58723500
H	3.12435300	2.30302200	5.66430800
C	3.71122700	1.47273300	3.76846400
H	4.12770600	0.57261800	4.20876400
C	4.12017600	-1.15506600	1.90589800
C	5.05443200	-2.12102600	2.30284100
H	6.11322600	-1.88097300	2.30424400
C	4.62666500	-3.38478600	2.71577500
H	5.35795700	-4.12397600	3.03004700
C	3.26607500	-3.69179800	2.74052200
H	2.93542500	-4.67005200	3.07386800
C	2.32848700	-2.73665100	2.33836100
H	1.26821900	-2.96209900	2.36834300
C	2.75261300	-1.47693700	1.91859900
H	2.02001700	-0.73668700	1.61132800
Cu	4.63350900	0.85921500	-0.94965800
C	6.80072400	-3.88120400	-2.09649200
C	6.21092200	-3.08889100	-1.11507700
C	5.26212500	-2.14879900	-1.49533400
C	4.90092200	-1.97893900	-2.84542100
C	5.49538000	-2.82271500	-3.80336700
C	6.45189800	-3.76651800	-3.43962200
H	6.47175700	-3.23281900	-0.07265900
H	4.77299200	-1.55691100	-0.73318500
H	5.21473800	-2.72277600	-4.84873400
H	6.92153600	-4.41365700	-4.17230300
C	3.92377800	-0.98800100	-3.28243700
H	3.43777500	-1.19226200	-4.23837400
N	3.61415800	0.07739900	-2.60223400
C	2.55195000	0.89526000	-2.96030400
C	1.82866900	0.68012500	-4.26202300
H	0.98648700	1.36502400	-4.35306100
H	2.49405500	0.84413600	-5.12276100
H	1.44742600	-0.34419300	-4.33957800
C	2.62086300	2.22731600	-2.39421700
O	3.42001000	2.59723600	-1.51301900
O	1.67838200	3.05892900	-2.87223400
C	1.61654600	4.36528200	-2.28673300
H	0.71058500	4.81657700	-2.69097100
H	1.55755300	4.29570300	-1.19942700
H	2.49772100	4.95054400	-2.56370600
C	7.54976700	0.40513700	-0.56977400
O	8.70420400	-0.08751900	-1.06744000
C	8.52482100	-0.19140700	-2.50676400
H	9.39568700	0.26715600	-2.97807100
H	8.48302500	-1.25416700	-2.75763400
C	7.19467700	0.55051500	-2.77867000
H	6.50687900	-0.07862300	-3.34845100
C	7.36443500	1.88701700	-3.52605700
H	8.12785300	2.46999700	-2.98772600
C	7.86179400	1.62696700	-4.95434200
H	8.02037100	2.57122100	-5.48500700

H	8.80676700	1.07269500	-4.97853500
H	7.11957200	1.04801600	-5.51885200
C	6.06161500	2.69188000	-3.55014400
H	5.71588700	2.94410200	-2.54633600
H	6.19877600	3.62146400	-4.11374600
H	5.26347200	2.11783900	-4.03728400
F	7.71671900	-4.79746300	-1.73470000

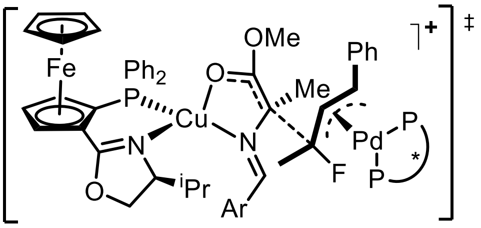

Cu-Nu(*re*-face) approach

Total SCF energy (M06-2X/def2TZVP/SMD(THF)): -9363.122885 a.u.

Thermal correction to Gibbs Free Energy at 298.15 K: 2.200913 a.u

Gibbs free energy at 298.15 K (M06-2X/def2TZVP/SMD(THF)): -9360.9219723 a.u.

Fe	-8.18278700	-1.57248300	-1.37706700
P	-6.58710900	0.75458700	0.59713700
N	-4.61183900	-0.57516500	-1.73742800
C	-7.01559600	-0.08306400	-2.13891200
C	-8.11043400	-0.31318100	-3.03671600
H	-8.01547200	-0.65292700	-4.05763400
C	-9.31832900	-0.08860800	-2.32542500
H	-10.31624700	-0.23981400	-2.71359900
C	-8.99082500	0.29646600	-0.99554100
H	-9.69067100	0.50165000	-0.19875400
C	-7.56749100	0.30035600	-0.85368400
C	-9.41626600	-3.18057300	-0.94895300
H	-10.49540200	-3.13843800	-1.00718900
C	-8.62470100	-2.84574100	0.19032800
H	-8.99532200	-2.49976200	1.14487400
C	-7.24863000	-2.97174500	-0.17309800
H	-6.39493200	-2.75334100	0.45298800
C	-7.19512500	-3.38302000	-1.53901500
H	-6.29456400	-3.52024100	-2.12136500
C	-8.53205100	-3.51223900	-2.01952900
H	-8.82363900	-3.76728300	-3.02931600
C	-7.64195800	0.27413000	2.01144100
C	-7.29086300	-0.87520800	2.73349100
H	-6.39285800	-1.42176700	2.46219100
C	-8.09442800	-1.31301600	3.78744900
H	-7.81957700	-2.20569500	4.34232400
C	-9.24455400	-0.60045000	4.13325200
H	-9.86804200	-0.93971300	4.95527500
C	-9.58549100	0.55909400	3.43140300
H	-10.47144800	1.12254900	3.70951800
C	-8.78553400	0.99866300	2.37713000
H	-9.03942600	1.91007400	1.84386600
C	-6.67444400	2.58496200	0.58736300
C	-7.15417700	3.30731500	-0.51161600

H	-7.57441500	2.78433400	-1.36438600
C	-7.09363800	4.70300400	-0.51661800
H	-7.46658000	5.25423900	-1.37514800
C	-6.55996700	5.38620100	0.57503700
H	-6.50579200	6.47066200	0.56614500
C	-6.08558300	4.66992500	1.67772900
H	-5.66083500	5.19593500	2.52711700
C	-6.13697500	3.27920400	1.68261200
H	-5.74729000	2.72697300	2.53333300
Cu	-4.55090600	-0.26045800	0.31079100
C	-3.19555500	5.01955400	-0.25918300
C	-3.58059600	3.81220800	-0.83218400
C	-3.39746200	2.63824300	-0.11407300
C	-2.86313000	2.66295500	1.18781200
C	-2.47950300	3.90540000	1.72773100
C	-2.64033100	5.08686100	1.01198600
H	-4.02317100	3.80954800	-1.82045900
H	-3.68166300	1.69119500	-0.56248000
H	-2.03987600	3.93922900	2.71964900
H	-2.34285700	6.04581100	1.42129200
C	-2.65611000	1.47984700	2.00781200
H	-2.09386000	1.65930400	2.92313900
N	-3.08492400	0.28118200	1.73040900
C	-2.70924400	-0.79240500	2.52418700
C	-2.10393500	-0.58092800	3.88802200
H	-1.83130100	-1.53632400	4.33713000
H	-1.21182600	0.04828000	3.85237800
H	-2.81950900	-0.08391400	4.56059700
C	-3.47874800	-1.99074200	2.29582500
O	-4.32900200	-2.14806300	1.39736900
O	-3.18850800	-3.00943500	3.13887700
C	-3.88352300	-4.23970600	2.89907500
H	-3.50390700	-4.93635600	3.64667700
H	-4.96241200	-4.10553800	3.01440700
H	-3.67901400	-4.61138300	1.89132200
C	-5.62310000	-0.31891900	-2.50189300
O	-5.37910100	-0.30187800	-3.83396100
C	-3.93394200	-0.36833700	-3.97371200
H	-3.71206000	-0.99806300	-4.83543900
H	-3.57404800	0.64726500	-4.14999500
C	-3.48231700	-0.95102100	-2.62453700
H	-2.56712400	-0.48567900	-2.25064500
C	-3.28775500	-2.48398300	-2.72813000
H	-4.10697700	-2.85952900	-3.36093800
C	-1.95316200	-2.77376500	-3.42653400
H	-1.83822100	-3.84570600	-3.61755700
H	-1.85098300	-2.25407600	-4.38610500
H	-1.12099600	-2.45670000	-2.78592400
C	-3.35246000	-3.21204700	-1.38667200
H	-4.29687300	-3.04229700	-0.87182000
H	-3.22578900	-4.29018800	-1.54166800
H	-2.55692700	-2.86385100	-0.72709500
F	-3.35468200	6.15691000	-0.96900600
Pd	1.91800900	-0.09468700	0.35583100
P	3.40401500	-1.93237300	0.29465500
P	3.22309000	1.50765800	-0.72391700
O	7.45753000	1.00611800	-0.76642400

O	6.82090900	-1.86698600	-2.16078400
C	2.91921200	-3.04515900	1.66818900
C	2.49292600	-4.36199300	1.44879900
H	2.58179500	-4.78290700	0.45722200
C	1.93033200	-5.11418200	2.48423900
C	1.84331700	-4.53063400	3.75773700
C	2.27978400	-3.22626000	4.01760100
C	2.78943400	-2.48532700	2.94487700
H	3.09146500	-1.45432200	3.09860600
C	1.38276100	-6.53032500	2.26111200
C	1.61905000	-7.02473000	0.82426800
H	2.68659600	-7.05252400	0.57969100
H	1.22603900	-8.04078100	0.71418200
H	1.11573500	-6.39212900	0.08469400
C	-0.13941600	-6.52203800	2.52906500
H	-0.65072700	-5.83760000	1.84230100
H	-0.55766100	-7.52521200	2.38657400
H	-0.36700300	-6.20385200	3.55172100
C	2.07294000	-7.51541800	3.22890200
H	1.90107700	-7.24871200	4.27628100
H	1.68763000	-8.52992100	3.07610100
H	3.15520200	-7.53204900	3.05966200
C	2.25158700	-2.60356200	5.42063100
C	1.52935300	-1.24093300	5.38182600
H	0.49490500	-1.35505900	5.04080300
H	1.50830500	-0.79364900	6.38196600
H	2.03587700	-0.53349200	4.71900700
C	3.70946000	-2.38945900	5.88618700
H	4.24987700	-1.71903300	5.21001300
H	3.72974600	-1.94581400	6.88848200
H	4.24971100	-3.34175300	5.92266700
C	1.53331500	-3.49844000	6.44338300
H	2.04024700	-4.46091200	6.56992400
H	1.51930900	-3.00375000	7.42009500
H	0.49449600	-3.69172700	6.15194900
C	3.62647700	-3.00819200	-1.16062300
C	2.91355400	-2.70863500	-2.32425900
H	2.28003700	-1.82965900	-2.32712600
C	3.04598300	-3.50910300	-3.46440200
C	3.87367000	-4.63569400	-3.37514900
C	4.59479400	-4.96988200	-2.21870100
C	4.48375400	-4.11478000	-1.11835600
H	5.04038000	-4.31025700	-0.21014300
C	5.40362100	-6.27504700	-2.17130700
C	6.41541800	-6.33662000	-3.33449300
H	7.16575800	-5.54539300	-3.24527200
H	6.93886700	-7.29921700	-3.32639300
H	5.92866400	-6.23337500	-4.30940700
C	4.41771600	-7.45859800	-2.29947500
H	3.87542900	-7.42890200	-3.25018000
H	4.95705800	-8.41144300	-2.24830300
H	3.67800500	-7.43980100	-1.49199600
C	6.17758100	-6.42666200	-0.85121900
H	5.50498300	-6.46906200	0.01280200
H	6.75386800	-7.35756200	-0.86623100
H	6.88053300	-5.60024300	-0.69674100
C	2.30061800	-3.21279400	-4.77221600

C	3.30696500	-3.14395400	-5.94089100
H	3.84475000	-4.08761600	-6.07559200
H	2.78340300	-2.92440400	-6.87845500
H	4.04495600	-2.35503000	-5.76567400
C	1.54923800	-1.87342400	-4.71294100
H	2.23295100	-1.04140600	-4.51695900
H	1.05162400	-1.68719800	-5.67095200
H	0.77728600	-1.87274500	-3.93771500
C	1.28046800	-4.34182300	-5.03448100
H	0.55710900	-4.40899400	-4.21462800
H	0.72968200	-4.15219100	-5.96332500
H	1.77285200	-5.31538900	-5.12828500
C	4.37233200	2.44649400	0.33434300
C	5.20674300	3.43669000	-0.19966300
H	5.18229200	3.62492700	-1.26571100
C	6.03993300	4.18852900	0.63525800
C	6.06725100	3.85310900	1.99687000
C	5.28840900	2.82847700	2.55043800
C	4.41153600	2.14893300	1.69952800
H	3.76041700	1.36921600	2.07397900
C	2.19384800	2.72582900	-1.61962200
C	2.26294700	4.10428100	-1.39983600
H	2.99892800	4.49015400	-0.70967800
C	1.36364400	4.97548900	-2.03068800
C	0.43067100	4.42742500	-2.91584800
C	0.35163500	3.05233000	-3.18063600
C	1.21815100	2.20710800	-2.48655100
H	1.15837400	1.13277400	-2.61875700
C	6.83154300	5.40418400	0.12838600
C	6.19602400	6.67175400	0.74514600
H	6.25810300	6.65656700	1.83829200
H	6.71236900	7.57074300	0.38870200
H	5.13908800	6.75132200	0.47121900
C	6.78278500	5.52703600	-1.40388400
H	5.76207800	5.68502300	-1.76929400
H	7.38259400	6.38495900	-1.72505500
H	7.18525400	4.63311000	-1.89400800
C	8.31143500	5.33103100	0.55752300
H	8.81689400	4.48012300	0.09175700
H	8.83539600	6.24232000	0.24824000
H	8.42371100	5.23962300	1.64219400
C	5.40291000	2.50747800	4.04684400
C	4.93004200	3.72982700	4.86279500
H	3.89772400	3.98673200	4.60919900
H	4.98326100	3.51577400	5.93690700
H	5.55383100	4.60816300	4.66576200
C	4.54888900	1.28861100	4.43689500
H	4.85003500	0.39878700	3.87346900
H	4.67658800	1.07295600	5.50329000
H	3.48525800	1.46988100	4.25763700
C	6.87302200	2.19536400	4.39964800
H	7.53273200	3.04250900	4.18646000
H	6.96397900	1.96492300	5.46746700
H	7.23187400	1.33415300	3.82884600
C	1.35934500	6.48832100	-1.77169800
C	0.01516700	6.87832600	-1.11691500
H	-0.12643400	6.35137300	-0.16761700

H	-0.01311600	7.95600400	-0.91790400
H	-0.84004100	6.63289900	-1.75347200
C	2.50761900	6.91961100	-0.84430100
H	3.48407300	6.68307900	-1.28138200
H	2.46845200	8.00239700	-0.68619100
H	0.96453300	5.57745800	1.60078300
C	1.51867800	7.24568600	-3.10797100
H	0.70111800	7.02858700	-3.80229500
H	1.52477900	8.32716200	-2.93096500
H	2.45942400	6.97450000	-3.59976700
C	-0.63607100	2.54004400	-4.23668800
C	-2.07163000	2.94001700	-3.84599300
H	-2.16910700	4.01854500	-3.69097600
H	-2.77643300	2.65565700	-4.63660300
H	-2.36659100	2.44637100	-2.91504900
C	-0.57647300	1.01236700	-4.38983000
H	-0.82911700	0.49844000	-3.45573800
H	-1.28834100	0.68792000	-5.15679500
H	0.41621600	0.67585600	-4.70234100
C	-0.27595200	3.17463300	-5.59847500
H	-0.95770100	2.81577800	-6.37848400
H	-0.34607900	4.26624600	-5.56715800
H	0.74693300	2.91273600	-5.89065800
C	5.70896400	-0.44776500	-0.21087800
C	5.09048800	-1.32494600	0.69819800
C	5.70240900	-1.61275000	1.92695400
H	5.24378800	-2.31193500	2.61420800
C	6.91043400	-1.00956100	2.26167500
H	7.38439100	-1.24120500	3.21122200
C	7.52037400	-0.10685900	1.39339900
C	6.92584100	0.16395100	0.15775400
C	8.66188800	1.67920600	-0.43998400
H	9.49007700	0.97303700	-0.29947700
H	8.87879300	2.32858900	-1.28929600
C	4.27980000	0.72454400	-2.01174300
C	4.03949800	0.95425400	-3.37561700
H	3.31745000	1.69978300	-3.68186500
C	4.73420400	0.23282500	-4.34083100
H	4.54915100	0.42327700	-5.39423600
C	5.66414500	-0.73659300	-3.97276400
C	5.92217100	-0.95565900	-2.61701200
C	5.24561900	-0.22090700	-1.61843800
C	7.48191300	-2.68770800	-3.10949000
H	8.11021500	-3.36418400	-2.52842400
H	8.11298700	-2.09459500	-3.78343700
H	8.53907100	2.28764000	0.46272700
H	6.18747900	-1.29913600	-4.73545900
H	6.76148700	-3.27035300	-3.69476900
H	8.45403200	0.36314800	1.67504900
H	3.95248600	-5.28827600	-4.23847000
H	6.71457500	4.42210800	2.65595500
H	1.41838500	-5.11310200	4.56525100
H	-0.25844200	5.09463100	-3.42228800
C	-0.57933200	-2.74724000	1.42643200
H	-0.25762100	-2.91503000	2.45526600
H	-1.50408600	-3.29503200	1.24597400
C	-0.74076000	-1.29142200	1.15471200

C	0.06867500	-0.28188500	1.68300000
H	0.50713000	-0.52056400	2.64422400
C	0.19671700	1.03878300	1.14210700
H	-0.39142600	1.27297100	0.25936300
C	0.59713100	2.20215500	1.94742600
C	0.93611700	2.10972700	3.30974800
C	0.60246600	3.47677200	1.35371600
C	1.28061200	3.24216300	4.04309700
H	0.91705300	1.14785400	3.80921400
C	0.96815000	4.60515800	2.08252400
H	0.30779900	3.57509300	0.31612400
C	1.30922500	4.49813600	3.43178700
H	1.52680800	3.14254500	5.09636400
H	2.45072500	6.43751700	0.13703400
H	1.58211400	5.38063100	4.00250300
F	-1.32212300	-1.03193200	-0.04556100
H	0.19469900	-3.15647500	0.76803600

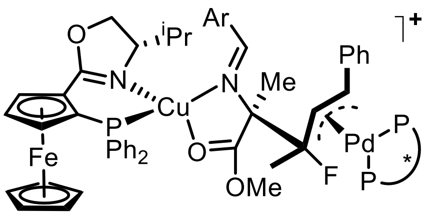

Int-6

Total SCF energy (M06-2X/def2TZVP/SMD(THF)): -9363.162438 a.u.

Thermal correction to Gibbs Free Energy at 298.15 K: 2.200214 a.u

Gibbs free energy at 298.15 K (M06-2X/def2TZVP/SMD(THF)): -9360.962224 a.u.

Pd	1.94847900	-0.17652800	-0.68758800
P	2.33195400	2.02459700	0.05027400
P	3.77523300	-1.11235400	0.44957700
O	7.09103700	1.45288200	2.01831900
O	4.89138700	2.85030800	3.56007200
C	1.59879000	3.23387100	-1.12629500
C	0.62424100	4.17083300	-0.76445100
H	0.41329800	4.32682100	0.28364800
C	-0.11188400	4.85979000	-1.73650500
C	0.19150000	4.62296500	-3.08476200
C	1.19127400	3.72573600	-3.48325600
C	1.86720300	3.01858200	-2.48408300
H	2.59547300	2.26150900	-2.75648500
C	-1.28098700	5.78057300	-1.35358700
C	-1.14169500	6.30409100	0.08741900
H	-0.19318400	6.83381000	0.23104200
H	-1.95713000	7.00026200	0.31137500
H	-1.19570300	5.49558500	0.82012100
C	-2.59019400	4.96270800	-1.45983800
H	-2.57803800	4.11374100	-0.76937100
H	-3.45772000	5.58685000	-1.21296700

H	-2.72965500	4.57308100	-2.47453800
C	-1.36655500	7.00108500	-2.29222900
H	-1.59872400	6.71844400	-3.32351800
H	-2.16316000	7.67352600	-1.95544600
H	-0.42589900	7.56244100	-2.29728600
C	1.57067100	3.51025400	-4.95543100
C	1.55448600	2.00499000	-5.29529600
H	0.55511700	1.58079500	-5.14401500
H	1.83228900	1.84924400	-6.34384200
H	2.25935100	1.43854500	-4.68135900
C	2.99579500	4.06482900	-5.17590200
H	3.72362400	3.55666900	-4.53646700
H	3.30547700	3.92456700	-6.21829700
H	3.03404800	5.13572500	-4.94837000
C	0.61133100	4.22794500	-5.91896100
H	0.62957200	5.31398800	-5.77994000
H	0.90702800	4.02400600	-6.95342200
H	-0.42190600	3.88334300	-5.79234500
C	1.68567900	2.47116400	1.70416400
C	1.00337600	1.48545400	2.42768300
H	0.90507900	0.50270000	1.98101700
C	0.48441700	1.76131700	3.69830600
C	0.63362200	3.06479700	4.18978200
C	1.29745100	4.08041400	3.48634900
C	1.85346700	3.75225000	2.24468400
H	2.41034900	4.48864900	1.67854800
C	1.31502200	5.51386000	4.04040200
C	1.76292700	5.54225600	5.51609200
H	2.79200800	5.18810500	5.62317000
H	1.71936300	6.56788700	5.89932100
H	1.12465500	4.92580100	6.15618000
C	-0.12205800	6.07641800	3.94792800
H	-0.82613500	5.46298100	4.52052500
H	-0.16169500	7.09879300	4.34175700
H	-0.46087200	6.10026100	2.90699900
C	2.25249500	6.43060700	3.23636000
H	1.91932600	6.54655500	2.19932400
H	2.27221800	7.42811300	3.68777500
H	3.27801400	6.04465500	3.22341800
C	-0.22224900	0.70115200	4.55662600
C	0.60721400	0.46269300	5.83701900
H	0.70382100	1.38024800	6.42757000
H	0.12178300	-0.29281500	6.46651400
H	1.61133000	0.11488400	5.58722400

C	-0.36453700	-0.63724900	3.80981800
H	0.61452100	-1.05023500	3.54237500
H	-0.87570700	-1.36546400	4.44682400
H	-0.95616800	-0.53169300	2.89443000
C	-1.62476200	1.19462800	4.97157200
H	-2.26651300	1.34780200	4.10212500
H	-2.09799500	0.45267400	5.62329700
H	-1.57572800	2.13780500	5.52483500
C	5.37277000	-0.89966700	-0.39878400
C	6.58993700	-1.16409600	0.23821100
H	6.58244300	-1.47478500	1.27497300
C	7.79671100	-0.99535700	-0.44325800
C	7.74211600	-0.52162400	-1.76213200
C	6.54227600	-0.19911400	-2.40760600
C	5.35079800	-0.41984500	-1.71028700
H	4.38876100	-0.20471500	-2.15897000
C	3.60430900	-2.91147800	0.74810200
C	4.65645800	-3.81620800	0.59836700
H	5.64055400	-3.44082600	0.35919800
C	4.44234500	-5.19373900	0.72003600
C	3.13896800	-5.63394300	0.98093000
C	2.05573700	-4.75291400	1.11764900
C	2.31216200	-3.38428000	1.01238800
H	1.50576300	-2.66285200	1.07680000
C	9.15292200	-1.30530900	0.20243200
C	9.80504900	-2.48796500	-0.54562100
H	9.95444700	-2.25932100	-1.60610400
H	10.78286000	-2.72560400	-0.11009000
H	9.17508000	-3.38225000	-0.48081800
C	9.01023200	-1.67973300	1.68723600
H	8.42701500	-2.59819700	1.81928300
H	10.00063700	-1.85446800	2.12116800
H	8.52277600	-0.87939600	2.25339000
C	10.06710900	-0.06556200	0.10504100
H	9.60028300	0.79071300	0.60100900
H	11.03057300	-0.26273100	0.58908800
H	10.26833100	0.21591300	-0.93317900
C	6.55556400	0.36160100	-3.83520200
C	6.91977100	-0.77741800	-4.81131400
H	6.19092900	-1.59164100	-4.74084900
H	6.93343600	-0.41080100	-5.84514500
H	7.90933700	-1.18937500	-4.58512000
C	5.18309900	0.93661700	-4.22700200
H	4.87090500	1.71340800	-3.51960100

H	5.23875800	1.38635000	-5.22440300
H	4.41110500	0.16296900	-4.25299000
C	7.59139100	1.49962200	-3.95051400
H	8.61215300	1.15125900	-3.76642200
H	7.56846000	1.92857000	-4.95895100
H	7.37092100	2.29543700	-3.23115100
C	5.57719600	-6.21215800	0.54498400
C	5.26757200	-7.10851200	-0.67368000
H	5.20461600	-6.50744700	-1.58762100
H	6.05798200	-7.85562300	-0.81041800
H	4.31852600	-7.64130100	-0.55382900
C	6.93695300	-5.53090900	0.30961800
H	7.21696600	-4.88511400	1.14962300
H	7.71609500	-6.29290800	0.20196100
H	6.93687900	-4.92398500	-0.60237700
C	5.68572600	-7.08405500	1.81438400
H	4.76027200	-7.63487300	2.01100800
H	6.49308600	-7.81705500	1.70407300
H	5.90227500	-6.46649100	2.69307300
C	0.63779300	-5.30478100	1.31437100
C	0.33115700	-6.33202900	0.20178200
H	0.99960600	-7.19670100	0.24511900
H	-0.69611800	-6.70362700	0.29877200
H	0.44306800	-5.87720900	-0.78864700
C	-0.42690800	-4.19770300	1.24251800
H	-0.37050600	-3.63088200	0.30775500
H	-1.42276600	-4.64910100	1.29429100
H	-0.34412100	-3.48403000	2.06852600
C	0.53866500	-5.99269500	2.69185900
H	-0.46326200	-6.41409500	2.84205900
H	1.26307200	-6.80833100	2.78484500
H	0.73422000	-5.27607000	3.49721300
C	4.94423900	1.81278100	1.09229500
C	4.12428800	2.43669600	0.12818800
C	4.69929900	3.27386200	-0.83929400
H	4.07002200	3.77304400	-1.56523700
C	6.07489600	3.48414400	-0.86868000
H	6.50673600	4.13312900	-1.62519000
C	6.89895200	2.87477700	0.07293800
C	6.33136400	2.06219500	1.05426500
C	8.10672600	2.24322400	2.62688600
H	7.70502200	3.21661000	2.92952800
H	8.43263900	1.68611700	3.50755900
C	4.05589100	-0.37900200	2.11061900

C	3.75079900	-1.12676900	3.25866200
H	3.45987100	-2.16618400	3.16235800
C	3.84796300	-0.54730700	4.51745600
H	3.63907100	-1.13952000	5.40386300
C	4.21505200	0.79052600	4.65745900
C	4.51364700	1.54021500	3.51807700
C	4.45272500	0.96717500	2.22907400
C	4.96442000	3.47855300	4.82743900
H	5.22990800	4.51873600	4.63119700
H	5.73341500	3.01804600	5.46116500
H	8.96929100	2.39133000	1.96670600
H	4.27358600	1.23005300	5.64505500
H	3.99834500	3.44000500	5.34321800
H	7.97191800	3.02721500	0.04630100
H	0.20541800	3.30241800	5.15753900
H	8.67406400	-0.38374900	-2.29973800
H	-0.37142000	5.14860100	-3.84377400
H	2.95655000	-6.70085500	1.05873400
C	-1.19459700	1.43024500	-1.56866200
H	-0.99342800	1.97390400	-2.49592400
H	-2.19286500	1.67973200	-1.21745700
C	-1.00813800	-0.06467700	-1.74869800
C	0.39390700	-0.41020100	-2.15507200
H	0.70742700	0.15180300	-3.02998500
C	0.99238100	-1.67399100	-1.90600900
H	0.47823900	-2.34237000	-1.22164100
C	1.95923600	-2.34114200	-2.79685000
C	2.41387600	-1.76742000	-3.99825400
C	2.45604500	-3.61319700	-2.45273400
C	3.34559500	-2.42194500	-4.80182900
H	2.03522300	-0.79945100	-4.30942300
C	3.39066700	-4.26431900	-3.25308300
H	2.12778300	-4.07567000	-1.52830400
C	3.84839700	-3.67170000	-4.43253800
H	3.67904300	-1.95300900	-5.72377200
H	3.76011400	-5.24081700	-2.95228500
H	4.57619000	-4.17871500	-5.05898900
F	-1.30098900	-0.67309700	-0.49534200
H	-0.47240700	1.76853200	-0.83235900
Fe	-7.69608400	-1.88828000	1.77625800
P	-4.65417900	-0.36929100	1.31395900
N	-6.40615200	-0.89613400	-1.52899800
C	-7.47922200	-0.23531700	0.60649600
C	-8.74688300	-0.15817500	1.27308400

H	-9.70607500	-0.14876700	0.77698800
C	-8.51393100	-0.17558900	2.67150100
H	-9.27506900	-0.18688200	3.43942100
C	-7.11093600	-0.25099200	2.88976200
H	-6.62658700	-0.32320400	3.85107500
C	-6.44145100	-0.29163700	1.62202000
C	-8.30314700	-3.56476900	2.83326300
H	-8.79235600	-3.52441100	3.79692300
C	-6.89433300	-3.60026500	2.61025800
H	-6.12591200	-3.58668100	3.37006500
C	-6.67350500	-3.58210800	1.19865900
H	-5.71400100	-3.55161800	0.70337700
C	-7.94502600	-3.53669800	0.55378800
H	-8.10853800	-3.46473000	-0.51263800
C	-8.95330000	-3.52496000	1.56266800
H	-10.01919000	-3.45006400	1.39595100
C	-4.03233400	-1.43505000	2.66440300
C	-3.52703000	-2.70120200	2.34450400
H	-3.42481000	-2.98909700	1.30187300
C	-3.15526600	-3.58311200	3.36147000
H	-2.77177100	-4.56657900	3.11171600
C	-3.27130300	-3.20109600	4.69859100
H	-2.98545700	-3.89063800	5.48730700
C	-3.73431100	-1.92224700	5.02076500
H	-3.79821600	-1.61084400	6.05924300
C	-4.10339100	-1.03991300	4.00777600
H	-4.43057700	-0.03657400	4.25930100
C	-4.05521000	1.30549600	1.77045900
C	-4.88125400	2.29618900	2.31919500
H	-5.92644200	2.08427000	2.51854400
C	-4.36059000	3.55833800	2.61498800
H	-5.00636800	4.31867700	3.04492100
C	-3.01682500	3.83986000	2.36712000
H	-2.61315000	4.81792000	2.60687000
C	-2.19014400	2.85966700	1.81293600
H	-1.14193500	3.06486300	1.62907600
C	-2.70579200	1.60011700	1.51127500
H	-2.06200900	0.84481600	1.07370000
Cu	-4.41979300	-0.88309700	-0.86452300
C	-6.99966100	3.53618600	-2.17860600
C	-6.25007400	2.95822800	-1.15635000
C	-5.20206200	2.11467400	-1.49803000
C	-4.90066900	1.84583200	-2.84401000
C	-5.64431100	2.48436700	-3.85010000

C	-6.70940000	3.31991900	-3.52454200
H	-6.47669900	3.18240800	-0.12022500
H	-4.58810600	1.68453000	-0.71845500
H	-5.39849000	2.31073400	-4.89456200
H	-7.30406100	3.81235400	-4.28591000
C	-3.79872700	0.95701900	-3.21153600
H	-3.32040700	1.17436300	-4.16933700
N	-3.37612100	0.00495300	-2.46024600
C	-2.10927500	-0.67001500	-2.76636600
C	-1.63382100	-0.52907100	-4.21778300
H	-0.76509200	-1.16160600	-4.38740900
H	-2.42402100	-0.83334800	-4.91269600
H	-1.34783600	0.50167100	-4.44268200
C	-2.30821600	-2.14676100	-2.39210700
O	-3.13984100	-2.54692400	-1.58573300
O	-1.46690700	-2.95026100	-3.02183700
C	-1.47472600	-4.34468400	-2.65157800
H	-0.56896400	-4.75631100	-3.09394100
H	-1.45921200	-4.44308200	-1.56616500
H	-2.36758000	-4.82854400	-3.05440700
C	-7.37331200	-0.39243400	-0.83962300
O	-8.46673100	0.00735700	-1.52407000
C	-8.15933100	-0.15526500	-2.93353600
H	-9.00584300	-0.66441800	-3.39635800
H	-8.04509900	0.84271400	-3.36259900
C	-6.85131700	-0.97896400	-2.93695400
H	-6.09242500	-0.51372000	-3.57374400
C	-7.05656500	-2.43665200	-3.39709700
H	-7.90577400	-2.83901300	-2.82362100
C	-7.41365400	-2.47059900	-4.88915300
H	-7.60705500	-3.49725500	-5.21582600
H	-8.30411800	-1.87720700	-5.12459700
H	-6.58288800	-2.08091100	-5.49163600
C	-5.82924900	-3.30646500	-3.11962800
H	-5.57571900	-3.32655100	-2.05837100
H	-6.00680800	-4.33410100	-3.45540500
H	-4.95521700	-2.92413500	-3.66075600
F	-8.02031700	4.34476700	-1.85887000

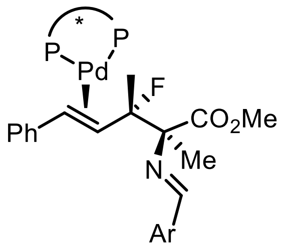

Int-7

Total SCF energy (M06-2X/def2TZVP/SMD(THF)): -4903.929208 a.u.

Thermal correction to Gibbs Free Energy at 298.15 K: 1.734965 a.u

Gibbs free energy at 298.15 K (M06-2X/def2TZVP/SMD(THF)): -4902.194243 a.u.

Pd	0.12808200	0.27799400	0.76120800
P	-0.38668000	-1.91377800	0.20208200
P	1.88969700	0.55767600	-0.75517600
O	3.93628800	-2.91546300	-2.44847300
O	1.17491800	-3.73558700	-3.58117900
C	-1.25659100	-2.86639200	1.50922500
C	-2.17082600	-3.89511700	1.25155800
H	-2.37937100	-4.16483500	0.22616700
C	-2.82471400	-4.54793300	2.29753500
C	-2.52908600	-4.15400300	3.61440500
C	-1.62110300	-3.13181400	3.90541100
C	-1.00038800	-2.48625800	2.82745000
H	-0.31833100	-1.65836500	2.99741600
C	-3.84972700	-5.66306600	2.04825500
C	-4.02484400	-5.96715500	0.54986300
H	-3.08714000	-6.30279200	0.09289300
H	-4.76299400	-6.76613700	0.41993000
H	-4.37898900	-5.09158100	-0.00518900
C	-5.21854400	-5.23090600	2.61755100
H	-5.56858700	-4.31450600	2.12959500
H	-5.96688900	-6.01527800	2.45210700
H	-5.16582800	-5.03879600	3.69414000
C	-3.38338900	-6.95601300	2.75160400
H	-3.28046700	-6.81573900	3.83244400
H	-4.10698400	-7.76309200	2.58577100
H	-2.41242800	-7.28105200	2.36153300
C	-1.30253600	-2.67343500	5.33499500
C	-1.74571000	-1.20307200	5.50206400
H	-2.82067600	-1.09481400	5.31792400
H	-1.53399200	-0.85009600	6.51843400
H	-1.21880000	-0.54677000	4.80309000
C	0.21954000	-2.77500400	5.57785800
H	0.78531000	-2.15136400	4.87906800
H	0.46575400	-2.44685600	6.59480000
H	0.56457000	-3.80823300	5.45887000
C	-2.02171500	-3.52128600	6.39622800
H	-1.74107200	-4.57824100	6.32668300
H	-1.74967400	-3.16884600	7.39710800
H	-3.11115900	-3.44870200	6.30426200
C	-1.40235700	-2.13866400	-1.29647400
C	-2.10019100	-1.03243000	-1.77917600
H	-1.98090100	-0.07922400	-1.27516500

C	-2.93417300	-1.14397800	-2.90258100
C	-3.03175700	-2.39372600	-3.51735600
C	-2.32010500	-3.52115100	-3.06734400
C	-1.50121600	-3.37513000	-1.95046800
H	-0.90420400	-4.20363500	-1.59249300
C	-2.45292500	-4.85179100	-3.81867800
C	-1.99741100	-4.65180200	-5.28025100
H	-0.95801300	-4.31137600	-5.30582100
H	-2.06940600	-5.59350900	-5.83790500
H	-2.60989200	-3.90683900	-5.79823600
C	-3.92612900	-5.31135600	-3.79067200
H	-4.59017400	-4.57503900	-4.25493200
H	-4.04227100	-6.25800400	-4.33230000
H	-4.26361900	-5.46403900	-2.75914700
C	-1.58641900	-5.95774500	-3.19335500
H	-1.89114400	-6.17411200	-2.16297300
H	-1.69481800	-6.88248400	-3.77126100
H	-0.52897800	-5.67724200	-3.18778200
C	-3.66588000	0.10197600	-3.41796900
C	-2.60926700	1.12252000	-3.89289800
H	-2.01677800	0.71297000	-4.71812400
H	-3.09364600	2.04551600	-4.23372900
H	-1.91769600	1.38023600	-3.08631600
C	-4.50614300	0.72742000	-2.28179900
H	-3.88671300	1.08029900	-1.45441900
H	-5.07005500	1.58796300	-2.66002700
H	-5.22709400	0.00246100	-1.88607100
C	-4.60199000	-0.21536800	-4.59500900
H	-5.37510200	-0.93945500	-4.31306400
H	-5.10675800	0.70094300	-4.91987100
H	-4.05465300	-0.61466900	-5.45654600
C	3.53313500	-0.02889200	-0.20944600
C	4.68650100	0.03378400	-1.00188900
H	4.60322800	0.39946200	-2.01807700
C	5.92989900	-0.33920100	-0.47643400
C	5.95941300	-0.85036600	0.83013800
C	4.81427600	-0.97765900	1.62627700
C	3.60316900	-0.52951800	1.09378800
H	2.68834100	-0.56688500	1.67245200
C	2.08527800	2.33022700	-1.17991700
C	3.29998900	3.01204000	-1.07430300
H	4.19309200	2.45731800	-0.82670600
C	3.35836900	4.40173600	-1.24102400
C	2.17172700	5.07927400	-1.53622200
C	0.93221600	4.43010800	-1.64741800
C	0.90614400	3.04799300	-1.44726800
H	-0.03769700	2.51467200	-1.45921600
C	7.24954800	-0.09793700	-1.22893600
C	8.01129000	1.03093900	-0.49630400
H	8.25834600	0.74039300	0.53020000
H	8.94736100	1.26431300	-1.01837100
H	7.40730700	1.94167500	-0.44863900
C	7.01278000	0.33612900	-2.68520000
H	6.48149000	1.29209500	-2.74416200
H	7.97386300	0.46135700	-3.19587700
H	6.43036900	-0.40935700	-3.23846700
C	8.13992100	-1.35785000	-1.24127300

H	7.68050200	-2.16514800	-1.81854000
H	9.10732900	-1.12679700	-1.70241700
H	8.33695200	-1.73373200	-0.23274600
C	4.91808000	-1.54667100	3.04715300
C	5.71496700	-0.55388900	3.92091800
H	5.21194700	0.41799300	3.94994500
H	5.80242300	-0.93079500	4.94753700
H	6.72716500	-0.40080000	3.53021300
C	3.53183600	-1.75791300	3.68279800
H	2.91440500	-2.43061000	3.07836300
H	3.64806100	-2.20420000	4.67714900
H	2.99508500	-0.81227800	3.80279300
C	5.64365300	-2.90818100	3.01224900
H	6.66431300	-2.82008300	2.62561500
H	5.70744900	-3.32885600	4.02282100
H	5.10144700	-3.61568900	2.37698300
C	4.66841300	5.19035400	-1.10652200
C	4.50981300	6.27413500	-0.01833500
H	4.26688200	5.82199200	0.94932300
H	5.44106200	6.84243500	0.09302100
H	3.71240700	6.98252200	-0.26319900
C	5.84890000	4.28644200	-0.71717700
H	6.03953900	3.52518000	-1.48131700
H	6.75920600	4.88719200	-0.61346700
H	5.67148300	3.77547000	0.23568000
C	4.99634600	5.86384600	-2.45669300
H	4.20123000	6.54896000	-2.76798700
H	5.92765200	6.43848700	-2.38306700
H	5.11833700	5.11241700	-3.24486100
C	-0.32386600	5.25003000	-1.97522000
C	-0.51690300	6.35162800	-0.91035800
H	0.34885200	7.02060400	-0.85434000
H	-1.39359300	6.96101300	-1.16035100
H	-0.68953600	5.91157800	0.07477500
C	-1.59541400	4.38621000	-2.01534400
H	-1.81389600	3.91706600	-1.05318900
H	-2.45295200	5.02030600	-2.26865300
H	-1.52495600	3.60809300	-2.78232200
C	-0.13448400	5.90099000	-3.36358200
H	-1.01702900	6.49669500	-3.62544900
H	0.73744400	6.56361400	-3.38835400
H	0.00301800	5.13616700	-4.13684900
C	1.95317900	-2.59922700	-1.23305500
C	1.14669900	-2.88221300	-0.11265700
C	1.60436000	-3.77324700	0.87165600
H	0.98153500	-3.99093300	1.73112100
C	2.85087500	-4.37473900	0.75168600
H	3.19474600	-5.06573100	1.51635700
C	3.66745500	-4.10269100	-0.34478400
C	3.21220200	-3.22732700	-1.33288600
C	5.24170300	-3.45077300	-2.55533000
H	5.22326900	-4.54479100	-2.65153900
H	5.67262500	-3.01423300	-3.45829600
C	1.56816200	-0.30930400	-2.34616100
C	1.21310300	0.41687000	-3.49097800
H	1.23812400	1.49976400	-3.46877600
C	0.82241200	-0.23905800	-4.65565900

H	0.53763600	0.33760700	-5.53125000
C	0.79832200	-1.62964900	-4.70354900
C	1.17488900	-2.35864200	-3.57559700
C	1.53917100	-1.72197200	-2.37648900
C	1.89088600	-4.35334800	-4.64469600
H	1.94851800	-5.41475200	-4.39304300
H	2.89996500	-3.93438300	-4.72016800
H	5.85025600	-3.17160400	-1.68910200
H	0.48422400	-2.14790900	-5.60307100
H	1.37536600	-4.24824400	-5.60752600
H	4.63915900	-4.57377200	-0.42124800
H	-3.67026400	-2.50309800	-4.38561100
H	6.91653800	-1.14234000	1.25034500
H	-3.03394700	-4.65812000	4.42959400
H	2.20594300	6.15494000	-1.67389200
C	-3.38604500	0.18805200	1.53853500
H	-3.35412300	-0.30327000	2.51410600
H	-4.42620500	0.37277200	1.25774900
C	-2.57156500	1.47801500	1.54807300
C	-1.19954700	1.28706500	2.14778400
H	-1.21366800	0.80525000	3.12048600
C	-0.12043800	2.13890100	1.80187100
H	-0.28696300	2.89150400	1.03794300
C	1.09028100	2.34418300	2.61156500
C	1.35795800	1.63615900	3.79933400
C	2.05255900	3.27768500	2.17477500
C	2.54115700	1.83979100	4.50573000
H	0.63647700	0.91392900	4.16861800
C	3.23876400	3.47107400	2.87698500
H	1.86554100	3.83624400	1.26290600
C	3.49520700	2.75308400	4.04791400
H	2.72151700	1.27930400	5.41963000
H	3.96806300	4.18781700	2.50847700
H	4.41763700	2.90822100	4.60018800
F	-2.40069700	1.86631300	0.19463200
H	-2.95010100	-0.49612800	0.81278800
C	-8.60922200	3.57084000	-1.46063800
C	-7.27614200	3.87683300	-1.73098200
C	-6.32002500	3.61664800	-0.75799100
C	-6.69296700	3.05555600	0.47597800
C	-8.04099700	2.76079600	0.71579200
C	-9.01166300	3.01677400	-0.25163700
H	-7.01210300	4.30846900	-2.69061800
H	-5.27058900	3.83414700	-0.92520900
H	-8.33340200	2.32435300	1.66777500
H	-10.06015600	2.79487200	-0.08535500
C	-5.67688500	2.75775800	1.49808700
H	-6.05715700	2.29339400	2.41938100
N	-4.45017700	3.01890500	1.28739100
C	-3.39919900	2.66466800	2.22517200
C	-3.85246500	2.24261000	3.63226500
H	-2.99313800	1.97424900	4.24761900
H	-4.37574800	3.06557100	4.12793000
H	-4.52505500	1.38300000	3.58969800
C	-2.47638000	3.90180800	2.28756300
O	-2.24915800	4.64288000	1.35828100
O	-1.93338900	4.06187700	3.50970300

C	-0.97604300	5.12764000	3.62253800
H	-0.69768400	5.15549300	4.67597300
H	-0.09991900	4.91090700	3.00793700
H	-1.42098500	6.07512200	3.30934500
F	-9.53866000	3.82050000	-2.40549700

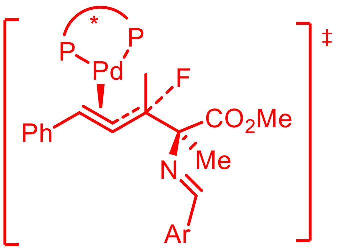

TS-8A

Total SCF energy (M06-2X/def2TZVP/SMD(THF)): -4903.885255 a.u.

Thermal correction to Gibbs Free Energy at 298.15 K: 1.735574 a.u

Gibbs free energy at 298.15 K (M06-2X/def2TZVP/SMD(THF)): -4902.149681 a.u.

Pd	0.21883600	0.55974000	0.66326600
P	-0.59678400	-1.58728000	0.09291800
P	2.11987900	0.47142300	-0.68948500
O	3.61170800	-3.34036800	-2.34790600
O	0.88458400	-3.64160600	-3.60036200
C	-1.66059200	-2.34054300	1.37582100
C	-2.91162300	-2.88759000	1.07150400
H	-3.20546800	-2.97907500	0.03587400
C	-3.79387800	-3.25536300	2.08801800
C	-3.37961800	-3.08605600	3.41913900
C	-2.12768400	-2.56236700	3.75680000
C	-1.27611600	-2.18720600	2.70990200
H	-0.31997300	-1.72037000	2.92918400
C	-5.20764700	-3.76905100	1.78585600
C	-5.44669500	-3.95925700	0.27721900
H	-4.73751500	-4.67312200	-0.15791200
H	-6.45717200	-4.34837600	0.11169200
H	-5.36100000	-3.01560300	-0.27213800
C	-6.22807900	-2.73496500	2.31230800
H	-6.07892200	-1.76372200	1.82768500
H	-7.25243800	-3.06887700	2.10767100
H	-6.13017000	-2.58740300	3.39267400
C	-5.43456700	-5.12581500	2.48533100
H	-5.32323200	-5.04900900	3.57146900
H	-6.44628000	-5.49468600	2.27959600
H	-4.71830900	-5.87341500	2.12609100
C	-1.68114500	-2.33550800	5.20712000
C	-1.30674700	-0.84855700	5.39884400
H	-2.13380700	-0.18317700	5.13180800
H	-1.03067300	-0.66041600	6.44325000
H	-0.44810600	-0.57335500	4.77875800
C	-0.44581800	-3.21644300	5.49334700
H	0.37859600	-2.98260000	4.81219500
H	-0.09090700	-3.05631700	6.51856800
H	-0.68827100	-4.27909100	5.37714900
C	-2.78233400	-2.68902200	6.21934600

H	-3.07175300	-3.74410000	6.15559200
H	-2.41832000	-2.50654700	7.23611200
H	-3.67716400	-2.07468400	6.07130200
C	-1.55477000	-1.68848200	-1.44996100
C	-2.03984400	-0.50084900	-1.99168100
H	-1.82111300	0.42937900	-1.47743200
C	-2.77725900	-0.50393600	-3.18469400
C	-3.02429000	-1.73846600	-3.78807100
C	-2.55487800	-2.95477900	-3.25902100
C	-1.80714300	-2.91244700	-2.08486800
H	-1.39360000	-3.81684300	-1.65799300
C	-2.87762100	-4.26946400	-3.97913500
C	-2.37328600	-4.19349800	-5.43593300
H	-1.29532600	-4.00747000	-5.45405400
H	-2.57168200	-5.13637600	-5.95923800
H	-2.86287200	-3.39135500	-5.99696100
C	-4.40648500	-4.48494600	-3.97080800
H	-4.93190000	-3.66628500	-4.47344100
H	-4.66524300	-5.41854000	-4.48486800
H	-4.78347000	-4.54332600	-2.94343400
C	-2.20887500	-5.47645600	-3.30041600
H	-2.57440100	-5.61757100	-2.27699700
H	-2.43750600	-6.38938100	-3.86141800
H	-1.12151500	-5.35804600	-3.26261200
C	-3.28412900	0.82348000	-3.75828000
C	-2.10121300	1.80600500	-3.89779000
H	-1.33403500	1.40338800	-4.56776400
H	-2.44989700	2.76397900	-4.30065100
H	-1.62389100	2.00757400	-2.93494400
C	-4.33543300	1.41556200	-2.79619000
H	-3.93646500	1.59143600	-1.79237500
H	-4.70553800	2.37175600	-3.18186000
H	-5.19476800	0.74360200	-2.69409000
C	-3.92909600	0.65240600	-5.14322400
H	-4.81925800	0.01516700	-5.10146600
H	-4.24408100	1.62970100	-5.52444800
H	-3.22817700	0.21942800	-5.86659100
C	3.56247100	-0.43897900	-0.03779800
C	4.74807100	-0.57791600	-0.77171400
H	4.79368700	-0.17082300	-1.77422700
C	5.86423300	-1.20180700	-0.20381100
C	5.72022700	-1.75032100	1.07899700
C	4.53171500	-1.67476000	1.81565600
C	3.46032800	-0.97944200	1.24697500
H	2.52789400	-0.85412600	1.78265700
C	2.71885800	2.15323600	-1.10074400
C	4.01927400	2.58049000	-0.81920400
H	4.73011900	1.87165600	-0.42176500
C	4.38880800	3.91919900	-1.00147600
C	3.42974700	4.80512400	-1.50199400
C	2.12110000	4.40931000	-1.81583500
C	1.77338900	3.07786200	-1.57500500
H	0.75613500	2.74315300	-1.74260800
C	7.24297900	-1.20882500	-0.88404400
C	8.19313000	-0.32385500	-0.04434400
H	8.32178800	-0.72578700	0.96599800
H	9.18177900	-0.26961800	-0.51585000

H	7.80055800	0.69277100	0.04942800
C	7.18291400	-0.64624900	-2.31386600
H	6.86487200	0.40199000	-2.32654400
H	8.17588700	-0.69529100	-2.77368200
H	6.49109200	-1.21846900	-2.94250500
C	7.83375500	-2.63208800	-0.95134800
H	7.24396900	-3.27453300	-1.61080200
H	8.85523200	-2.59500200	-1.34748700
H	7.87819500	-3.10700900	0.03353400
C	4.45487600	-2.30358800	3.21325600
C	5.38813700	-1.52059200	4.16187500
H	5.09053300	-0.46911800	4.21096000
H	5.34608100	-1.94200600	5.17367000
H	6.42895200	-1.56290700	3.82214500
C	3.02511300	-2.26450100	3.78095900
H	2.32381300	-2.78830700	3.12316400
H	3.00427000	-2.75571500	4.76009300
H	2.67144900	-1.23866600	3.91656000
C	4.90656200	-3.77809000	3.15025500
H	5.94437600	-3.87595700	2.81545100
H	4.83538400	-4.23600000	4.14374200
H	4.27398400	-4.34616300	2.46193200
C	5.79295500	4.43617400	-0.65987300
C	5.68316900	5.54628800	0.40804100
H	5.21039300	5.16777300	1.32059400
H	6.67904000	5.92315200	0.66925900
H	5.08766600	6.39313200	0.05282100
C	6.70009400	3.32274700	-0.11273500
H	6.84274800	2.52653200	-0.85110600
H	7.68686600	3.73284600	0.12778200
H	6.29168900	2.87255800	0.79855300
C	6.44928800	5.01146800	-1.93364500
H	5.86641100	5.83842000	-2.35152800
H	7.45378700	5.38873500	-1.70822500
H	6.53860300	4.23991700	-2.70662500
C	1.11988200	5.43820700	-2.36035200
C	0.73700200	6.40704600	-1.22012500
H	1.61812500	6.92524900	-0.82660600
H	0.02989200	7.16357900	-1.58005100
H	0.26546100	5.86985200	-0.39023800
C	-0.15754500	4.77354300	-2.90131600
H	-0.72346600	4.26428300	-2.11808200
H	-0.81500900	5.53661600	-3.33142800
H	0.07013200	4.04461500	-3.68724200
C	1.75912000	6.23405500	-3.51898800
H	1.03441400	6.94904100	-3.92457600
H	2.63682200	6.80342100	-3.19865600
H	2.06853000	5.56442000	-4.32939400
C	1.67587500	-2.65745700	-1.20883700
C	0.77862100	-2.79197300	-0.13298900
C	0.99630100	-3.78093600	0.83918800
H	0.29612900	-3.89684200	1.65682700
C	2.09702200	-4.62377400	0.75010400
H	2.25066100	-5.39269100	1.50176800
C	3.00574600	-4.49709200	-0.29871400
C	2.78814200	-3.52362200	-1.27521500
C	4.71044500	-4.22099900	-2.49338700

H	4.37706900	-5.25719900	-2.63827700
H	5.25015800	-3.88340100	-3.38001000
C	1.73447700	-0.32838900	-2.30512200
C	1.58713100	0.44566800	-3.46522000
H	1.80157000	1.50594200	-3.43900000
C	1.17487600	-0.13675900	-4.66026900
H	1.05661200	0.47857500	-5.54772100
C	0.92135000	-1.50356300	-4.72643400
C	1.09557200	-2.28514500	-3.58509900
C	1.47990200	-1.71584600	-2.35590200
C	1.46407200	-4.35860000	-4.68420900
H	1.40015000	-5.41496700	-4.41438900
H	2.51280100	-4.07122700	-4.81693200
H	5.37453600	-4.17306300	-1.62411800
H	0.59101100	-1.96040600	-5.65267800
H	0.91940700	-4.20366200	-5.62335600
H	3.86209800	-5.15728800	-0.34997400
H	-3.59772000	-1.76600200	-4.70641200
H	6.57661600	-2.24068900	1.53022300
H	-4.06935500	-3.34820300	4.21148900
H	3.70646800	5.84381300	-1.64525000
C	-1.87515000	3.32524000	0.19664500
H	-2.10293500	2.74203600	-0.70164000
H	-2.64827700	4.08708000	0.28650500
C	-1.93640800	2.45474900	1.41359600
C	-0.77616400	2.06198000	2.10863300
H	-0.93994700	1.57529400	3.06076600
C	0.54844500	2.44208300	1.73228000
H	0.67735900	3.20003500	0.96652000
C	1.71055900	2.30548500	2.62326000
C	1.69789300	1.51106000	3.78539600
C	2.89732800	2.99253800	2.30506600
C	2.82835600	1.40683500	4.59098900
H	0.79374100	0.98294900	4.06513700
C	4.03197900	2.87296700	3.10233700
H	2.91816400	3.62421900	1.42376800
C	4.00539400	2.07985800	4.25157200
H	2.79084100	0.79727700	5.48968700
H	4.93668400	3.41244700	2.83579800
H	4.88523600	1.99784600	4.88315700
F	-2.59910900	4.04122400	2.75883400
H	-0.90272100	3.80114800	0.07963600
C	-8.51672900	3.22763300	-1.75014900
C	-7.24600300	3.78363200	-1.87796800
C	-6.26447600	3.42360500	-0.96214700
C	-6.54432400	2.51510200	0.07112700
C	-7.83956800	1.99147500	0.18118200
C	-8.83444700	2.33720400	-0.73203400
H	-7.05077700	4.48903300	-2.67897600
H	-5.26594100	3.84089200	-1.02226700
H	-8.06756900	1.30244400	0.99038900
H	-9.84003200	1.93627400	-0.66395300
C	-5.51003100	2.11377700	1.03759100
H	-5.87822700	1.63121000	1.94699300
N	-4.27245000	2.32596300	0.81090500
C	-3.30799800	1.95176900	1.83411900
C	-3.36351600	0.43888300	2.07582300

H	-2.55363000	0.09789900	2.71292900
H	-4.30605700	0.17240400	2.56156800
H	-3.30272900	-0.08724300	1.12017100
C	-3.61867100	2.74494100	3.18674700
O	-4.74737700	3.14517600	3.44650300
O	-2.82565400	2.15261800	4.19212900
C	-2.89839000	2.85887000	5.42425200
H	-2.25403500	2.32214300	6.12619900
H	-2.53635000	3.88297600	5.28680500
H	-3.92679200	2.88693900	5.79823400
F	-9.47255600	3.57334400	-2.64001800

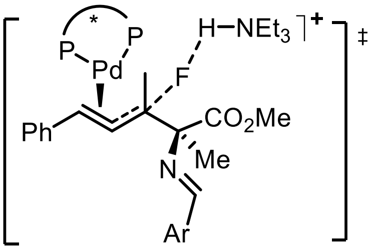

TS-8B

Total SCF energy (M06-2X/def2TZVP/SMD(THF)): -5196.768319 a.u.

Thermal correction to Gibbs Free Energy at 298.15 K: 1.948953 a.u

Gibbs free energy at 298.15 K (M06-2X/def2TZVP/SMD(THF)): -5194.819366 a.u.

Pd	-0.37548600	-0.35471700	0.50221500
P	-0.45461700	1.99809800	0.27197100
P	-2.28175300	-0.78851000	-0.77314000
O	-5.24020900	2.36931800	-1.63574500
O	-2.97336200	3.88078700	-2.91475100
C	0.36730900	2.90997100	1.63173400
C	1.06481100	4.10332500	1.43861200
H	1.10296900	4.53624700	0.44949600
C	1.72490000	4.72699100	2.50367000
C	1.66562000	4.11364600	3.76094400
C	0.98666800	2.90707500	3.98605100
C	0.33481000	2.31687700	2.90091900
H	-0.18248900	1.37019600	3.01234900
C	2.51520200	6.02972100	2.32055000
C	2.42492900	6.56631000	0.88098300
H	1.38975400	6.78302600	0.59393300
H	2.99409300	7.49826000	0.79970100
H	2.83951600	5.85916600	0.15388800
C	4.00106500	5.76566400	2.64882200
H	4.41562600	5.00234800	1.98022900
H	4.58861700	6.68293700	2.52694400
H	4.13147100	5.41768000	3.67871900
C	1.95839600	7.10837400	3.27443400
H	2.03615700	6.80460600	4.32317800
H	2.51586200	8.04451900	3.15607400
H	0.90300800	7.30955000	3.06037200
C	1.00535200	2.26687200	5.37976300
C	0.34434400	0.87798600	5.38383900
H	0.85519700	0.19605900	4.69489200
H	0.39702700	0.44638100	6.38936700
H	-0.71265200	0.92379000	5.10033200

C	0.24880900	3.17794300	6.36895400
H	-0.79816700	3.29249300	6.06705800
H	0.26937600	2.75042400	7.37826800
H	0.69572300	4.17631600	6.41616000
C	2.47027200	2.09893100	5.84008600
H	2.97802100	3.06174100	5.95118400
H	2.50857900	1.59191700	6.81148400
H	3.03365900	1.50446800	5.11301300
C	0.23035300	2.70803500	-1.26055900
C	1.07769400	1.91270900	-2.02749700
H	1.25901400	0.89411000	-1.70557900
C	1.66147600	2.40055300	-3.20515200
C	1.36994900	3.71536800	-3.57235600
C	0.50968800	4.54177600	-2.82547500
C	-0.06376300	4.01662700	-1.66907000
H	-0.76307500	4.60137600	-1.08616100
C	0.23167500	5.97282200	-3.30109800
C	-0.33997800	5.93031200	-4.73441100
H	-1.25705800	5.33435500	-4.75962100
H	-0.57489300	6.94279000	-5.08207900
H	0.36747600	5.49021900	-5.44426600
C	1.55198000	6.77333300	-3.29345900
H	2.30353300	6.32170800	-3.94936500
H	1.37808400	7.79929600	-3.63819200
H	1.97186700	6.81950300	-2.28226400
C	-0.78225000	6.69349200	-2.39702200
H	-0.40376200	6.80504700	-1.37441400
H	-0.97522700	7.69835700	-2.78742300
H	-1.73272000	6.15290000	-2.35422900
C	2.57582000	1.48179800	-4.02534500
C	1.79459900	0.20624400	-4.41040700
H	0.91169200	0.45630800	-5.00752500
H	2.43052600	-0.46953400	-4.99455800
H	1.44781300	-0.34015000	-3.52878900
C	3.80519500	1.09641300	-3.17296700
H	3.52332200	0.57406500	-2.25410300
H	4.47152000	0.43737000	-3.74193300
H	4.37718700	1.98638800	-2.88772800
C	3.07183300	2.15180200	-5.31647700
H	3.66934300	3.04576700	-5.10676800
H	3.70580700	1.45424700	-5.87424900
H	2.23947200	2.43934800	-5.96825600
C	-3.87916200	-0.62462600	0.09118600
C	-5.10035000	-0.85000100	-0.55774900
H	-5.09710600	-1.08932500	-1.61400300
C	-6.30192400	-0.79917900	0.15743200
C	-6.23804900	-0.43643400	1.51109200
C	-5.03707600	-0.15513800	2.17445200
C	-3.85040900	-0.28589600	1.44720600
H	-2.88856600	-0.11519800	1.91386800
C	-2.23195800	-2.48779200	-1.45672100
C	-3.22319800	-3.43307400	-1.17996100
H	-4.09791000	-3.12853500	-0.62483500
C	-3.07354800	-4.76793100	-1.57786200
C	-1.91667000	-5.12000700	-2.28079100
C	-0.90933800	-4.19563600	-2.59491100
C	-1.07247300	-2.88308000	-2.14493800

H	-0.29523900	-2.14863800	-2.31871700
C	-7.64716400	-1.22070200	-0.45555800
C	-8.10612300	-2.51824500	0.24941900
H	-8.26781300	-2.35477600	1.31990700
H	-9.04765500	-2.87537200	-0.18395500
H	-7.35711700	-3.30861400	0.14232600
C	-7.53194400	-1.49555600	-1.96406600
H	-6.85042100	-2.32676400	-2.17661500
H	-8.51386800	-1.76376900	-2.36780100
H	-7.17363400	-0.61405000	-2.50804700
C	-8.72691000	-0.13968200	-0.24359800
H	-8.48964300	0.77110400	-0.80021900
H	-9.69671600	-0.50494700	-0.59992500
H	-8.84236700	0.12756800	0.81125700
C	-5.05346500	0.23464800	3.65872400
C	-5.51974600	-0.98346300	4.48532300
H	-4.84840100	-1.83334700	4.33014200
H	-5.53098600	-0.73897200	5.55434900
H	-6.53072900	-1.29490300	4.20117800
C	-3.66086900	0.66538900	4.15413000
H	-3.27750300	1.51368500	3.57778000
H	-3.72311900	0.96790100	5.20556400
H	-2.93888800	-0.15303100	4.08509300
C	-6.02868700	1.40972700	3.88246100
H	-7.05665400	1.14701300	3.61337500
H	-6.02931500	1.70251600	4.93870300
H	-5.73365200	2.27646000	3.28390300
C	-4.12626500	-5.83926700	-1.26236300
C	-3.47754400	-6.96537400	-0.42810500
H	-3.07503100	-6.57554300	0.51321000
H	-4.21908400	-7.73612300	-0.18900000
H	-2.65643700	-7.44891500	-0.96688100
C	-5.31143600	-5.26669800	-0.46848700
H	-5.83470000	-4.49054700	-1.03688500
H	-6.03251500	-6.06243800	-0.25425300
H	-4.99465000	-4.83398200	0.48678600
C	-4.66711100	-6.42576800	-2.58452400
H	-3.87189900	-6.88638200	-3.17941400
H	-5.42103000	-7.19450400	-2.37971000
H	-5.13278200	-5.64394900	-3.19465300
C	0.33834000	-4.65253400	-3.36667900
C	1.22007400	-5.49801900	-2.42079600
H	0.67926800	-6.37780800	-2.05634500
H	2.12156700	-5.84412400	-2.94143100
H	1.53111300	-4.91261000	-1.54810000
C	1.16460500	-3.46496700	-3.89200000
H	1.58664700	-2.86055500	-3.08565100
H	2.00200600	-3.83714700	-4.49235400
H	0.56275900	-2.80749900	-4.52880500
C	-0.07086300	-5.50814900	-4.58489000
H	0.81986500	-5.80571200	-5.14968000
H	-0.59429600	-6.42346100	-4.29423700
H	-0.72702100	-4.94313800	-5.25573800
C	-3.08134100	2.30906700	-0.71977500
C	-2.19038800	2.60515400	0.32797300
C	-2.65179900	3.26522600	1.47739900
H	-1.96266500	3.50444800	2.27764200

C	-3.98907300	3.62638800	1.58768100
H	-4.33655100	4.14767200	2.47478900
C	-4.89142100	3.33076600	0.56804300
C	-4.43603800	2.68178400	-0.58150000
C	-6.59996500	2.76565600	-1.57213700
H	-6.69607000	3.85828800	-1.53115900
H	-7.06352700	2.39170800	-2.48641900
C	-2.38635800	0.33957500	-2.22597000
C	-2.09076800	-0.12809600	-3.51471600
H	-1.90043400	-1.18042700	-3.67900200
C	-2.05760300	0.74694800	-4.59687200
H	-1.82284000	0.37043800	-5.58844400
C	-2.33758300	2.09838700	-4.41820100
C	-2.66342800	2.56561600	-3.14515800
C	-2.67230700	1.70752400	-2.02794200
C	-3.87163600	4.49360200	-3.83576400
H	-4.18198500	5.43226500	-3.37252500
H	-4.74453400	3.85333800	-4.00144000
H	-7.09963600	2.32379600	-0.70407000
H	-2.30836000	2.78371300	-5.25783800
H	-3.39184700	4.71780500	-4.79555200
H	-5.93085900	3.61400300	0.67303000
H	1.81241900	4.11854800	-4.47473000
H	-7.16296700	-0.38892100	2.07619100
H	2.17902100	4.58404000	4.59305700
H	-1.79342700	-6.15113000	-2.59248300
C	2.31868400	-1.78377800	-0.72590200
H	1.78549900	-0.97191400	-1.22711200
H	3.29937400	-1.88571600	-1.17945100
C	2.44079400	-1.49075900	0.74718400
C	1.27756100	-1.59421600	1.56551300
H	1.38043500	-1.29990800	2.59996400
C	0.12827300	-2.35512500	1.18091000
H	0.18864800	-2.97607400	0.29203800
C	-0.88030100	-2.81876900	2.14827600
C	-1.04695900	-2.23651900	3.41919300
C	-1.72750600	-3.88443700	1.79279500
C	-2.01862000	-2.70691700	4.29813300
H	-0.41862500	-1.40466900	3.71782000
C	-2.70914500	-4.34328900	2.66741200
H	-1.60751500	-4.34975600	0.82024000
C	-2.85952900	-3.75985200	3.92706100
H	-2.12556000	-2.24582100	5.27603700
H	-3.35210300	-5.16715600	2.37069500
H	-3.61683700	-4.12439400	4.61461000
F	3.40417800	-3.02808200	1.15259300
H	1.74379500	-2.69651200	-0.87298100
C	8.83008100	-0.18568900	-2.61238300
C	7.64140700	-0.87456000	-2.85730700
C	6.61538500	-0.78608700	-1.92617700
C	6.77088700	-0.01741300	-0.75862900
C	7.97558700	0.66762600	-0.54776600
C	9.01537200	0.58717900	-1.47157700
H	7.54118500	-1.45451500	-3.76849900
H	5.67305200	-1.29666400	-2.08973100
H	8.10198600	1.26468100	0.35152500
H	9.95456100	1.10849700	-1.32392000

C	5.70687900	0.05392600	0.25066100
H	5.91148900	0.71919900	1.10073600
N	4.64052800	-0.63404300	0.14254900
C	3.59647200	-0.53462400	1.15946800
C	3.08441000	0.91738900	1.26562400
H	2.27586000	0.99566300	1.98640200
H	3.89344500	1.58708100	1.57519700
H	2.71679400	1.24915600	0.29247300
C	4.17224000	-0.98567000	2.52550500
O	5.28856800	-1.43043900	2.69967600
O	3.31019500	-0.79095400	3.53487200
C	3.73139700	-1.28803000	4.82022400
H	2.91359200	-1.05665100	5.50021600
H	3.89660000	-2.36655600	4.76466300
H	4.65128700	-0.79209200	5.13593100
F	9.82437200	-0.27340600	-3.51004300
N	5.69866900	-4.17091000	1.04005800
C	6.90873300	-3.86795900	1.89084700
H	6.52104900	-3.57689000	2.86496900
H	7.45419000	-4.81246200	1.99051100
C	6.03324700	-4.25242200	-0.42666200
H	6.41562400	-3.26806500	-0.69632200
H	6.84385400	-4.98007400	-0.53658100
C	4.99107400	-5.40739800	1.53909700
H	4.13767100	-5.55243400	0.88045600
H	5.68385200	-6.24646900	1.41843700
C	4.82837900	-4.59543900	-1.29637700
H	5.05300100	-4.32734600	-2.33321800
H	4.58283600	-5.66037000	-1.27697500
H	3.95711200	-4.02627600	-0.96840800
C	4.48996500	-5.25812100	2.96973500
H	3.86866800	-6.12533600	3.21284000
H	5.29677500	-5.21078500	3.70542800
H	3.86772000	-4.36218300	3.03486400
C	7.81090000	-2.76570100	1.35830500
H	8.57879500	-2.56985500	2.11330600
H	8.32170400	-3.03692400	0.43102700
H	7.24793600	-1.84596300	1.20941000
H	4.96312300	-3.41645100	1.13477500

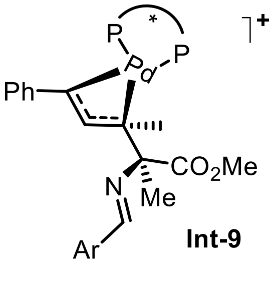

Int-9

Total SCF energy (M06-2X/def2TZVP/SMD(THF)): -4803.922314 a.u.

Thermal correction to Gibbs Free Energy at 298.15 K: 1.735221 a.u

Gibbs free energy at 298.15 K (M06-2X/def2TZVP/SMD(THF)): -4802.1807093 a.u.

Pd	0.07660900	0.68372300	0.59138700
P	-0.52820400	-1.54030500	0.01580200
P	2.05839800	0.64082600	-0.59784200

O	3.77219200	-3.16520500	-2.35288000
O	1.17758800	-3.44922100	-3.66122000
C	-1.52733400	-2.27508800	1.35808100
C	-2.62918800	-3.09400500	1.10321400
H	-2.88120600	-3.33563100	0.08059600
C	-3.41520300	-3.57787300	2.15468800
C	-3.06452500	-3.20774700	3.45861600
C	-1.97873000	-2.37027100	3.74933800
C	-1.20517400	-1.91813100	2.67551800
H	-0.36520300	-1.25268700	2.84394400
C	-4.64707500	-4.46064600	1.91750700
C	-4.84648800	-4.78682300	0.42802200
H	-3.98618700	-5.32452200	0.01321100
H	-5.72720800	-5.42540200	0.30504700
H	-5.00704000	-3.88264500	-0.16949300
C	-5.90108900	-3.71488900	2.42488100
H	-6.03075600	-2.76638200	1.89135100
H	-6.79777600	-4.32380200	2.26338100
H	-5.83471500	-3.49299100	3.49484500
C	-4.48555300	-5.78892900	2.68762700
H	-4.38307300	-5.62652900	3.76519000
H	-5.36209000	-6.42707900	2.52925100
H	-3.59956200	-6.33398200	2.34372400
C	-1.68751800	-1.97150300	5.20243100
C	-0.71052800	-0.78689800	5.28403600
H	-1.10628500	0.08188600	4.74675700
H	-0.56280200	-0.49999700	6.33071200
H	0.27201900	-1.03394900	4.87368700
C	-1.07138200	-3.18180500	5.93572300
H	-0.12688100	-3.48025300	5.46693500
H	-0.87021200	-2.93485800	6.98465600
H	-1.74599600	-4.04421000	5.91332600
C	-2.99524300	-1.54505000	5.90480700
H	-3.70792900	-2.36958000	5.99669500
H	-2.77616500	-1.19345800	6.91948600
H	-3.48345100	-0.73454500	5.35313200
C	-1.48236800	-1.74331200	-1.51895500
C	-2.03482600	-0.60261400	-2.09974200
H	-1.84167000	0.35950900	-1.63788700
C	-2.80224500	-0.69233000	-3.26989600
C	-3.02086700	-1.96544000	-3.80200800
C	-2.48289900	-3.13585300	-3.23719700
C	-1.68660700	-3.00424200	-2.09874400
H	-1.21352900	-3.86922300	-1.65168000
C	-2.83496900	-4.50535900	-3.83487000
C	-2.65674600	-4.50181300	-5.36728900
H	-1.62773700	-4.25785000	-5.64736700
H	-2.89252700	-5.49168200	-5.77286500
H	-3.31717900	-3.78204700	-5.85964500
C	-4.31593600	-4.80583700	-3.50984900
H	-4.97840700	-4.04473700	-3.93566100
H	-4.60782100	-5.77877000	-3.92201300
H	-4.48149800	-4.82906600	-2.42808900
C	-1.96255000	-5.62678700	-3.24449400
H	-2.15131500	-5.76748200	-2.17450500
H	-2.18993500	-6.57492100	-3.74246100
H	-0.89567500	-5.41681100	-3.37479500

C	-3.39732300	0.57774300	-3.89143700
C	-2.30215100	1.65887400	-4.02775500
H	-1.48273400	1.30430600	-4.66108300
H	-2.72246300	2.56464900	-4.47917900
H	-1.87272700	1.93969100	-3.06164900
C	-4.52977600	1.09109200	-2.97517800
H	-4.18056300	1.29953100	-1.95900200
H	-4.95979500	2.01338800	-3.38251800
H	-5.33385500	0.35093800	-2.89896500
C	-3.98313100	0.32147500	-5.29052000
H	-4.83249200	-0.36905900	-5.25926200
H	-4.34584200	1.26322500	-5.71539400
H	-3.23060000	-0.08773900	-5.97392200
C	3.41050300	-0.29831100	0.16521400
C	4.57810200	-0.56825100	-0.56006000
H	4.64250200	-0.25111700	-1.59297300
C	5.63687500	-1.25204200	0.04021800
C	5.46833800	-1.68130500	1.36428900
C	4.29496700	-1.46841800	2.09673800
C	3.26885900	-0.74396300	1.48113500
H	2.34536400	-0.53010700	2.00346100
C	2.72724500	2.30100500	-0.96593000
C	4.06556500	2.62610200	-0.71644000
H	4.72407800	1.87191500	-0.31369200
C	4.54308000	3.91627800	-0.96012900
C	3.64739500	4.86923400	-1.46891400
C	2.30019600	4.58297700	-1.71827800
C	1.85307800	3.28536700	-1.43909300
H	0.81014600	3.03136400	-1.59455100
C	6.95352400	-1.53858300	-0.69239700
C	8.09508700	-0.76863500	0.00633600
H	8.19699500	-1.06573000	1.05532700
H	9.05010700	-0.96607100	-0.49398300
H	7.91255500	0.31081700	-0.01951000
C	6.89340400	-1.10803100	-2.16727200
H	6.74933500	-0.02627900	-2.26931500
H	7.83510400	-1.36124700	-2.66518000
H	6.07909500	-1.61420000	-2.69654500
C	7.25413200	-3.05147200	-0.64067500
H	6.43510800	-3.61931000	-1.09079100
H	8.17384600	-3.27543500	-1.19247300
H	7.38746600	-3.40934800	0.38479600
C	4.17832200	-2.00094500	3.53058600
C	5.09983300	-1.16261800	4.44289200
H	4.81748300	-0.10558300	4.40977700
H	5.03167900	-1.50920700	5.48093300
H	6.14605100	-1.24188700	4.12857100
C	2.73549500	-1.90450700	4.05408000
H	2.03969400	-2.45506000	3.41076100
H	2.67662700	-2.33130000	5.06123900
H	2.40150000	-0.86559100	4.11503100
C	4.60571700	-3.48261800	3.58657100
H	5.65387900	-3.62240600	3.30575900
H	4.48562700	-3.86857300	4.60502900
H	3.99156700	-4.08851200	2.91327200
C	6.00464400	4.30517000	-0.70159100
C	6.06072500	5.52917800	0.23769500

H	5.58398400	5.30917500	1.19917200
H	7.10217400	5.80895300	0.43095300
H	5.55809500	6.40068000	-0.19273800
C	6.79867200	3.15991400	-0.05101100
H	6.84504400	2.27975500	-0.70152500
H	7.82706200	3.48492400	0.13700800
H	6.36236900	2.85370100	0.90653300
C	6.67118900	4.66132400	-2.04870100
H	6.16489300	5.49833300	-2.54044900
H	7.71759600	4.94657300	-1.89179500
H	6.64843300	3.80556500	-2.73250400
C	1.31237100	5.64022300	-2.23275800
C	0.37774000	6.04773500	-1.07209800
H	0.95213900	6.45559400	-0.23317400
H	-0.33326500	6.81324600	-1.40333600
H	-0.19893600	5.19456600	-0.70192300
C	0.47132600	5.06260900	-3.39198500
H	-0.13491700	4.20514400	-3.08156300
H	-0.21584500	5.82606900	-3.77259100
H	1.11354200	4.74094600	-4.21944900
C	2.02965400	6.90097600	-2.74555800
H	1.29405200	7.60075100	-3.15500800
H	2.56364200	7.42315100	-1.94472200
H	2.74565500	6.66307700	-3.54000600
C	1.80863300	-2.50969600	-1.22295600
C	0.90844200	-2.67021800	-0.15212200
C	1.13688400	-3.65558200	0.81827700
H	0.43664600	-3.78554700	1.63413600
C	2.25274300	-4.48289400	0.72585500
H	2.41831000	-5.25200300	1.47418300
C	3.15462400	-4.33782200	-0.32486300
C	2.92863300	-3.36102200	-1.29620700
C	4.35749300	-4.32429000	-2.93928500
H	3.59646200	-5.09313400	-3.11230800
H	4.77983500	-3.99720900	-3.89114500
C	1.73212500	-0.14639200	-2.23041200
C	1.53510900	0.67113700	-3.35464900
H	1.66902800	1.74161800	-3.28195200
C	1.19466000	0.11023600	-4.57939400
H	1.04784200	0.75108300	-5.44372000
C	1.05723800	-1.26891900	-4.71412500
C	1.26831600	-2.09053900	-3.60574100
C	1.58968900	-1.54438700	-2.34242100
C	1.25298000	-4.06587100	-4.93933200
H	1.29435300	-5.13962600	-4.75105800
H	2.15460100	-3.74620400	-5.47571100
H	5.16187500	-4.73916500	-2.32230100
H	0.79489800	-1.69335400	-5.67510100
H	0.37095100	-3.84633700	-5.54960500
H	4.02737800	-4.97740600	-0.38725200
H	-3.63248400	-2.05990700	-4.68971100
H	6.28400500	-2.21434000	1.83991200
H	-3.67305300	-3.57117000	4.27870700
H	4.01644600	5.86640400	-1.67144600
C	-2.09380200	2.97821300	-0.19851000
H	-2.66126300	2.38379100	-0.91384800
H	-2.60601800	3.94701200	-0.14487200

C	-2.10064000	2.34173500	1.16100000
C	-0.98338200	2.29715700	1.97242600
H	-1.11144700	1.91198200	2.97864200
C	0.35782800	2.62851000	1.54205100
H	0.46183400	3.34496500	0.73456000
C	1.49294800	2.62779400	2.47831900
C	1.50790500	1.84872800	3.65103900
C	2.62607700	3.40839900	2.18387600
C	2.61450900	1.85066700	4.49456500
H	0.65443100	1.22868400	3.90121800
C	3.73903900	3.39414400	3.02054600
H	2.62754400	4.02767900	1.29337700
C	3.74081200	2.61626000	4.17983600
H	2.60129200	1.24939300	5.39890600
H	4.60413200	4.00170600	2.77263100
H	4.60470000	2.61382100	4.83754300
H	-1.08430600	3.13009600	-0.57583500
C	-8.85427300	3.76079600	-1.40106800
C	-7.54781700	3.77462900	-1.88988800
C	-6.51654700	3.38412000	-1.04737800
C	-6.78432500	2.97969700	0.27181000
C	-8.10824700	2.98872100	0.73475800
C	-9.15417000	3.37588500	-0.09905000
H	-7.36492400	4.09390700	-2.91022200
H	-5.48943100	3.38971400	-1.39135700
H	-8.32104300	2.68743400	1.75707800
H	-10.18392700	3.38714700	0.24012900
C	-5.71046400	2.54940900	1.17052500
H	-6.02222000	2.35879500	2.20625000
N	-4.50596100	2.40615300	0.77407900
C	-3.48959800	1.91838000	1.70007300
C	-3.59879600	0.38200600	1.74892400
H	-2.82134600	-0.05336100	2.37038500
H	-4.57616800	0.09045200	2.14621200
H	-3.50436700	-0.01234700	0.73414900
C	-3.63489400	2.55656400	3.10586100
O	-4.08531900	3.65857900	3.31188900
O	-3.15813900	1.75525400	4.08548500
C	-3.17248600	2.33648300	5.40719300
H	-2.77094300	1.56749200	6.06484900
H	-2.55020200	3.23415200	5.42858900
H	-4.19314700	2.59937100	5.69148900
F	-9.85354000	4.13735400	-2.21594300

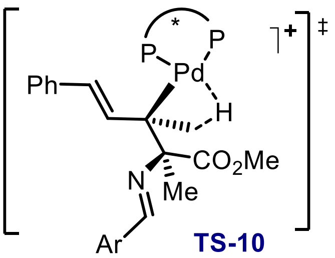

TS-10

Total SCF energy (M06-2X/def2TZVP/SMD(THF)): -4803.877028 a.u.

Thermal correction to Gibbs Free Energy at 298.15 K: 1.733791 a.u

Gibbs free energy at 298.15 K (M06-2X/def2TZVP/SMD(THF)): -4802.143237 a.u.

Pd	-0.52273200	0.44643000	-1.38810200
P	-2.31650700	0.21904900	0.06281100
P	1.04198300	-0.48920400	0.21011500
O	-0.18845300	-1.47691500	4.46647700
O	-2.34113800	-3.01822900	3.28971900
C	-3.59631700	1.49668500	-0.21666200
C	-4.90054000	1.30767800	0.26523300
H	-5.14926900	0.38624600	0.77015800
C	-5.87533800	2.28554900	0.08214300
C	-5.50910100	3.46141500	-0.59737000
C	-4.22158100	3.67975000	-1.08908300
C	-3.25958300	2.67556500	-0.88195500
H	-2.24840000	2.80662100	-1.25002800
C	-7.31431100	2.11275800	0.58479600
C	-7.52307300	0.76100000	1.28834200
H	-6.87933800	0.65761100	2.16947100
H	-8.56116900	0.67726600	1.62575100
H	-7.32344900	-0.08068800	0.61563600
C	-8.28579000	2.19025700	-0.61314900
H	-8.07170300	1.39877400	-1.33980200
H	-9.31995100	2.07042800	-0.27094000
H	-8.21570200	3.15144600	-1.13215800
C	-7.63921900	3.23982500	1.58921500
H	-7.54719700	4.23014600	1.13192700
H	-8.66613200	3.13589800	1.95742800
H	-6.96189000	3.20207400	2.44971800
C	-3.83593500	4.94647300	-1.86527800
C	-5.00320400	5.93939100	-1.98401800
H	-5.35010500	6.27709000	-1.00118700
H	-4.67730000	6.82391900	-2.54132600
H	-5.85472900	5.50676700	-2.52051800
C	-3.39599700	4.53821500	-3.28943900
H	-4.20697300	4.02354900	-3.81644000
H	-3.12497800	5.42704800	-3.87281300
H	-2.52881400	3.87226500	-3.25893500
C	-2.66445700	5.65266000	-1.14945000
H	-1.77612000	5.01815600	-1.10668900
H	-2.39873000	6.57386900	-1.68266500
H	-2.94227800	5.92503600	-0.12559900
C	-3.12869200	-1.38735800	-0.21599100
C	-2.83440100	-2.07360500	-1.39266400
H	-2.06203500	-1.67859500	-2.04350900
C	-3.50618900	-3.25770500	-1.72597900
C	-4.47754300	-3.71987100	-0.83635100
C	-4.77165600	-3.07076900	0.37699500
C	-4.07517900	-1.90240400	0.68264000
H	-4.23964400	-1.39662500	1.62493600
C	-5.85185400	-3.64730000	1.30154200
C	-5.54498800	-5.13052900	1.59951100
H	-4.55279400	-5.23969300	2.04900100
H	-6.28525400	-5.53917400	2.29623100
H	-5.56856600	-5.74501500	0.69446200
C	-7.22046200	-3.54473500	0.59305600
H	-7.22564300	-4.09375400	-0.35425100
H	-8.01125500	-3.95982000	1.22855000
H	-7.46960200	-2.49992900	0.37745600
C	-5.93287700	-2.88779200	2.63671200

H	-6.24584400	-1.84796500	2.49191000
H	-6.67368400	-3.36379700	3.28755700
H	-4.96979300	-2.88425800	3.15667700
C	-3.12414000	-4.00500200	-3.01083000
C	-1.71333400	-4.60368800	-2.82571300
H	-1.70490300	-5.31673800	-1.99488200
H	-1.39423900	-5.12858600	-3.73267500
H	-0.97163600	-3.82947000	-2.60902300
C	-3.10744900	-3.02855400	-4.20715000
H	-2.36752300	-2.23275300	-4.07198400
H	-2.84955100	-3.56593600	-5.12683500
H	-4.08722000	-2.55917800	-4.34820900
C	-4.10450600	-5.14386200	-3.33534500
H	-5.13022400	-4.77529600	-3.44832800
H	-3.81466900	-5.62288800	-4.27638300
H	-4.10007000	-5.91796100	-2.56051200
C	1.31566700	0.71526800	1.56486900
C	2.03883700	0.38556800	2.72123400
H	2.33128300	-0.64123700	2.88381300
C	2.37685900	1.36640400	3.65061800
C	1.99250900	2.69381100	3.38140600
C	1.27430200	3.05351300	2.24118000
C	0.93147900	2.03591700	1.33780100
H	0.39179000	2.27462500	0.42613300
C	2.72572200	-1.23119500	0.05345600
C	3.10164400	-2.22985400	0.96491500
H	2.38183400	-2.58302300	1.68856000
C	4.37914200	-2.78700600	0.93784600
C	5.26817000	-2.33394300	-0.04677500
C	4.92683900	-1.35192900	-0.98215000
C	3.64568100	-0.78401300	-0.89954500
H	3.35524800	0.01401100	-1.56744300
C	3.14371400	1.04354600	4.94073800
C	4.48622800	1.80634300	4.96535300
H	4.34288300	2.88642400	4.86085400
H	5.00490400	1.62774100	5.91411800
H	5.14870200	1.47725800	4.15770300
C	3.43553700	-0.46119700	5.07163300
H	4.07776800	-0.81862300	4.25833500
H	3.95592000	-0.65794300	6.01468400
H	2.51365600	-1.05229700	5.06066800
C	2.29397000	1.48735100	6.15148200
H	1.31581600	0.99670800	6.13783800
H	2.79872300	1.22807500	7.08894600
H	2.12182800	2.56822200	6.15235200
C	0.87970200	4.50105000	1.92224100
C	1.51877000	4.90848000	0.57539100
H	1.17126600	4.26935100	-0.24072000
H	1.25185000	5.94472500	0.33283100
H	2.61039500	4.83689500	0.62346500
C	-0.65617700	4.59646500	1.80986700
H	-1.13129000	4.32234200	2.75743900
H	-0.95531900	5.62010400	1.55793700
H	-1.04946100	3.93318400	1.03413800
C	1.35229800	5.48689700	3.00187900
H	2.44323600	5.48518800	3.10330600
H	1.04619100	6.50310300	2.73213200

H	0.91467000	5.25826300	3.98003900
C	4.81250000	-3.88050100	1.92218700
C	5.18537900	-5.15166100	1.12796700
H	6.00886600	-4.96774400	0.43056000
H	5.49723900	-5.95051600	1.81032100
H	4.32706800	-5.51220700	0.54929700
C	6.03785200	-3.38897500	2.72231300
H	5.79133700	-2.48967700	3.29827800
H	6.36980500	-4.16268100	3.42384200
H	6.88124000	-3.14709200	2.06784300
C	3.69591600	-4.24228500	2.91694100
H	2.80214800	-4.61958200	2.40723700
H	4.04916600	-5.02697800	3.59401200
H	3.40343100	-3.38266900	3.53077400
C	5.95101200	-0.93182900	-2.04689100
C	6.57321900	-2.18344500	-2.70408600
H	5.79804800	-2.81238000	-3.15624900
H	7.27066400	-1.88165500	-3.49326600
H	7.13429700	-2.79507600	-1.99146300
C	7.05550800	-0.09468600	-1.36605000
H	7.55177800	-0.66344500	-0.57233500
H	7.81683000	0.20143200	-2.09723300
H	6.63712100	0.81236100	-0.91907500
C	5.31439300	-0.09306200	-3.16734300
H	6.07496900	0.15190900	-3.91632400
H	4.51528300	-0.64498200	-3.67552200
H	4.90529900	0.84652600	-2.79336300
C	-1.23430000	-0.65839100	2.53163800
C	-1.96067800	0.34409000	1.86120700
C	-2.35726500	1.50096300	2.54774100
H	-2.90973100	2.27688700	2.03397300
C	-2.04624000	1.65021800	3.89551000
H	-2.35919700	2.54510600	4.42498100
C	-1.33557300	0.66378900	4.57619600
C	-0.92563500	-0.48312800	3.89340600
C	-0.28493700	-1.64852300	5.87550600
H	-1.33387700	-1.67263700	6.19357800
H	0.18926100	-2.60725500	6.09209400
C	0.10375200	-1.95896500	0.81083000
C	0.31834500	-3.15840000	0.10698900
H	1.01715900	-3.18985000	-0.72162300
C	-0.33658800	-4.31995500	0.49246900
H	-0.15895200	-5.24266600	-0.05060200
C	-1.21377400	-4.31515200	1.57808900
C	-1.46569000	-3.11968500	2.24779500
C	-0.83167200	-1.91897800	1.85330100
C	-2.54515000	-4.17458000	4.09282600
H	-3.08707900	-3.83286700	4.97629300
H	-1.58565200	-4.61389900	4.39024600
H	0.24513400	-0.86034100	6.42149700
H	-1.71420800	-5.22879700	1.87589900
H	-3.14887600	-4.93081700	3.57983400
H	-1.09369600	0.79705700	5.62381900
H	-5.02395000	-4.62212800	-1.08008600
H	2.27498400	3.46355800	4.08860700
H	-6.26499300	4.22138500	-0.74895000
H	6.26058800	-2.76856600	-0.08710600

C	-0.69318400	0.94801800	-3.90227900
C	0.65745700	0.83812200	-3.57509000
C	1.35768400	-0.44966400	-3.61863400
H	2.32068800	-0.45934200	-3.12769100
C	0.89346000	-1.59060500	-4.17682800
H	-0.03319000	-1.56552200	-4.74801500
C	1.52005800	-2.90716300	-4.08402100
C	2.50784600	-3.20728400	-3.12561700
C	1.10252300	-3.93251300	-4.95149700
C	3.06317000	-4.48056300	-3.04808800
H	2.82823900	-2.44928600	-2.42376700
C	1.66091600	-5.20658800	-4.87586200
H	0.33320300	-3.72178100	-5.69013400
C	2.64348900	-5.48690400	-3.92369700
H	3.81786500	-4.68542700	-2.29371800
H	1.32759800	-5.98257500	-5.55869800
H	3.07387000	-6.48185400	-3.86056700
C	6.12306600	3.03692400	0.99994900
C	4.99308400	2.23147900	1.11590500
C	4.09060900	2.20054600	0.06450800
C	4.30810900	2.95950600	-1.09588500
C	5.44802800	3.77689700	-1.17448500
C	6.36772400	3.81450100	-0.13002700
H	4.81992600	1.65376800	2.01417700
H	3.19269100	1.61303400	0.14780400
H	5.62165900	4.37743800	-2.06350500
H	7.25826100	4.43131800	-0.17374100
C	3.36162700	2.92303000	-2.21019600
H	3.45210400	3.75253100	-2.92338700
N	2.48737100	1.99440100	-2.34897000
C	1.52705900	2.10661200	-3.47624800
C	2.24429600	2.25482600	-4.83541800
H	1.51362600	2.33057600	-5.64544800
H	2.87728000	3.14227900	-4.86582700
H	2.86273400	1.36995700	-5.00698100
C	0.68597000	3.33850900	-3.09628300
O	-0.15704500	3.34350000	-2.21736400
O	1.06747000	4.43747500	-3.75466200
C	0.44379800	5.67333200	-3.33989900
H	-0.63084800	5.62989900	-3.52217600
H	0.62566500	5.84600700	-2.27730000
H	0.91040800	6.44658500	-3.94833100
F	6.99680000	3.07606500	2.01935600
H	-1.23289600	0.08764400	-4.28204800
H	-1.14459300	1.91127700	-4.10937400
H	-1.69729400	0.88868300	-2.41161200
